# Responsiveness to pulmonary rehabilitation in COPD is associated with changes in microbiota

**DOI:** 10.1186/s12931-023-02339-z

**Published:** 2023-01-25

**Authors:** Sara Melo-Dias, Miguel Cabral, Andreia Furtado, Sara Souto-Miranda, Maria Aurora Mendes, João Cravo, Catarina Rodrigues Almeida, Alda Marques, Ana Sousa

**Affiliations:** 1 Department of Medical Sciences, Institute of Biomedicine, University of Aveiro, 3810-193 Aveiro, Portugal; 2 Lab3R – Respiratory Research and Rehabilitation Laboratory, School of Health Sciences (ESSUA), University of Aveiro, Aveiro, Portugal; 3 Institute of Biomedicine (iBiMED), University of Aveiro, Aveiro, Portugal; 4 Department of Respiratory Medicine, Maastricht University Medical Centre, NUTRIM School of Nutrition and Translational Research in Metabolism, Faculty of Health, Medicine and Life Sciences, Maastricht University, Maastricht, The Netherlands; 5 Department of Pulmonology, Hospital Center of Baixo Vouga, Aveiro, Portugal

**Keywords:** Oral microbiota, Inflammation, COPD, Pulmonary rehabilitation, Responsiveness

## Abstract

**Background:**

Pulmonary Rehabilitation (PR) is one of the most cost-effective therapies for chronic obstructive pulmonary disease (COPD) management. There are, however, people who do not respond to PR and reasons for non-response are mostly unknown. PR is likely to change the airway microbiota and this could play a role in its responsiveness. In this study we have explored the association between PR effectiveness and specific alterations in oral microbiota and inflammation.

**Methods:**

A prospective longitudinal study was conducted. Data on exercise capacity, dyspnoea, impact of disease and 418 saliva samples were collected from 76 patients, half of whom participated in a 12-weeks PR programme. Responders and non-responders to PR (dyspnoea, exercise-capacity and impact of disease) were defined based on minimal clinically important differences.

**Results:**

Changes in microbiota, including *Prevotella melaninogenica* and *Streptococcus* were observed upon PR. *Prevotella*, previously found to be depleted in severe COPD, increased during the first month of PR in responders. This increase was negatively correlated with *Streptococcus* and *Lautropia*, known to be enriched in severe cases of COPD. Simultaneously, an anti-inflammatory commensal of the respiratory tract, *Rothia,* correlated strongly and negatively with several pro-inflammatory markers, whose levels were generally boosted by PR. Conversely, in non-responders, the observed decline in *Prevotella* correlated negatively with *Streptococcus* and *Lautropia* whose fluctuations co-occurred with several pro-inflammatory markers.

**Conclusions:**

PR is associated with changes in oral microbiota. Specifically, PR increases salivary *Prevotella melaninogenica* and avoids the decline in *Rothia* and the increase in *Streptococcus* and *Lautropia* in responders, which may contribute to the benefits of PR.

**Supplementary Information:**

The online version contains supplementary material available at 10.1186/s12931-023-02339-z.

## Introduction

Chronic obstructive pulmonary disease (COPD) is heterogenous and complex and therefore, difficult to treat and manage. Pulmonary rehabilitation (PR) is a grade A non-pharmacological therapy and one of the most cost-effective approaches for the management of COPD [1]. Compared to pharmacological treatment, PR is three to five times more effective in improving exercise capacity, dyspnoea, and quality of life [2]. Response to PR is however multidimensional and heterogeneous, which means that for the same outcome patients are not equally responsive [3]. Reasons behind non-response are mostly unknown [3, 4].

An association between the airway microbiota, including the oral microbiota [5], and disease severity has been extensively established in people with COPD [6–8]. However, the impact of PR on the airway microbiota has not yet been investigated, mainly because of the difficulty in collecting airway samples, such as sputum and bronchoalveolar lavage (BAL), routinely. Considering that only around 30% of people with COPD have productive cough [1], inducing sputum production becomes the only viable alternative. Unfortunately, this is challenging to collect outside the hospital context and requires both specialized personnel and equipment. The acquisition of BAL samples is even more challenging, as it is limited to hospital centres and bronchoscopy in patients with COPD carries a significantly higher risk of complications such as pneumonia, respiratory failure and desaturation compared with those with normal lung function [9]. In this scenario, saliva emerges as a viable non-invasive alternative to sampling the airway microbiota, which collection is simple and can be performed in different settings, namely at-home. Moreover, the oral and lower airway microbiotas are highly correlated [10], given the topological continuity between the niches, implying oral bacteria as the major colonizers of the lungs through aspiration [10–12].

An additional reason to choose saliva to evaluate the effect of PR in the airway microbiota, is the role of oral bacteria in nitrate metabolism. Exercise training, one of the main components of PR, stimulates the synthesis of nitric oxide by the human body, which is a key regulator of skeletal muscle blood flow, contractility, and mitochondrial function [13]. Oral microbiota has been strongly implicated in exercise performance, mainly due to its essential role in nitrate-nitrite-nitric oxide pathway [14, 15], with recent studies showing nitrate oral supplementation to enhance PR effectiveness [16]. The oral microbiota although acknowledged as essential for this positive effect, was never studied.

On the other hand, microbiota modifications are frequently connected with an inflammatory response, which is well known to be modulated by exercise [17] and affected by PR, although with inconsistent results [18–20].

Here, we have explored the association between PR and changes in oral microbiota and inflammatory markers of people with COPD to propose that PR effectiveness could be related, at least partially, with bacterial-driven immune regulation.

## Methods

A prospective longitudinal cohort study was conducted. Ethical approvals were obtained from Administração Regional de Saúde Centro (64/2016), Centro Hospitalar do Baixo Vouga (including Estarreja’s Hospital; 08-03-17) and Agrupamento dos Centros de Saúde do Baixo Vouga. Written informed consent was obtained from all participants.

The study was reported following the “Strengthening the Reporting of Observational Studies in Epidemiology” statement [21]. Participants were identified and referenced by clinicians who briefly explained the purposes of the study. Participants were eligible if diagnosed with COPD according to the Global Initiative for Chronic Obstructive Lung Disease (GOLD) criteria [1] and stable with no acute exacerbations in the month prior to enrolment. Exclusion criteria were presence of severe cardiac, musculoskeletal, or neuromuscular diseases, signs of cognitive impairment, active neoplasia or immune diseases, that could hinder their participation in PR.

The opportunity to be integrated in the PR programme was offered to all participants. Those who did not accept to participate in the intervention but agreed with being monitored during the 5-month period were included in the control group. The intervention group (n = 38) undertook a 12-week community-based PR program, whereas the control group (n = 38) did not.

Sociodemographic (age, sex), anthropometric (height and weight) and general clinical (smoking habits; medication, long-term oxygen use; number of acute exacerbations and hospitalizations in the past year and comorbidities—Charlson Comorbidity Index) data were collected with a structured questionnaire. Lung function was assessed with spirometry as recommended. Exercise capacity was assessed with the six-minute walk test (6MWT), a self-paced test of walking capacity. Impact of the disease was assessed with the COPD assessment test (CAT), a 8-item questionnaire, each assessed with a 6-point Likert scale. Dyspnoea at rest was assessed with the modified Borg Scale (mBorg), a 10-item scale. Clinical data (pre-post PR for the intervention group and M0-M3 for the control group) was collected with a structured protocol adapted from the team published work [22].

Saliva samples were collected monthly with the passive drool method. Prior to sample collection, patients were advised to drink a glass of water (especially if they had recently drunk coffee or citrus juice) and to provide 3–4 mL of saliva using a labelled sample collection cup. Subsequently, the sample was transported in a cooler to the lab as quickly as possible and preserved at − 80 °C until DNA extraction and/or inflammatory markers’ quantification (stored at − 80 °C for 1 to 6 months).Response/non-response to PR was determined based on published minimal clinical importance differences (MCIDs), i.e., − 1 point for mBorg [23]; 25 m for 6MWT [24] and − 2 points for CAT [25].

Deep sequencing of the V4 hypervariable region of 16S rRNA gene (F515/R806 primer pair) was performed for all samples. The bead assay LEGENDplex™ Human Inflammation Panel 1 (13-plex) (BioLegend, San Diego, CA, USA) was used to quantify inflammatory markers (IL-1β, IFN-α2, IFN-γ, TNF-α, MCP-1, IL-6, IL-8, IL-10, IL-12p70, IL-17A, IL-18, IL-23, and IL-33) in both groups in three timepoints, M0, M1 and M3. QIIME2 2020.8 [26, 27] was used to perform microbiota analyses. All statistical analyses were performed in GraphPad Prism 8 [28] and R software v 3.6.1 [29]. Longitudinal models (*lme4* package [30]) and repeated-measures correlations (*rmcorr* package [31]) were performed in R software v 3.6.1 [29]. The full description of the methodology used, including all steps of data collection, processing, data analyses and metadata, are available in Additional files 1, 2 and 3).

## Results

### Cohort characterisation

Seventy-six individuals with COPD were included in this study, 38 (29 male, 72 ± 9y, BMI: 26 ± 4 kg/m^2^, FEV_1_pp 49.2 ± 16% predicted, GOLD A-8, B-20, C-0, D-10) in the intervention group and the remaining 38 patients (31 male, 70 ± 8y, BMI: 26.4 ± 4.8 kg/m^2^, FEV_1_pp 52.3 ± 19.8% predicted, GOLD A-17, B-12, C-1, D-8) in the control group. Table 1 summarizes the baseline characteristics of the two groups. No significant differences were found between groups (see Table 1), and therefore, no adjustment for confounding factors was performed.

**Table 1:** Sociodemographic, anthropometric, and clinical characteristics of individuals with chronic obstructive pulmonary disease at beginning of the study (n = 76)

Characteristics	Intervention group (n = 38)	Control group (n = 38)	*p*-value
Age (years), mean ± SD	72 ± 9	70 ± 7.60	0.19^a^
Gender, n (%)			0.57^b^
Male	29 (76%)	31 (82%)	
Female	9 (24%)	7 (18%)	
BMI (kg/m^2^), mean ± SD	26 ± 4	26.4 ± 4.8	0.72^c^
FEV_1PP_, mean ± SD	49.2 ± 16.0	52.3 ± 19.8	0.53^a^
FVC_PP_, mean ± SD	77.8 ± 17.3	76.9 ± 27.9	0.68^a^
Pack-years, mean ± SD	42.4 ± 43.0	53.0 ± 51.2	0.27^a^
Comorbidities (CCI), mean ± SD	4.2 ± 1.4	3.8 ± 1.3	0.10^a^
GOLD grade, n (%)			0.22^d^
1	3 (8%)	6 (16%)	
2	14 (37%)	12 (32%)	
3	20 (53%)	15 (39%)	
4	1 (3%)	5 (13%)	
GOLD group, n (%)			0.069^d^
A	8 (21%)	17 (45%)	
B	20 (53%)	12 (32%)	
C	0 (0%)	1 (3%)	
D	10 (26%)	8 (21%)	
Medication, n (%)			
ICS	21 (55%)	19 (50%)	0.8^d^
LABA	33 (87%)	30 (79%)	0.36^d^
LAMA	24 (63%)	25 (66%)	> 0.99
LTRA	2 (5%)	2 (5%)	> 0.99^d^
SABA	9 (24%)	5 (13%)	0.37^d^
SAMA	2 (5%)	0 (5%)	0.49^d^
Macrolides in stability	0 (0%)	0 (0%)	> 0.99
Number of exacerbations in the last year, n (%)			0.60^b^
0 or 1	27 (71%)	29 (76%)	
≥ 2 or 1 with a hospitalisation	11 (29%)	9 (24%)	
Number of hospitalisations in the last year due to COPD, n (%)			0.67^d^
0	32 (84%)	34 (89%)	
1	2 (5%)	3 (8%)	
2	3 (8%)	1 (3%)	
Missing values	1 (3%)	0 (0%)	
mBORG, mean ± SD	0.8 ± 1.3	0.5 ± 1.0	0.3^a^
CAT, mean ± SD	17.1 ± 8.1	11.7 ± 7.4	0.003^a^
Walked distance in 6MWT, mean ± SD	389.0 ± 133.9	457.5 ± 82.8	0.045^a^

SD: standard deviation, n (%): absolute and relative frequency, respectively; BMI: body mass index, FEV_1pp_: forced expiratory volume in 1 s percent predicted, CCI: charlson comorbidity index; GOLD: global initiative for chronic obstructive lung disease; ICS: inhaled corticosteroids; LABA: long-acting beta agonists, LAMA: long-acting muscarinic agonists, LTRA: leukotriene receptor antagonists, SABA: short-acting beta agonists; SAMA: short-acting muscarinic agonists; ^a^p-value obtained with Mann Whitney U-test; ^b^ p-value obtained with Chi-square test; ^c^ p-value obtained with unpaired t-test with Welch’s correction; ^d^p-value obtained with Fisher’s exact test

Oral microbiota was similar between groups, being composed by two major phyla, Firmicutes (~ 43%) and Bacteroidetes (~ 24%), followed by Proteobacteria, Fusobacteria, Actinobacteria and ten low abundant phyla (< 2%) (Additional file 1: Fig. S1).

### Changes in oral microbiota and inflammatory response are associated with PR

We followed the temporal dynamics of beta-diversity (global rate of change (grc) of Weighted-Unifrac distance) in patients for a period of 5 months, including the 12 weeks of PR, to understand the impact of PR in microbiota composition. As a control for steady-state microbiota fluctuations, an independent cohort of patients who did not undergo PR, was surveyed for an equivalent amount of time.

**Fig. 1:**
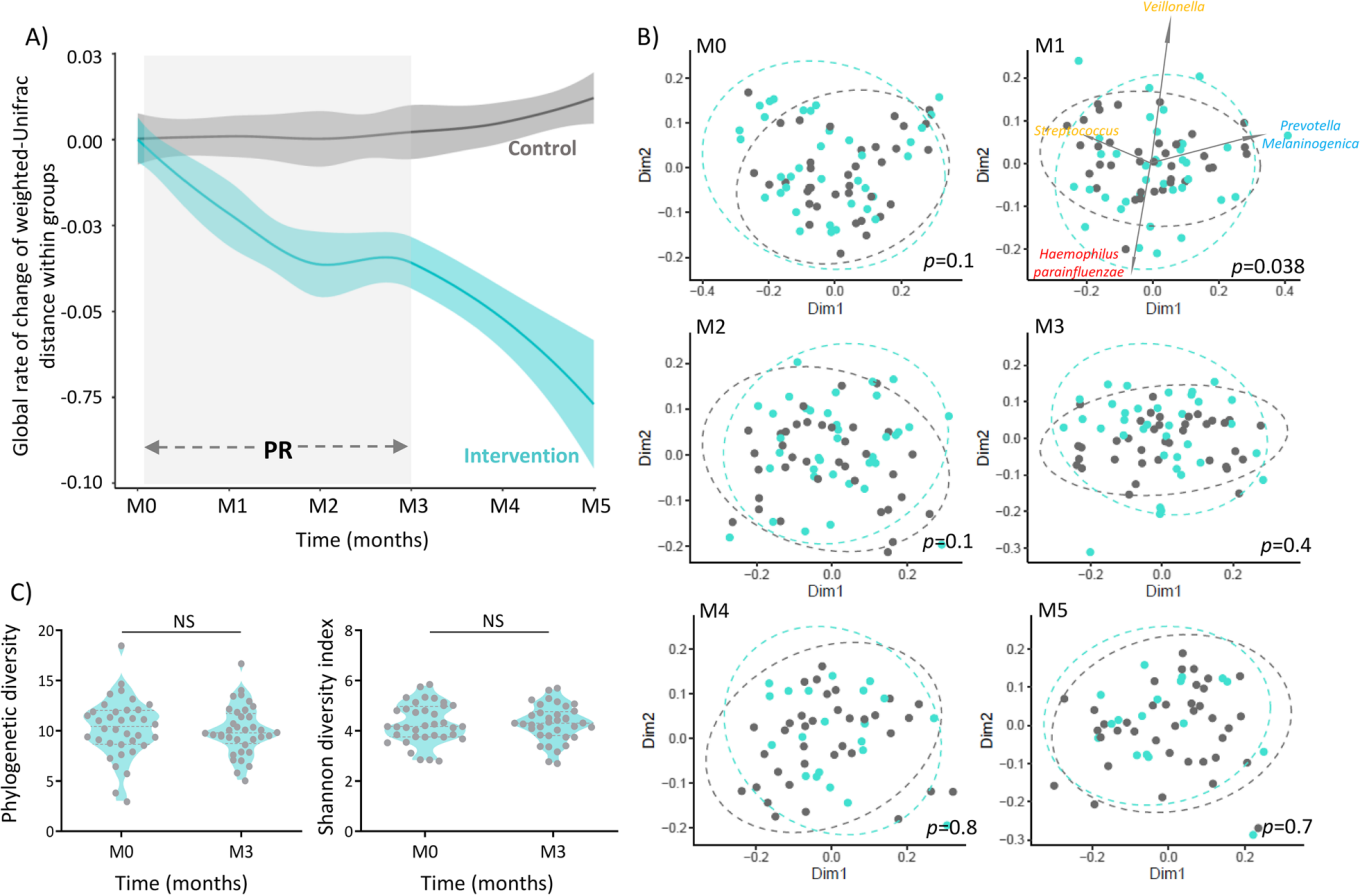
Pulmonary rehabilitation modulates the microbiota composition of people with COPD, but does not affect alpha diversity. **A** Global rate of change of beta diversity was estimated by subtraction of baseline values at all timepoints. Blue and grey loess lines were fitted over the data points representing beta-diversity relative to baseline along time in the intervention and control groups, respectively. The blue and grey areas represent the 0.95 confidence intervals. (M0 = immediately prior PR; M1, M2, M3 = 1, 2, 3 months after initiating PR; M4, M5 = 1 and 2 months after finishing PR). Intervention and control group presented different microbiota dynamics over the 5-month follow-up (LMER on Weighted Unifrac distance matrix, Group:Time-Point, p < 0.001). The grey rectangle indicates the time interval spanning PR. **B** Principal coordinate analysis of Weighted Unifrac distance between groups in each timepoint. (M0, M1, M2, M3, M4 and M5). Significant differences in microbiota composition between groups were observed in M1 (PERMANOVA, F = 2.27, *p* = 0.038). Blue and grey dots and ellipses represent intervention and control groups, respectively. The biplot represented in M1 show the top ASVs contributing for sample segregation (*5608c3e6c9de9ceb79610e7786bd0ac4: Veillonella, d0b698c7298bf04110a6d2f220879bfb: Prevotella melaninogenica, e27680d4009f98f30248d823bc17fb8e: Haemophilus parainfluenzae, a5189f77a2cfeab3bc1602ff5c8ac3e9: Streptococcus*). **C** No pre-post differences were observed in alpha diversity (Phylogenetic index and Shannon diversity index) in the Intervention group (Wilcoxon test: phylogenetic diversity: W = − 85. p = 0.48; Shannon diversity index: W = − 1 p > 0.99). *p < 0.05, **p < 0.01, ***p < 0.001

The dynamics of microbiota composition was significantly different between patients undergoing PR and controls (Fig. 1A) (lmer on Weighted-Unifrac distance (grc), Group:Time-point, F = 7.58, p < 0.001).

Principal coordinate analysis of pairwise distances (Weighted Unifrac) between groups in each timepoint showed significant differences in microbiota composition between groups upon 1 month of PR (Fig. 1B) (PERMANOVA, F = 2.3, *p* = 0.038). The top four amplicon sequence variants (ASVs) responsible for individual’s separation after the first month of PR were identified as *Veillonella*, *Prevotella melaninogenica*, *Haemophilus parainfluenzae* and *Streptococcus.*

Regarding microbiota diversity within individuals (alpha diversity), no significant changes were observed upon PR (Fig. 1C) (Wilcoxon test on phylogenetic diversity: W = − 85. p = 0.48; Wilcoxon test on Shannon diversity: W = − 1. p > 0.99).

Next, we compared the microbiota composition of the intervention group at baseline with that at M1, M2 and M3, to identify which taxa could have been affected by PR. PERMANOVA (M0vsM1: F = 0.6, *p*_*adjust*_ > 0.99; M0vsM2: F = 1.3, *p*_*adjust*_ > 0.99; M0vsM3: F = 1.2, *p*_*adjust*_ > 0.99; M0vsM4: F = 1, *p*_*adjust*_ > 0.99; M0vsM5: F = 1.4, *p*_*adjust*_ =  > 0.99), ANCOM and LEfSe detected no significant differences between pre- and post-PR microbiota composition. This is compatible with the inherent correlation among the groups (composed by the same individuals) being stronger than differences introduced by PR.

To overcome this limitation, given the longitudinal design of the experiment, we next tested for differences in taxa dynamics between the intervention and control group. This was performed using linear mixed-effect models. The temporal dynamics of the phylum Fusobacteria (lmer, Group:Time-point, F = 5.9, p = 0.02) and several oral genera, such as *Fusobacterium*, *Streptococcus*, *Dialister* and *Selenomonas* were observed to be associated with PR (Table 2). Target testing for the top four ASVs distinguishing patients on M1 was also performed. Remarkably, significant alterations in the relative abundances of *Prevotella melaninogenica* and *Streptococcus*, two ASVs previously associated with disease severity in people with COPD [5], were also found (lmer, *P. melaninogenica:* Group:Time-point, F = 4.34, p = 0.04; lmer, *Streprococcus:* Group:Time-point, F = 4.96, p = 0.03).

**Table 2:** Summary table of significant linear mixed effect models established to assess differences in microbiota dynamics between intervention and control groups

OTUs/ASVs longitudinal dynamics	Test's statistics	*p*-value (TP:Group)
Fusobacteria	5.86	0.016
*Atopobium*	5.51	0.019
*Streptococcus*	5.03	0.026
*Dialister*	6.39	0.012
*Parvimonas*	4.75	0.030
*Fusobacterium*	7.00	0.009
*a5189f77a2cfeab3bc1602ff5c8ac3e9: Streptococcus*	4.96	0.027
*d0b698c7298bf04110a6d2f220879bfb: Prevotella melaninogenica*	4.34	0.038
*e27680d4009f98f30248d823bc17fb8e: Haemophilus parainfluenzae*	0.18	0.673
*5608c3e6c9de9ceb79610e7786bd0ac4: Veillonella*	3.30	0.070

Models were produced based on the longitudinal relative frequencies of phyla, genera, and ASVs *a5189f77a2cfeab3bc1602ff5c8ac3e9, d0b698c7298bf04110a6d2f220879bfb, e27680d4009f98f30248d823bc17fb8e* and *5608c3e6c9de9ceb79610e7786bd0ac4*. Data upon relative frequencies was transformed with arcsine and square root transformation. TP: time-point

**Fig. 2:**
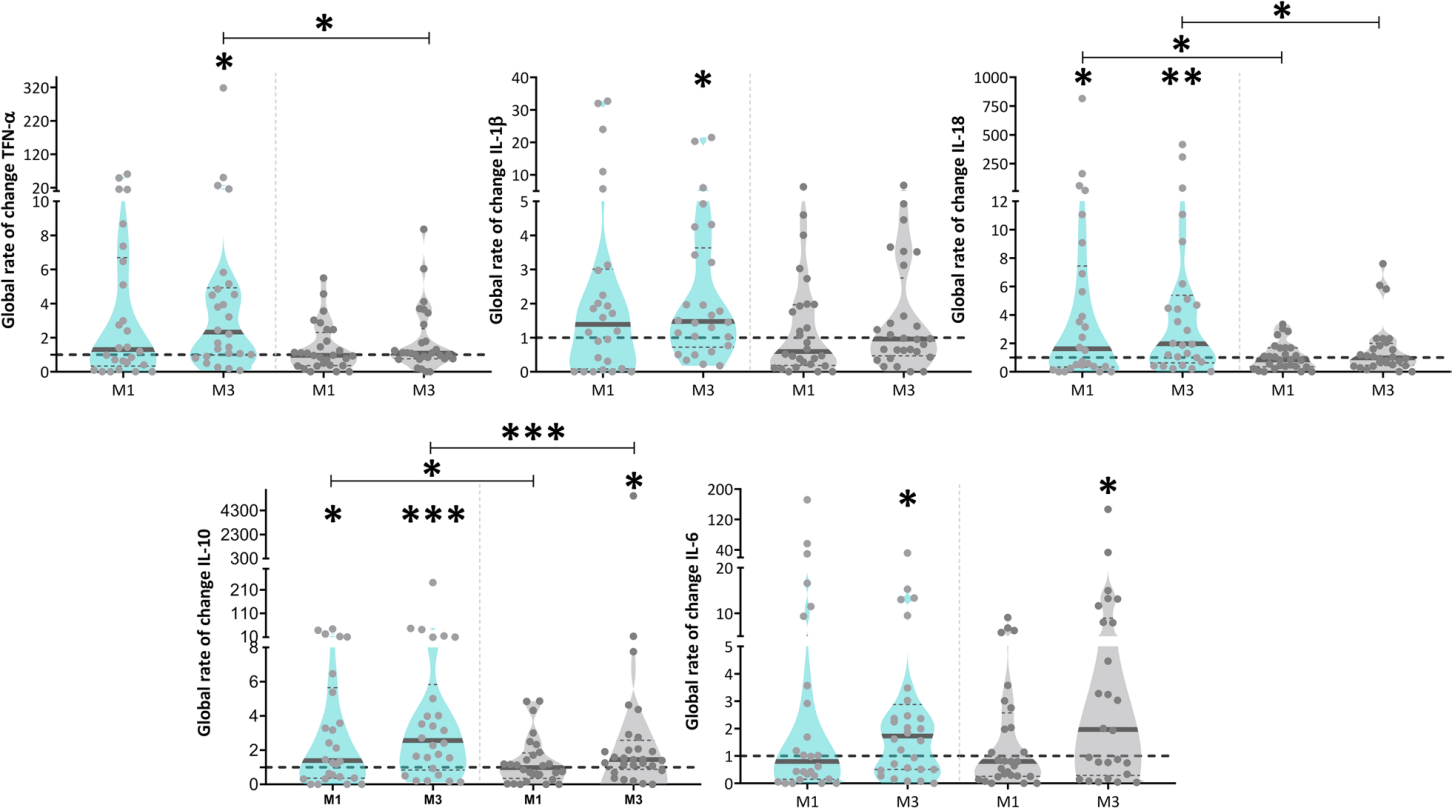
The inflammatory profile of people with COPD changes upon pulmonary rehabilitation. Global rate of change represents the ratio between cytokine values measured at baseline and M1 or M3 (i.e. M1/M0 and M3/M0). Differences between M1 and the baseline and between M3 and the baseline were assessed by Wilcoxon signed-rank, in each group (intervention (blue) and control (grey)). Differences between groups at M1 and M3 were assessed by Mann–Whitney U-test. p < 0.05, **p < 0.01, **p < 0.001. All cytokines for which a significant shift was observed are represented in this figure. Additional file 1: Fig. S2 shows the data for the remaining 8 cytokines

Considering that changes in microbiota are expected to be related with the inflammatory response, we further quantified a panel of 13 cytokines in three time points: M0, M1 and M3 in both intervention and control groups (Fig. 2 and Additional file 1: Fig. S2). Significant changes in the inflammatory profile of patients undergoing PR were observed (Fig. 2). Specifically, upon one month of PR (M1) significant increases in the amounts of IL-10 and IL-18 were observed (Wilcoxon signed-rank: IL-10, W = 163 *p*_*adjust*_ = 0.038; IL-18, W = 165 *p*_*adjust*_ = 0.035). No significant changes were observed in the same period in control group. After three months of PR (M3), TNF-α, IL-1β, IL-18, IL-10 and IL-6 were also significantly higher than at baseline (M0) (Wilcoxon signed-rank: IL-1β, W = 205 *p*_*adjust*_ = 0.016; TNF-α, W = 251 *p*_*adjust*_ = 0.002; IL-10, W = 263 *p*_*adjust*_ = 0.0008; IL-6, W = 189 *p*_*adjust*_ = 0.03; IL-18, W = 233 *p*_*adjust*_ = 0.004). IL-10 and IL-6 also increased in the control group over the same time interval (Wilcoxon signed-rank: IL-10, W = 225 *p*_*adjust*_ = 0.018; IL-6, W = 224 *p*_*adjust*_ = 0.019), suggesting that these cytokines may be less stable.

Regarding comparisons between the groups, shifts in IL-10 and IL-18 were significantly higher after 1 month of PR than the equivalent shifts in the control group (Mann–Whitney U-test, IL-10, U = 180 *p* = 0.043; IL-18, U = 173 *p* = 0.03). Over 3 months of PR, TNF-α, IL-18 and IL-10 had significantly higher shifts in the intervention than in the control group (Mann–Whitney U-test, TNF-α, U = 246 *p* = 0.04; IL-10, U = 196 *p* = 0.0004; IL-18, U = 249 *p* = 0.046). No additional significant differences were found in both groups (Additional file 1: Fig. S2).

Interestingly, the variance associated with cytokine shifts over time was generally higher in the intervention than in the control group (Additional file 1: Table S2). This is compatible with PR generally influencing cytokine levels.

### Oral microbiota and inflammatory response vary differentially among responders and non-responders during PR

PR improved all outcomes significantly (Wilcoxon test, p < 0.05), except for BMI (W = − 114, p = 0.31) and mBorg (W = 37, p = 0.5) (Additional file 1: Table S3). Mean improvements only exceeded the MCID for exercise capacity (6MWT, mean-diff: 45.43; Cohen’s d = 0.34) (Additional file 1: Table S2). Additional file 1: Fig. S3 represents the overlap between responders (R) and non-responders (NR) to PR in exercise capacity (6MWT), impact of disease (CAT) and dyspnoea (mBorg). From 38 patients, 24% responded in dyspnoea, 63% responded in exercise capacity and 63% responded in the impact of disease. 16% of patients responded simultaneously to all three domains and 16% failed to respond to any domain.

**Fig. 3:**
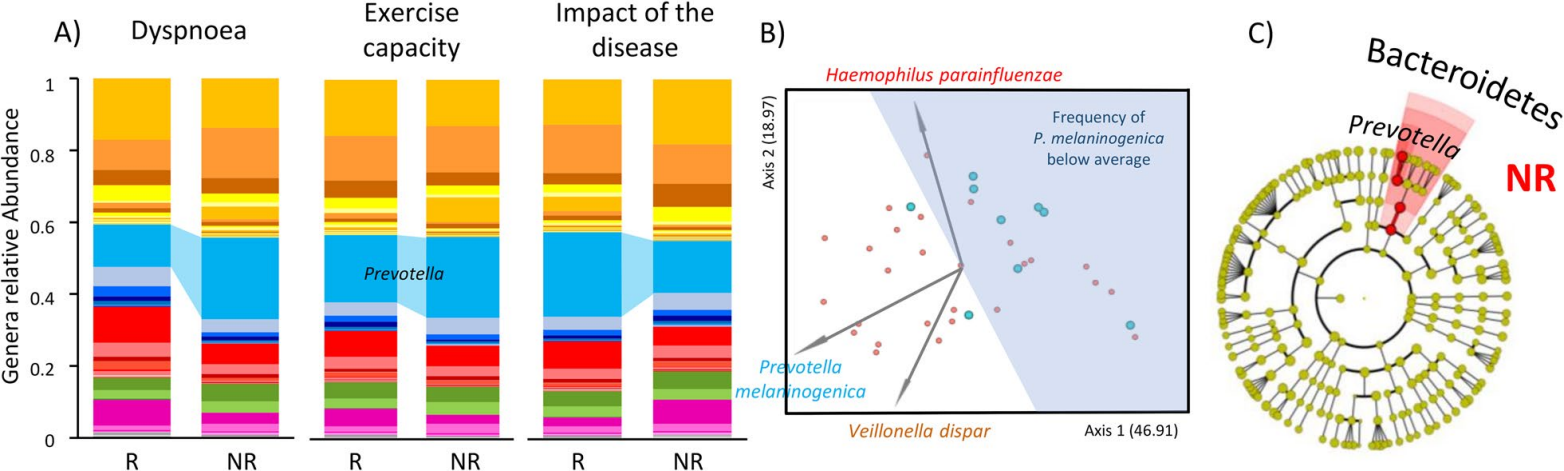
**R**esponders and non-responders present distinct microbiota profiles prior to pulmonary rehabilitation.** A** Mean frequency of phyla and genera of bacteria present in Responders (R) and non-responders (NR) to dyspnoea, exercise capacity and impact of the disease. **B** R and NR to dyspnoea showed distinct microbiota composition prior to PR (PERMANOVA, p = 0.04). PCoA analysis using Emperor on Weighted UniFrac distance. The biplot (grey arrows) represent the 3 most relevant ASVs for R and NR segregation. *Prevotella melaninogenica* (ASV: d0b698c7298bf04110a6d2f220879bfb) was the major contributor for segregation of R and NR followed by *Veillonella dispar* (ASV: 5608c3e6c9de9ceb79610e7786bd0ac) and *Haemophilus parainfluenzae* (ASV: e27680d4009f98f30248d823bc17fb8e). In blue is represented the area where samples presented a mean frequency of *P. melaninogenica* below the average of the dataset*.* 78% of R showed reduced mean frequencies of *P. melaninogenica* prior to PR while only 37% of NR followed the same trend. **C** Cladogram highlighting differentially abundant genera between R and NR to dyspnoea prior to PR, inferred by linear discriminant analysis (LEfSe) at a significance cut-off of 3. *Prevotella* is significantly enriched in NR to dyspnoea, with an effect-size of 9

We analysed differences in microbiota composition among R and NR at M0 to query for a possible relationship between microbiota composition before PR and its effectiveness (Fig. 3A). Principal coordinate analysis of pairwise distances (Weighted-Unifrac) showed significant differences between R and NR to dyspnoea (PERMANOVA p = 0.04) and captured 66% of total diversity (Fig. 3B). Furthermore, the ASV responsible for the biggest separation between individuals in this analysis, was *P. melaninogenica*, whose frequency was below average in 75% of R. In accordance, the differential abundance analysis (LEfSe) showed that *Prevotella* (Fig. 3C) was significantly enriched in NR to dyspnoea, with an effect-size of 9. No significant differences were observed for the other domains.

Next, we used linear mixed effect models to compare the microbiota temporal dynamics between R and NR. Despite being differentially represented in R and NR (to dyspnoea) before PR, the frequency trajectory of *P. melaninogenica* was not significantly different among the two sets of patients during the intervention. Instead, several other taxa were found to have significantly different trajectories between the groups (Table 3 and Additional file 1: Fig. S4). Regarding the exercise capacity, the frequency trajectory of *Lautropia* (family *Burkholderiaceae*) was significantly different between R and NR (lmer, Group:Time-point, F = 2.9, p = 0.02), reaching higher values in NR by the end of PR (Additional file 1: Fig. S3). The dynamics of *P. melaninogenica* was distinct among R and NR (lmer, Group:Time-point, F = 2.6, p = 0.03) to impact of disease (Additional file 1: Fig. S4).

**Table 3:** Summary table of significant linear mixed effect models established to assess differences in microbiota dynamics between responders and non-responders to dyspnoea, exercise capacity and impact of the disease

OTUs/ASVs longitudinal dynamics	Test's statistics	*p*-value (TP:Group)
Response to dyspnoea during exercise (Borg Modified Scale of Dyspnoea) MCID: − 1 point		
Spirochaetes	2.33	0.046
TM7	5.40	0.000
*Oribacterium*	2.68	0.024
*Dialister*	2.69	0.023
*Schwartzia*	2.55	0.036
*[Mogibacteriaceae]*	2.45	0.036
*Bulleidia*	3.08	0.011
*Treponema*	2.33	0.046
Response to exercise capacity (6-min walk test) MCID: + 25 m		
*Lautropia*	2.87	0.017
Response to impact of the disease (COPD assessment test) MCID: -2 points		
*d0b698c7298bf04110a6d2f220879bfb: Prevotella melaninogenica*	2.58	0.029

Models were produced based on the longitudinal relative frequencies of phyla, genera, *a5189f77a2cfeab3bc1602ff5c8ac3e9* and *d0b698c7298bf04110a6d2f220879bfb*. Data upon relative frequencies was transformed with arcsine and square root transformation. TP: time-point

We compared the salivary levels of cytokines present in R and NR to question whether responsiveness to PR was related with the inflammatory response. Distinct patterns of inflammatory response were observed between R and NR, in all domains (Additional file 1: Fig. S5). Significant shifts during PR were always towards values higher than the baseline and occurred mainly in NR patients. Specifically, after PR, levels of TNFα, IL-1β, IL-18 and IL-10 were higher in NR of all domains, whereas only TNF-α and IL-10 showed significant changes in R patients (please see Additional file 1: Fig. S5 for details on the statistical tests results). Interestingly, the variance in cytokine shifts after PR was also mostly higher in NR than in R (Additional file 1: Tables S4–6).

### PR effectiveness is related with specific bacteria-inflammation correlation signatures

**Fig. 4:**
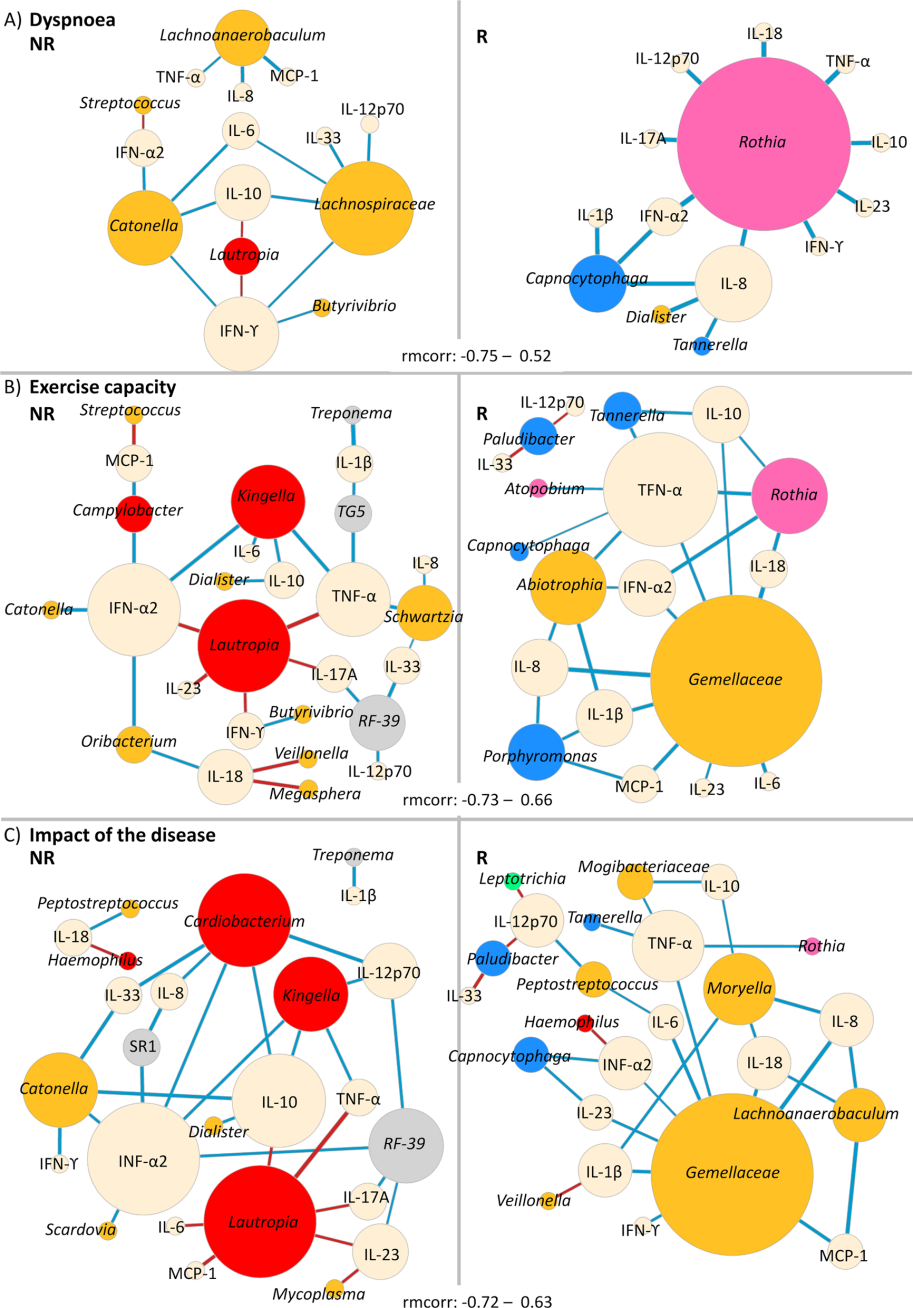
Responders and non-responders to dyspnoea, exercise capacity and impact of the disease present distinct patterns of longitudinal bacteria-inflammatory markers’ correlation. Correlation networks representing the repeated-measures correlations between bacteria and inflammatory markers in non-responders and responders to **A** dyspnoea (rmcorr: [− 0.75–0.52]) **B** exercise capacity (rmcorr: [− 0.73–0.66]) and **C** impact of the disease (rmcorr: [− 0.72–0.63]). Prior to the analysis, genera and ASVs: d0b698c7298bf04110a6d2f220879bfb and *a5189f77a2cfeab3bc1602ff5c8ac3e9* relative frequencies were transformed with arcsine square root transformation and inflammatory markers’ concentration was transformed with log_10_. The diameter of the nodes is proportional to the number of connections. Bacterial nodes are coloured with the colour code of each bacterial phyla (Firmicutes: yellow, Bacteroidetes: blue; Proteobacteria: red; Fusobacteria: green Actinobacteria: pink; other phyla: grey). Inflammatory markers’ nodes are represented in beige. Positive correlations are represented in red, while negative correlations are represented in blue. The width of edges is proportional to the correlation coefficient

Independent analyses revealed an association between PR effectiveness and either changes in oral microbiota or inflammatory markers. We explored the patterns of longitudinal correlation between the two to understand in what extent PR effectiveness is linked with specific bacteria-inflammation associations. Figure 4 represents the network of significant correlations between inflammatory markers and bacterial genera inferred for the three domains. Remarkably, R and NR presented distinct patterns of bacteria-cytokine correlation (Fig. 4).

In all groups of NR, *Lautropia* showed a strong positive correlation with several pro-inflammatory cytokines*,* being the strongest with TNF-α in NR to exercise capacity (rmcorr = 0.65, *p* = 0.002) and impact of the disease (rmcorr = 0.63, *p* = 0.002). Conversely, *Rothia* presented a negative correlation with several pro-inflammatory markers in all groups of R, with the strongest correlation with TNF-α (rmcorr = − 0.73, *p* = 0.007). Furthermore, *Gemellaceae*, was also negatively correlated with several inflammatory markers (strongest with IL-8, rmcorr = − 0.69, *p* < 0.0001) in R to exercise capacity and impact of the disease. Strikingly, except for a positive correlation between *Haemophilu*s and IFN-α2 in R to impact of the disease (rmcorr = 0.43, *p* = 0.03), all significant correlations with Proteobacteria were found in NR.

**Fig. 5:**
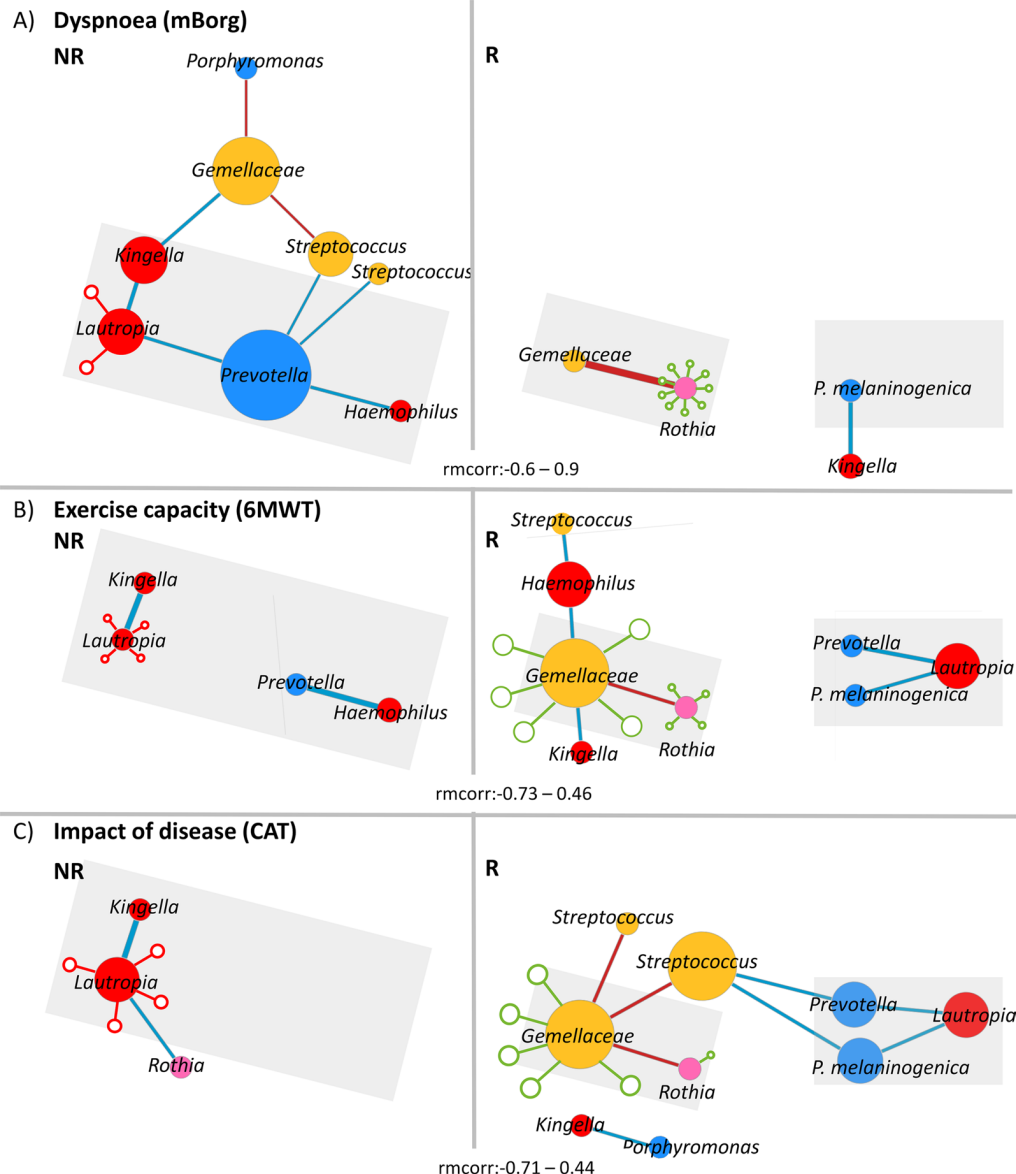
**R**esponders and non-responders to dyspnoea, exercise capacity and impact of the disease present distinct patterns of longitudinal bacteria-bacteria correlation. Correlation networks representing the longitudinal correlations between a subset of bacterial genera and ASVs: d0b698c7298bf04110a6d2f220879bfb and *a5189f77a2cfeab3bc1602ff5c8ac3e9* non-responders and responders to **A** dyspnoea (rmcorr: [ 0.6–0.9]), **B** exercise capacity (rmcorr: [− 0.73–0.46]), and **C** impact of the disease (rmcorr: [− 0.71–0.44]). Grey triangles highlight common patterns of bacterial correlations between domains. Empty blue and red circles represent cytokines with which the correspondent taxon negatively and positively correlates, respectively. These correlations were inferred through the correlation analysis between bacteria-inflammatory markers and are represented here to facilitate the interpretation of the bacteria-bacteria correlation network. Prior to the analysis, genera and ASVs: d0b698c7298bf04110a6d2f220879bfb and *a5189f77a2cfeab3bc1602ff5c8ac3e9* relative frequencies were transformed with arcsine square root transformation. The diameter of the nodes is proportional to the number of connections. Bacterial nodes were coloured according to the colour code of each bacterial phyla (Firmicutes: yellow, Bacteroidetes: blue; Proteobacteria: red; Fusobacteria: green Actinobacteria: pink; other phyla: grey). Positive correlations were represented in red, while negative correlations were represented in blue. The width of edges is proportional to the correlation coefficient

We then performed a longitudinal correlation analysis between bacterial frequencies to gain further insight on bacterial interactions that might be related with PR responsiveness but are not necessarily linked with the immune response (Fig. 5). In an effort to be conservative, since compositional data are intrinsically correlated, we chose a subset of taxa for this analysis. We included the major hubs found in the correlation between bacteria and inflammatory markers (*Lautropia*, *Rothia*, *Gemellaceae* and *Kingella*), plus five other oral taxa previously associated [5] with severity (*Prevotella*, *P. melaninogenica*, *Streptococcus*, *Streptococcus* sp., *Haemophilus* and *Porphyromonas*).

In this analysis, *Prevotella*, whose frequency drops during PR in all NR (Additional file 1: Fig. S6 A–C), was negatively correlated with *Haemophilus* (rmcorr = − 0.46, *p* = 0.005), *Streptococcus* (rmcorr = − 0.37, *p* = 0.03) and *Lautropia* (rmcorr = − 0.41, *p* = 0.01) in NR to dyspnoea and with *Haemophilus* (rmcorr = -0.73, *p* = 0.0004) in NR to exercise capacity. Conversely, during the first month of PR, the frequency of *P. melaninogenica* increased in all R (Additional file 1: Fig. S6A–C) and was negatively correlated with *Kingella* (R to dyspnoea, rmcorr = -0.59, *p* = 0.04), *Lautropia* (R to exercise capacity, rmcorr = − 0.52, *p* = 0.004; R to impact of the disease, rmcorr = − 0.43, *p* = 0.03) and *Streptococcus* (R to impact of the disease, rmcorr = − 0.41, *p* = 0.04). *Gemellaceae* and *Rothia* were strongly positively correlated in all R (strongest in dyspnoea, rmcorr = 0.9, *p* < 0.0001).

## Discussion

We show, for the first time, that oral microbiota changes with PR and that shifts in specific taxa appear to be strongly related with its effectiveness. By tracking two independent groups of patients for almost half a year we were able to distinguish between steady-state microbiota fluctuations and those possibly induced by PR. Contrary to the simple expectation that a well-established therapy should positively impact on patients’ microbiota, a more complex reality was unravelled. Overall, PR was not able to boost microbiota diversity within patients, a hallmark of dysbiosis, causing only low magnitude changes in a few taxa, and elicited the secretion of several pro-inflammatory cytokines.

However, when considering separately R and NR, taxa potentially related with responsiveness emerges and a differential inflammatory response becomes apparent. Previously, we have observed that low frequency of *Prevotella*, and in particular *P. melaninogenica*, was associated with a recent history of severe exacerbation [5]. Here we observed that in the first month of PR *Prevotella* was boosted in R of all domains. We hypothesize that this boost could be directly or indirectly related with the ability to respond. For example, by directly inducing the reduction of lung epithelial cell permeability (by modulating expression of tight junction proteins) [32], or indirectly by promoting lung innate immune responses, particularly against *Streptococcus pneumoniae* [33] and *Lautropia,* to which it was negatively correlated.

In fact, it has been recently observed that *P. melaninogenica* is able to promote *S. pneumoniae* rapid clearance from the lung via TLR2 activation, recruitment of neutrophils and upregulation of TNFα and IL-10 [34]. Interestingly, it was also observed that the aspiration of a mixture of 3 species of human oral commensals in mice, including *P. melaninogenica*, decreased host´s susceptibility to *S. pneumoniae* [33].

Supporting the hypothesis that, by counteracting *Lautropia*, *Prevotella* could be positively associated with PR effectiveness, is the observation that *Lautropia* is enriched in the oral microbiota of more severe cases of COPD [5] and co-occur with high levels of inflammatory markers in the eosinophilic COPD [35], an endotype that has been suggested to be predictive of worse outcome upon PR [36].

Conversely, in NR to dyspnoea and exercise capacity, the opposite scenario was observed, that is, a clear decline in *Prevotella* upon PR, negatively correlated with *Haemophilus* (exercise capacity) and with *Streptococcus* and *Lautropia* (dyspnoea). A pro-inflammatory role in COPD has been repeatedly attributed to the first two taxa [37] which could likely contribute to the lack of response to this therapy. Besides this, *Lautropia* positively correlated with several pro-inflammatory markers in all groups of NR.

Another striking observation, possibly related with the success of PR across the three domains, was the co-occurrence of *Rothia* and *Gemellacea* (in R) and its negative correlation with several pro-inflammatory markers during PR. *Rothia mucilaginosa* (the most abundant species of *Rothia* genus in our dataset) has been identified as an anti-inflammatory bacterium when present in the lungs (even in low abundance) of people with chronic respiratory diseases [38]. This has been attributed to its ability to reduce the levels of several pro-inflammatory cytokines and NF-kB activation in response to immune stimulation by pathogenic bacteria like *Pseudomonas aeruginosa* and *Staphylococcus aureus* [38].

Considering the impact of PR on individual species, this was significant on the longitudinal dynamics of two particular ASVs, *P. melaninogenica* and *Streptococcus*, previously suggested to be associated with COPD severity [5]. Moreover, the longitudinal dynamics of *P. melaninogenica* was distinct among R and NR to impact of the disease, supporting its importance to PR effectiveness.

Besides its immunomodulatory properties, *P. melaninogenica* could have an additional role in PR effectiveness, since it belongs to the most prevalent genus of oral nitrate-reducing bacteria [39]. These are essential for the nitrate-nitrite-nitric oxide pathway which is implicated in exercise performance and recovery [14, 15]. Coherently, it has been shown that nitrate oral supplementation increased the beneficial effects of PR [16].

Several inconsistencies have been reported regarding the effects of PR in the inflammatory status of people with COPD. Although it is unlikely that PR increases inflammation in the long-term, our findings corroborate the previous reported increase of TNF-α in plasma of people with COPD upon PR [23]. The observed increase of IL-10 suggest an attempt to counterbalance the rise of pro-inflammatory cytokines during PR, possibly via inhibition of nuclear factor kappa B (NF-kB) and the synthesis of pro-inflammatory cytokines [24].

Furthermore, it has been observed that an inflammatory response (measured by an increase in serum levels of IL-1β, TNF-α and IL-10) is necessary for recovery from exercise-induced muscle damage [27, 28]. Given the overlap between inflammatory profiles of saliva and serum [25, 26], our results could reflect a similar process.

Some limitations of our study need to be acknowledged. First, the use of saliva for COPD assessment is still exploratory. Although the microbiota of upper and lower airways is highly correlated, with oral bacteria being the major colonizers of the lungs through microaspiration [10–12], more studies, besides Melo-Dias et al. [5] validating its biological relevance in the context of COPD are needed. Additionally, despite longitudinal studies including PR being very resource demanding, further works with similar design should be carried out, ideally including multicentric trials with larger cohorts to evaluate the robustness of the findings. This is particularly important to assess the association between responsiveness and microbiota modulation. Second, clinical data collection was performed pre-post intervention and therefore it is not possible to adjust longitudinal models for multiple confounders such as occurrence of exacerbations, treatment with inhaled-corticosteroids and/or antibiotics.

Overall, besides responsiveness to PR being multidimensional and heterogeneous, giving rise to a moderate overlap in individuals response across domains, PR-induced changes in microbiota revealed surprisingly consistent patterns among R and NR. Furthermore, our findings suggest that PR effectiveness could be associated with a controlled inflammatory response to exercise, i.e., the inflammatory response to exercise occurs accompanied by efficient regulatory mechanisms. These mechanisms can be mediated by bacteria and could be tested in vitro.

Future studies should address the implications and stability of these findings, clarifying the role of oral microbiota both as a biomarker of PR responsiveness and as a therapeutic target.

## Supplementary Information

**Additional file 1.** Supplementary methods, tables and figures. **Supplementary Table 1**. Number of saliva samples collected per group in each timepoint over the 5-month period. **Supplementary Table 2**. Variance of the global rate of change estimated for each cytokine at M1 and M3 in the intervention and control groups. **Supplementary Table 3**. Summary of the effects of pulmonary rehabilitation in people with chronic obstructive pulmonary disease (n=38). **Supplementary Table 4**. Variance of the global rate of change estimated for each cytokine at M1 and M3 in R and NR to dyspnoea (mBorg).** Supplementary Table 5**. Variance of the global rate of change estimated for each cytokine at M1 and M3 in R and NR to exercise capacity (6MWT).** Supplementary Table 6**. Variance of the global rate of change estimated for each cytokine at M1 and M3 in R and NR to the impact of disease (CAT). **Supplementary Figure 1**. Mean frequency of phyla and genera of bacteria present in intervention and control groups at baseline (M0). **Supplementary Figure 2**. Violin plots representing the global rate of change of IFN-α2, IFN-ϒ, MCP-1, IL-8, IL-23, IL-33, IL-17A, IL-18 and IL-12p70 in saliva from patients submitted to the 12-week pulmonary rehabilitation programme and controls. **Supplementary Figure 3**. Venn diagram showing the percentage of overlap between patients’ (n=38) responsiveness to each domain: dyspnoea, exercise capacity and impact of the disease. **Supplementary Figure 4**. Relative frequencies over time of taxa presenting significantly different dynamics between responders and non-responders.   **Supplementary Figure 5**. Responsiveness to pulmonary rehabilitation (PR) was associated with specific alterations in the inflammatory markers. ** Supplementary Figure 6**. Frequency of Prevotella and P. melaninogenica over time in responders and non-responders to A) dyspnoea, B) exercise capacity and C) impact of the disease.

**Additional file 2.** Summary of DECONTAM results preformed with the prevalence method and a threshold of 0.5 for identification of contaminants in the dataset.

**Additional file 3.** Clinical database summarizing the response to PR per patient in each domain (dyspnoea (mBorg), exercise capacity (6MWT) and impact of disease (CAT).

## Data Availability

The dataset supporting the conclusions of this article is included within the article (and its additional file(s)). Furthermore, raw sequencing data was deposited in National Centre for Biotechnology Information’s (NCBI) Sequence Read Archive (SRA) (BioProject PRJNA872131). Scripts for data analyses are provided in Additional file 1.

## References

[1] 2022 GOLD Reports. Global Initiative for Chronic Obstructive Lung Disease - GOLD. https://goldcopd.org/2022-gold-reports-2/. Accessed 7 Jan 2022.

[2] Casaburi R (2018). Pulmonary rehabilitation: where we’ve succeeded and where we’ve failed. COPD J Chronic Obstr Pulm Dis.

[3] Augustin IML, Franssen FME, Houben-Wilke S (2022). Multidimensional outcome assessment of pulmonary rehabilitation in traits-based clusters of COPD patients. PLoS ONE.

[4] Spruit MA, Augustin IML, Vanfleteren LE (2015). Differential response to pulmonary rehabilitation in COPD: multidimensional profiling. Eur Respir J.

[5] Melo-Dias S, Valente C, Andrade L (2022). Saliva as a non-invasive specimen for COPD assessment. Respir Res.

[6] Budden KF, Shukla SD, Rehman SF (2019). Functional effects of the microbiota in chronic respiratory disease. Lancet Respir Med.

[7] Marsland BJ, Gollwitzer ES (2014). Host–microorganism interactions in lung diseases. Nat Rev Immunol.

[8] Ubags NDJ, Marsland BJ (2017). Mechanistic insight into the function of the microbiome in lung diseases. Eur Respi J.

[9] Rand IAD, Blaikley J, Booton R (2013). British Thoracic Society guideline for diagnostic flexible bronchoscopy in adults: accredited by NICE. Thorax.

[10] Gaeckle NT, Pragman AA, Pendleton KM (2020). The oral-lung axis: the impact of oral health on lung health. Respir Care.

[11] Mammen MJ, Scannapieco FA, Sethi S (2000). Oral-lung microbiome interactions in lung diseases. Periodontol.

[12] Huffnagle GB, Dickson RP, Lukacs NW (2017). The respiratory tract microbiome and lung inflammation: a two-way street. Mucosal Immunol.

[13] Dyakova EY, Kapilevich LV, Shylko VG (2015). Physical exercise associated with NO production: signaling pathways and significance in health and disease. Front Cell Dev Biol.

[14] Bescos R, Brookes ZLS, Belfield LA (2020). Modulation of oral microbiota: a new frontier in exercise supplementation. PharmaNutrition.

[15] Cutler C, Kiernan M, Willis JR (2019). Post-exercise hypotension and skeletal muscle oxygenation is regulated by nitrate-reducing activity of oral bacteria. Free Radical Biol Med.

[16] Pavitt MJ, Tanner RJ, Lewis A (2020). Oral nitrate supplementation to enhance pulmonary rehabilitation in COPD: ON-EPIC a multicentre, double-blind, placebo-controlled, randomised parallel group study. Thorax.

[17] Clauss M, Gérard P, Mosca A (2021). Interplay between exercise and gut microbiome in the context of human health and performance. Front Nutr.

[18] Gammal AIE, O’Farrell R, O’Mahony L (2015). Systemic inflammatory markers and disease severity in chronic obstructive pulmonary disease—the effect of acute exercise and pulmonary rehabilitation. Open J Respir Dis.

[19] van der Vlist J, Janssen TWJ (2010). The potential anti-inflammatory effect of exercise in chronic obstructive pulmonary disease. Respiration.

[20] Gigliotti F, Coli C, Lombardelli L (2015). Effect of pulmonary rehabilitation (PR) on inflammation status in patients with COPD. Eur Respir J.

[21] von Elm E, Altman DG, Egger M (2007). Strengthening the reporting of observational studies in epidemiology (STROBE) statement: guidelines for reporting observational studies. BMJ.

[22] Marques A, Jácome C, Rebelo P (2019). Improving access to community-based pulmonary rehabilitation: 3R protocol for real-world settings with cost-benefit analysis. BMC Public Health.

[23] Jones PW, Beeh KM, Chapman KR (2014). Minimal clinically important differences in pharmacological trials. Am J Respir Crit Care Med.

[24] Holland AE, Hill CJ, Rasekaba T (2010). Updating the minimal important difference for six-minute walk distance in patients with chronic obstructive pulmonary disease. Arch Phys Med Rehabil.

[25] Kon SSC, Canavan JL, Jones SE (2014). Minimum clinically important difference for the COPD Assessment Test: a prospective analysis. Lancet Respir Med.

[26] Bolyen E, Rideout JR, Dillon MR (2019). Reproducible, interactive, scalable and extensible microbiome data science using QIIME 2. Nat Biotechnol.

[27] QIIME 2. https://qiime2.org/. Accessed 12 Jan 2022.

[28] Prism - GraphPad. https://www.graphpad.com/scientific-software/prism/. Accessed 12 Jan 2022.

[29] R: The R Project for Statistical Computing. https://www.r-project.org/. Accessed 12 Jan 2022.

[30] Bates D, Mächler M, Bolker B (2015). Fitting linear mixed-effects models using lme4. J Stat Softw.

[31] Bakdash JZ, Marusich LR (2017). Repeated measures correlation. Front Psychol.

[32] Ramsheh MY, Haldar K, Esteve-Codina A (2021). Lung microbiome composition and bronchial epithelial gene expression in patients with COPD versus healthy individuals: a bacterial 16S rRNA gene sequencing and host transcriptomic analysis. Lancet Microbe.

[33] Wu BG, Sulaiman I, Tsay JCJ (2021). Episodic aspiration with oral commensals induces a MyD88-dependent, pulmonary T-helper cell type 17 response that mitigates susceptibility to *Streptococcus pneumoniae*. Am J Respir Crit Care Med.

[34] Horn KJ, Schopper MA, Drigot ZG (2022). Airway Prevotella promote TLR2-dependent neutrophil activation and rapid clearance of *Streptococcus pneumoniae* from the lung. Nat Commun.

[35] Wang Z, Locantore N, Haldar K (2021). Inflammatory endotype–associated airway microbiome in chronic obstructive pulmonary disease clinical stability and exacerbations: a multicohort longitudinal analysis. Am J Respir Crit Care Med.

[36] Blood eosinophils and pulmonary rehabilitation in COPD - PubMed. https://pubmed.ncbi.nlm.nih.gov/34777651/. Accessed 24 Jul 2022.

[37] Celli BR, Wedzicha JA (2019). Update on clinical aspects of chronic obstructive pulmonary disease. N Engl J Med.

[38] Rothia mucilaginosa is an anti-inflammatory bacterium in the respiratory tract of patients with chronic lung disease | European Respiratory Society. https://erj.ersjournals.com/content/early/2021/09/16/13993003.01293-2021. Accessed 12 May 2022. 10.1183/13993003.01293-2021 PMC9068977 34588194

[39] Burleigh MC, Liddle L, Monaghan C (2018). Salivary nitrite production is elevated in individuals with a higher abundance of oral nitrate-reducing bacteria. Free Radic Biol Med.

