# Supplementary material for: Responsiveness to pulmonary rehabilitation in COPD is associated with changes in microbiota
Source: Respir Res. 2023 Jan 25;24:29. doi: 10.1186/s12931-023-02339-z (PMC9875510; doi:10.1186/s12931-023-02339-z)
Supplement: Supplementary file 1 — Additional file 1. Supplementary methods, tables and figures. Supplementary Table 1. Number of saliva samples collected per group in each timepoint over the 5-month period. Supplementary Table 2. Variance of the global rate of change estimated for each cytokine at M1 and M3 in the intervention and control groups. Supplementary Table 3. Summary of the effects of pulmonary rehabilitation in people with chronic obstructive pulmonary disease (n=38). Supplementary Table 4. Variance of the global rate of change estimated for each cytokine at M1 and M3 in R and NR to dyspnoea (mBorg). Supplementary Table 5. Variance of the global rate of change estimated for each cytokine at M1 and M3 in R and NR to exercise capacity (6MWT). Supplementary Table 6. Variance of the global rate of change estimated for each cytokine at M1 and M3 in R and NR to the impact of disease (CAT). Supplementary Figure 1. Mean frequency of phyla and genera of bacteria present in intervention and control groups at baseline (M0). Supplementary Figure 2. Violin plots representing the global rate of change of IFN-α2, IFN-ϒ, MCP-1, IL-8, IL-23, IL-33, IL-17A, IL-18 and IL-12p70 in saliva from patients submitted to the 12-week pulmonary rehabilitation programme and controls. Supplementary Figure 3. Venn diagram showing the percentage of overlap between patients’ (n=38) responsiveness to each domain: dyspnoea, exercise capacity and impact of the disease. Supplementary Figure 4. Relative frequencies over time of taxa presenting significantly different dynamics between responders and non-responders. Supplementary Figure 5. Responsiveness to pulmonary rehabilitation (PR) was associated with specific alterations in the inflammatory markers. Supplementary Figure 6. Frequency of Prevotella and P. melaninogenica over time in responders and non-responders to A) dyspnoea, B) exercise capacity and C) impact of the disease. [file 12931_2023_2339_MOESM1_ESM.docx]

**Responsiveness to pulmonary rehabilitation in COPD is associated with changes in microbiota**

Sara Melo-Dias^1,2,3^, Msc, Miguel Cabral^1,3^, Msc, Andreia Furtado^1,3^, Msc, Sara Souto-Miranda^2,4^, Msc, Maria Aurora Mendes^3,5^, MD, João Cravo^5^, MD, Catarina R. Almeida^1,3^, PhD, Alda Marques^2,3^, PhDǂ and Ana Sousa^1,3^, PhDǂ

**Affiliations:**

^1^Department of Medical Sciences, University of Aveiro, Aveiro, Portugal; ^2^Lab3R – Respiratory Research and Rehabilitation Laboratory, School of Health Sciences (ESSUA), University of Aveiro, Aveiro, Portugal; ^3^Institute of Biomedicine (iBiMED), University of Aveiro, Aveiro, Portugal; ^4^Department of Respiratory Medicine, Maastricht University Medical Centre, NUTRIM School of Nutrition and Translational Research in Metabolism, Faculty of Health, Medicine and Life Sciences, Maastricht University, Maastricht, The Netherlands.; ^5^Department of Pulmonology of the Hospital Center of Baixo Vouga

ǂThese authors contributed equally to this study.

**Correspondence:** Ana Sousa, Department of Medical Sciences, Institute of Biomedicine, University of Aveiro, 3810-193 Aveiro, Portugal (amsousa@ua.pt)

**ADDITIONAL FILE**

# Methods

## Participants and sample collection

The pulmonary rehabilitation (PR) program was composed by twice a week 60-minute sessions of moderate exercise training and psychoeducational sessions once every other week. Detailed description of the PR program can be found elsewhere [1]. Patients were followed monthly for 5 consecutive months. Thirty-eight patients undertook a 12-week community-based PR program, intervention group, and the remaining 38 integrated the control group. In intervention group the time frame encompasses 1 month before PR, 3 months during PR and 2 months after PR. The PR program was delivered by a multidisciplinary team of healthcare professionals. During this period sociodemographic, anthropometric, clinical data were collected at M0 and M3 (corresponding to pre-post intervention) and saliva samples (monthly, passive drool method) were collected using a structured protocol [1].

Sociodemographic (age, sex, educational level), anthropometric (weight and height to compute body mass index), clinical (smoking habits, number of exacerbations and hospitalizations in the past year, past 3 months and past month, medication used, long-term oxygen, comorbidities - Charlson Comorbidity Index [2], level of airway obstruction-spirometry (FEV_1_, FVC, FEV_1_pp) (MicroLab 3535, CareFusion, Kent, UK) [3,4], medication including long term oxygen therapy, impact of the disease – COPD Assessment Test (CAT) [5,6], exercise capacity – six-minute walk test (6MWT) [7,8] data were collected following a published structured protocol of the team [1].  Dyspnoea at rest was assessed with the modified Borg Scale (mBorg) which is a 10-item scale. GOLD grades were defined according to FEV1 percentage predicted for each individual. GOLD groups were defined combining the number of exacerbations and hospital admissions of each patient in the year before enrolment with their CAT scores. From the initially predicted 456 samples a total of 418 saliva samples were collected, the number of samples collected per group and timepoint can be found in table S1. That is, our study has a 9% rate of missing saliva samples, particularly in the last timepoints M4 and M5. This can be largely explained by difficulties in reaching patients and keeping them motivated to collaborate in data collection for a long period.

*Response to PR*

Response/non-response to PR was determined with the published minimal clinical importance differences (MCIDs) for the modified Borg scale-Dyspnoea (mBorg), 6MWT and COPD assessment test (CAT) were: -1 point [9] , 25m [10] and -2 points [11], respectively.

## DNA extraction

Prior to DNA extraction, samples were thawed at room temperature and centrifuged at 10,000xg for 10 minutes. Supernatants were saved for subsequent quantification of inflammatory markers and pellets were used for DNA extraction following QIAamp DNA Mini Kit (Qiagen, Hilden, Germany) protocol with minor modifications: initial sample volume was set to 400µL and the volumes of buffers and Qiagen protease were adjusted. Elution volume was reduced to a quarter of the recommended. Thirty-eight negative controls where saliva was replaced by phosphate-buffered saline were performed in order to control for background bacterial contamination. Quality and quantity of the extracted DNA was assessed in Denovix DS-11 spectrophotometer, with OD260/280 and OD260/230 ratios.

## 16S rRNA gene amplification and sequencing


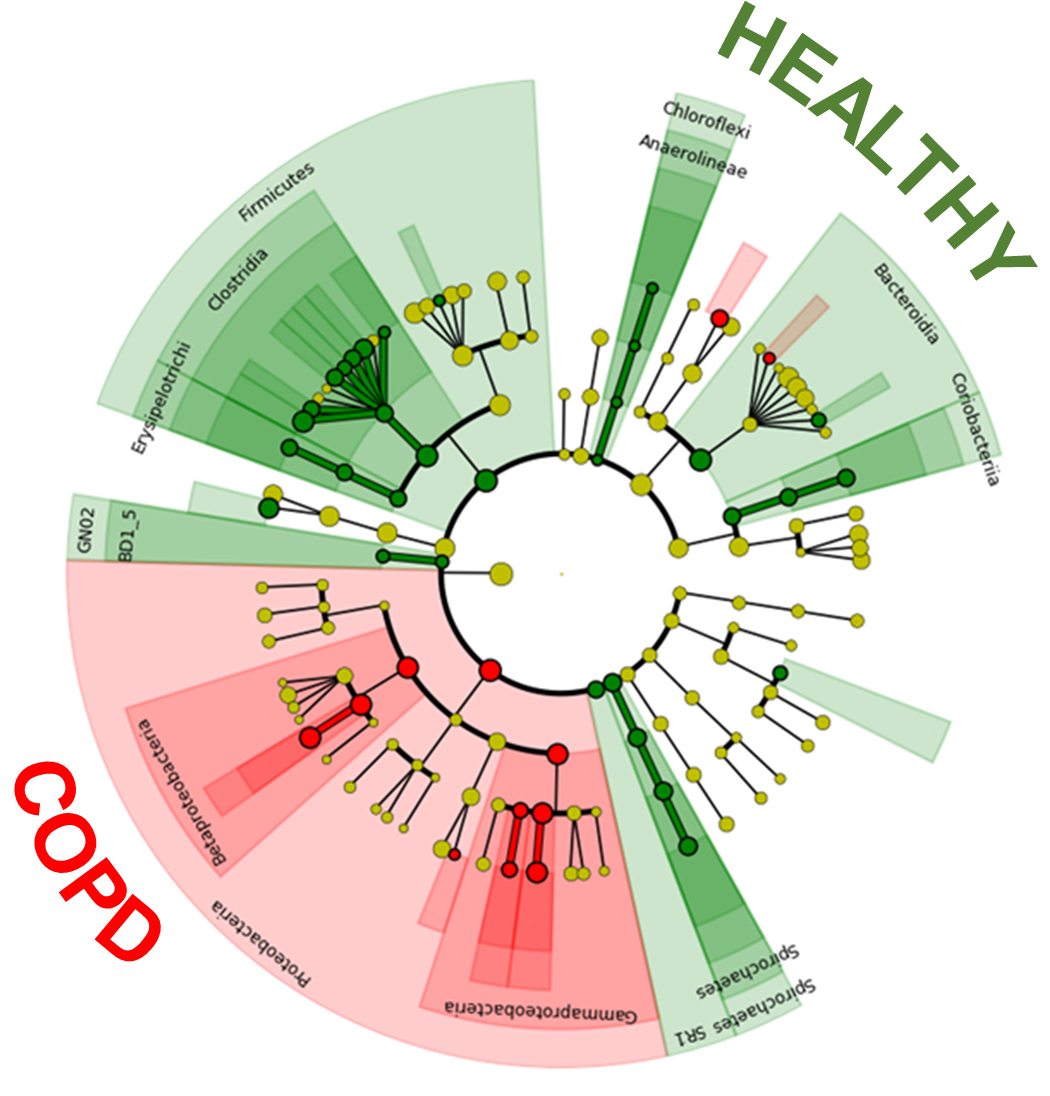
16S rRNA gene amplification and sequencing was carried out at the Gene Expression Unit from Instituto Gulbenkian de Ciência following the implemented protocol. Briefly, for each sample, the hypervariable V4 region of 16S rRNA gene was amplified, using universal pair of primers F515 (5′-CACGGTCGKCGGCGCCATT-3′) / R806 (5′-GGACTACHVGGGTWTCTAAT-3′). Samples were then pair-end-sequenced on an Illumina MiSeq Benchtop Sequencer, following Illumina recommendations.

## Quantification of inflammatory markers in saliva samples

The bead assay LEGENDplex™ Human Inflammation Panel 1 (13-plex) with V-bottom Plate (BioLegend, San Diego, CA, USA), previously used successfully with saliva samples [12,13], was used to quantify inflammatory markers (IL-1β, IFN-α2, IFN-γ, TNF-α, MCP-1, IL-6, IL-8, IL-10, IL-12p70, IL-17A, IL-18, IL-23, and IL-33) in oral samples from intervention and control groups.. Specifically, the supernatants obtained after sample centrifugation for 10 min at 10.000g, from the intervention (at baseline (M0), after 1 month of PR (M1), and after 3 months of PR (M3)) and control groups (in the timepoints M0, M1 and M3), were used for cytokine quantification following manufacturer’s recommendations [14]. Data acquisition was performed with BD AccuriTM C6 Plus flow cytometer and analysed with online version of LEGENDplex™ Data Analysis Software Suite [14]. For values below the detection limit an estimate was obtained using the standard curve. For values above the threshold of detection, the value corresponding to the maximum detection limit was used to replace cytokine values.

## Microbiota, inflammatory markers, and statistical analyses

*Sample characterisation (relative to Table 1)*

Descriptive statistics was used to characterize clinical date of intervention and control groups at baseline. Data normality was assessed with Shapiro-Wilk and D’Agostino-Pearson omnibus normality tests, ensuring the assumptions of parametric statistics approach. Comparisons between intervention and control group were conducted with unpaired t-test with Welch’s correction, when quantitative data followed normal distribution, Mann-Whitney U-test, when quantitative data violated the assumptions of parametric tests and Chi-square test, when qualitative data was considered (statistical analyses were performed in GraphPad Prism 8 [15] and R software v 3.6.1 [16]). Statistical significance was considered for p-values below 0.05.

*Analysis of illumina paired-end reads*

QIIME2 2020.8 [11,17] was used to perform microbiota analyses. Demultiplexed 16s paired-end sequences were imported and q2-vsearch plugin [18] was applied to join forward and reverse reads. Quality control was assessed via q-score base filtering, chimera removing and 16S-denoising with Deblur [19,20]. Next, to exclude bacterial background contaminations, we used the *DECONTAM* package [21,22] from *R* software [16] with the prevalence method and a threshold of 0.5, meaning that ASVs were classified as contaminants if present in a higher fraction of negative controls than true samples. This allowed us to identify 235 contaminant ASVs (available in Additional file 2), that together with ASVs from *mitochondria*, *chloroplasts* and *cyanobacteria*were removed from the dataset prior to conducting subsequent analyses. Results from previous steps were summarized in a feature table. Q2-phylogeny plugin [23] was next employed to produce a MAFFT alignment [24] of ASVs which was later used to construct a rooted phylogeny with FastTree2 [25] for subsequent applications.

Taxonomy assignment of ASVs was performed with q2-feature-classifier plugin [26,27], through classify-sklearn method with pre-trained Naïve Bayes classifier against 99%-eHOMD_v15.1 reference database [28] (sequences trimmed to only include 250bp of V4 region, bound by the F515/R806 primer pair).

Categorized samples’ metadata used for analyses is supplied in Additional file 3. Subsequent analyses were performed upon ASVs. Differential abundance analyses were conducted with on both ASVs and OTUs at taxonomic level 6.

*Diversity analyses*

Alpha- and beta-diversities were estimated with q2-diversity plugin [29] after rarefaction to 8000 sequences per sample (subsample without replacement). Spatial dissimilarities between bacterial communities of different groups and/or periods were assessed with principal coordinate analyses (PCoA) and biplots based on Weighted Unifrac distance. Wilcoxon test and Friedman’s test with Dunn’s correction were employed to compare alpha diversity among groups, periods and/or timepoints (statistical analyses were performed in *GraphPad Prism 8* [15] and *R stats* package [30] from *R* software [16]). Statistical significance was considered for p-values below 0.05.

Differences in beta-diversity between groups, periods and/or time points were quantified by permutational multivariate analysis of variance (PERMANOVA) [31] conducted with *adonis2* function [32] (*vegan* package [33] from *R* software [16]). For all statistical analyses a p-value of ≤0.05, corrected for multiple testing whenever necessary, was considered statistically significant.

*Differential abundance analyses of OTUs*

Analysis of composition of microbiomes (ANCOM) [34] and Linear discriminant effect size (LEfSe) analysis [35] were performed to identify differentially abundant OTUs between groups, periods and/or time points. LEfSe is an algorithm for high-dimensional biomarker discovery that uses linear discriminant analysis to estimate the effect size of each taxon and does not account for the compositional nature of the microbiota. ANCOM uses a log ratio analysis to make point estimates of the variance and mean, taking into consideration the compositional nature of the data. These analyses were conducted with the feature table collapsed at genus taxonomic level (L6). LEfSe was performed in the online version [36] with an Linear Discriminant Analysis score of 3 for significance. ANCOM was performed in *R* software with *ANCOM 2.0* script [37] with taxa-wise multiple correction and a W cut-off of significance of 0.7, both recommended by the developer, based on simulation data.

*Longitudinal analyses (linear mixed-effects models)*

- *Longitudinal differential abundance analyses of OTUs with ANCOM II*

Longitudinal differential abundance analyses were carried out between patients undergoing PR and controls, or R and NR to exercise capacity, dyspnoea and impact of the disease, with analysis of composition of microbiomes (ANCOM II) [38]. Similar to classic ANCOM, ANCOM II uses a log ratio analysis to make point estimates of the variance and mean [39], taking into consideration the compositional nature of the data, but comprises an additional step to deal with different types of zeros, which was further described by Kaul et al [38]. These analyses were conducted with the feature table collapsed at genus taxonomic level (L6) as well as with the feature table not collapsed, e.g., using only ASVs. ANCOM II was performed in R software [16] using ANCOM 2.1 script [40] with taxa-wise multiple correction and a W cut-off of 0.7 significance (both recommended by the developer, based on simulation data).

- *Data normalization and transformation*

To evaluate the global rate of change of beta diversity of patients undergoing PR and controls, all the timepoints of both groups were normalized through subtraction of baseline values.

- Differences in the longitudinal dynamics of OTUs/ASVs’ frequencies between groups and between R and NR to exercise capacity, dyspnoea, and impact of the disease were assessed based on the arcsine square root transformed relative frequencies in each timepoint. Additionally, raw data was filtered so that the analysis included only ASVs/OTUs that were present in ≥20% of the samples. *Loess Lines*

Loess Lines plots were produced to observe dissimilar tendencies during the 5 consecutive months (M0 to M5) of the study between patients undergoing PR and controls or R and NR to exercise capacity, dyspnoea and impact of the disease. In brief, Loess regressions were fitted over longitudinal data beta-diversity and ASVs/OTUs’ relative frequencies and then plotted with ggplot2 package from R software [16].

- *Linear mixed-effects models*

Longitudinal analyses with linear mixed-effects models, were performed to compare the longitudinal dynamics of beta- diversity (Weighted Unifrac distances) and relative frequencies of genera and 4 ASVs (top 4 ASVs responsible for patients’ separation in principal coordinate analysis), between patients undergoing PR and controls. Differences in the longitudinal trajectories of genera/ASVs’ relative frequencies between R and NR to exercise capacity, dyspnoea and impact of the disease were also evaluated. Regarding the models for beta diversity, independent variables included the timepoints (5 months) and the experimental design groups (intervention and control). Subjects were incorporated in the model as random factors, using *lmer* function of the *lme4* package [41] of R software [16]. P-values were obtained using the *Anova* function from *R-stats* package [30] and statistically significant results were considered valid if the assumptions of the model were validated.

To assess the effect of PR in the dynamics of each OTU/ASVs, the arcsine square root transformed frequencies of each OTU/ASV were defined as dependent variable in the correspondent model. Moreover, the experimental design groups (intervention and control or R and NR to exercise capacity, dyspnoea and impact of the disease) and timepoints were set as independent variables. Subjects were adjusted as a random factor. P-values were obtained using the *Anova* function and statistically significant results were considered valid if the assumptions of the model were validated. Additionally, contrast analysis with the Bonferroni correction was executed to identify the timepoints in which the mean abundance of relevant OTUs/ASVs is significantly different between R and NR to the several domains. Statistical significance was considered for p-values below 0.05.

*Analyses of inflammatory markers*

To assess changes of each inflammatory marker, the ratio between each timepoint and the baseline (M1/M0 and M3/M0) was estimated. Wilcoxon Signed-Ranks Test (GraphPad Prism 8) with post-hoc false discovery rate (FDR) correction from multiple comparisons was applied to compare ratios at M1 and M3 to baseline in all experimental groups. Mann-Whitney U-test was used to assess the differences in the median ratio of each inflammatory marker per time-point (M1 vs M1 and M3 vs M3, respectively), among the experimental groups. Z-test for variance (*PairedData* package [42] from *R* software [16]) were executed to assess differences in the variance of ratios of each inflammatory marker per time-point (M1 vs M1 and M3 vs M3, respectively), among the experimental groups. Statistical significance was considered for *p-values* below 0.05.

*Repeated Measures Correlations*

Repeated measures correlations were performed to assess the longitudinal co-variation of the frequency of genera/ASVs and inflammatory markers during PR, in the groups of R and NR to exercise capacity, dyspnoea and impact of the disease. Correlations were computed with Rmcorr function from *rmcorr* package [43] (R software [16]). Prior to correlation analyses, estimated values of inflammatory markers were transformed with log_10_ transformation, OTUs list was filtered to consider only OTUs that were present in at least 20% of the samples. Furthermore, the filtered dataset was transformed with arcsine square root transformation. Patients with only two longitudinal measurements, M0 and M3, were also included in the analyses. Statistical significance was considered for p-values below 0.05, however results were only accepted as valid if the assumptions of the *rmcorr* model were validated (namely, model residuals normally distributed and centered in zero). Valid correlations, according to previous criteria, were then plotted in correlation network plots with *igraph* package ([44] from R software [16], with node diameter proportional to the number of correlations and the edges’ width proportional to the correlation strength.

The same analysis (including timepoints M0, M1 and M3) with a subset of bacterial genera and ASVs was carried out to assess bacterial interactions that could be related with PR responsiveness. Since compositional data is intrinsically correlated the subgroup of bacterial genera/ASVs included the major hubs found in the correlation between bacteria and inflammatory markers (*Lautropia*, *Rothia*, *Gemellaceae* and *Kingella*) and six other oral taxa previously associated with severity [45] (*Prevotella*, *P. melaninogenica*, *Streptococcus*, *Streptococcus* sp., *Haemophilus* and *Porphyromonas*).

# Additional Tables

**Table S1 –**  Number of saliva samples collected per group in each timepoint over the 5-month period.

| Time-points | Intervention  (n=38) | Control  (n=38) |  |
| --- | --- | --- | --- |
| M0 | 38 | 38 |  |
| M1 | 37 | 38 |  |
| M2 | 38 | 38 |  |
| M3 | 37 | 38 |  |
| M4 | 22 | 38 |  |
| M5 | 18 | 38 |  |
| Total | 190 | 228 | 418 |

**Table S2 –**  Variance of the global rate of change estimated for each cytokine at M1 and M3 in the intervention and control groups. Z-test for variance was performed to assess significant differences between the groups.

| Time-point | Cytokine | Intervention (n=26) | Control (n=28) | test’s statistics | *p-value* |
| --- | --- | --- | --- | --- | --- |
| M1 | IL-1β | 89.5 | 2.5 | 36.1 | <0.0001 |
|  | IFN-α2 | 149.0 | 0.2 | 743.2 | <0.0001 |
|  | IFN-ϒ | 92.4 | 2.7 | 34.4 | <0.0001 |
|  | TNF-α | 220.3 | 2.0 | 111.4 | <0.0001 |
|  | MCP-1 | 1242.3 | 3.5 | 357.2 | <0.0001 |
|  | IL-6 | 1214.6 | 5.8 | 210.3 | <0.0001 |
|  | IL-8 | 1796.4 | 3.5 | 517.0 | <0.0001 |
|  | IL-10 | 126.0 | 2.0 | 63.3 | <0.0001 |
|  | IL-12p70 | 3.6 | 2.6 | 1.4 | 0.4 |
|  | IL-17A | 12.5 | 0.3 | 45.9 | <0.0001 |
|  | IL-18 | 25885.7 | 0.9 | 28096.0 | <0.0001 |
|  | IL-23 | 180.8 | 1.4 | 130.2 | <0.0001 |
|  | IL-33 | 3.1 | 0.5 | 5.9 | <0.0001 |
| M3 | IL-1β | 29.2 | 3.0 | 9.8 | <0.0001 |
|  | IFN-α2 | 19.3 | 0.1 | 151.9 | <0.0001 |
|  | IFN-ϒ | 61.3 | 0.4 | 152.8 | <0.0001 |
|  | TNF-α | 3867.6 | 3.7 | 1050.3 | <0.0001 |
|  | MCP-1 | 6062.1 | 737803.0 | 0.008 | <0.0001 |
|  | IL-6 | 828.6 | 47.7 | 17.4 | <0.0001 |
|  | IL-8 | 1002.9 | 208.3 | 4.8 | 0.0001 |
|  | IL-10 | 2248.1 | 1085998.9 | 0.002 | <0.0001 |
|  | IL-12p70 | 5.6 | 0.6 | 9.5 | <0.0001 |
|  | IL-17A | 28.0 | 0.3 | 97.2 | <0.0001 |
|  | IL-18 | 9716.7 | 3.5 | 2750.1 | <0.0001 |
|  | IL-23 | 87.0 | 652.1 | 0.1 | <0.0001 |
|  | IL-33 | 2.2 | 0.2 | 13.7 | <0.0001 |
| *p-values were* obtained with Z-test for variance | | | | | |

**Table S3 –**  Summary of the effects of pulmonary rehabilitation in people with chronic obstructive pulmonary disease (n=38)

| **Clinical Parameters** | mean Pre (n=38) | SD | mean Post (n=38) | SD | p-value | Mean Difference | Cohen’s D |
| --- | --- | --- | --- | --- | --- | --- | --- |
| BMI | 26.03 | 4.3 | 25.8 | 4.1 | 0.07 | -0.28 | 0.31 |
| mBorg | 0.79 | 1.2 | 1.1 | 1.7 | 0.5 | 0.28 | 0.18 |
| CAT | 17.08 | 8.03 | 13.5 | 7.4 | 0.001 | -3.6 | 0.46 |
| 6MWT: walked distance in m | 389 | 132.2 | 434.4 | 134.2 | 0.0006 | 45.4 | 0.34 |
| BMI: Body Mass Index; mBorg: modified Borg Scale of Dyspnoea; CAT: COPD assessment test; 6MWT: six-minute walk test | | | | | | | |

**Table S4 –**  Variance of the global rate of change estimated for each cytokine at M1 and M3 in R and NR to dyspnoea (mBorg). Z-test for variance was performed to assess significant differences between R and NR.

| Time-point | Cytokine | NR (n=19) | R (n=7) | test’s statistics | *p-value* |
| --- | --- | --- | --- | --- | --- |
| M1 | IL-1β | 98.6 | 76.4 | 0.8 | 0.8 |
|  | IFN-α2 | 202.9 | 1.3 | 0.006 | <0.0001 |
|  | IFN-ϒ | 123.4 | 3.0 | 0.02 | 0.0002 |
|  | TNF-α | 290.7 | 12.4 | 0.04 | 0.0008 |
|  | MCP-1 | 10.3 | 4627.5 | 447.5 | <0.0001 |
|  | IL-6 | 203.6 | 4113.9 | 20.2 | <0.0001 |
|  | IL-8 | 16.1 | 6696.4 | 416.0 | <0.0001 |
|  | IL-10 | 163.9 | 10.0 | 0.06 | 0.002 |
|  | IL-12p70 | 4.6 | 0.2 | 0.05 | 0.001 |
|  | IL-17A | 16.5 | 0.3 | 0.02 | <0.0001 |
|  | IL-18 | 1436.0 | 94746.6 | 66.0 | <0.0001 |
|  | IL-23 | 245.6 | 1.4 | 0.006 | <0.0001 |
|  | IL-33 | 3.8 | 1.1 | 0.3 | 0.14 |
| 344M3 | IL-1β | 20.2 | 53.2 | 2.6 | 0.1 |
|  | IFN-α2 | 24.0 | 8.1 | 0.3 | 0.18 |
|  | IFN-ϒ | 18.2 | 161.6 | 8.9 | 0.0003 |
|  | TNF-α | 5239.5 | 329.0 | 0.06 | 0.003 |
|  | MCP-1 | 2.9 | 22412.2 | 7796.1 | <0.0001 |
|  | IL-6 | 1108.1 | 128.0 | 0.1 | 0.013 |
|  | IL-8 | 10.1 | 3717.1 | 367.9 | <0.0001 |
|  | IL-10 | 2999.4 | 369.2 | 0.1 | 0.02 |
|  | IL-12p70 | 6.6 | 3.4 | 0.5 | 0.42 |
|  | IL-17A | 32.4 | 18.3 | 0.6 | 0.49 |
|  | IL-18 | 4862.1 | 24570.4 | 5.1 | 0.007 |
|  | IL-23 | 29.7 | 227.1 | 7.6 | 0.0007 |
|  | IL-33 | 2.5 | 1.9 | 0.8 | 0.79 |
| *p-values were* obtained with Z-test for variance | | | | | |

**Table S5 –**  Variance of the global rate of change estimated for each cytokine at M1 and M3 in R and NR to exercise capacity (6MWT). Z-test for variance was performed to assess significant differences between R and NR.

| Time-point | Cytokine | NR (n=10) | R (n=16) | test's statistics | *p-value* |
| --- | --- | --- | --- | --- | --- |
| M1 | IL-1β | 99.9 | 88.9 | 0.9 | 0.81 |
|  | IFN-α2 | 382.1 | 2.9 | 0.008 | <0.0001 |
|  | IFN-ϒ | 199.1 | 27.8 | 0.1 | 0.001 |
|  | TNF-α | 491.3 | 19.1 | 0.04 | <0.0001 |
|  | MCP-1 | 17.9 | 2024.1 | 113.0 | <0.0001 |
|  | IL-6 | 349.4 | 1811.8 | 5.2 | 0.017 |
|  | IL-8 | 29.7 | 2918.0 | 98.3 | <0.0001 |
|  | IL-10 | 263.4 | 7.3 | 0.03 | <0.0001 |
|  | IL-12p70 | 7.2 | 1.1 | 0.15 | 0.002 |
|  | IL-17A | 29.5 | 1.4 | 0.05 | <0.0001 |
|  | IL-18 | 2534.3 | 41015.4 | 16.2 | 0.0002 |
|  | IL-23 | 467.2 | 1.9 | 0.004 | <0.0001 |
|  | IL-33 | 5.3 | 1.7 | 0.3 | 0.054 |
| M3 | IL-1β | 35.3 | 27.3 | 0.8 | 0.63 |
|  | IFN-α2 | 47.7 | 1.0 | 0.02 | <0.0001 |
|  | IFN-ϒ | 53.6 | 68.5 | 1.3 | 0.73 |
|  | TNF-α | 9730.2 | 15.3 | 0.002 | <0.0001 |
|  | MCP-1 | 5.1 | 9858.5 | 1918.8 | <0.0001 |
|  | IL-6 | 2081.7 | 26.6 | 0.01 | <0.0001 |
|  | IL-8 | 17.3 | 1632.1 | 94.3 | <0.0001 |
|  | IL-10 | 5630.9 | 100.1 | 0.02 | <0.0001 |
|  | IL-12p70 | 5.5 | 6.0 | 1.1 | 0.93 |
|  | IL-17A | 65.1 | 4.7 | 0.07 | <0.0001 |
|  | IL-18 | 9209.6 | 10663.3 | 1.2 | 0.85 |
|  | IL-23 | 189.6 | 23.9 | 0.1 | 0.0005 |
|  | IL-33 | 2.9 | 2.0 | 0.7 | 0.47 |
| *p-values were* obtained with Z-test for variance | | | | | |

**Table S6 –**  Variance of the global rate of change estimated for each cytokine at M1 and M3 in R and NR to the impact of disease (CAT). Z-test for variance was performed to assess significant differences between R and NR.

| Time-point | Cytokine | NR (n=12) | R (n=14) | test's statistics | *p-value* |
| --- | --- | --- | --- | --- | --- |
| M1 | IL-1β | 47.3 | 131.1 | 2.8 | 0.099 |
|  | IFN-α2 | 1.0 | 276.3 | 287.0 | <0.0001 |
|  | IFN-ϒ | 3.0 | 168.7 | 56.2 | <0.0001 |
|  | TNF-α | 19.0 | 385.9 | 20.3 | <0.0001 |
|  | MCP-1 | 2692.6 | 14.0 | 0.005 | <0.0001 |
|  | IL-6 | 2401.8 | 232.5 | 0.1 | 0.0002 |
|  | IL-8 | 3883.9 | 22.1 | 0.006 | <0.0001 |
|  | IL-10 | 40.3 | 105.9 | 5.1 | 0.01 |
|  | IL-12p70 | 0.9 | 6.1 | 6.4 | 0.004 |
|  | IL-17A | 0.5 | 22.5 | 45.2 | <0.0001 |
|  | IL-18 | 54805.0 | 1964.3 | 0.04 | <0.0001 |
|  | IL-23 | 2.1 | 337.0 | 164.2 | <0.0001 |
|  | IL-33 | 1.9 | 4.4 | 2.4 | 0.16 |
| M3 | IL-1β | 33.0 | 27.7 | 0.8 | 0.76 |
|  | IFN-α2 | 5.6 | 32.0 | 5.7 | 0.007 |
|  | IFN-ϒ | 31.5 | 90.9 | 2.9 | 0.09 |
|  | TNF-α | 227.0 | 7097.8 | 31.3 | <0.0001 |
|  | MCP-1 | 13133.7 | 3.5 | 0.0003 | <0.0001 |
|  | IL-6 | 90.2 | 1473.0 | 16.3 | <0.0001 |
|  | IL-8 | 2174.3 | 13.2 | 0.006 | <0.0001 |
|  | IL-10 | 160.9 | 4086.2 | 25.4 | <0.0001 |
|  | IL-12p70 | 5.1 | 6.4 | 1.3 | 0.71 |
|  | IL-17A | 9.1 | 45.5 | 5.0 | 0.01 |
|  | IL-18 | 14073.7 | 6639.2 | 0.5 | 0.2 |
|  | IL-23 | 124.2 | 61.6 | 0.5 | 0.23 |
|  | IL-33 | 2.8 | 1.9 | 0.7 | 0.48 |
| *p-values were* obtained with Z-test for variance | | | | | |

# Additional Figures


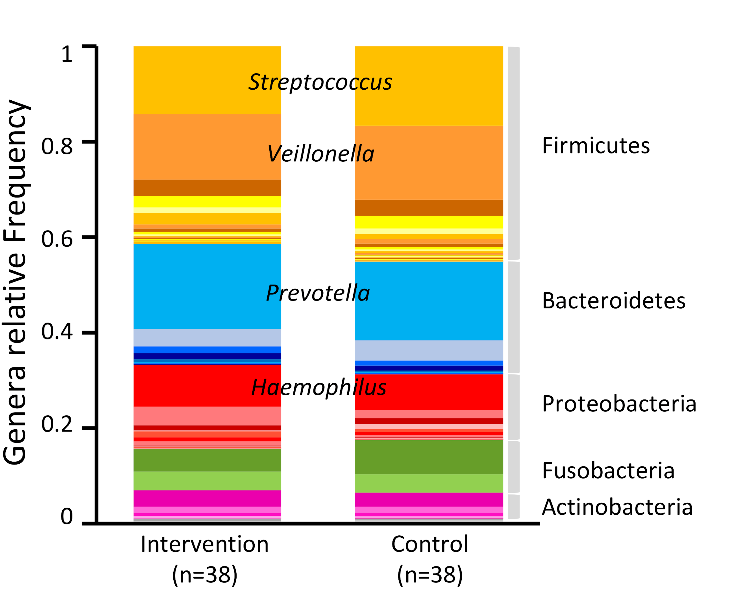


**Figure S1. Mean frequency of phyla and genera of bacteria present in intervention and control groups at baseline (M0).**


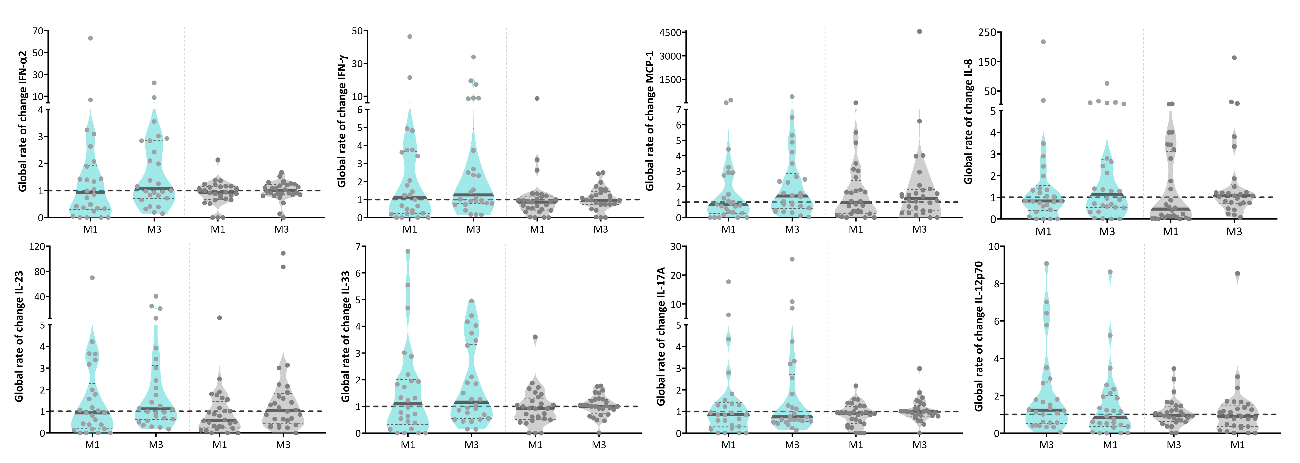


**Figure S2. Violin plots representing the global rate of change of IFN-α2, IFN-ϒ, MCP-1, IL-8, IL-23, IL-33, IL-17A, IL-18 and IL-12p70 in saliva from patients submitted to the 12-week pulmonary rehabilitation programme (blue) and controls (grey)**. Global rate of change represents the ratio between cytokine values measured at baseline and M1 or M3 (*i.e.* M1/M0 and M3/M0). Differences between M1 and the baseline and between M3 and the baseline were assessed by Wilcoxon signed-rank, in each group (intervention (blue) and control (grey)). Differences between groups at M1 and M3 were assessed by Mann-Whitney U-test. According to these criteria no significant shifts were observed for the cytokines represented in the figure.


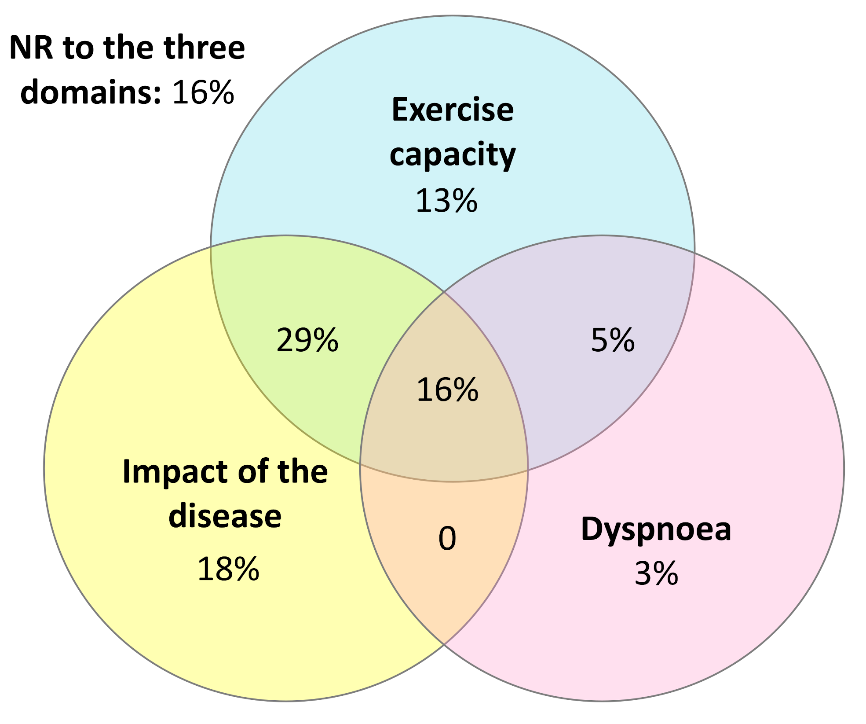


**Figure S3.** Venn diagram showing the percentage of overlap between patients’ (n=38) responsiveness to each domain: dyspnoea, exercise capacity and impact of the disease.


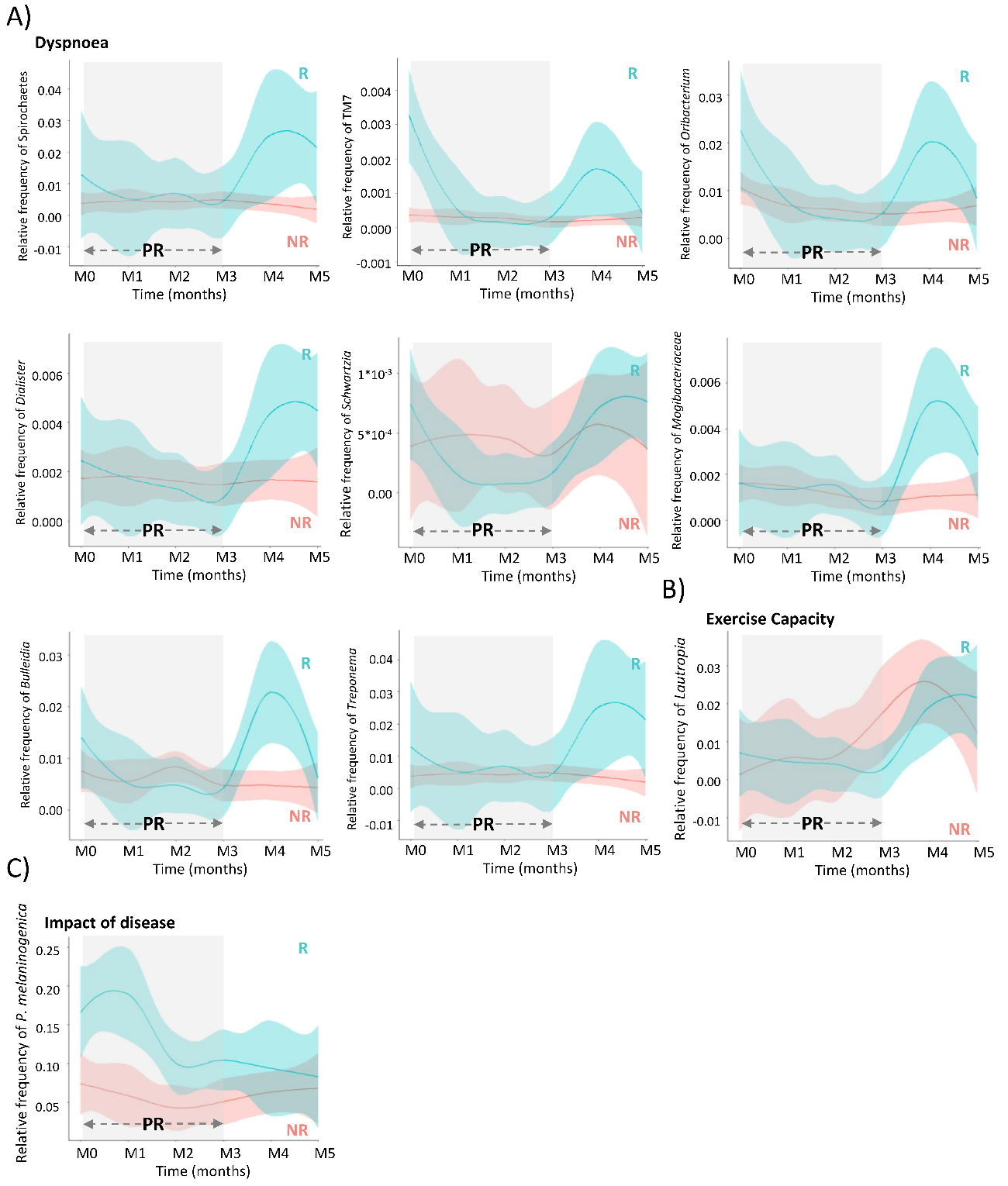


**Figure S4. Relative frequencies over time of taxa presenting significantly different dynamics between responders and non-responders.** Linear mixed-effects models were applied to arcsine square root transformed frequencies of ASVs/OTUs to determine the taxa presenting differential dynamics between R and NR to A) dyspnoea during exercise, B) exercise capacity and C) impact of the disease. Grey rectangles include all the time-points where patients were under pulmonary rehabilitation. Blue and red loess lines were fitted over the data points representing the relative frequency of the correspondent taxon in responders and non-responders (M0=immediately prior to PR; M1, M2, M3= 1,2,3 months after initiating PR; M4, M5=1 and 2 months after terminating PR). The blue and red areas represent the 0.95 confidence intervals.


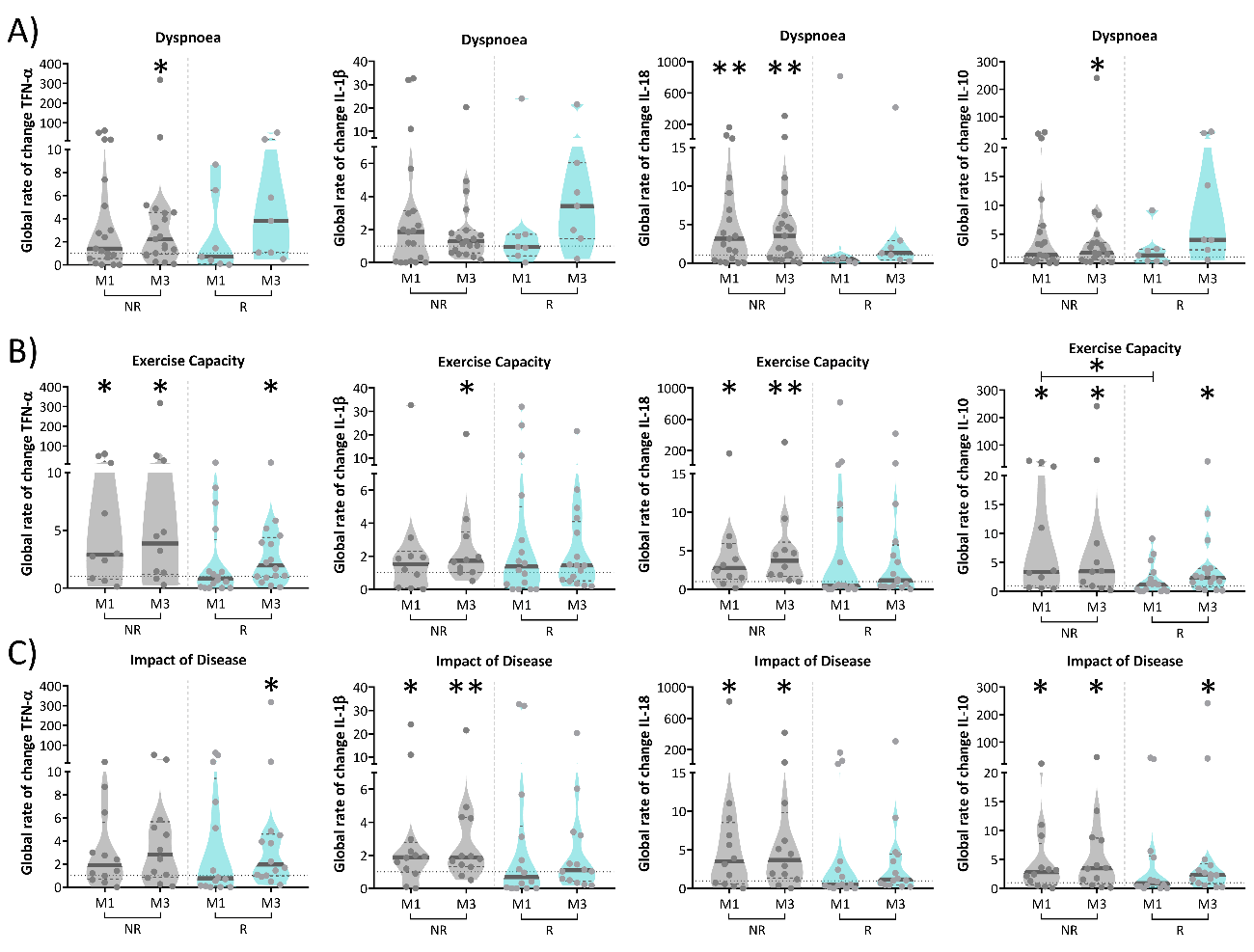


**Figure S5. Responsiveness to pulmonary rehabilitation (PR) was associated with specific alterations in the inflammatory markers.** Global rate of change represents the ratio between cytokine values measured at baseline and M1 or M3 (*i.e*. M1/M0 and M3/M0). Differences between M1 and the baseline and between M3 and the baseline were assessed by Wilcoxon signed-rank, in each group (intervention (blue) and control (grey)). Differences between groups (R and NR) at M1 and M3 were assessed by Mann-Whitney U-test. *p<0.05, **p<0.01, **p<0.001. A) dyspnoea during exercise, B) exercise capacity and C) impact of the disease.


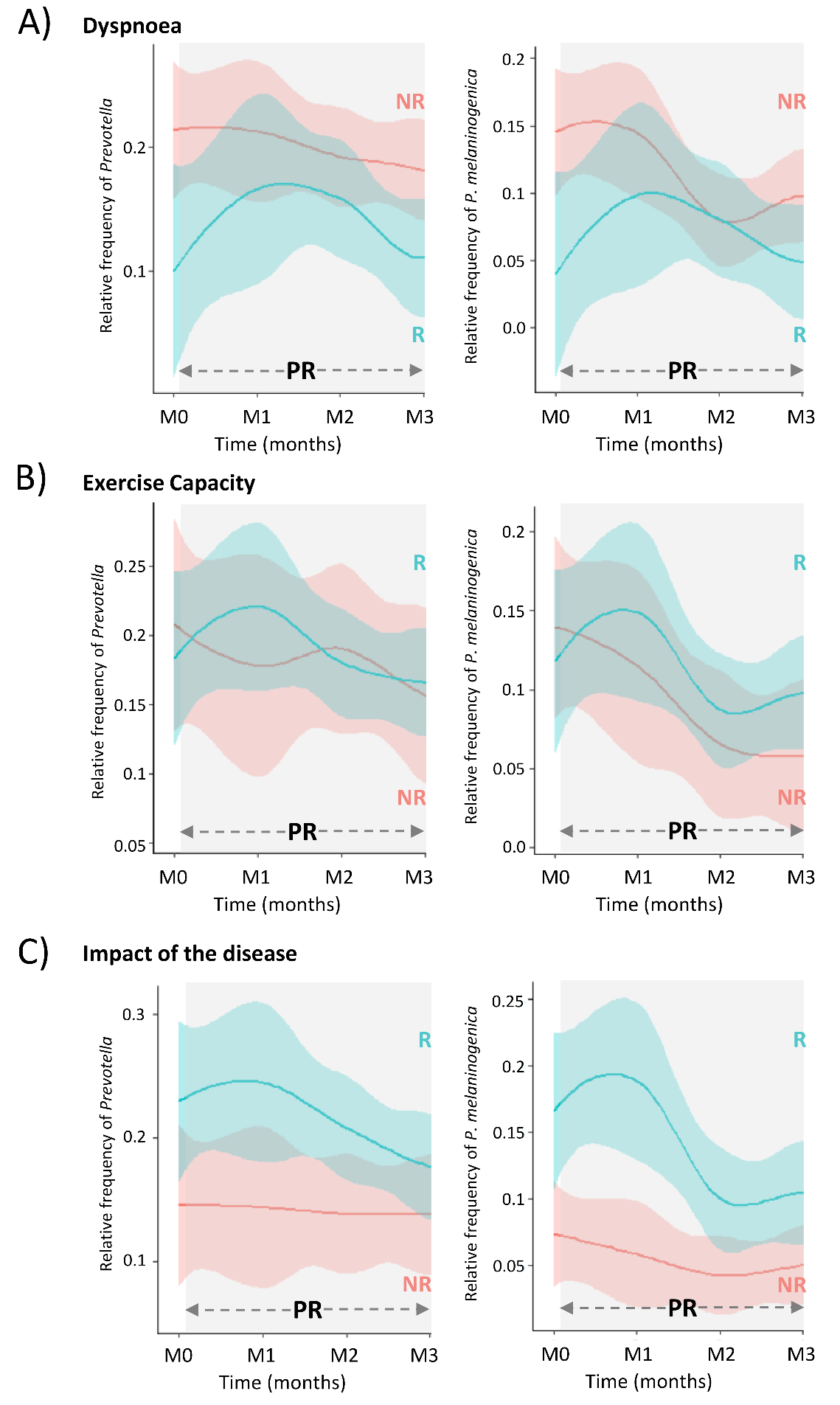


**Figure S6. Frequency of *Prevotella* and *P. melaninogenica* over time in responders and non-responders to A) dyspnoea, B) exercise capacity and C) impact of the disease.** The grey rectangles include all the time-points where patients were under pulmonary rehabilitation. Blue and red loess lines were fitted over the data points representing the relative frequency of the correspondent taxon in responders and non-responders (M0=immediately prior to PR; M1, M2, M3= 1,2,3 months after initiating PR). The blue and red areas represent the 0.95 confidence intervals.

# Code for data analyses

Qiime2 v20.8

The scripts provided were constructed based on the complete dataset, appropriate selection of samples was performed for analyses considering specific time-points and groups of patients with the command: “*Filter samples”*

*Sequencing data import using prepared manifest file:*

qiime tools import **\**

--type 'SampleData[PairedEndSequencesWithQuality]' **\**

--input-path pe-33-manifest.tsv **\**

--output-path paired-end-demux.qza **\**

--input-format PairedEndFastqManifestPhred33

*Joining of the read pairs, and quality assessment:*

qiime vsearch join-pairs \

--p-allowmergestagger \

--i-demultiplexed-seqs paired-end-demux.qza \

--o-joined-sequences demux-joined.qza

qiime demux summarize \

--i-data demux-joined.qza \

--o-visualization demux-joined.qzv

qiime quality-filter q-score \

--i-demux demux-joined.qza \

--o-filtered-sequences demux-joined-filtered.qza \

--o-filter-stats demux-joined-filter-stats.qza

*Denoising with Deblur and statistics visualization:*

qiime deblur denoise-16S \

--i-demultiplexed-seqs demux-joined-filtered.qza \

--p-trim-length 250 \

--p-sample-stats \

--o-representative-sequences rep-seqs.qza \

--o-table table.qza \

--o-stats deblur-stats.qza

qiime feature-table summarize \

--i-table table.qza \

--o-visualization table.qzv \

--m-sample-metadata-file sample-metadata.tsv

qiime feature-table tabulate-seqs \

--i-data rep-seqs.qza \

--o-visualization rep-seqs.qzv

qiime deblur visualize-stats \

--i-deblur-stats deblur-stats.qza \

--o-visualization deblur-stats.qzv

*Filtering data to remove contaminant ASVs found with DECONTAM:*

qiime feature-table filter-features \

--i-table table.qza \

--m-metadata-file features-to-keep.txt \ #features-to-keep.txt is the list of non-contaminant ASVs

--o-filtered-table filt-table.qza

qiime feature-table filter-seqs \

--i-data rep-seqs.qza \

--i-table filt-table.qza \

--o-filtered-data filt-rep-seqs.qza

*Extract eHOMD_15.1 reference sequences according to primers and sequence length, train Naïve-bayes classifier and preform taxonomy assignment:*

#Extract reference sequences

qiime tools import \

--type 'FeatureData[Sequence]' \

--input-path HOMD_16S_rRNA_RefSeq_V15.1.p9.fasta \

--output-path HOMD_otus.qza

qiime tools import \

--type 'FeatureData[Taxonomy]' \

--input-format HeaderlessTSVTaxonomyFormat \

--input-path HOMD_16S_rRNA_RefSeq_V15.1.qiime.taxonomy \

--output-path ref-taxonomy.qza

qiime feature-classifier extract-reads \

--i-sequences HOMD_otus.qza \

--p-f-primer GTGCCAGCMGCCGCGGTAA \

--p-r-primer GGACTACHVGGGTWTCTAAT \

--p-trunc-len 250 \

--p-min-length 100 \

--p-max-length 400 \

--o-reads ref-seqs.qza

#Train the Naïve-bayes classifier

qiime feature-classifier fit-classifier-naive-bayes \

--i-reference-reads ref-seqs.qza \

--i-reference-taxonomy HOMD_taxonomy.qza \

--o-classifier classifier.qza

#Taxonomy assignment

qiime feature-classifier classify-sklearn \

--i-classifier HOMD_classifier.qza \

--i-reads rep-seqs.qza \

--o-classification taxonomy.qza

qiime feature-classifier classify-sklearn \

--i-classifier HOMD_classifier.qza \

--i-reads filt-rep-seqs.qza \

--o-classification filt-taxonomy.qza

*Filter samples (e.g. analyses conducted only at M0):*

qiime feature-table filter-samples \

--i-table filt-table.qza \

--m-metadata-file samples-to-keep_M0.txt \ #samples-to-keep.txt is the list of samples to retain in each specific analysis

--o-filtered-table M0-table.qza

*Sequences alignment and generation of a rooted tree for phylogenetic diversity analyses:*

qiime phylogeny align-to-tree-mafft-fasttree \

--i-sequences filt-rep-seqs.qza \

--o-alignment aligned-rep-seqs.qza \

--o-masked-alignment masked-aligned-rep-seqs.qza \

--o-tree unrooted-tree.qza \

--o-rooted-tree rooted-tree.qza

*Rarefaction and alfa and beta diversity analyses:*

#Alpha-rarefaction

qiime diversity alpha-rarefaction \

--i-table filt-table.qza \

--i-phylogeny rooted-tree.qza \

--p-max-depth 20000 \

--m-metadata-file sample-metadata.tsv \

--o-visualization alpha-rarefaction.qzv

#Diversity analyses

qiime diversity core-metrics-phylogenetic \

--i-phylogeny rooted-tree.qza \

--i-table filt-table.qza \

--p-sampling-depth 8000 \

--m-metadata-file sample-metadata.tsv \

--output-dir core-metrics-results

*Calculation of ASVs relative abundances:*

qiime feature-table relative-frequency \

--i-table filt-table.qza \

--o-relative-frequency-table freq-table.qza

*From PCoAs to Biplots:*

qiime diversity pcoa \

--i-distance-matrix core-metrics-results/weighted_unifrac_distance_matrix.qza \

--p-number-of-dimensions 2 \

--o-pcoa weighted_unifrac_2dpcoa_results.qza

qiime diversity pcoa-biplot \

--i-pcoa weighted_unifrac_2dpcoa_results.qza \

--i-features freq-table.qza \

--o-biplot 2D-weighted_unifrac_biplot_pcoa_results.qza

qiime emperor biplot \

--i-biplot 2D-weighted_unifrac_biplot_pcoa_results.qza \

--m-sample-metadata-file sample-metadata.tsv \

--m-feature-metadata-file filt-taxonomy.qza \

--p-ignore-missing-samples \

--p-number-of-features 3 \

--o-visualization 2D-3-biplot_wU.qzv

*Taxonomy collapse to genera (L6) level and genera relative abundances calculation:*

#taxa collapse

qiime taxa collapse \

--i-table filt-table.qza \

--i-taxonomy filt-taxonomy.qza \

--p-level 6 \

--o-collapsed-table collapsed-filtered-table-l6.qza

#Abundance calculation

qiime feature-table relative-frequency\

--i-table collapsed-filtered-table-l6.qza \

--o-relative-frequency-table collapsed-frequency-filtered-table-l6.qza

*Exporting ASVs’ table, genera table, genera relative abundance table, alpha-diversity vectors and beta-diversity matrixes:*

#Exporting the ASVs’ table, nr of reads and abundance

qiime tools export \

--input-path filt-table.qza \

--output-path abs-table-ASVs

qiime tools export \

--input-path freq-table.qza \

--output-path rel-table-ASVs

#Exporting the genera table, nr of reads and abundance

qiime tools export \

--input-path collapsed-filtered-table-l6.qza \

--output-path abs-table-L6

qiime tools export \

--input-path collapsed-frequency-filtered-table-l6.qza \

--output-path rel-table-L6

#Converting biom 2.0 table into .txt for lefse

biom convert -i feature-table.biom -o otu_table_rel_l6.tsv --to-tsv --header-key taxonomy

#Exporting alpha diversity vectors

qiime tools extract \

--input-path core-metrics-results/shannon_vector.qza \

--output-path extracted-shannon

qiime tools extract \

--input-path core-metrics-results/faith_pd_vector.qza \

--output-path extracted-faith

#Exporting beta-diversity matrixes

qiime tools extract \

--input-path core-metrics-results/weighted_unifrac_distance_matrix.qza \

--output-path extracted-wu

R software

*DECONTAM:*

library(tidyverse)

library(qiime2R)

library(phyloseq)

library(ggplot2)

library(decontam)

?isContaminant

phy<-qza_to_phyloseq(features="E:/PRISMA-final!/PRISMA - SARA/R decontam/table.qza", metadata="E:/PRISMA-final!/PRISMA - SARA/R decontam/sample-metadata.txt", taxonomy="E:/PRISMA-final!/PRISMA - SARA/R decontam/taxonomy.qza", tmp="C:/tmp")

head(sample_data(phy))

df <- as.data.frame(sample_data(phy))

df$LibrarySize <- sample_sums(phy)

df <- df[order(df$LibrarySize),]

df$Index <- seq(nrow(df))

ggplot(data=df, aes(x=Index, y=LibrarySize, color=Sample_or_Control)) + geom_point()

##identify contaminants - prevalence

sample_data(phy)$is.neg <- sample_data(phy)$Sample_or_Control == "Control"

contamdf.prev <- isContaminant(phy, method="prevalence", neg="is.neg")

table((contamdf.prev)$contaminant)

head(which(contamdf.prev$contaminant))

head(which(contamdf.prev))

show(contamdf.prev)

contamdf.prev05 <- isContaminant(phy, method="prevalence", neg="is.neg", batch.combine="minimum", threshold=0.5, normalize=TRUE )

table(contamdf.prev05$contaminant)

table(contamdf.prev05)

show(contamdf.prev05)

# Make phyloseq object of presence-absence in negative controls and true samples

phy.pa <- transform_sample_counts(phy, function(abund) 1*(abund>0))

phy.pa.neg <- prune_samples(sample_data(phy.pa)$Sample_or_Control == "Control", phy.pa)

phy.pa.pos <- prune_samples(sample_data(phy.pa)$Sample_or_Control == "Sample", phy.pa)

# Make data.frame of prevalence in positive and negative samples

df.pa <- data.frame(pa.pos=taxa_sums(phy.pa.pos), pa.neg=taxa_sums(phy.pa.neg),

contaminant=contamdf.prev$contaminant)

ggplot(data=df.pa, aes(x=pa.neg, y=pa.pos, color=contaminant)) + geom_point() +

xlab("Prevalence (Negative Controls)") + ylab("Prevalence (True Samples)")

#writte table

write.table(contamdf.prev05, "E:/PRISMA-final!/PRISMA - SARA/R decontam/contamdf.prev0.5.txt", sep="\t")

*PCoA and PERMANOVA (example M0 RvsNR to mBorg):*

library(qiime2R)

library(phyloseq)

library(ggplot2)

library(vegan)

library(readxl)

library(ggordiplots)

library(ggthemes)

library(ggfortify)

wuM0=read.table(file = 'M0distance-matrix.tsv', sep = '\t', header = TRUE)

wuM0_1<- wuM0[,-1]

rownames(wuM0_1)<-wuM0[,1]

phy<-qza_to_phyloseq(features="D:/PRISMA-final!/PcoA spider/table-M0.qza", metadata="D:/PRISMA-final!/PcoA spider/sample-metadata.tsv", taxonomy="D:/PRISMA-final!/PcoA spider/taxonomy.qza", tree="D:/PRISMA-final!/PcoA spider/rooted-tree.qza", tmp="C:/tmp")

head(sample_data(phy))

phy

sampledf <- data.frame(sample_data(phy))

PcOA_wU <- cmdscale (wuM0_1, eig = TRUE)

gg_ordiplot(PcOA_wU, groups = sampledf$RBMSD, spiders = TRUE, ellipse = FALSE)

phy_group <- get_variable(phy, "RmBorg")

wu_mBorg<- adonis2(wuM0_1 ~ RmBorg, data = sampledf, method = "wunifrac", by=NULL, permutations = 999)

print(wu_mBorg)

*ANCOM 2.1 (Intervention vs Control, longitudinal comparison):*

library(readr)

library(tidyverse)

library(nlme)

library(dplyr)

library(ggplot2)

library(compositions)

# OTU table should be a matrix/data.frame with each feature in rows and sample in columns.

# Metadata should be a matrix/data.frame containing the sample identifier.

# Data Pre-Processing

feature_table_pre_process = function(feature_table, meta_data, sample_var, group_var = NULL,

out_cut = 0.05, zero_cut = 0.90, lib_cut, neg_lb){

feature_table = data.frame(feature_table, check.names = FALSE)

meta_data = data.frame(meta_data, check.names = FALSE)

# Drop unused levels

meta_data[] = lapply(meta_data, function(x) if(is.factor(x)) factor(x) else x)

# Match sample IDs between metadata and feature table

sample_ID = intersect(meta_data[, sample_var], colnames(feature_table))

feature_table = feature_table[, sample_ID]

meta_data = meta_data[match(sample_ID, meta_data[, sample_var]), ]

# 1. Identify outliers within each taxon

if (!is.null(group_var)) {

group = meta_data[, group_var]

z = feature_table + 1 # Add pseudo-count (1)

f = log(z); f[f == 0] = NA; f = colMeans(f, na.rm = T)

f_fit = lm(f ~ group)

e = rep(0, length(f)); e[!is.na(group)] = residuals(f_fit)

y = t(t(z) - e)

outlier_check = function(x){

# Fitting the mixture model using the algorithm of Peddada, S. Das, and JT Gene Hwang (2002)

mu1 = quantile(x, 0.25, na.rm = T); mu2 = quantile(x, 0.75, na.rm = T)

sigma1 = quantile(x, 0.75, na.rm = T) - quantile(x, 0.25, na.rm = T); sigma2 = sigma1

pi = 0.75

n = length(x)

epsilon = 100

tol = 1e-5

score = pi*dnorm(x, mean = mu1, sd = sigma1)/((1 - pi)*dnorm(x, mean = mu2, sd = sigma2))

while (epsilon > tol) {

grp1_ind = (score >= 1)

mu1_new = mean(x[grp1_ind]); mu2_new = mean(x[!grp1_ind])

sigma1_new = sd(x[grp1_ind]); if(is.na(sigma1_new)) sigma1_new = 0

sigma2_new = sd(x[!grp1_ind]); if(is.na(sigma2_new)) sigma2_new = 0

pi_new = sum(grp1_ind)/n

para = c(mu1_new, mu2_new, sigma1_new, sigma2_new, pi_new)

if(any(is.na(para))) break

score = pi_new * dnorm(x, mean = mu1_new, sd = sigma1_new)/

((1-pi_new) * dnorm(x, mean = mu2_new, sd = sigma2_new))

epsilon = sqrt((mu1 - mu1_new)^2 + (mu2 - mu2_new)^2 +

(sigma1 - sigma1_new)^2 + (sigma2 - sigma2_new)^2 + (pi - pi_new)^2)

mu1 = mu1_new; mu2 = mu2_new; sigma1 = sigma1_new; sigma2 = sigma2_new; pi = pi_new

}

if(mu1 + 1.96 * sigma1 < mu2 - 1.96 * sigma2){

if(pi < out_cut){

out_ind = grp1_ind

}else if(pi > 1 - out_cut){

out_ind = (!grp1_ind)

}else{

out_ind = rep(FALSE, n)

}

}else{

out_ind = rep(FALSE, n)

}

return(out_ind)

}

out_ind = matrix(FALSE, nrow = nrow(feature_table), ncol = ncol(feature_table))

out_ind[, !is.na(group)] = t(apply(y, 1, function(i)

unlist(tapply(i, group, function(j) outlier_check(j)))))

feature_table[out_ind] = NA

}

# 2. Discard taxa with zeros >= zero_cut

zero_prop = apply(feature_table, 1, function(x) sum(x == 0, na.rm = T)/length(x[!is.na(x)]))

taxa_del = which(zero_prop >= zero_cut)

if(length(taxa_del) > 0){

feature_table = feature_table[- taxa_del, ]

}

# 3. Discard samples with library size < lib_cut

lib_size = colSums(feature_table, na.rm = T)

if(any(lib_size < lib_cut)){

subj_del = which(lib_size < lib_cut)

feature_table = feature_table[, - subj_del]

meta_data = meta_data[- subj_del, ]

}

# 4. Identify taxa with structure zeros

if (!is.null(group_var)) {

group = factor(meta_data[, group_var])

present_table = as.matrix(feature_table)

present_table[is.na(present_table)] = 0

present_table[present_table != 0] = 1

p_hat = t(apply(present_table, 1, function(x)

unlist(tapply(x, group, function(y) mean(y, na.rm = T)))))

samp_size = t(apply(feature_table, 1, function(x)

unlist(tapply(x, group, function(y) length(y[!is.na(y)])))))

p_hat_lo = p_hat - 1.96 * sqrt(p_hat * (1 - p_hat)/samp_size)

struc_zero = (p_hat == 0) * 1

# Whether we need to classify a taxon into structural zero by its negative lower bound?

if(neg_lb) struc_zero[p_hat_lo <= 0] = 1

# Entries considered to be structural zeros are set to be 0s

struc_ind = struc_zero[, group]

feature_table = feature_table * (1 - struc_ind)

colnames(struc_zero) = paste0("structural_zero (", colnames(struc_zero), ")")

}else{

struc_zero = NULL

}

# 5. Return results

res = list(feature_table = feature_table, meta_data = meta_data, structure_zeros = struc_zero)

return(res)

}

# ANCOM main function

ANCOM = function(feature_table, meta_data, struc_zero = NULL, main_var, p_adj_method = "BH",

alpha = 0.05, adj_formula = NULL, rand_formula = NULL, ...){

# OTU table transformation:

# (1) Discard taxa with structural zeros (if any); (2) Add pseudocount (1) and take logarithm.

if (!is.null(struc_zero)) {

num_struc_zero = apply(struc_zero, 1, sum)

comp_table = feature_table[num_struc_zero == 0, ]

}else{

comp_table = feature_table

}

comp_table = log(as.matrix(comp_table) + 1)

n_taxa = dim(comp_table)[1]

taxa_id = rownames(comp_table)

n_samp = dim(comp_table)[2]

# Determine the type of statistical test and its formula.

if (is.null(rand_formula) & is.null(adj_formula)) {

# Basic model

# Whether the main variable of interest has two levels or more?

if (length(unique(meta_data%>%pull(main_var))) == 2) {

# Two levels: Wilcoxon rank-sum test

tfun = stats::wilcox.test

} else{

# More than two levels: Kruskal-Wallis test

tfun = stats::kruskal.test

}

# Formula

tformula = formula(paste("x ~", main_var, sep = " "))

}else if (is.null(rand_formula) & !is.null(adj_formula)) {

# Model: ANOVA

tfun = stats::aov

# Formula

tformula = formula(paste("x ~", main_var, "+", adj_formula, sep = " "))

}else if (!is.null(rand_formula)) {

# Model: Mixed-effects model

tfun = nlme::lme

# Formula

if (is.null(adj_formula)) {

# Random intercept model

tformula = formula(paste("x ~", main_var))

}else {

# Random coefficients/slope model

tformula = formula(paste("x ~", main_var, "+", adj_formula))

}

}

# Calculate the p-value for each pairwise comparison of taxa.

p_data = matrix(NA, nrow = n_taxa, ncol = n_taxa)

colnames(p_data) = taxa_id

rownames(p_data) = taxa_id

for (i in 1:(n_taxa - 1)) {

# Loop through each taxon.

# For each taxon i, additive log ratio (alr) transform the OTU table using taxon i as the reference.

# e.g. the first alr matrix will be the log abundance data (comp_table) recursively subtracted

# by the log abundance of 1st taxon (1st column) column-wisely, and remove the first i columns since:

# the first (i - 1) columns were calculated by previous iterations, and

# the i^th column contains all zeros.

alr_data = apply(comp_table, 1, function(x) x - comp_table[i, ])

# apply(...) allows crossing the data in a number of ways and avoid explicit use of loop constructs.

# Here, we basically want to iteratively subtract each column of the comp_table by its i^th column.

alr_data = alr_data[, - (1:i), drop = FALSE]

n_lr = dim(alr_data)[2] # number of log-ratios (lr)

alr_data = cbind(alr_data, meta_data) # merge with the metadata

# P-values

if (is.null(rand_formula) & is.null(adj_formula)) {

p_data[-(1:i), i] = apply(alr_data[, 1:n_lr, drop = FALSE], 2, function(x){

suppressWarnings(tfun(tformula,

data = data.frame(x, alr_data,

check.names = FALSE))$p.value)

}

)

}else if (is.null(rand_formula) & !is.null(adj_formula)) {

p_data[-(1:i), i] = apply(alr_data[, 1:n_lr, drop = FALSE], 2, function(x){

fit = tfun(tformula,

data = data.frame(x, alr_data, check.names = FALSE),

na.action = na.omit)

summary(fit)[[1]][main_var, "Pr(>F)"]

}

)

}else if (!is.null(rand_formula)) {

p_data[-(1:i), i] = apply(alr_data[, 1:n_lr, drop = FALSE], 2, function(x){

fit = tfun(fixed = tformula,

data = data.frame(x, alr_data, check.names = FALSE),

random = formula(rand_formula),

na.action = na.omit, ...)

anova(fit)[main_var, "p-value"]

}

)

}

}

# Complete the p-value matrix.

# What we got from above iterations is a lower triangle matrix of p-values.

p_data[upper.tri(p_data)] = t(p_data)[upper.tri(p_data)]

diag(p_data) = 1 # let p-values on diagonal equal to 1

p_data[is.na(p_data)] = 1 # let p-values of NA equal to 1

# Multiple comparisons correction.

q_data = apply(p_data, 2, function(x) p.adjust(x, method = p_adj_method))

# Calculate the W statistic of ANCOM.

# For each taxon, count the number of q-values < alpha.

W = apply(q_data, 2, function(x) sum(x < alpha))

# Organize outputs

out_comp = data.frame(taxa_id, W, row.names = NULL, check.names = FALSE)

# Declare a taxon to be differentially abundant based on the quantile of W statistic.

# We perform (n_taxa - 1) hypothesis testings on each taxon, so the maximum number of rejections is (n_taxa - 1).

out_comp = out_comp%>%mutate(detected_0.9 = ifelse(W > 0.9 * (n_taxa -1), TRUE, FALSE),

detected_0.8 = ifelse(W > 0.8 * (n_taxa -1), TRUE, FALSE),

detected_0.7 = ifelse(W > 0.7 * (n_taxa -1), TRUE, FALSE),

detected_0.6 = ifelse(W > 0.6 * (n_taxa -1), TRUE, FALSE))

# Taxa with structural zeros are automatically declared to be differentially abundant

if (!is.null(struc_zero)){

out = data.frame(taxa_id = rownames(struc_zero), W = Inf, detected_0.9 = TRUE,

detected_0.8 = TRUE, detected_0.7 = TRUE, detected_0.6 = TRUE,

row.names = NULL, check.names = FALSE)

out[match(taxa_id, out$taxa_id), ] = out_comp

}else{

out = out_comp

}

# Draw volcano plot

# Calculate clr

clr_table = apply(feature_table, 2, clr)

# Calculate clr mean difference

eff_size = apply(clr_table, 1, function(y)

lm(y ~ x, data = data.frame(y = y,

x = meta_data %>% pull(main_var),

check.names = FALSE))$coef[-1])

if (is.matrix(eff_size)){

# Data frame for the figure

dat_fig = data.frame(taxa_id = out$taxa_id, t(eff_size), y = out$W, check.names = FALSE) %>%

mutate(zero_ind = factor(ifelse(is.infinite(y), "Yes", "No"), levels = c("Yes", "No"))) %>%

gather(key = group, value = x, rownames(eff_size))

# Replcace "x" to the name of covariate

dat_fig$group = sapply(dat_fig$group, function(x) gsub("x", paste0(main_var, " = "), x))

# Replace Inf by (n_taxa - 1) for structural zeros

dat_fig$y = replace(dat_fig$y, is.infinite(dat_fig$y), n_taxa - 1)

fig = ggplot(data = dat_fig) + aes(x = x, y = y) +

geom_point(aes(color = zero_ind)) +

facet_wrap(~ group) +

labs(x = "CLR mean difference", y = "W statistic") +

scale_color_discrete(name = "Structural zero", drop = FALSE) +

theme_bw() +

theme(plot.title = element_text(hjust = 0.5), legend.position = "top",

strip.background = element_rect(fill = "white"))

fig

} else{

# Data frame for the figure

dat_fig = data.frame(taxa_id = out$taxa_id, x = eff_size, y = out$W) %>%

mutate(zero_ind = factor(ifelse(is.infinite(y), "Yes", "No"), levels = c("Yes", "No")))

# Replace Inf by (n_taxa - 1) for structural zeros

dat_fig$y = replace(dat_fig$y, is.infinite(dat_fig$y), n_taxa - 1)

fig = ggplot(data = dat_fig) + aes(x = x, y = y) +

geom_point(aes(color = zero_ind)) +

labs(x = "CLR mean difference", y = "W statistic") +

scale_color_discrete(name = "Structural zero", drop = FALSE) +

theme_bw() +

theme(plot.title = element_text(hjust = 0.5), legend.position = "top")

fig

}

res = list(out = out, fig = fig)

return(res)

}

**###INTERVENTION vs CONTROL**

#importing data#

otu_data = read_tsv("D:/PRISMA-final!/ANCOM 2.1/INTvsCNT.tsv", skip = 1)

otu_id = otu_data$`feature-id`

otu_data = data.frame(otu_data[, -1], check.names = FALSE)

rownames(otu_data) = otu_id

meta_data = read_tsv("D:/PRISMA-final!/ANCOM 2.1/sample-metadata.tsv")[-1, ]

meta_data = meta_data %>% rename(Sample.ID = `#SampleID`)

#data processing#

feature_table = otu_data; sample_var = "Sample.ID"; group_var = "Group"

out_cut = 0.05; zero_cut = 0.90; lib_cut = 8000; neg_lb = TRUE

prepro = feature_table_pre_process(feature_table, meta_data, sample_var, group_var,

out_cut, zero_cut, lib_cut, neg_lb)

feature_table = prepro$feature_table # Preprocessed feature table

meta_data = prepro$meta_data # Preprocessed metadata

struc_zero = prepro$structure_zeros # Structural zero info

# Step 2: ANCOM

main_var = "Group"; p_adj_method = "fdr"; alpha = 0.05

adj_formula = NULL; rand_formula = "~ 1 | Subject"

control = lmeControl(maxIter = 100, msMaxIter = 100, opt = "optim")

t_start = Sys.time()

res = ANCOM(feature_table, meta_data, struc_zero, main_var, p_adj_method,

alpha, adj_formula, rand_formula, control = control)

t_end = Sys.time()

t_run = t_end - t_start # around 30s

write_csv(res$out, "D:/PRISMA-final!/ANCOM 2.1/results_INT_CTR.csv")

# Step 3: Volcano Plot

# Number of taxa except structural zeros

n_taxa = ifelse(is.null(struc_zero), nrow(feature_table), sum(apply(struc_zero, 1, sum) == 0))

# Cutoff values for declaring differentially abundant taxa

cut_off = c(0.9 * (n_taxa -1), 0.8 * (n_taxa -1), 0.7 * (n_taxa -1), 0.6 * (n_taxa -1))

names(cut_off) = c("detected_0.9", "detected_0.8", "detected_0.7", "detected_0.6")

# Annotation data

dat_ann = data.frame(x = min(res$fig$data$x), y = cut_off["detected_0.7"], label = "W[0.7]")

fig = res$fig +

geom_hline(yintercept = cut_off["detected_0.7"], linetype = "dashed") +

geom_text(data = dat_ann, aes(x = x, y = y, label = label),

size = 4, vjust = -0.5, hjust = 0, color = "orange", parse = TRUE)

fig

*Formatting beta-diversity matrix, global rate of change and Linear mixed effect model between Intervention and Control groups*

library(readr)

library(readr)

beta <- read_delim("weighted-unifrac-filtered-distance-matrix.tsv",

"\t", escape_double = FALSE, trim_ws = TRUE)

View(beta)

beta=beta[,-1]

rownames(beta)=colnames(beta)

#########################################

##### Matrizes com os dados todos #######

#########################################

########

## M0 ##

########

library(tidyverse)

M0=matches(c("M0"), vars=colnames(beta1))

betaM0=beta1[,M0]

rownames(betaM0)=rownames(beta1)

View(betaM0)

M0R=matches(c(

"sample3R451M0",

"sample3R452M0",

"sample3R455M0",

"sample508M0",

"sample509M0",

"sample519M0",

"sample526M0",

"sample527M0",

"sample540M0",

"sample578M0",

"sample583M0",

"sample649M0",

"sample663M0",

"sample665M0",

"sample676M0",

"sample678M0",

"sample694M0",

"sample695M0",

"sample794M0",

"sample795M0",

"sample801M0",

"sample807M0",

"sample809M0",

"sample830M0",

"sample833M0",

"sample837M0",

"sample851M0",

"sample880M0",

"sample884M0",

"sample885M0",

"sample891M0",

"sample897M0",

"sample907M0",

"sample934M0",

"sample935M0",

"sampleP101M0",

"sampleP201M0",

"sampleP204M0"), vars=colnames(betaM0))

betaM0=betaM0[,-M0R]

M0C=matches(c("sample3R451M0",

"sample3R452M0",

"sample3R455M0",

"sample508M0",

"sample509M0",

"sample519M0",

"sample526M0",

"sample527M0",

"sample540M0",

"sample578M0",

"sample583M0",

"sample649M0",

"sample663M0",

"sample665M0",

"sample676M0",

"sample678M0",

"sample694M0",

"sample695M0",

"sample794M0",

"sample795M0",

"sample801M0",

"sample807M0",

"sample809M0",

"sample830M0",

"sample833M0",

"sample837M0",

"sample851M0",

"sample880M0",

"sample884M0",

"sample885M0",

"sample891M0",

"sample897M0",

"sample907M0",

"sample934M0",

"sample935M0",

"sampleP101M0",

"sampleP201M0",

"sampleP204M0"), vars=colnames(beta1))

betaM0=cbind(betaM0,beta1[,M0C])

betaM0=as.data.frame(t(betaM0))

colnames(betaM0)=colnames(beta1)

#Dados originais#

loess.linesM0=NULL

for (i in 1:nrow(betaM0)) {

loess.linesM0=c(loess.linesM0,betaM0[i,])

}

loess.linesM0=as.data.frame(loess.linesM0)

loess.linesM0=t(loess.linesM0)

TPM0=c(rep(3,length(loess.linesM0)))

DEM0=c(rep("I",34*381),rep("C",37*381))

########

## M1 ##

########

M1=matches(c("M1"), vars=colnames(beta1))

betaM1=beta1[,M1]

rownames(betaM1)=rownames(beta1)

View(betaM1)

M1R=matches(c("sample3R451M1",

"sample3R452M1",

"sample3R455M1",

"sample508M1",

"sample509M1",

"sample519M1",

"sample526M1",

"sample527M1",

"sample540M1",

"sample578M1",

"sample583M1",

"sample649M1",

"sample663M1",

"sample665M1",

"sample676M1",

"sample678M1",

"sample694M1",

"sample695M1",

"sample794M1",

"sample795M1",

"sample801M1",

"sample807M1",

"sample809M1",

"sample830M1",

"sample833M1",

"sample837M1",

"sample851M1",

"sample880M1",

"sample884M1",

"sample885M1",

"sample891M1",

"sample897M1",

"sample907M1",

"sample934M1",

"sample935M1",

"sampleP101M1",

"sampleP201M1",

"sampleP204M1"), vars=colnames(betaM1))

betaM1=betaM1[,-M1R]

M1C=matches(c("sample3R451M1",

"sample3R452M1",

"sample3R455M1",

"sample508M1",

"sample509M1",

"sample519M1",

"sample526M1",

"sample527M1",

"sample540M1",

"sample578M1",

"sample583M1",

"sample649M1",

"sample663M1",

"sample665M1",

"sample676M1",

"sample678M1",

"sample694M1",

"sample695M1",

"sample794M1",

"sample795M1",

"sample801M1",

"sample807M1",

"sample809M1",

"sample830M1",

"sample833M1",

"sample837M1",

"sample851M1",

"sample880M1",

"sample884M1",

"sample885M1",

"sample891M1",

"sample897M1",

"sample907M1",

"sample934M1",

"sample935M1",

"sampleP101M1",

"sampleP201M1",

"sampleP204M1"), vars=colnames(beta1))

betaM1=cbind(betaM1,beta1[,M1C])

betaM1=as.data.frame(t(betaM1))

colnames(betaM1)=colnames(beta1)

#Dados originais#

loess.linesM1=NULL

for (i in 1:nrow(betaM1)) {

loess.linesM1=c(loess.linesM1,betaM1[i,])

}

loess.linesM1=as.data.frame(loess.linesM1)

loess.linesM1=t(loess.linesM1)

TPM1=c(rep(4,length(loess.linesM1)))

DEM1=c(rep("I",34*381),rep("C",34*381))

########

## M2 ##

########

M2=matches(c("M2"), vars=colnames(beta1))

betaM2=beta1[,M2]

rownames(betaM2)=rownames(beta1)

View(betaM2)

M2R=matches(c("sample3R451M2",

"sample3R452M2",

"sample3R455M2",

"sample508M2",

"sample509M2",

"sample519M2",

"sample526M2",

"sample527M2",

"sample540M2",

"sample578M2",

"sample583M2",

"sample649M2",

"sample663M2",

"sample665M2",

"sample676M2",

"sample678M2",

"sample694M2",

"sample695M2",

"sample794M2",

"sample795M2",

"sample801M2",

"sample807M2",

"sample809M2",

"sample830M2",

"sample833M2",

"sample837M2",

"sample851M2",

"sample880M2",

"sample884M2",

"sample885M2",

"sample891M2",

"sample897M2",

"sample907M2",

"sample934M2",

"sample935M2",

"sampleP101M2",

"sampleP201M2",

"sampleP204M2"), vars=colnames(betaM2))

betaM2=betaM2[,-M2R]

M2C=matches(c("sample3R451M2",

"sample3R452M2",

"sample3R455M2",

"sample508M2",

"sample509M2",

"sample519M2",

"sample526M2",

"sample527M2",

"sample540M2",

"sample578M2",

"sample583M2",

"sample649M2",

"sample663M2",

"sample665M2",

"sample676M2",

"sample678M2",

"sample694M2",

"sample695M2",

"sample794M2",

"sample795M2",

"sample801M2",

"sample807M2",

"sample809M2",

"sample830M2",

"sample833M2",

"sample837M2",

"sample851M2",

"sample880M2",

"sample884M2",

"sample885M2",

"sample891M2",

"sample897M2",

"sample907M2",

"sample934M2",

"sample935M2",

"sampleP101M2",

"sampleP201M2",

"sampleP204M2"), vars=colnames(beta1))

betaM2=cbind(betaM2,beta1[,M2C])

betaM2=as.data.frame(t(betaM2))

colnames(betaM2)=colnames(beta1)

loess.linesM2=NULL

for (i in 1:nrow(betaM2)) {

loess.linesM2=c(loess.linesM2,betaM2[i,])

}

loess.linesM2=as.data.frame(loess.linesM2)

loess.linesM2=t(loess.linesM2)

TPM2=c(rep(5,length(loess.linesM2)))

DEM2=c(rep("I",35*381),rep("C",34*381))

########

## M3 ##

########

M3=matches(c("M3"), vars=colnames(beta1))

betaM3=beta1[,M3]

rownames(betaM3)=rownames(beta1)

View(betaM3)

M3R=matches(c("sample3R451M3",

"sample3R452M3",

"sample3R455M3",

"sample508M3",

"sample509M3",

"sample519M3",

"sample526M3",

"sample527M3",

"sample540M3",

"sample578M3",

"sample583M3",

"sample649M3",

"sample663M3",

"sample665M3",

"sample676M3",

"sample678M3",

"sample694M3",

"sample695M3",

"sample794M3",

"sample795M3",

"sample801M3",

"sample807M3",

"sample809M3",

"sample830M3",

"sample833M3",

"sample837M3",

"sample851M3",

"sample880M3",

"sample884M3",

"sample885M3",

"sample891M3",

"sample897M3",

"sample907M3",

"sample934M3",

"sample935M3",

"sampleP101M3",

"sampleP201M3",

"sampleP204M3"), vars=colnames(betaM3))

betaM3=betaM3[,-M3R]

M3C=matches(c("sample3R451M3",

"sample3R452M3",

"sample3R455M3",

"sample508M3",

"sample509M3",

"sample519M3",

"sample526M3",

"sample527M3",

"sample540M3",

"sample578M3",

"sample583M3",

"sample649M3",

"sample663M3",

"sample665M3",

"sample676M3",

"sample678M3",

"sample694M3",

"sample695M3",

"sample794M3",

"sample795M3",

"sample801M3",

"sample807M3",

"sample809M3",

"sample830M3",

"sample833M3",

"sample837M3",

"sample851M3",

"sample880M3",

"sample884M3",

"sample885M3",

"sample891M3",

"sample897M3",

"sample907M3",

"sample934M3",

"sample935M3",

"sampleP101M3",

"sampleP201M3",

"sampleP204M3"), vars=colnames(beta1))

betaM3=cbind(betaM3,beta1[,M3C])

betaM3=as.data.frame(t(betaM3))

colnames(betaM3)=colnames(beta1)

loess.linesM3=NULL

for (i in 1:nrow(betaM3)) {

loess.linesM3=c(loess.linesM3,betaM3[i,])

}

loess.linesM3=as.data.frame(loess.linesM3)

loess.linesM3=t(loess.linesM3)

TPM3=c(rep(6,length(loess.linesM3)))

DEM3=c(rep("I",35*381),rep("C",35*381))

########

## M4 ##

########

M4=matches(c("M4"), vars=colnames(beta1))

betaM4=beta1[,M4]

rownames(betaM4)=rownames(beta1)

View(betaM4)

M4C=matches(c("sample3R451M4",

"sample3R452M4",

"sample3R455M4",

"sample508M4",

"sample509M4",

"sample519M4",

"sample526M4",

"sample527M4",

"sample540M4",

"sample578M4",

"sample583M4",

"sample649M4",

"sample663M4",

"sample665M4",

"sample676M4",

"sample678M4",

"sample694M4",

"sample695M4",

"sample794M4",

"sample795M4",

"sample801M4",

"sample807M4",

"sample809M4",

"sample830M4",

"sample833M4",

"sample837M4",

"sample851M4",

"sample880M4",

"sample884M4",

"sample885M4",

"sample891M4",

"sample897M4",

"sample907M4",

"sample934M4",

"sample935M4",

"sampleP101M4",

"sampleP201M4",

"sampleP204M4"), vars=colnames(beta1))

betaM4=cbind(betaM4,beta1[,M4C])

betaM4=as.data.frame(t(betaM4))

colnames(betaM4)=colnames(beta1)

loess.linesM4=NULL

for (i in 1:nrow(betaM4)) {

loess.linesM4=c(loess.linesM4,betaM4[i,])

}

loess.linesM4=as.data.frame(loess.linesM4)

loess.linesM4=t(loess.linesM4)

TPM4=c(rep(7,length(loess.linesM4)))

DEM4=c(rep("I",18*381),rep("C",33*381))

########

## M5 ##

########

M5=matches(c("M5"), vars=colnames(beta1))

betaM5=beta1[,M5]

rownames(betaM5)=rownames(beta1)

View(betaM5)

M5C=matches(c("sample3R451M5",

"sample3R452M5",

"sample3R455M5",

"sample508M5",

"sample509M5",

"sample519M5",

"sample526M5",

"sample527M5",

"sample540M5",

"sample578M5",

"sample583M5",

"sample649M5",

"sample663M5",

"sample665M5",

"sample676M5",

"sample678M5",

"sample694M5",

"sample695M5",

"sample794M5",

"sample795M5",

"sample801M5",

"sample807M5",

"sample809M5",

"sample830M5",

"sample833M5",

"sample837M5",

"sample851M5",

"sample880M5",

"sample884M5",

"sample885M5",

"sample891M5",

"sample897M5",

"sample907M5",

"sample934M5",

"sample935M5",

"sampleP101M5",

"sampleP201M5",

"sampleP204M5"), vars=colnames(beta1))

betaM5=cbind(betaM5,beta1[,M5C])

betaM5=as.data.frame(t(betaM5))

colnames(betaM5)=colnames(beta1)

loess.linesM5=NULL

for (i in 1:nrow(betaM5)) {

loess.linesM5=c(loess.linesM5,betaM5[i,])

}

loess.linesM5=as.data.frame(loess.linesM5)

loess.linesM5=t(loess.linesM5)

TPM5=c(rep(8,length(loess.linesM5)))

DEM5=c(rep("I",16*381),rep("C",36*381))

# Data frame for weighted unifrac in each timepoint (TP)

#### TP 1 ####

BC1I=betaM0[matches("M0", vars = rownames(betaM0)),matches("M0", vars = rownames(beta1))]

BC1I=BC1I[,-M0R]

colnames(BC1I)=rownames(BC1I)

BC1I[upper.tri(BC1I)] <- NA

BC1I[BC1I==0]=NA

BC1WI=NULL

for(i in 1:nrow(BC1I)){

BC1WI=c(BC1WI,BC1I[i,])

}

BC1WI=as.data.frame(BC1WI)

F1=1;Fn=0

for(i in 1:nrow(BC1I)){

for(j in 1:ncol(BC1I)){

Fn=Fn+F1

colnames(BC1WI)[Fn]=paste(rownames(BC1I)[i],"/",colnames(BC1I)[j])

}

}

colnames(BC1WI)=sub("M0","",colnames(BC1WI))

colnames(BC1WI)=sub("M0","",colnames(BC1WI))

BC1C=beta1[M0C,M0C]

BC1C[upper.tri(BC1C)] <- NA

BC1C[BC1C==0]=NA

rownames(BC1C)=colnames(BC1C)

BC1WC=NULL

for(i in 1:nrow(BC1C)){

BC1WC=c(BC1WC,BC1C[i,])

}

BC1WC=as.data.frame(BC1WC)

F1=1;Fn=0

for(i in 1:nrow(BC1C)){

for(j in 1:nrow(BC1C)){

Fn=Fn+F1

colnames(BC1WC)[Fn]=paste(rownames(BC1C)[i],"/",colnames(BC1C)[j])

}

}

colnames(BC1WC)=sub("M0","",colnames(BC1WC))

colnames(BC1WC)=sub("M0","",colnames(BC1WC))

#### TP 2 ####

BC2I=betaM1[matches("M1", vars = rownames(betaM1)),matches("M1", vars = rownames(beta1))]

BC2I=BC2I[,-M1R]

colnames(BC2I)=rownames(BC2I)

BC2I[upper.tri(BC2I)] <- NA

BC2I[BC2I==0]=NA

BC2WI=NULL

for(i in 1:nrow(BC2I)){

BC2WI=c(BC2WI,BC2I[i,])

}

BC2WI=as.data.frame(BC2WI)

F1=1;Fn=0

for(i in 1:nrow(BC2I)){

for(j in 1:nrow(BC2I)){

Fn=Fn+F1

colnames(BC2WI)[Fn]=paste(rownames(BC2I)[i],"/",colnames(BC2I)[j])

}

}

colnames(BC2WI)=sub("M1","",colnames(BC2WI))

colnames(BC2WI)=sub("M1","",colnames(BC2WI))

BC2C=beta1[M1C,M1C]

BC2C[upper.tri(BC2C)] <- NA

BC2C[BC2C==0]=NA

rownames(BC2C)=colnames(BC2C)

BC2WC=NULL

for(i in 1:nrow(BC2C)){

BC2WC=c(BC2WC,BC2C[i,])

}

BC2WC=as.data.frame(BC2WC)

F1=1;Fn=0

for(i in 1:nrow(BC2C)){

for(j in 1:nrow(BC2C)){

Fn=Fn+F1

colnames(BC2WC)[Fn]=paste(rownames(BC2C)[i],"/",colnames(BC2C)[j])

}

}

colnames(BC2WC)=sub("M1","",colnames(BC2WC))

colnames(BC2WC)=sub("M1","",colnames(BC2WC))

#### TP 3 ####

BC3I=betaM2[matches("M2", vars = rownames(betaM2)),matches("M2", vars = rownames(beta1))]

BC3I=BC3I[,-M2R]

BC3I[upper.tri(BC3I)] <- NA

BC3I[BC3I==0]=NA

colnames(BC3I)=rownames(BC3I)

BC3WI=NULL

for(i in 1:nrow(BC3I)){

BC3WI=c(BC3WI,BC3I[i,])

}

BC3WI=as.data.frame(BC3WI)

F1=1;Fn=0

for(i in 1:nrow(BC3I)){

for(j in 1:nrow(BC3I)){

Fn=Fn+F1

colnames(BC3WI)[Fn]=paste(rownames(BC3I)[i],"/",colnames(BC3I)[j])

}

}

colnames(BC3WI)=sub("M2","",colnames(BC3WI))

colnames(BC3WI)=sub("M2","",colnames(BC3WI))

BC3C=beta1[M2C,M2C]

BC3C[upper.tri(BC3C)] <- NA

BC3C[BC3C==0]=NA

rownames(BC3C)=colnames(BC3C)

BC3WC=NULL

for(i in 1:nrow(BC3C)){

BC3WC=c(BC3WC,BC3C[i,])

}

BC3WC=as.data.frame(BC3WC)

F1=1;Fn=0

for(i in 1:nrow(BC3C)){

for(j in 1:nrow(BC3C)){

Fn=Fn+F1

colnames(BC3WC)[Fn]=paste(rownames(BC3C)[i],"/",colnames(BC3C)[j])

}

}

colnames(BC3WC)=sub("M2","",colnames(BC3WC))

colnames(BC3WC)=sub("M2","",colnames(BC3WC))

#### TP 4 ####

BC4I=betaM3[matches("M3", vars = rownames(betaM3)),matches("M3", vars = rownames(beta1))]

BC4I=BC4I[,-M3R]

BC4I[upper.tri(BC4I)] <- NA

BC4I[BC4I==0]=NA

colnames(BC4I)=rownames(BC4I)

BC4WI=NULL

for(i in 1:nrow(BC4I)){

BC4WI=c(BC4WI,BC4I[i,])

}

BC4WI=as.data.frame(BC4WI)

F1=1;Fn=0

for(i in 1:nrow(BC4I)){

for(j in 1:nrow(BC4I)){

Fn=Fn+F1

colnames(BC4WI)[Fn]=paste(rownames(BC4I)[i],"/",colnames(BC4I)[j])

}

}

colnames(BC4WI)=sub("M3","",colnames(BC4WI))

colnames(BC4WI)=sub("M3","",colnames(BC4WI))

BC4C=beta1[M3C,M3C]

BC4C[upper.tri(BC4C)] <- NA

BC4C[BC4C==0]=NA

rownames(BC4C)=colnames(BC4C)

BC4WC=NULL

for(i in 1:nrow(BC4C)){

BC4WC=c(BC4WC,BC4C[i,])

}

BC4WC=as.data.frame(BC4WC)

F1=1;Fn=0

for(i in 1:nrow(BC4C)){

for(j in 1:nrow(BC4C)){

Fn=Fn+F1

colnames(BC4WC)[Fn]=paste(rownames(BC4C)[i],"/",colnames(BC4C)[j])

}

}

colnames(BC4WC)=sub("M3","",colnames(BC4WC))

colnames(BC4WC)=sub("M3","",colnames(BC4WC))

#### TP 5 ####

BC5I=beta1[M4,M4]

BC5I[upper.tri(BC5I)] <- NA

BC5I[BC5I==0]=NA

rownames(BC5I)=colnames(BC5I)

BC5WI=NULL

for(i in 1:nrow(BC5I)){

BC5WI=c(BC5WI,BC5I[i,])

}

BC5WI=as.data.frame(BC5WI)

F1=1;Fn=0

for(i in 1:nrow(BC5I)){

for(j in 1:nrow(BC5I)){

Fn=Fn+F1

colnames(BC5WI)[Fn]=paste(rownames(BC5I)[i],"/",colnames(BC5I)[j])

}

}

colnames(BC5WI)=sub("M4","",colnames(BC5WI))

colnames(BC5WI)=sub("M4","",colnames(BC5WI))

BC5C=beta1[M4C,M4C]

BC5C[upper.tri(BC5C)] <- NA

BC5C[BC5C==0]=NA

rownames(BC5C)=colnames(BC5C)

BC5WC=NULL

for(i in 1:nrow(BC5C)){

BC5WC=c(BC5WC,BC5C[i,])

}

BC5WC=as.data.frame(BC5WC)

F1=1;Fn=0

for(i in 1:nrow(BC5C)){

for(j in 1:nrow(BC5C)){

Fn=Fn+F1

colnames(BC5WC)[Fn]=paste(rownames(BC5C)[i],"/",colnames(BC5C)[j])

}

}

colnames(BC5WC)=sub("M4","",colnames(BC5WC))

colnames(BC5WC)=sub("M4","",colnames(BC5WC))

#### TP 6 ####

BC6I=beta1[M5,M5]

BC6I[upper.tri(BC6I)] <- NA

BC6I[BC6I==0]=NA

rownames(BC6I)=colnames(BC6I)

BC6WI=NULL

for(i in 1:nrow(BC6I)){

BC6WI=c(BC6WI,BC6I[i,])

}

BC6WI=as.data.frame(BC6WI)

F1=1;Fn=0

for(i in 1:nrow(BC6I)){

for(j in 1:nrow(BC6I)){

Fn=Fn+F1

colnames(BC6WI)[Fn]=paste(rownames(BC6I)[i],"/",colnames(BC6I)[j])

}

}

colnames(BC6WI)=sub("M5","",colnames(BC6WI))

colnames(BC6WI)=sub("M5","",colnames(BC6WI))

BC6C=beta1[M5C,M5C]

BC6C[upper.tri(BC6C)] <- NA

BC6C[BC6C==0]=NA

rownames(BC6C)=colnames(BC6C)

BC6WC=NULL

for(i in 1:nrow(BC6C)){

BC6WC=c(BC6WC,BC6C[i,])

}

BC6WC=as.data.frame(BC6WC)

F1=1;Fn=0

for(i in 1:nrow(BC6C)){

for(j in 1:nrow(BC6C)){

Fn=Fn+F1

colnames(BC6WC)[Fn]=paste(rownames(BC6C)[i],"/",colnames(BC6C)[j])

}

}

colnames(BC6WC)=sub("M5","",colnames(BC6WC))

colnames(BC6WC)=sub("M5","",colnames(BC6WC))

#### all TPs #####

BCig=cbind(BC1WI,BC1WC,BC2WI,BC2WC,BC3WI,BC3WC,BC4WI,BC4WC,BC5WI,BC5WC,BC6WI,BC6WC)

BCig=t(BCig)

BCig=as.data.frame(BCig)

BCig$TimePoint=c(rep(3,(ncol(BC1WC)+ncol(BC1WI))),rep(4,(ncol(BC2WC)+ncol(BC2WI))),rep(5,(ncol(BC3WC)+ncol(BC3WI))),rep(6,(ncol(BC4WC)+ncol(BC4WI))),rep(7,(ncol(BC5WC)+ncol(BC5WI))),rep(8,(ncol(BC6WC)+ncol(BC6WI))))

BCig$Grupo=c(rep("PR",ncol(BC1WI)),rep("Control",ncol(BC1WC)),rep("PR",ncol(BC2WI)),rep("Control",ncol(BC2WC)),rep("PR",ncol(BC3WI)),rep("Control",ncol(BC3WC)),rep("PR",ncol(BC4WI)),rep("Control",ncol(BC4WC)),rep("PR",ncol(BC5WI)),rep("Control",ncol(BC5WC)),rep("PR",ncol(BC6WI)),rep("Control",ncol(BC6WC)))

BCig$id=rownames(BCig)

BCig$TP=as.factor(BCig$TimePoint)

BCig$Grupo=as.factor(BCig$Grupo)

colnames(BCig)=c("BC","TimePoint","Grupo","id","TP")

BCig=BCig[complete.cases(BCig),]

library(tools)

BCig$id=file_path_sans_ext(BCig$id)

BCig$Baseline=NULL

for(j in 1:1225){

for (i in 1:nrow(BCig)){

if(BCig$id[i]==BCig$id[j]){

BCig$Baseline[i]=BCig$BC[j]

}

}

}

BCig$Baseline[round(BCig$Baseline,7)==0.2510891]=NA #change for the first value of the matrix

BCig$Baseline[BCig$id=="sample3R493...sample3R506"]=0.2510891 #change for the first value of the matrix

#global rate of change

BCig$Norm_BC=NULL

for(i in 1:nrow(BCig)){

BCig$Norm_BC[i]=BCig$BC[i]-BCig$Baseline[i]

}

BCig$Norm_BC[BCig$TP=="3"]=0

BCig$TimePoint=BCig$TimePoint-2

#experimental design

library(ggthemes)

ggplot(data=BCig, aes(TimePoint,Norm_BC))+

geom_smooth(method="loess", aes(color=Grupo), se=T)+

ylab("Weighted Unifrac (Global Rate of Change)")+

labs(colour="Group")+

theme_classic()+

scale_x_continuous(labels=as.character(BCig$TimePoint),breaks=BCig$TimePoint)

library(lme4)

modelGRC1 <- lmer(Norm_BC ~ Grupo * TP + (1|id), data = BCig)

LME.GCR=Anova(modelGRC1, test.statistic = "F", type = "III")

LME.GCR

### quality assessment ###

AIC(modelGRC1)

library(MuMIn)

r.squaredGLMM(modelGRC1)

res=residuals(modelGRC1)

mean(res)

qqnorm(res, datax = TRUE)

qqline(res, datax = TRUE)

#contrast analyses

library(gmodels)

library(lsmeans)

ref3<-lsmeans(modelGRC1 ,c("Grupo","TP"))

ref3 #check the combination of factors for contrasts

ContrastVec <- list(M3=c(1,-1,0,0,0,0,0,0,0,0,0,0),M4=c(0,0,1,-1,0,0,0,0,0,0,0,0), M5=c(0,0,0,0,1,-1,0,0,0,0,0,0),M6=c(0,0,0,0,0,0,1,-1,0,0,0,0),M7=c(0,0,0,0,0,0,0,0,1,-1,0,0),M8=c(0,0,0,0,0,0,0,0,0,0,1,-1))

summary(contrast(ref3, ContrastVec), adjust = "bonferroni")

#descriptive analysis’ table

M_WIG.G=aggregate(Norm_BC~ Grupo+TimePoint, data = BCig, FUN= "mean" )

SD_WIG.G=aggregate(Norm_BC~ Grupo+TimePoint, data = BCig, FUN= "sd" )

SD_WIG.G$BC=as.numeric(SD_WIG.G$Norm_BC)

Table7=M_WIG.G

Table7$TimePoint=factor(rep(c("M3","M4","M5","M6","M7","M8"), each=2), levels = c("M3","M4","M5","M6","M7","M8"))

Table7$Norm_BC=round(as.numeric(Table7$Norm_BC),2)

View(Table7)

for(i in 1:12){

Table7$Norm_BC[i]=paste(Table7$Norm_BC[i],"\u00B1", round(SD_WIG.G$Norm_BC[i],2))}

colnames(Table7)=c("Grupo","Timepoint","Mean \u00B1 SD")

ref3=as.data.frame(ref3)

Table7$lsmean=NULL

for(i in 1:12){

Table7$lsmean[i]=paste(round(ref3[i,3],2),"\u00B1",round(ref3[i,4],3))

}

Table7=Table7[,c(2,1,3,4)]

library(kableExtra)

kbl(Table7) %>%

kable_classic(full_width=F) %>%

column_spec(1, bold = T) %>%

row_spec(c(0),bold = T)%>%

collapse_rows(columns = 1, valign = "middle")

#library(xlsx)

#write.xlsx(Table7,"Tabelas descritivas.xlsx", sheetName ="Wheighted_U_IG_BL",append=TRUE)

#write.xlsx(Table7,"Tabelas descritivas.xlsx", sheetName ="BC_IG_BL",append=TRUE)

ggline(BCig, x="TP",y="Norm_BC",add = c("mean_se"), color = "Grupo")+ylab("Weighted Unifrac")#Mean plot

ggline(ref3, x="TP",y="lsmean",add = c("mean_se"), color = "Grupo")+ylab("Weighted Unifrac")#Mean plot

#LSMEANS graphs

ref1=as.data.frame(ref1)

colnames(ref1)[1]="Group"

lsmeans1=ref1[,c(1,2,3)]

library(ggplot2)

library(ggpubr)

ggline(ref1, x="TP",y="lsmean", color = "Group")+ylab("lsmeans Weighted Unifrac")

ref3=as.data.frame(ref3)

colnames(ref3)[1]="Group"

lsmeans3=ref3[,c(1,2,3)]

library(ggplot2)

library(ggpubr)

ggline(ref3, x="TP",y="lsmean", color = "Group")+ylab("lsmeans GRC Weighted Unifrac")+labs(colour="Group")

# Effect size

library(effectsize)

library(lme4)

library(car)

library(lmerTest)

model2.1=lmer(BC ~ Grupo * TP + Baseline + (1|id), data = BCig)

summary(model2.1)

summary(model2)

anova(model2.1)#Kenward-Roger (Kenward & Roger, 1997) and Satterthwaite (1941) approach to calculate residuals degrees of freedom

summary(model2)

F_to_eta2(14,5,4356,ci=0.95)

*Linear Mixed effect models, contrast analysis and Loess lines plots (e.g. longitudinal dynamic of Dialister between R and NR to mBorg)*

library(readxl)

longitudinal_data <- read_excel("All_OTUs_longitudinal_compl.xlsx")

#removal of genera that were present in <20% of the samples

OTU_selected=NULL

n=1

for(i in 8:ncol(All_OTUs_longitudinal_compl)){

if(sum(Dados_clean[,i]>0)>=83){

OTU_selected[n]=colnames(All_OTUs_longitudinal_compl)[i]

n = n + 1

}

}

Dados_clean_filtered=Dados_clean[,OTU_selected]

Dados_clean_filtered=as.data.frame(cbind(Dados_clean_filtered,Dados_clean[,c(7:8)],Dados_clean[,1:6],Dados_clean[,204]))

#R e NR

Dados_clean_filtered[,81]=as.factor(Dados_clean_filtered[,81])

Dados_clean_filtered[,82]=as.factor(Dados_clean_filtered[,82])

Dados_clean_filtered[,83]=as.factor(Dados_clean_filtered[,83])

Dados_clean_filtered[,84]=as.factor(Dados_clean_filtered[,84])

Dados_clean_filtered[,85]=as.factor(Dados_clean_filtered[,85])

Dados_clean_filtered[,86]=as.factor(Dados_clean_filtered[,86])

Dados_PR=subset(Dados_clean_filtered,Group=="PR")

#linear mixed effect models between R and NR to mBorg

Resultados_modelos_mBorg=data.frame(OTU=NULL,Statistic=NULL,Pvalue=NULL,Assumptions=NULL)

library(lme4)

library(car)

n=1

for(i in 1:79){

modelo1 <- lmer(Dados_PR_raw[,i] ~ TP*RmBorg + (1|OTU_ID), data = Dados_PR)

LME=Anova(modelo1, test.statistic = "F", type = "III")

if(LME[4,4]<0.05){

Resultados_modelos_mBorg[n,1]=colnames(Dados_PR)[i]

Resultados_modelos_mBorg[n,2]=LME[4,1]

Resultados_modelos_mBorg[n,3]=LME[4,4]

n=n+1

}

}

write.xlsx(Resultados_modelos_mBorg,"OTUs_ASVs_long.xlsx", append = TRUE, sheetName = "mBorg")

#validation of assumptions

modelo_dia_mborg <- lmer(`k__Bacteria;p__Firmicutes;c__Clostridia;o__Clostridiales;f__Veillonellaceae;g__Dialister`~ TP*RmBorg + (1|OTU_ID), data = Dados_PR)

res=residuals(modelo_dia_mborg)

qqnorm(res)

qqline(res)

plot(modelo1)

#contrast analysis

ref1<-lsmeans(modelo_sch_bmsr,c("RmBorg","TP"))

ref1 #check the combination of factors for contrasts

ContrastVec <- list(M1=c(1,-1,0,0,0,0,0,0,0,0,0,0),M2=c(0,0,1,-1,0,0,0,0,0,0,0,0), M3=c(0,0,0,0,1,-1,0,0,0,0,0,0),M4=c(0,0,0,0,0,0,1,-1,0,0,0,0),M5=c(0,0,0,0,0,0,0,0,1,-1,0,0),M6=c(0,0,0,0,0,0,0,0,0,0,1,-1))

summary(contrast(ref1, ContrastVec), adjust = "bonferroni")

#loess lines plot

ggplot(data=Dados_, aes(TP,Dados_PR$`k__Bacteria;p__Firmicutes;c__Clostridia;o__Clostridiales;f__Veillonellaceae;g__Dialister`))+

geom_smooth(method="loess", aes(fill=RmBORG,color= RmBORG, group= RmBORG), alpha=0.3)+

ylab("Abundance")+

labs(colour='Response to mBORG', fill='Response to mBORG')+

labs(colour="Response to mBORG ")+

theme_classic()+

scale_x_continuous(labels=as.character(Dados_clean_filtered$TP),breaks=Dados_clean_filteres$TP)+

ggtitle("Dialister")

*Repeated-measures correlation (e.g. correlations between the log10 of inflammatory cytokines and arcsine square root transformed genera relative abundances in NR to exercise capacity)*

###NR_6MWT####

library(readxl)

longitudinal_data <- read_excel("log10_longitudinal_NR6MWT_l6.xlsx")

longitudinal_data[,5:198]=apply(longitudinal_data[,5:198],2,as.numeric)#selecionar colunas com os dados numéricos

longitudinal_data$`#OTU ID`=gsub("\\M.*","",longitudinal_data$`#OTU ID`)#retirar os M1, M2, M3, M4

longitudinal_data

#removal of genera that were present in <20% of the samples

OTU_selected=NULL

n=1

for(i in 18:ncol(longitudinal_data)){

if(sum(longitudinal_data[,i]>0)>=7)

OTU_selected[n]=colnames(longitudinal_data)[i]

n = n + 1

}

}

longitudinal_data_d=longitudinal_data[,OTU_selected]

#transformation of relative abundances with arcsine square root transformation

longitudinal_data_e=asin(sqrt(longitudinal_data_d))

longitudinal_data_f=as.data.frame(cbind(longitudinal_data_e,longitudinal_data[,1:17

sampleID=NULL

#Sample ID

i=1

n=1

j=1

while(i<=nrow(longitudinal_data_f)){

if(longitudinal_data_f$`#OTU ID`[i]==longitudinal_data_f$`#OTU ID`[j]){

sampleID[i]=n

i=i+1

} else if (longitudinal_data_f$`#OTU ID`[i]!=longitudinal_data_f$`#OTU ID`[j]){

n = n + 1

j=i

}

}

longitudinal_data_f$sampleID=sampleID

Matriz_corr=data.frame(NULL)

Pvalues=data.frame(NULL)

library(rmcorr)

for(i in 68:80){#columns with cytokines

for(n in 1:63){#columns with OTUs/ASVs

r=rmcorr(longitudinal_data_f$sampleID,longitudinal_data_f[,i],longitudinal_data_f[,n],dataset = longitudinal_data_f)

Matriz_corr[i-67,n]=r$r

Pvalues[i-67,n]=r$p

}

}

rownames(Matriz_corr)=colnames(longitudinal_data_f[68:80])#Colunas das citocinas

rownames(Matriz_corr)=gsub("\\ .*","",colnames(longitudinal_data_f[68:80]))#Colunas das citocinas

#Reducing genera taxonomic annotation

for(i in 1:length(colnames(Matriz_corr))){

if(grepl("g__",colnames(longitudinal_data_f)[i])& !grepl("f__;",colnames(longitudinal_data_f)[i])|!grepl("g__",colnames(longitudinal_data_f)[i])){

colnames(Matriz_corr)[i]=paste(gsub(".*;f__", "", colnames(longitudinal_data_f)[i]), gsub("g*.", "", colnames(longitudinal_data_f)[i]))

} else if (grepl("f__;",colnames(longitudinal_data_f)[i])& !grepl("o__;",colnames(longitudinal_data_f)[i])){

colnames(Matriz_corr)[i]=paste(gsub(".*;o__", "", colnames(longitudinal_data_f)[i]), gsub("f*.", "", colnames(longitudinal_data_f)[i]))

} else if (grepl("o__;",colnames(longitudinal_data_f)[i])& !grepl("c__;",colnames(longitudinal_data_f)[i])) {

colnames(Matriz_corr)[i]=paste(gsub(".*;c__", "", colnames(longitudinal_data_f)[i]), gsub("o*.", "", colnames(longitudinal_data_f)[i]))

}

}

colnames(Matriz_corr)

rownames(Pvalues)=colnames(longitudinal_data_f[68:80])#Colunas das citocinas

rownames(Pvalues)=gsub("\\ .*","",colnames(longitudinal_data_f[68:80]))

colnames(Pvalues)=paste(gsub(".*_", "", colnames(longitudinal_data_f)[1:63]) )#Colunas das OTUs ASVs

#export correlation matrix

write.xlsx(Matriz_corr,"Matrix_log10_Citocinas_arcsin_OTUs_NR_6MWTl6.xlsx")

#assessment of assumptions validation

corr_sign=data.frame(Correlation=NULL, P=NULL, Coefficient=NULL, Norm_Validation=NULL)

l=0

for(i in 1:63) {#colunas das ASVs OTUs na matrix de corr

for(n in 1:13){#linhas das citocinas na matrix de corr

if(Pvalues[n,i]<0.05){

l=l+1

corr_sign[l,1]=paste(colnames(Matriz_corr)[i],"VS",rownames(Matriz_corr)[n])

corr_sign[l,2]=Pvalues[n,i]

corr_sign[l,3]=Matriz_corr[n,i]

}

}

}

colnames(corr_sign)=c("Correlation","P-value", "Coefficient")

normal_test=as.data.frame(NULL)

library(rmcorr)

for(i in 68:80){#Colunas das citocinas

for(n in 1:63){#Colunas das ASVs OTUs

r=rmcorr(longitudinal_data_f$sampleID,longitudinal_data_f[,i],longitudinal_data_f[,n],dataset = longitudinal_data_f)

res=residuals(r$model)

shap=shapiro.test(res)[2]

normal_test[i-67,n]=shap#Mudar número para a primeira coluna das citocinas menos 1.

}

}

#normally distributed residuals

l=1

for(i in 1:63) {#columns with OTUs/ASVs

for(n in 1:13){#rows with cytokines

l=l+1

if(normal_test[n,i]<0.05){

corr_sign[l,4]="Not Validated"

} else{

corr_sign[l,4]="Validated"

}

}

}

corr_sign=corr_sign[complete.cases(corr_sign),]

colnames(corr_sign)[4]="Residual Normal Distribution"

library(xlsx)

write.xlsx(corr_sign,"log10_Citocinas_OTUs_NR_6MWTl6.xlsx")

*Correlation network plots (e.g. network based on the significant correlations between the log10 of inflammatory cytokines and arcsine square root transformed genera relative abundances in NR to mBorg)*

library(readxl)

NR_BMSD <- read_excel("Matrix_log10_Citocinas_arcsin_OTUs_NR_mBorg.xlsx")

library(tidyr)

OTUs=c(rep(c('ASV_streptococcus','Lachnoanaerobaculum','Butyrivibrio','Catonella','Lachnospiraceae', 'Lautropia'),each = 13))#Colocar o nome das bactérias que se quer representar

citocinas = c(rep(NR_BMSD$...1,6))

corr=c(NR_mBorg$str_sp,

NR_mBorg$`Lachnospiraceae;g__Lachnoanaerobaculum`,

NR_mBorg$`Lachnospiraceae;g__Butyrivibrio`,

NR_mBorg$`Lachnospiraceae;g__Catonella`,

NR_mBorg$`Lachnospiraceae;__`,

NR_mBorg$`Burkholderiaceae;g__Lautropia`)

data=data.frame(OTU = OTUs, Citokynes = citocinas, Correlation = corr)#all data

data

data_sign = data[c(2,17,18,20,29,41,42,45,47,55,58,60,61,65,68,73),]#select only the correlations validating the assumptions

data_sign <- data_sign[order(data_sign$Correlation),]

data_sign

nodes_sign <- data.frame(

name=c('ASV_streptococcus','Lachnoanaerobaculum','Butyrivibrio','Catonella','Lachnospiraceae', 'Lautropia',

NR_mBorg$...1[c(2,3,4,5,6,7,8,9,13)]), #cytokines

carac=c(rep("Bacteria",6), rep('Citokyne',9))

)

#nodes <- data.frame(

# name=c('Lautropia','Rothia','Gemellaceae',Matrix_log10_Citocinas_arcsin_OTUs_NR_6MWTl6$...1),

#carac=c(rep("Bacteria",3), rep('Citokyne',13))

#)

library(igraph)

# Turn it into igraph object

network <- graph_from_data_frame(d=data_sign, vertices=nodes_sign, directed=F)

V(network)

# Make a palette of colors

vcolrs<-c('goldenrod1','goldenrod1','goldenrod1','goldenrod1','goldenrod1','red','papayawhip', 'papayawhip','papayawhip','papayawhip','papayawhip','papayawhip','papayawhip','papayawhip','papayawhip')

ecolrs <- ifelse( E(network)$Correlation >0, "firebrick3", "deepskyblue3")

deg <- degree(network, mode="all")*1.5

# Make the plot

plot(network, vertex.color=vcolrs, edge.color=ecolrs,

vertex.label.color="black",

vertex.frame.color="#ffffff",

vertex.shape="circle",

vertex.label.family="Arial",

vertex.label.cex=1,

edge.width=as.integer(abs(E(network)$Correlation)*10),

vertex.size=deg*10,

edge.label.cex=0.8)

#open a graphical interface to re-arrange the plot

tkplot(network, vertex.color=vcolrs, edge.color=ecolrs,

vertex.label.color="black",

vertex.frame.color="#ffffff",

vertex.shape="circle",

vertex.label.family="Arial",

vertex.label.cex=1,

edge.width=as.integer(abs(E(network)$Correlation)*10),

vertex.size=deg*10,

edge.label.cex=0.8)

write_graph(network, "NR_BMSD_network.gml", format = "gml")

*Pitman-Morgan paired variance test (e.g. Variance of Il-1β in NR to exercise capacity)*

library(readxl)

var_6mwt <- read_excel("var_6mwt.xlsx")

library(PairedData)

Var.test(var_6mwt$Il1bM1NR, var_6mwt$IL1bM2NR,paired=TRUE)

#p=0.01

Var.test(var_6mwt$IL1bM2NR, var_6mwt$IL1bM4NR,paired=TRUE)

#p=0.007

Var.test(var_6mwt$IL1bM1NR, var_6mwt$IL1bM4NR,paired=TRUE)

#p=0.05

#adjusting for multiple comparisons

a<-c(0.01, 0.007, 0.053)

p.adjust(a, method = "bonferroni", n = 3) #0.030 0.021 0.159

# References

1 Marques A, Jácome C, Rebelo P, *et al.* Improving access to community-based pulmonary rehabilitation: 3R protocol for real-world settings with cost-benefit analysis. *BMC Public Health* 2019;**19**:676. doi:10.1186/s12889-019-7045-1

2 Charlson ME, Pompei P, Ales KL, *et al.* A new method of classifying prognostic comorbidity in longitudinal studies: development and validation. *J Chronic Dis* 1987;**40**:373–83. doi:10.1016/0021-9681(87)90171-8

3 GOLD - Global Strategy for Diagnosis, Management, and prevention of chronic obstructive pulmonary disease 2020 report. 2020. 1–141.

4 Standardization of Spirometry 2019 Update. An Official American Thoracic Society and European Respiratory Society Technical Statement | American Journal of Respiratory and Critical Care Medicine. https://www.atsjournals.org/doi/full/10.1164/rccm.201908-1590ST (accessed 28 Oct 2022).

5 Jones PW, Harding G, Berry P, *et al.* Development and first validation of the COPD Assessment Test. *European Respiratory Journal* 2009;**34**:648–54. doi:10.1183/09031936.00102509

6 Jones PW, Tabberer M, Chen W-H. Creating scenarios of the impact of COPD and their relationship to COPD Assessment Test (CAT^TM^) scores. *BMC Pulm Med* 2011;**11**:42. doi:10.1186/1471-2466-11-42

7 ATS Statement. *Am J Respir Crit Care Med* 2002;**166**:111–7. doi:10.1164/ajrccm.166.1.at1102

8 Holland AE, Spruit MA, Troosters T, *et al.* An official European Respiratory Society/American Thoracic Society technical standard: field walking tests in chronic respiratory disease. *European Respiratory Journal* 2014;**44**:1428–46. doi:10.1183/09031936.00150314

9 Jones PW, Beeh KM, Chapman KR, *et al.* Minimal clinically important differences in pharmacological trials. *Am J Respir Crit Care Med* 2014;**189**:250–5. doi:10.1164/rccm.201310-1863PP

10 Holland AE, Hill CJ, Rasekaba T, *et al.* Updating the minimal important difference for six-minute walk distance in patients with chronic obstructive pulmonary disease. *Arch Phys Med Rehabil* 2010;**91**:221–5. doi:10.1016/j.apmr.2009.10.017

11 Bolyen E, Rideout JR, Dillon MR, *et al.* Reproducible, interactive, scalable and extensible microbiome data science using QIIME 2. *Nat Biotechnol* 2019;**37**:852–7. doi:10.1038/s41587-019-0209-9

12 Baeshen HA. Assessment of salivary pro inflammatory cytokines profile level in patients treated with labial and lingual fixed orthodontic appliances. *PLoS One* 2021;**16**:e0249999. doi:10.1371/journal.pone.0249999

13 Maydych V, Claus M, Watzl C, *et al.* Attention to Emotional Information Is Associated With Cytokine Responses to Psychological Stress. *Frontiers in Neuroscience* 2018;**12**.https://www.frontiersin.org/articles/10.3389/fnins.2018.00687 (accessed 25 Nov 2022).

14 LEGENDplex^TM^. https://www.biolegend.com/en-us/legendplex (accessed 21 Mar 2022).

15 Prism - GraphPad. https://www.graphpad.com/scientific-software/prism/ (accessed 12 Jan 2022).

16 R: The R Project for Statistical Computing. https://www.r-project.org/ (accessed 12 Jan 2022).

17 QIIME 2. https://qiime2.org/ (accessed 12 Jan 2022).

18 vsearch — QIIME 2 2020.8.0 documentation. https://docs.qiime2.org/2020.8/plugins/available/vsearch/ (accessed 30 Dec 2021).

19 deblur — QIIME 2 2020.8.0 documentation. https://docs.qiime2.org/2020.8/plugins/available/deblur/ (accessed 30 Dec 2021).

20 Amir A, McDonald D, Navas-Molina JA, *et al.* Deblur Rapidly Resolves Single-Nucleotide Community Sequence Patterns. *mSystems* 2017;**2**:e00191-16, /msys/2/2/e00191-16.atom. doi:10.1128/mSystems.00191-16

21 Callahan B, Davis NM, Ernst FGM. decontam: Identify Contaminants in Marker-gene and Metagenomics Sequencing Data. 2021. doi:10.18129/B9.bioc.decontam

22 Callahan B. benjjneb/decontam. 2021.https://github.com/benjjneb/decontam (accessed 12 Jan 2022).

23 Phylogenetic inference with q2-phylogeny — QIIME 2 2021.11.0 documentation. https://docs.qiime2.org/2021.11/tutorials/phylogeny/?highlight=phylogeny (accessed 30 Dec 2021).

24 Katoh K, Misawa K, Kuma K, *et al.* MAFFT: a novel method for rapid multiple sequence alignment based on fast Fourier transform. *Nucleic Acids Res* 2002;**30**:3059–66. doi:10.1093/nar/gkf436

25 FastTree 2 – Approximately Maximum-Likelihood Trees for Large Alignments. https://journals.plos.org/plosone/article?id=10.1371/journal.pone.0009490 (accessed 23 Jul 2019).

26 Bokulich NA, Kaehler BD, Rideout JR, *et al.* Optimizing taxonomic classification of marker-gene amplicon sequences with QIIME 2’s q2-feature-classifier plugin. *Microbiome* 2018;**6**:90. doi:10.1186/s40168-018-0470-z

27 QIIME 2 Library. https://library.qiime2.org/plugins/q2-feature-classifier/3/ (accessed 30 Dec 2021).

28 F. Escapa I, Huang Y, Chen T, *et al.* Construction of habitat-specific training sets to achieve species-level assignment in 16S rRNA gene datasets. *Microbiome* 2020;**8**:65. doi:10.1186/s40168-020-00841-w

29 diversity — QIIME 2 2020.8.0 documentation. https://docs.qiime2.org/2020.8/plugins/available/diversity/ (accessed 30 Dec 2021).

30 R: The R Stats Package. https://stat.ethz.ch/R-manual/R-devel/library/stats/html/00Index.html (accessed 12 Jan 2022).

31 Anderson MJ. A new method for non-parametric multivariate analysis of variance. *Austral Ecology* 2001;**26**:32–46. doi:10.1111/j.1442-9993.2001.01070.pp.x

32 adonis function - RDocumentation. https://www.rdocumentation.org/packages/vegan/versions/2.4-2/topics/adonis (accessed 15 Sep 2021).

33 vegan-package: Community Ecology Package: Ordination, Diversity and... in vegan: Community Ecology Package. https://rdrr.io/cran/vegan/man/vegan-package.html (accessed 12 Jan 2022).

34 Mandal S, Van Treuren W, White RA, *et al.* Analysis of composition of microbiomes: a novel method for studying microbial composition. *Microb Ecol Health Dis* 2015;**26**:27663. doi:10.3402/mehd.v26.27663

35 Segata N, Izard J, Waldron L, *et al.* Metagenomic biomarker discovery and explanation. *Genome Biol* 2011;**12**:R60. doi:10.1186/gb-2011-12-6-r60

36 Huttenhower C. Galaxy / Hutlab - Harvard. http://huttenhower.sph.harvard.edu/galaxy/ (accessed 19 Sep 2019).

37 Mandal, Siddhartha. Research - Dr. Siddhartha Mandal: ANCOM 2.0 - updated code. https://sites.google.com/site/siddharthamandal1985/research (accessed 19 Sep 2019).

38 Kaul A, Mandal S, Davidov O, *et al.* Analysis of Microbiome Data in the Presence of Excess Zeros. *Front Microbiol* 2017;**8**:2114. doi:10.3389/fmicb.2017.02114

39 Mandal S, Van Treuren W, White RA, *et al.* Analysis of composition of microbiomes: a novel method for studying microbial composition. *Microbial Ecology in Health & Disease* 2015;**26**. doi:10.3402/mehd.v26.27663

40 Lin FH. User Manual for ANCOM v2.1. 2022.https://github.com/FrederickHuangLin/ANCOM (accessed 31 Jan 2022).

41 Bates D, Mächler M, Bolker B, *et al.* Fitting Linear Mixed-Effects Models Using lme4. *Journal of Statistical Software* 2015;**67**:1–48. doi:10.18637/jss.v067.i01

42 PairedData.pdf. https://cran.r-project.org/web/packages/PairedData/PairedData.pdf (accessed 1 Aug 2022).

43 Bakdash JZ, Marusich LR. Repeated Measures Correlation. *Frontiers in Psychology* 2017;**8**.https://www.frontiersin.org/article/10.3389/fpsyg.2017.00456 (accessed 15 Mar 2022).

44 igraph – Network analysis software. https://igraph.org/ (accessed 22 Jul 2022).

45 Melo-Dias S, Valente C, Andrade L, *et al.* Saliva as a non-invasive specimen for COPD assessment. *Respiratory Research* 2022;**23**:16. doi:10.1186/s12931-022-01935-9

# STROBE Statement—Checklist of items that should be included in reports of cohort studies

|  | Item No | Recommendation |
| --- | --- | --- |
| **Title and abstract** | 1 | (*a*) Indicate the study’s design with a commonly used term in the title or the abstract  **YES** |
|  |  | (*b*) Provide in the abstract an informative and balanced summary of what was done and what was found  **YES** |
| Introduction | | |
| Background/rationale | 2 | Explain the scientific background and rationale for the investigation being reported **YES** |
| Objectives | 3 | State specific objectives, including any prespecified hypotheses **YES** |
| Methods | | |
| Study design | 4 | Present key elements of study design early in the paper **YES** |
| Setting | 5 | Describe the setting, locations, and relevant dates, including periods of recruitment, exposure, follow-up, and data collection **YES** |
| Participants | 6 | (*a*) Give the eligibility criteria, and the sources and methods of selection of participants. Describe methods of follow-up **YES** |
|  |  | (*b*) For matched studies, give matching criteria and number of exposed and unexposed **YES (detailed description in Additional file)** |
| Variables | 7 | Clearly define all outcomes, exposures, predictors, potential confounders, and effect modifiers. Give diagnostic criteria, if applicable **YES (detailed description in Additional file)** |
| Data sources/ measurement | 8* | For each variable of interest, give sources of data and details of methods of assessment (measurement). Describe comparability of assessment methods if there is more than one group **YES (detailed description in Additional file)** |
| Bias | 9 | Describe any efforts to address potential sources of bias **YES (detailed description in Additional file)** |
| Study size | 10 | Explain how the study size was arrived at **n.a.** |
| Quantitative variables | 11 | Explain how quantitative variables were handled in the analyses. If applicable, describe which groupings were chosen and why **YES (detailed description in Additional file)** |
| Statistical methods | 12 | (*a*) Describe all statistical methods, including those used to control for confounding **YES (detailed description in Additional file)** |
|  |  | (*b*) Describe any methods used to examine subgroups and interactions **YES (detailed description in Additional file)** |
|  |  | (*c*) Explain how missing data were addressed **YES (detailed description in Additional file)** |
|  |  | (*d*) If applicable, explain how loss to follow-up was addressed **YES (detailed description in Additional file)** |
|  |  | (*e*) Describe any sensitivity analyses **n.a.** |
| Results | | |
| Participants | 13* | (a) Report numbers of individuals at each stage of study—eg numbers potentially eligible, examined for eligibility, confirmed eligible, included in the study, completing follow-up, and analysed **YES** |
|  |  | (b) Give reasons for non-participation at each stage **YES** |
|  |  | (c) Consider use of a flow diagram **n.a.** |
| Descriptive data | 14* | (a) Give characteristics of study participants (eg demographic, clinical, social) and information on exposures and potential confounders **YES** |
|  |  | (b) Indicate number of participants with missing data for each variable of interest **YES** |
|  |  | (c) Summarise follow-up time (eg, average and total amount) **YES** |
| Outcome data | 15* | Report numbers of outcome events or summary measures over time |
| Main results | 16 | (*a*) Give unadjusted estimates and, if applicable, confounder-adjusted estimates and their precision (eg, 95% confidence interval). Make clear which confounders were adjusted for and why they were included **YES** |
|  |  | (*b*) Report category boundaries when continuous variables were categorized **YES** |
|  |  | (*c*) If relevant, consider translating estimates of relative risk into absolute risk for a meaningful time period **n.a** |
| Other analyses | 17 | Report other analyses done—eg analyses of subgroups and interactions, and sensitivity analyses **YES** |
| Discussion | | |
| Key results | 18 | Summarise key results with reference to study objectives **YES** |
| Limitations | 19 | Discuss limitations of the study, taking into account sources of potential bias or imprecision. Discuss both direction and magnitude of any potential bias **YES** |
| Interpretation | 20 | Give a cautious overall interpretation of results considering objectives, limitations, multiplicity of analyses, results from similar studies, and other relevant evidence **YES** |
| Generalisability | 21 | Discuss the generalisability (external validity) of the study results **YES** |
| Other information | | |
| Funding | 22 | Give the source of funding and the role of the funders for the present study and, if applicable, for the original study on which the present article is based **YES** |

*Give information separately for exposed and unexposed groups.

**Note:** An Explanation and Elaboration article discusses each checklist item and gives methodological background and published examples of transparent reporting. The STROBE checklist is best used in conjunction with this article (freely available on the Web sites of PLoS Medicine at http://www.plosmedicine.org/, Annals of Internal Medicine at http://www.annals.org/, and Epidemiology at http://www.epidem.com/). Information on the STROBE Initiative is available at http://www.strobe-statement.org.
