# Supplementary material for: Responsiveness to pulmonary rehabilitation in COPD is associated with changes in microbiota
Source: Respir Res. 2023 Jan 25;24:29. doi: 10.1186/s12931-023-02339-z (PMC9875510; doi:10.1186/s12931-023-02339-z)
Supplement: Supplementary file 2 — Additional file 2. Summary of DECONTAM results preformed with the prevalence method and a threshold of 0.5 for identification of contaminants in the dataset. [file 12931_2023_2339_MOESM2_ESM.pdf]

Summary of DECONTAM results preformed with the prevalence method and a threshold of 0.5 for identification of contaminants in the dataset.

ASV code	Prevalence in samples	Score	Contaminant
f65ef8a64333fc9aa05887b2aa16f257	30	4.93566934530699E-35	TRUE
8c8598245ea67244fb156b6f31192a41	51	6.55556091875317E-22	TRUE
4bb2a60ebd87faa796298b17bcc4ebef	122	7.12476858009646E-20	TRUE
6c891abaa8f2147383dd332e601800eb	25	5.64462712512244E-19	TRUE
073d0c62d1257a783d7508c138432fc4	22	5.95502824269748E-19	TRUE
609cfe075d3af9506da2e29b0db659fe	20	8.07960447514411E-17	TRUE
20ad5b343a158e617aa2f39adbf005b6	18	1.29146710631416E-12	TRUE
e191f1f77e171985e4ce4e16cfd52089	11	1.13823341316519E-11	TRUE
39032f4b6765815d5012773e6807e4be	14	1.98170460521423E-11	TRUE
d65886bee0f292fbbf5fc64ff1bc0b24	221	1.6692749528622E-10	TRUE
fd657e81a8537d9ff329c0703a0f7f63	10	1.72962979000839E-10	TRUE
20cbb7a8b00c1670d0175f2d742e655b	9	2.52133562311872E-09	TRUE
217791e2b9f75aa3915ad540e35c3612	13	1.0669565420676E-08	TRUE
54be6aa07bc7127f2df3048332e7f0cc	6	7.05006830680515E-08	TRUE
fd2b0b0983f5c7a61e3117545344180c	12	1.19572186337503E-07	TRUE
d23fbef2f31d48eda40876cdbc49933a	194	2.32906586692203E-07	TRUE
574d164310944193d8fc13dc10346e58	45	3.99850620766581E-07	TRUE
1db319dd24afdc2890163fd4cfd776e0	7	0.0000004703898033813	TRUE
8fbb45639f32c85085e06660994f97ae	7	0.0000004703898033813	TRUE
083310805ab3a26fd0b20219868d894b	10	4.78885615151771E-07	TRUE
11f63e800d538ce61113253faf0f0363	10	4.78885615151771E-07	TRUE
e15cb616bbfc94e116f873ba1b50aabc	10	4.78885615151771E-07	TRUE
ed7a396f4b38c1b98b597e923ae53f22	5	1.04469193996803E-06	TRUE
e941a4a1ad9617d44833702de4f0ca82	5	1.04469193996803E-06	TRUE
2bbd06be7ee1e2cef688d5d3b880053f	5	1.04469193996803E-06	TRUE
fca9741d7bcdc137ad78a45a0f50c3b6	30	1.36535722961629E-06	TRUE
5785bb04044f25bd01768aa97d019b2e	8	1.79321917846439E-06	TRUE
8f09bac714c02dc9415770271349dc99	196	1.80482872811538E-06	TRUE
77c55b0f6c379835576843cf02171325	17	4.57816661895207E-06	TRUE
05592ceac1f1697c9020de02157cec66	6	5.98614890787024E-06	TRUE
1187d3f6bbc3c560b79f47cf9ad873bd	49	9.12411188077238E-06	TRUE
c7ef526f7263c4a3683d096b6748a0fb	4	0.0000150558544296955	TRUE
98bc1ec29a4c0f9fbf70d7bfa48a48b6	4	0.0000150558544296955	TRUE
1ea83794b96a00695927de44a90aa26d	4	0.0000150558544296955	TRUE
d8f298eccc87fa0854033cac0ce4baca	4	0.0000150558544296955	TRUE
9ab98702e9cf7c27aa339109ea83e689	4	0.0000150558544296955	TRUE
57ef5006312a9662d87d57c1404c1a87	7	0.0000200061185749385	TRUE
548c3f420c3c6a1eb10f35aed1eeb0c1	7	0.0000200061185749385	TRUE
eefaf42aa3481703873ea5b975cca9bf	15	0.0000200562316647648	TRUE
6ba560a76661744bc65eed04953c1314	5	0.0000721451963282275	TRUE
7f1487c191bfa6ccc9085821aadf4324	65	0.0000897630873541179	TRUE
891019e319f5d8d088ed4f9379fd63ef	208	0.000164834348740025	TRUE
ec9d0f20c8c61eb5032d07937cf1af3c	6	0.000207385864939935	TRUE
0a738d348f5e152a2521b6eb922ef446	6	0.000207385864939935	TRUE
365bc413ed3cf82d8737939b5581c3cc	6	0.000207385864939935	TRUE
791ca5935b55de1be66b9c83d56e4167	3	0.000211212129284659	TRUE
c873733a1c472c123f8063297a02c773	3	0.000211212129284659	TRUE
42a8ef3012c07a08d6fbabaef0273cbb	3	0.000211212129284659	TRUE
a20597397ea624f1d40736bbb973dff1	3	0.000211212129284659	TRUE
ee02b9d7321d96af270ccd201ba17b6b	3	0.000211212129284659	TRUE
048633e2cfb6cd41a06beacf26f04cb6	3	0.000211212129284659	TRUE
6fadc82ce33939380ddb960da7f411c1	3	0.000211212129284659	TRUE
f075744cdedbbbec727c7e1b2037c3e6	3	0.000211212129284659	TRUE
30554407069b2367b77ee68c1982db14	3	0.000211212129284659	TRUE
b2bba50ae5022b13d0cd6641814e0713	3	0.000211212129284659	TRUE
79544aab951bcf5f54f663c1dc3f2912	3	0.000211212129284659	TRUE
b83aaa7e85c9c03fa9485e12be27116b	3	0.000211212129284659	TRUE
e58f4afd38d5d2317e576701eac632a4	3	0.000211212129284659	TRUE
8d3565638a5a89a00aa3233b2b027489	4	0.000814736808279286	TRUE
7bae376e41b6259952179d259f069c1e	4	0.000814736808279286	TRUE
5b84f9aefbea73054bcb5ebbf3904893	4	0.000814736808279286	TRUE
a8a7c72cd7f5319290396ea098c97020	4	0.000814736808279286	TRUE
e6f6be4ccfb278fe562ca7dffb543652	9	0.00153092250680292	TRUE
32cfdd0837255fb3842fc282e432ec52	9	0.00153092250680292	TRUE
e795fed13ead27da7c3bedd15b4a59c1	5	0.00196365213243004	TRUE
a5e3cfb18eb2fd335108bf5b8d60b739	5	0.00196365213243004	TRUE
d62009c3d7662c9c54d23242713f314e	2	0.00288656576689034	TRUE
d5695af3afad7256ad94ba6a38ccd4fb	2	0.00288656576689034	TRUE
e2fcc92ed4f145071525b58f2f750e39	2	0.00288656576689034	TRUE
e55f7ffdc508fc02e0ce54bfb21e6038	2	0.00288656576689034	TRUE
7fa99bd3ee34c6d339e3ff01bd88427e	2	0.00288656576689034	TRUE
0eb8e63cb8f7cb7aa58b403dc29da7de	2	0.00288656576689034	TRUE
464a52deb6e8c4e2b57d980a8d18f04c	2	0.00288656576689034	TRUE
2d34c22edce4b1f2d8a5228ad78f8ea8	2	0.00288656576689034	TRUE
6a9e6cd156ec3e7ca51f4912bfe49614	2	0.00288656576689034	TRUE
da8b2afed60ab7f62166e969e4322f11	6	0.0037851032560835	TRUE
f92c2786afd33f8c9eddd65bcb666f42	18	0.00487193302555845	TRUE
50a3b56e0b7db75ee9daccf7a751ba41	19	0.00630479812169273	TRUE
84716245f53ab2236b84538a9cdd74ed	7	0.0063823686613895	TRUE
ed2e115df9ed4a9cf8de15df330c428e	262	0.00647182724575958	TRUE

cfeb33d30aa1b73ecca815bc65ff35f0	3	0.00844848517138625	TRUE
038cda24080464defef44077e55d614a	3	0.00844848517138625	TRUE
1772eec427522cda596e760ad3ebef61	3	0.00844848517138625	TRUE
a5d1217aa1cbc154c499796f9040d954	14	0.00977619312112795	TRUE
6628b08e13117c52417f5dfee4d9eafd	8	0.00983688431712284	TRUE
b4587918bfe7955deb4fe9932ec07c3d	4	0.0164745460842033	TRUE
2ce83ce8633190af0e5f51af6c6d7da8	4	0.0164745460842033	TRUE
31de871bad103340eaac73b2383506fe	10	0.0195459279544366	TRUE
b57744bd30af9f7a4d538d0aba27596b	11	0.0258714571922254	TRUE
d50706f84ad2be04b147f0a3b36039d3	5	0.0267556257599366	TRUE
1fa7e42cad8192ac5a0f3e2319fb1089	5	0.0267556257599366	TRUE
7b922bd11ea3f2de8a1d01bc0314871f	438	0.0311556538676718	TRUE
bc3d8f06abbe8f3820be7ab8cc2eb279	6	0.0390864277175264	TRUE
0d57a518ec5291ce0ee0643456940a90	38	0.0399684744186126	TRUE
1369aba02dd8b749b4c4c2a799d34207	13	0.0415311220541261	TRUE
107aa7e6b56274803bffd1ccff5b2ee6	14	0.050854414641935	TRUE
d353248c8a7d31bc9f8378a320b77924	14	0.050854414641935	TRUE
8bc54d575f88d09b2ba16b7f571812ae	31	0.0517381435573177	TRUE
6ba7d4f61fd8143e8aa856596c09b9dd	7	0.0532669077242673	TRUE
4908017090823380efa89d3e236c811e	33	0.0655854886123842	TRUE
dc8201773df81f21b280e07a6f320208	8	0.0691034263704471	TRUE
1ea779464fee4aab61b2adbe93db1d13	8	0.0691034263704471	TRUE
7bfaa7f90dff6c876675062e176f2b47	8	0.0691034263704471	TRUE
4dd2f7772c7e05fe793cb8d593a59dd0	8	0.0691034263704471	TRUE
642137de3ea503ff59288deb908c51fa	8	0.0691034263704471	TRUE
aeb091a4d196e216c7ed9c8cc29f36a8	8	0.0691034263704471	TRUE
67cea148133734a4183fb020f6740b4e	2	0.0769230769230769	TRUE
83e8f941ebfaf966ed1b7d81e21e43a6	2	0.0769230769230769	TRUE
403c354ef8601d5aba74b7ee78058e87	2	0.0769230769230769	TRUE
0d3f7ffbeccd21fbb5aa31e55f638069	2	0.0769230769230769	TRUE
508b4814a200ebb7843f78ebe4251e05	2	0.0769230769230769	TRUE
3caf30133a391f2f85e2365d24923375	2	0.0769230769230769	TRUE
d630cbac6a5674e8efe19b8a32751f57	2	0.0769230769230769	TRUE
65dfa12d95100cd914a50aa14ab0c74e	2	0.0769230769230769	TRUE
33c14b40a4d663250dffefdb3ce1f5b4	2	0.0769230769230769	TRUE
8e364327698c6e82d690bae5d042c737	2	0.0769230769230769	TRUE
80feb2fb070545be75f78cbd6b520d5f	2	0.0769230769230769	TRUE
8253a32e37cc65a796a92ee67b50d870	17	0.0845266956187741	TRUE
8fcdcca36d7698798f6aa46b028ac8f7	9	0.0864096619651255	TRUE
b0d5a4ec8e74ac4c6cb4ecb9406183d8	9	0.0864096619651255	TRUE
415033681d70ab871d00f8e199d5485d	9	0.0864096619651255	TRUE
3dd272f8e982d693ab208a7f3d552074	9	0.0864096619651255	TRUE
536bca28d3d5384a4b1880b427e53dcc	10	0.105007314060969	TRUE
5c96ec70d2839b27eb48f2226c96e2ca	10	0.105007314060969	TRUE
4a765a31e0cf23936b0d98af5f0b7b81	3	0.115173403255331	TRUE
9cd7f253e5f398b99b607964de56e802	3	0.115173403255331	TRUE
006447e914f38b29a86b92aab7b8145d	3	0.115173403255331	TRUE
e07ec8b4bb1731226c8bbe954aa9dfd7	3	0.115173403255331	TRUE
495baebe669e1a07a3638212d694929c	3	0.115173403255331	TRUE
b32b830756e43af68ac23a4f88d98118	3	0.115173403255331	TRUE
ba14674a604faae7a83e0ab9d7151dcd	49	0.115653659271681	TRUE
df6485f0c59f870ffa34796e56205884	4	0.153031417037874	TRUE
6836e6f80ca06e620471efbe1f47dffe	4	0.153031417037874	TRUE
2700db6dd1459de93b072b5f8f90c146	4	0.153031417037874	TRUE
8089fc5cea667447a21f0c665b788e9c	4	0.153031417037874	TRUE
872d11b3c9c9e72c129296b6ab82e76a	4	0.153031417037874	TRUE
776b3cb9d774b6e0c45c45ccc1764f1b	4	0.153031417037874	TRUE
0d87b112bc336b97248bf4757a668689	4	0.153031417037874	TRUE
1bfddc56f0549c7953359140bb8c64b1	13	0.166895948842549	TRUE
d372b6450fe1d69ae6cb673dfafdce6d	14	0.18905177036381	TRUE
490a5a0203d6a3ce512fe7566a89299c	5	0.190342995483322	TRUE
546a3202fd799a7240d2592ac0444502	5	0.190342995483322	TRUE
1b18df940027dc40b173be56b5b2c39a	5	0.190342995483322	TRUE
8cd7646d4eee2b702e680c9a36aa93ee	5	0.190342995483322	TRUE
e3064dc22ea1c1084cae7e065ec8305c	35	0.19916119737622	TRUE
074478577353df0840cde9827351d2e9	15	0.211740989485119	TRUE
a022ba393ceb3a7e109b171c5e901b31	15	0.211740989485119	TRUE
1189d4ef3af60ba943cc73161a4097a9	15	0.211740989485119	TRUE
7e06f6f4c9c89933a432f36767cf8f5c	6	0.226978141364239	TRUE
16357c104069b5c62302e913480dce46	6	0.226978141364239	TRUE
127b623fd88482c6bf125beda04dba52	6	0.226978141364239	TRUE
8b2b5fea59a9f7c4b75b285985f8bc45	6	0.226978141364239	TRUE
9cd232a91d762e72b8695ef807e4b595	6	0.226978141364239	TRUE
56d2f08c47e1fe5e8b11c758e3df45c4	6	0.226978141364239	TRUE
7cbcb853e8b0fc4e4ae9ad8a8893a7df	6	0.226978141364239	TRUE
42ae34a9e758d6ba2c23b9e32be90e80	6	0.226978141364239	TRUE
d0fd1a04107c82982b6555553b7bcf41	6	0.226978141364239	TRUE
b558365592f7a8d645f69873f6cda354	6	0.226978141364239	TRUE
e78a758dd0cf4ecdeec205371b241817	6	0.226978141364239	TRUE
262dc7bf78e937b0c7065aef82e18d08	6	0.226978141364239	TRUE
53e1b42a26f12c7056f1acb645630a26	6	0.226978141364239	TRUE
74d8b8501ebe056c7685c5f09a15ad1d	6	0.226978141364239	TRUE

6e5eea14c8f71331e69a36f9df2e47cf	6	0.226978141364239	TRUE
e52a5d6f0e2560fe327b2f502dd5a024	6	0.226978141364239	TRUE
8aa02408859b45f0f98146372cadf479	6	0.226978141364239	TRUE
2cb9077e3906b6434cc06d7bf5c82e57	175	0.235878918358532	TRUE
6145f51f090994c4a43f7cea73f90091	17	0.2582325274065	TRUE
e269d8c81692a109f5be5e0d96cc45f4	17	0.2582325274065	TRUE
d5cd3fcafc36006692274229875ace16	7	0.262828389827809	TRUE
168860bd1024ae270960f35b81de4cc3	7	0.262828389827809	TRUE
029af632b2cc681c2baac604bc1d0f87	7	0.262828389827809	TRUE
7840fd72d5760bfb9d7c7d6e05e15b1d	7	0.262828389827809	TRUE
ca3d5ad542f41c1b2dca122a276dd68e	7	0.262828389827809	TRUE
ec5b70612ab541270f99ac9ccb03c57a	7	0.262828389827809	TRUE
5ec80473635e19defc7cb89911f32028	7	0.262828389827809	TRUE
bfe54af4c9180d37a0d76f6dafe79a5a	7	0.262828389827809	TRUE
527f393f41091fdc92723873b3f241f4	7	0.262828389827809	TRUE
4dfc1ccde14bb2c6f22c28071d19002d	7	0.262828389827809	TRUE
efb8602f3d0765aaf09b975799823da9	7	0.262828389827809	TRUE
5406ba4d58928df06e4f23d5a090f3d3	7	0.262828389827809	TRUE
65a39bc08ea93ac99b021f4dc1850237	7	0.262828389827809	TRUE
bd2ebc70501f7d867c204f94c4e483da	391	0.277100555069908	TRUE
340d2fc0f9822e35f88b16537341315c	8	0.297804452748937	TRUE
8c416f8840299049567cb009734c133d	8	0.297804452748937	TRUE
668fdb718997fc1589c7817655d4bb5f	8	0.297804452748937	TRUE
53d5533a6429e4aa7d6998450efe4a0b	8	0.297804452748937	TRUE
1ebadfa667c9a909e90f32ae471aee3b	8	0.297804452748937	TRUE
4fbbe364adcde3a84f51a161ebfa54b7	8	0.297804452748937	TRUE
7124e1c5c05a47eade99eb30e14ca10d	8	0.297804452748937	TRUE
b2487c386ddc1a950ba23034e0d108ba	53	0.298909268067355	TRUE
a71d241034f123d92d24606ffa097445	19	0.305485539889719	TRUE
d8ecd05fc246253fa7171e885a429464	20	0.329158787407426	TRUE
986d1160fa2c6d6e1b39adda581cbd09	9	0.331834080910981	TRUE
b474087ebf36a250a0d6e688aacc9b18	9	0.331834080910981	TRUE
de0880c59770b57d7bce7a58c29a63a4	9	0.331834080910981	TRUE
37bea876591b5db836c3cf500aeb2c4b	9	0.331834080910981	TRUE
743877d0dd17489090dfcc618262fe52	9	0.331834080910981	TRUE
2967fc5e7264a9da63556dc7aa011846	10	0.364860125823334	TRUE
29edd9d99bdeb69d7f7425b1fd0ba57e	10	0.364860125823334	TRUE
2e1a0c95b8b627148e4efc1a2e2124d3	10	0.364860125823334	TRUE
0bb1c8431343726b5826ba41bceb2deb	10	0.364860125823334	TRUE
69c82a0446a784e0f628baba67a71a38	76	0.379808917624895	TRUE
73bf8d1a5983e34a0cb84e3cae127815	433	0.382965201505468	TRUE
ef5b1d86471ba81aeaf0675cb5f133b6	11	0.396838784394777	TRUE
22c8e6e55e7d3f64fc2fdf7b5cc181a2	11	0.396838784394777	TRUE
6b0ff3b95f5a0170f8cf05f2d648be69	11	0.396838784394777	TRUE
0047d48fbaf4753f25019037ff802349	11	0.396838784394777	TRUE
c48ebcb64a879ac3131a8157726e0ce1	11	0.396838784394777	TRUE
5d6ee23084c6b9c96deb9a83295abc8a	78	0.408454653042303	TRUE
e370166a24f9ddb12cc92317e714f124	12	0.42773801098847	TRUE
44beba34c62eeb03966645efddedf0d9	12	0.42773801098847	TRUE
a32083174848f3d175ed977dc8eed9e9	12	0.42773801098847	TRUE
f5d0c756653e860f49eb2ef329840519	12	0.42773801098847	TRUE
c6357b5b5bb8c067c51faad5180b4e99	12	0.42773801098847	TRUE
40e44aaab0f468d14cc6f40ebef19cb0	12	0.42773801098847	TRUE
b1e1799fee04670207c1b71957c46d15	12	0.42773801098847	TRUE
03d52421a28d9b73c5d1716eb520718b	12	0.42773801098847	TRUE
22357830cad050ff79bf4e8f8488beb6	12	0.42773801098847	TRUE
147e062a32552d807a7a72da18b5c56b	12	0.42773801098847	TRUE
508524d40fa1145d6023b5a43a013f4c	12	0.42773801098847	TRUE
70e81e8f3ec3fdc237118834836a5ff0	12	0.42773801098847	TRUE
ab4429b988a4f6fc70f599b57a966c47	12	0.42773801098847	TRUE
677929eac52be081cdae08a0b9c70eb2	37	0.435706683699951	TRUE
55e6f47ff8b1dc2064c0182cb4f831a3	13	0.457536082595734	TRUE
b68071682d3bd6beb1a552c2500791cc	13	0.457536082595734	TRUE
48d9602985017b3a08622909440fccc9	13	0.457536082595734	TRUE
a36165e53c5432cedc59fba58c989704	13	0.457536082595734	TRUE
e3acd1847381805fd9cf7f67f238c142	13	0.457536082595734	TRUE
7d17d197d7978be0b6684209ce257e20	51	0.460922442542212	TRUE
1f4f4a82b05eeccd5361047345201e66	26	0.467192815197644	TRUE
f81aae39d658f14666bbd39fe410294e	26	0.467192815197644	TRUE
165cd1982e320b427a4cfa307f787b38	14	0.486220303988033	TRUE
f90e88c52df1bc147bfe9705270cb88f	14	0.486220303988033	TRUE
6f517a2da49d22439a74d5297bdb654f	14	0.486220303988033	TRUE
f342485eb28655371ab95467e436666b	27	0.489003842984167	TRUE
436e7f204759c2f369afb864438d1262	14	0.5	FALSE
c205d0ee4db8c2c892ebe949b0a069da	67	0.5	FALSE
38a149a8cc59529f0b4ce29d6a838176	162	0.500000000000001	FALSE
7fae0a4914d95fd2426aa8a2ea9ef487	236	0.500000000000001	FALSE
0cbf8d071fa1320c4a225b51196da078	85	0.506863732942227	FALSE
addf717222210dc7bcd173e43b0aae60	28	0.510360069573429	FALSE
9ceae8fed3a46d7b83b2131a74cedb29	15	0.513785840746033	FALSE
345b2e2f68d0022bf201b41dbd25743a	15	0.513785840746033	FALSE
7ed82ab99601f839d82875a9fed6caec	16	0.540234669026948	FALSE

3081ffab8e3fff703f35599fbe5571c4	16	0.540234669026948	FALSE
ce0fe9dd4a105852b029c2ab1d997024	56	0.541776569825547	FALSE
c112b3dc575ef3b721464d0b49daa905	43	0.545307707097171	FALSE
0dc8e0e89e89cf4f0c71808666f6c6e7	30	0.551591656513902	FALSE
f03dffd2e63efb9a0dbc61f7ffa6fe0a	76	0.564343462092671	FALSE
44c386760dcb28e8af3b9847120c5d76	17	0.565574631822175	FALSE
1b158b8b2922d4fcad5d9cea607cbb7d	299	0.568562781856852	FALSE
ee32615285e3f0dfe911611dbd723a22	2	0.574036511156187	FALSE
bd8963179f96afda80d644b420b327ef	2	0.574036511156187	FALSE
495cda065a2ac9820db531b20e15aa1a	2	0.574036511156187	FALSE
59ebca98884af4ca79e99f56319810e6	2	0.574036511156187	FALSE
651b456fad22829867a61aff2a233caa	2	0.574036511156187	FALSE
40e9bb5ebb3c3102bb2bb4efe90a3a91	2	0.574036511156187	FALSE
2af0887c08fc8372e9894be7dd331020	2	0.574036511156187	FALSE
fc49e88a96a3582603047d56f9834715	2	0.574036511156187	FALSE
b76be88631fc1577ea6251a4ea6ad5be	2	0.574036511156187	FALSE
ece6618fc76d78380d6aa565960048f5	2	0.574036511156187	FALSE
746cc895942c2f16e6f9e05d9a912f00	2	0.574036511156187	FALSE
d3326adfc4e7e9d8ea929cc2b5ced9f9	2	0.574036511156187	FALSE
ea60a6f4d4e904f8f2d74612da65f602	2	0.574036511156187	FALSE
569333c671b24e2363d54e4671bb4cfd	2	0.574036511156187	FALSE
eadced2b44e8b968c557dfd8b5ac02dc	2	0.574036511156187	FALSE
329dade18db9c95dd219f5689ee777a3	2	0.574036511156187	FALSE
2613b77df8ce69292ad18bac50100eb6	2	0.574036511156187	FALSE
45c9cc46f257ba8de190eedb3dda5a36	2	0.574036511156187	FALSE
5c596a361996ea8d199802e4e34a6dee	2	0.574036511156187	FALSE
5bbe0a3078b4537152c396ef08fe6e78	2	0.574036511156187	FALSE
fbe5f855d23b47ec4e757e0fa3818ce5	2	0.574036511156187	FALSE
acd5713a39bbcb804b886f98694671b7	2	0.574036511156187	FALSE
3a247cedad2882a4fd1104f0ab9a65ca	2	0.574036511156187	FALSE
0bd637004516b97302a1f56e4f4d337a	2	0.574036511156187	FALSE
146bc18aaafdf9fc303f5cc2870c8e52	2	0.574036511156187	FALSE
bd4660e763464929bf79b4eeea699730	2	0.574036511156187	FALSE
c54410317ca2d9aa7aa88daa1147b46e	2	0.574036511156187	FALSE
dcc015b723865292b60a75224f821f71	2	0.574036511156187	FALSE
0360a55d85a1a3707023ff92dc73f805	2	0.574036511156187	FALSE
49b154d4c99e12186f73403e5ee94e83	2	0.574036511156187	FALSE
8c186c80797f8c256dc61aefe23d3411	2	0.574036511156187	FALSE
bd6a338d9c0a72f4b1a8410f16b85232	2	0.574036511156187	FALSE
a2068af49962d42e50ab5c8a0b744b71	2	0.574036511156187	FALSE
2f08fd7c00722cdaeb01512616c093a5	2	0.574036511156187	FALSE
b76404fb4d67434e693101ad1595767c	2	0.574036511156187	FALSE
e20f803f58f5ce46feabb0516cd02431	2	0.574036511156187	FALSE
acffff1db4fa874c7ff9e2825dd29a4b	2	0.574036511156187	FALSE
39e98dd4e807cd97e92b9f2169cafa9f	2	0.574036511156187	FALSE
791618ea7f777b9c2e7f908eebc1f2a1	2	0.574036511156187	FALSE
919de377b64f65dd94f18d0fca292bac	2	0.574036511156187	FALSE
dbf82288883244531ea0b57e2ed04a2a	2	0.574036511156187	FALSE
6b4fab5c304a37dd85146749d0028d87	2	0.574036511156187	FALSE
66c196e506f48ac22b611efac4a498d7	2	0.574036511156187	FALSE
981823f8c26086d499b6917a34f5961c	2	0.574036511156187	FALSE
20cfc8421a8c7570128653af9f9e5c63	2	0.574036511156187	FALSE
9d73ad67d5a5acbf511c78c859272821	2	0.574036511156187	FALSE
e5abe350b1c88e1126e9074c6e93ef15	2	0.574036511156187	FALSE
b3d6fcd212a9fd5fde39a3d79fee2faa	2	0.574036511156187	FALSE
1730d6157fef2851906040de8a99d96b	2	0.574036511156187	FALSE
dc40f19361b9081547655a601487f5ae	2	0.574036511156187	FALSE
3ed213ab80d34478df6c82075098049d	2	0.574036511156187	FALSE
ccea7446425ef2321bda5cb26650e0df	2	0.574036511156187	FALSE
97e91a44d9196a4f419d23186ef530bc	2	0.574036511156187	FALSE
480018a3dae196593b9153ec81e6d9dd	2	0.574036511156187	FALSE
e13ef43b8d2b3a52b013425e712bfb02	2	0.574036511156187	FALSE
a70bdbc2f74cab32c202b027329fef19	2	0.574036511156187	FALSE
bdb6ca07b4442f773d274bb49ef6a218	2	0.574036511156187	FALSE
000e38f477632fa6299901badf2e0f06	2	0.574036511156187	FALSE
70d3445bf16d2a93bcc75728b6d56c4b	2	0.574036511156187	FALSE
0c72ae13d05b1abb477b63055be82120	2	0.574036511156187	FALSE
c24ecd06ba3dbda7902bf9911bf372b9	2	0.574036511156187	FALSE
19981ee4c04815f6a74c02c8278b4dac	2	0.574036511156187	FALSE
6bf39213fe84babc512eabc37315923b	2	0.574036511156187	FALSE
473bc5c589d3abf37972dd9c7cde20bb	2	0.574036511156187	FALSE
34216a28c46c028e1026aae8d27271d6	2	0.574036511156187	FALSE
6f0deb7981650ae3e9ee2c309a4565e4	2	0.574036511156187	FALSE
963582325831c0cd60c85bb2a828935f	2	0.574036511156187	FALSE
dd14a2ab8cafbc7651bdb1cce5773535	2	0.574036511156187	FALSE
22d32c27c94d47482fd25cb0e8c65e38	2	0.574036511156187	FALSE
acc70fa94c2ae515818445e37268f59f	2	0.574036511156187	FALSE
35d552e9aa4e7126cc2489e0b0aab0db	2	0.574036511156187	FALSE
495174360f9b450d25be0fcdb035630b	2	0.574036511156187	FALSE
1a528eea8d2f33474bd7f647cc1ba694	2	0.574036511156187	FALSE
5c6ac5a27dd6c66c995e76f28ff3fea4	2	0.574036511156187	FALSE
9dabf344a87b6c26d226351cc10a2a5d	2	0.574036511156187	FALSE

f59eb777bfe41f27f84f114609d558ce	2	0.574036511156187	FALSE
44cbe35f49cc2825a1cab0c45875238b	2	0.574036511156187	FALSE
66781e54d7c70751dd4315324ee6a730	2	0.574036511156187	FALSE
5bdaad19f2a370833f353f14c372764e	2	0.574036511156187	FALSE
eef731495f535ba4c0e34f7b6827b01d	2	0.574036511156187	FALSE
65a1f76dd20322568fa79a52f095c4d3	2	0.574036511156187	FALSE
7f0e8f6049e044128bb02c2904584fe9	2	0.574036511156187	FALSE
addb5446f7a31c8057158bfbe2e88a25	2	0.574036511156187	FALSE
c8f0d849875f193a6dfb78ce2f923fac	2	0.574036511156187	FALSE
d1a66fbb0ffb55d8ca5bde15afd2cbfd	2	0.574036511156187	FALSE
b75f8c1ee5be34e1e86f0794da0994a6	2	0.574036511156187	FALSE
d60b57fe3931d10aff6007227c714b94	2	0.574036511156187	FALSE
b524bdb0093f4363f6dcc957c3bc0cab	2	0.574036511156187	FALSE
4a32f1635f88214c4874eec85df56595	2	0.574036511156187	FALSE
1bc6c19320b6c5d08d6af72f39ca7028	2	0.574036511156187	FALSE
12c7139b414de8443898fa448413c98e	2	0.574036511156187	FALSE
59555b720479b4dae97998e8573280ce	2	0.574036511156187	FALSE
3f3a185ee907d56d95b84ccb84399854	2	0.574036511156187	FALSE
8c311c27fc6e8803ed957220d8645a52	2	0.574036511156187	FALSE
d8b253da31cae9272be8c76108df2c79	2	0.574036511156187	FALSE
11f72383f466cffd3d9c00fb0ddfa187	2	0.574036511156187	FALSE
6bf6bc06c4696e95747f2584057622c3	2	0.574036511156187	FALSE
5f0af45dcc3031ff73fa133f78f01dff	2	0.574036511156187	FALSE
d7059e9440d7d26e2e445d56303678d2	2	0.574036511156187	FALSE
59b13e3e404561803ca77c7fe3d03a0a	45	0.579476602130643	FALSE
69e611251f4d8582e312afa5737f033e	46	0.596027827052081	FALSE
ddbb3a2a7d6486a2201bb8770feb0a6d	3	0.606936130213229	FALSE
f5a382df02639db40c978149c852f631	3	0.606936130213229	FALSE
3c862616eb99d4336427f8c121410e30	3	0.606936130213229	FALSE
327af6c728df4ea81eb33ebb1c7b1f9c	3	0.606936130213229	FALSE
a256dcba109fe74b499e46900f7297bd	3	0.606936130213229	FALSE
8dc44508eb6a496df987c76cf6a23e10	3	0.606936130213229	FALSE
4ae0b8cee8d20168e08cb4f11ab7aeaa	3	0.606936130213229	FALSE
0d3677300a537a199d53c388dd4f9d14	3	0.606936130213229	FALSE
efea489e9aeea8ea8714c557cca5599d	3	0.606936130213229	FALSE
0cdc9b91d67a45a312f99f2e1a3b1462	3	0.606936130213229	FALSE
25817204502b01664ae7e0b787a23d35	3	0.606936130213229	FALSE
3d95c89ac99d998a8702babf9a83b566	3	0.606936130213229	FALSE
b6d5dae42d6441946b72536202970b6f	3	0.606936130213229	FALSE
78643cfd45f5d0e81fab3682a85bbe11	3	0.606936130213229	FALSE
6d51a149e8639a5edc3818184b07ef43	3	0.606936130213229	FALSE
cee08a446ff68f17526d7e203bbe871e	3	0.606936130213229	FALSE
e2dc6ea9ee0035962b6ecdbc882c648f	3	0.606936130213229	FALSE
ee496894344fde402c4a74ee69ad20fe	3	0.606936130213229	FALSE
ae31c634072e81af29cf4f857b5ceeba	3	0.606936130213229	FALSE
4675c263dc11b27a89c8b1a740a2b528	3	0.606936130213229	FALSE
445dc3e0e095d35c29018c23a15e9aed	3	0.606936130213229	FALSE
2605f4046444e0b60c1bbc901c97f065	3	0.606936130213229	FALSE
788883e3b7ee47924346c610b2ab6014	3	0.606936130213229	FALSE
85b4d7d480ef06ef2844b93115d87480	3	0.606936130213229	FALSE
8a0c8e17b96eaf823d7fc5b6ba2fa1a2	3	0.606936130213229	FALSE
3d1f0db0a5df73cc2642739c4ee2b98c	3	0.606936130213229	FALSE
3b80c7cd38deabc389b31026c8195e2a	3	0.606936130213229	FALSE
03f2666d9f52d8fccec13d113af7f470	3	0.606936130213229	FALSE
90eaad185a24ca78f31616c482b66874	3	0.606936130213229	FALSE
41997260ae3f1fa6e7c669acf6fa32f2	3	0.606936130213229	FALSE
8a5c6d9fa02edda0653c94cdf83899ca	3	0.606936130213229	FALSE
daefb5b04252122ce0f8c0b63169fd36	3	0.606936130213229	FALSE
7c57923c256db3ea4aa63f12e5afcc9f	3	0.606936130213229	FALSE
438dc2a2ea2405a782df5830ae62100e	3	0.606936130213229	FALSE
77dd072d2525de28b3d827121cdf463a	3	0.606936130213229	FALSE
e5816f291546ca05fda640ec24b5c7e3	3	0.606936130213229	FALSE
652b78d7b987b1efc1917822209ba690	3	0.606936130213229	FALSE
a99f6029c214a53baa6613443940b67a	3	0.606936130213229	FALSE
519d34f37ef0123b10f482f21b11bf54	3	0.606936130213229	FALSE
ade61be54713fafdfc05977a921ec339	3	0.606936130213229	FALSE
2fd73e273a5ab911a9bab5cec85e8178	3	0.606936130213229	FALSE
72ee964de9b0144e80347bd4e7112cf9	3	0.606936130213229	FALSE
1eeb8eeacd8184a41db687812348d06c	3	0.606936130213229	FALSE
a6fd733e44ef6fa8048ef0094de67f28	3	0.606936130213229	FALSE
cceb354db8986b34d8c5b435adfc4324	3	0.606936130213229	FALSE
9cab98d9be1a58a0486d67ec723285f0	3	0.606936130213229	FALSE
ca5e711152517b591d9d48b783f4942b	3	0.606936130213229	FALSE
ded5b76b4b3e821ef40d5df3fb48c91a	3	0.606936130213229	FALSE
65ff839c902a244022b20e77f4aed679	3	0.606936130213229	FALSE
3e5b4c5f9ec53fa58f406bb4872c2400	3	0.606936130213229	FALSE
579c3508fb7953140df95b7964b55fa0	3	0.606936130213229	FALSE
1289b5c9697f69da8b8f9648e728bc50	3	0.606936130213229	FALSE
0a9a722df3a13268be23ddee2cc2a0f6	3	0.606936130213229	FALSE
209ba71d1bcc4949b4d1fabd2ab1d5aa	3	0.606936130213229	FALSE
e474c7483a3a1001e73c7aeadf5b8ee0	3	0.606936130213229	FALSE
929d9a7f88bd83bc06d889c356d9a13e	3	0.606936130213229	FALSE

47f1f3b66aafeaf2c4c7f9684ab7275d	3	0.606936130213229	FALSE
fb5bc4108d4ca6924d7bad409faff095	3	0.606936130213229	FALSE
94d028d1bdb01f40929f957b02afb576	3	0.606936130213229	FALSE
f889135f2c43218e4ddd55532ff76c3c	3	0.606936130213229	FALSE
85539c5100ea1d50723a53eb3099d883	3	0.606936130213229	FALSE
89b63341871a7ebd775e6f7064b1707d	3	0.606936130213229	FALSE
c1951d3e81e7f747aa634297072ea77d	3	0.606936130213229	FALSE
d71af0f02e2ca087c76c0a2b710c0945	3	0.606936130213229	FALSE
43152d438ac75bad2ec2ec1457fc6ac7	3	0.606936130213229	FALSE
0959782df74fea06fad1164f978fdd0e	3	0.606936130213229	FALSE
a3e2eb7398560e8e86867617d8011442	3	0.606936130213229	FALSE
a8195c108dec446f5c967078c25ebb74	3	0.606936130213229	FALSE
f79e9a78ab07909675c88e20cb0d95f5	3	0.606936130213229	FALSE
d92fcfcf215ad9fb193679b04a6737c0	3	0.606936130213229	FALSE
3136568cc32e1c6e2d0f114157863862	3	0.606936130213229	FALSE
7f68fd259107cfa9ac346ed09c5ef371	3	0.606936130213229	FALSE
13cdfd5b7da440810afbd5555701d91b	3	0.606936130213229	FALSE
3272f6b65b719378bb264a3740b2b40b	3	0.606936130213229	FALSE
fbb4b3994b2616a402100d1577ce4399	3	0.606936130213229	FALSE
55a225ab357fc87843d85d47fb8f2282	3	0.606936130213229	FALSE
c67d690916dc301d204b7ccb2bcc2ef8	3	0.606936130213229	FALSE
c1ae341aa77f14e7a1e3e62e413527da	3	0.606936130213229	FALSE
f39c1375b0565eda3b6660be85427c29	3	0.606936130213229	FALSE
7b6814cac868c1b645d0d8265e0fdd1c	3	0.606936130213229	FALSE
c6890b2e0e1c9ef31f93bff534d80700	3	0.606936130213229	FALSE
fe8d7e83f839d336881ec8419bb51293	3	0.606936130213229	FALSE
e0c17f6dde47055ed0caa4a7745e30c9	3	0.606936130213229	FALSE
02e0ce559228307ed79ccde3fe7b80ab	3	0.606936130213229	FALSE
151b90216c2f9ee35e184dafd9021130	3	0.606936130213229	FALSE
6babf4c5597da7990b82913ea7ac3ba2	3	0.606936130213229	FALSE
85a6f256aa6e1022a8e6bc648f01c037	3	0.606936130213229	FALSE
362d9d88f3195db02bbb580a020b0836	3	0.606936130213229	FALSE
b48900c23466c96cfaf4e4703c038566	3	0.606936130213229	FALSE
91090378e3a2b9e5e491fb0a627fb4c8	3	0.606936130213229	FALSE
72bb4b354b241a0bd9abd7caec6e9bae	3	0.606936130213229	FALSE
e548b7816111a16362f9dbfaf34be313	3	0.606936130213229	FALSE
eee80ff9268cc2e906d5b61786b66fbc	3	0.606936130213229	FALSE
b7dc1eedb15b4565b44c28c5fbbe49f5	3	0.606936130213229	FALSE
4e454938d9d1ae77d79ae8bf299a983f	3	0.606936130213229	FALSE
c56ac869661ff7a8d192fede12c318b2	3	0.606936130213229	FALSE
a4911447e5266f8a7d9bcef199267664	3	0.606936130213229	FALSE
1cf5017ed722b6a562933b84a7f7eeaf	3	0.606936130213229	FALSE
67496c58a44c58d60e084c6ae9f5ead7	3	0.606936130213229	FALSE
252e8532fee4f48af5e39f7514eb9d0b	3	0.606936130213229	FALSE
749b5b5cdf99a6ba907ad7e654702c7c	3	0.606936130213229	FALSE
b20f67d057bf23d44987c07876722fbf	3	0.606936130213229	FALSE
29a2ae2201cb196a28835102b6fad6f5	3	0.606936130213229	FALSE
5e94d08d6cbcbf35ab8d171568aa7196	3	0.606936130213229	FALSE
942bfb95d8f5f87f5a4340c64becbad0	3	0.606936130213229	FALSE
80565df1fb13bea7c5ffe8520f5add51	3	0.606936130213229	FALSE
9f7cd43f82859743a9fb6294b413138a	3	0.606936130213229	FALSE
6faaa11948882a569d8900983904d12e	3	0.606936130213229	FALSE
a4416bcf0ce3d2e25b3db4e987f4d88f	3	0.606936130213229	FALSE
7a1fbb0c1134ad5443dd743ea4b6f3ac	3	0.606936130213229	FALSE
5719b9b59cc1ec3e172cc9b9f3f0730f	47	0.612204436863422	FALSE
e59eb4f8c8550ddeb4ba43baf144ae11	19	0.612983675440831	FALSE
d56403223c8765b24812c19f1f844349	19	0.612983675440831	FALSE
6d3e98290cbed32480eb921f276470a5	19	0.612983675440831	FALSE
860c2d10b074762187a1f323f5f22558	19	0.612983675440831	FALSE
38bfbd3a2cf83da8fe03cea0f6b4f5f3	19	0.612983675440831	FALSE
760eb607a6f9eabf73fc3bc008113f73	19	0.612983675440831	FALSE
92ac8b57e0db3e849b7c8b0df7802639	19	0.612983675440831	FALSE
da1f560e36ff5523b6eae1acb26e7799	20	0.635090590400632	FALSE
49dc837f8fb48a7b37318967391f96d8	20	0.635090590400632	FALSE
834d07b2a02c48a75e52cd3d63fc7315	162	0.635262861075058	FALSE
9178843fe6c1b2b119e81a324c06e5ed	4	0.637356551907521	FALSE
79065bc5c081ea35aac41a59226dfaff	4	0.637356551907521	FALSE
857690bde71e50e3f9d91d2b77c8e675	4	0.637356551907521	FALSE
144e1bdc5d10e64c663d3833d416183b	4	0.637356551907521	FALSE
e40124c65379aab7ddc74c3d1c35213f	4	0.637356551907521	FALSE
6829b5946a95b929e9904067d572db6f	4	0.637356551907521	FALSE
7fd8ac343154ef6966a03f376782be92	4	0.637356551907521	FALSE
8419a2a1403b971c044d92d057e01ecb	4	0.637356551907521	FALSE
4313fef965a323b897e452786b227480	4	0.637356551907521	FALSE
4e7b984fa0360b99ffee1ceb10a122d3	4	0.637356551907521	FALSE
258f50eeaf72af21d70c648397c668b6	4	0.637356551907521	FALSE
36b69044091011464daa0325da37f61a	4	0.637356551907521	FALSE
dc5030eb18fe395a50b8235f2e583dbd	4	0.637356551907521	FALSE
4b95050efbe5bb490a63b0410c951326	4	0.637356551907521	FALSE
25e956ad84aba7ccf51f84544fe3b8d6	4	0.637356551907521	FALSE
3837a63a524a7a187121536fff409284	4	0.637356551907521	FALSE
1cd63754d2a45e4da3e2c17c07c39931	4	0.637356551907521	FALSE

105e6198e75d958ec2beeb64369687e8	4	0.637356551907521	FALSE
42b8c9335747b0c86fc68d8adfce8315	4	0.637356551907521	FALSE
9654b46bd802f2c2748ecc5e77d97f21	4	0.637356551907521	FALSE
b7c98a016051abbbc602fa6163280ef8	4	0.637356551907521	FALSE
8c40ae61863ad1094a5fe3e7a1e56ee4	4	0.637356551907521	FALSE
e5fbcec9c3476c84ce40270b6f0bf4ed	4	0.637356551907521	FALSE
3cf8e3f2936c2215ad012e031a11dfc8	4	0.637356551907521	FALSE
5cc66b33870d22cf990e6a8ca618bb2a	4	0.637356551907521	FALSE
cad4e11393fe489023ae6a9724d6894d	4	0.637356551907521	FALSE
77f21f2da2d7b5fd7e7730b964e1ca40	4	0.637356551907521	FALSE
a509a05ddfc4a6df40cd9b1e75cb5f84	4	0.637356551907521	FALSE
618799965cb9fcfae385cc63346f300f	4	0.637356551907521	FALSE
bfd1c34f739028e507f085cdb991a0a2	4	0.637356551907521	FALSE
8d790eff268748c8792ab4fd07100e8a	4	0.637356551907521	FALSE
418d67cfd71b0f894bd6752f91a55124	4	0.637356551907521	FALSE
3f33b7ba47ff472f3dfa37699b3d4945	4	0.637356551907521	FALSE
fc1b76278975ee14760e108221ce14f0	4	0.637356551907521	FALSE
e56bd9b14d748dde0d00bfcba0c059b3	4	0.637356551907521	FALSE
ee5c297bd9fff3db3dca564f4de2b083	4	0.637356551907521	FALSE
392be618576de3abfe491746b24b64fc	4	0.637356551907521	FALSE
402e5913597695a16d7cad415ffff02f	4	0.637356551907521	FALSE
bfedbe08a11c47b4f1fba543a22b5d75	4	0.637356551907521	FALSE
0a9507c499d16037a527e88e32e7555a	4	0.637356551907521	FALSE
89623e037318e0f10a2b26123022dc25	4	0.637356551907521	FALSE
7f0a3c1eb166fa8b6b8c6169d5d2ee21	4	0.637356551907521	FALSE
5228eaef5a2de5ec5faecaf127b4488b	4	0.637356551907521	FALSE
2f1a6634dba0fcf6e04a10a0ab23bee9	4	0.637356551907521	FALSE
ccc5b420e1a3e5b891e8a467366084ee	4	0.637356551907521	FALSE
459a2ee9b40cae14ff8b1154046a45bf	4	0.637356551907521	FALSE
5ac0ca1b313546c59510815ec598f6d4	4	0.637356551907521	FALSE
9a1299533fdc202ea9dd1f8145e15c45	4	0.637356551907521	FALSE
1320e90134d8dd811304a92366e035b4	4	0.637356551907521	FALSE
7b25dfc72ef88a31085ef11f520cbc9e	4	0.637356551907521	FALSE
d6f114c9b544a83d9650ee4091d3c8e6	4	0.637356551907521	FALSE
6dcfd39fe967d9a976c330487e0ad0f0	4	0.637356551907521	FALSE
3cd349d8fddf16a5902fb77218065d47	4	0.637356551907521	FALSE
98976c388182f72eaf6444f252df2c4b	4	0.637356551907521	FALSE
f83d060cdb08f3a5c14531d80cb10d90	4	0.637356551907521	FALSE
7bb75a908f84cea53943cd1359b1deda	4	0.637356551907521	FALSE
1d4755131d0455f2ec5886e574308f11	4	0.637356551907521	FALSE
aba879ff52d1205f49a5c7c257679f61	4	0.637356551907521	FALSE
32e4f11674dad6bcd7909e3a195f3f3c	4	0.637356551907521	FALSE
f34bde4e8b265f13ee0a2e4b2e72532b	4	0.637356551907521	FALSE
39a7609929b77123cfe01080cd3a8ac8	4	0.637356551907521	FALSE
c6ff7bf11a21be39a971d86ac3f5ff02	4	0.637356551907521	FALSE
768d8ea38ea385a980b2218b8ccc6966	4	0.637356551907521	FALSE
391d3538ad8650017b13fe98bde557a8	4	0.637356551907521	FALSE
2178c86013f371f34ca8b6d82283dae9	4	0.637356551907521	FALSE
2bd44a9b4d580c5af16e7858b5554f05	4	0.637356551907521	FALSE
ece5f619b7415de3b8df0c94e1e708b0	4	0.637356551907521	FALSE
1b4572cc774d5173d5e09c03dbbca0eb	4	0.637356551907521	FALSE
88ab02c4a5b3b7fde63cdc6a21bdb6a5	4	0.637356551907521	FALSE
941e623512e7f598fc9942eaae0a6e70	4	0.637356551907521	FALSE
044e0092f4724300a52cce5c5fd4e882	4	0.637356551907521	FALSE
31921717848a5efcb875586d869f53f9	4	0.637356551907521	FALSE
71decf7564882efd1d2190fd29460d40	4	0.637356551907521	FALSE
16babc126b1836db96e5b5f9d59bc8a7	4	0.637356551907521	FALSE
31f7982d2607c5b3685d6c3974f2ed79	4	0.637356551907521	FALSE
8d4c51764c41d581fadd36ab1a7db29d	4	0.637356551907521	FALSE
30077f5042a2a53449f3afff0a7f21b7	4	0.637356551907521	FALSE
7cc6f23189123997fb56b704764660d8	4	0.637356551907521	FALSE
420001a74ca908712b4924c90f2fa112	4	0.637356551907521	FALSE
15a9309869945ab937c92fbe690903ef	4	0.637356551907521	FALSE
e503811c021343f5b11caed81d435f07	4	0.637356551907521	FALSE
4ce4bc11c99a28b0fe57853364b8451e	4	0.637356551907521	FALSE
adc1e763a2e0ce37c934f0ff2e25940d	4	0.637356551907521	FALSE
888f1e27e68fcfc06512e9bff356a16e	4	0.637356551907521	FALSE
b0d17476ed8f57285dc3fdf68d30d84c	4	0.637356551907521	FALSE
65e5847f2fe022a965dc8d4fca56434f	4	0.637356551907521	FALSE
06e10170152a7df3a946a557400c4bd7	4	0.637356551907521	FALSE
5e3736d7500f1847fde308557ffbcfdd	4	0.637356551907521	FALSE
fb3ace8db3915ddb8eecd36a0574f2f1	4	0.637356551907521	FALSE
63a00abba59283d87fa649e4b991da6e	4	0.637356551907521	FALSE
0a11bdf36506b66e44139ff447e2dead	4	0.637356551907521	FALSE
6c75cfbcda95ba7be4b96bd18203552b	4	0.637356551907521	FALSE
e0196d786487dae63ea300ec2eac2dad	4	0.637356551907521	FALSE
0ff344210a06866101d371ddca1193af	4	0.637356551907521	FALSE
41c984e77f65b6f4e3a141b213bb652e	4	0.637356551907521	FALSE
d51c2d5527534ea09b7c9279d8df7bb3	4	0.637356551907521	FALSE
8f62e1ce476fc35015f544e35bf6b791	4	0.637356551907521	FALSE
a10540eb078d6525325fbe1f2a607387	4	0.637356551907521	FALSE
a02a6468b6682c4ab0f0b31b8e080a30	4	0.637356551907521	FALSE

341d0af40ca4d417d5d4b795ecb0cf34	4	0.637356551907521	FALSE
64301da69a74b94ac07fc109172f2c1a	4	0.637356551907521	FALSE
264e7ee6f0bf9bd1888f5b1bc40e18c9	4	0.637356551907521	FALSE
3e30a1f52cc81e130b8c023988acf9d5	4	0.637356551907521	FALSE
0ea6c0d1c3fc13d6a4d01653e44f19e4	4	0.637356551907521	FALSE
cf4b69428c4fbe0a55b81d9cfcf7604d	4	0.637356551907521	FALSE
3d06d03c32c7b6782a377199e96f576e	4	0.637356551907521	FALSE
a35640f17ff92ea37bea4104d9bb7f1e	4	0.637356551907521	FALSE
9fdb12014b64c13ab70cac6ab62b02ef	4	0.637356551907521	FALSE
803a31819695ae771de888afe8af7e71	4	0.637356551907521	FALSE
290d9dfd81df5f7192e22b2b347f4120	4	0.637356551907521	FALSE
1734c3e181110615bded5b21025d3976	4	0.637356551907521	FALSE
91f9cd9795f7b8793d9e06af80b0d56d	4	0.637356551907521	FALSE
0af9cf4f3e0a17840c19fda47ff11769	4	0.637356551907521	FALSE
202df81eb46cfff330eeeecc7e00092e	4	0.637356551907521	FALSE
ab61dbbc92d649f771918bab6ab7bbd1	4	0.637356551907521	FALSE
1056f4b6efc9797605ca25674fac9912	4	0.637356551907521	FALSE
45c63731d8dbb3b9e663139022627d43	4	0.637356551907521	FALSE
c1b4edf475fac73cbb145b05d22e6e3a	4	0.637356551907521	FALSE
b1d39025553d5a2e77eed0fd245eee5d	4	0.637356551907521	FALSE
0c3ca4bdbcc55a1eaf440eb73dd01a8a	4	0.637356551907521	FALSE
0a0f6a64b9bca840e46c7285b457c36b	4	0.637356551907521	FALSE
7cbbaefaca7a9bbb37376880e4a0f14e	4	0.637356551907521	FALSE
87118b7c468f54f714cd3a4701f728a4	4	0.637356551907521	FALSE
c6aae8ae72923155732f2e48a26b2fb9	4	0.637356551907521	FALSE
6359fb9ad7c1e779f94c583a2ec6fce5	4	0.637356551907521	FALSE
3bab94bbc9e095798d906d0afdc6d67f	4	0.637356551907521	FALSE
3752c0937de8a03bbbfc765819c78099	4	0.637356551907521	FALSE
c967baaf09da450bd29583233ea1a781	4	0.637356551907521	FALSE
c0c03235e8911295a37e19ba1c0af1a3	4	0.637356551907521	FALSE
b73ff1487c71f12a47318e8e9df59f04	4	0.637356551907521	FALSE
90422dff863388c4c097236cdfe729f4	4	0.637356551907521	FALSE
dbf3b32e6fcd7f6db830760df87aa435	4	0.637356551907521	FALSE
f2cc6eac9ecbcfa96ebd1c178e4ec6a2	4	0.637356551907521	FALSE
052bea98ea58d64bc7e0d792d4bb43c8	4	0.637356551907521	FALSE
b4e05e11ee1b2518be2326d1972121d7	4	0.637356551907521	FALSE
7dbb203fe0dfef7a33364e079154fc20	4	0.637356551907521	FALSE
d39d3f4f4d4881c0b75864dbe733de6d	4	0.637356551907521	FALSE
00f2f103259e0d2d279bec1899a683d5	4	0.637356551907521	FALSE
fc1c8932bfec8f0f632c63b5095bb577	4	0.637356551907521	FALSE
22872cce3904e3a7a73c15e4e63fcc2a	4	0.637356551907521	FALSE
de260172685c660a90923d290b078fd3	4	0.637356551907521	FALSE
38316233d8ebd5de6fdb0b87bdf0c285	4	0.637356551907521	FALSE
d482a8fc557bc969959b0c2a704c2c16	4	0.637356551907521	FALSE
79813aad3f28c522fd69a1e1e7f5892f	4	0.637356551907521	FALSE
ef628a53847821349bbe7988bcafb924	4	0.637356551907521	FALSE
85cfaa63370df8961eebc7b05ba076fb	4	0.637356551907521	FALSE
96416a69b1811ba8778fa127f20a3b15	4	0.637356551907521	FALSE
8efc96acc5fc0d9b6bda279be150150b	4	0.637356551907521	FALSE
e4ab5016c0eac0e4fbb1c8ea75b0ca28	4	0.637356551907521	FALSE
2aab0c408ab7e997e07d713b70cc5824	4	0.637356551907521	FALSE
6854a1092addd43549b415370cdb2d0f	4	0.637356551907521	FALSE
d31fb970ff9b6217462a4e0e52ca4b5e	4	0.637356551907521	FALSE
a88c5da1937f04d0c44ef7243e9476cb	4	0.637356551907521	FALSE
8cf45fe9e1251163b5d801841857a873	4	0.637356551907521	FALSE
a3699e0b9b431b8f11e1065820deaeb6	4	0.637356551907521	FALSE
901e24ec7d14fc177f7f9c15ab0f972d	4	0.637356551907521	FALSE
9a11856898d64d5906fca69779018c7d	4	0.637356551907521	FALSE
46e6034076b1f5aa371734d5c41605c8	4	0.637356551907521	FALSE
9232ae2e99a9314f625e80cc5f7a1b77	4	0.637356551907521	FALSE
15d8de3365192fca85622a7366050ee6	4	0.637356551907521	FALSE
8081227a3285bf886a9aeb207a890c38	4	0.637356551907521	FALSE
0cf93a42d2f2697d21739461b118851b	4	0.637356551907521	FALSE
3baabf8cc3e85c0f8b1d2790db20f838	229	0.647158144172996	FALSE
77d6a7db6de3bb1f15a02fdb36f90627	69	0.652990903333722	FALSE
eac36cc96b0e7277d4a485dbe6199beb	21	0.656163025642436	FALSE
d31af52f792634b80a89d3d310e619b4	21	0.656163025642436	FALSE
8f8539ce9266ce2c572b682f190cc810	50	0.658378182086215	FALSE
26c9f4f844da09bdd5c1603be193969c	36	0.662109791353658	FALSE
a1a3200b76bcd6000a0914892d370b6e	5	0.665479921351427	FALSE
442cc2db14651564914488bfdffd5071	5	0.665479921351427	FALSE
4c91e1ea3c1279e207a971df915d6178	5	0.665479921351427	FALSE
d6819c57d6aacc8113578b04b0911dc2	5	0.665479921351427	FALSE
79639ce66720319d718c17711fae3704	5	0.665479921351427	FALSE
8bfe73e7d3c17f599aec113950d751de	5	0.665479921351427	FALSE
501c1cf67a877c81057dd3fb330ddc18	5	0.665479921351427	FALSE
a412a5462ab0b34fa3b0b6dcdfa66b7f	5	0.665479921351427	FALSE
5ba826b6728c5f7a97293ac6c310a446	5	0.665479921351427	FALSE
e5ca098c7ee4d0fc9ff60e16e577c2b0	5	0.665479921351427	FALSE
03efc0161abb4e11e93f1870e9d5df40	5	0.665479921351427	FALSE
4b2862d8ddf6848328ccc64dbc6af0c2	5	0.665479921351427	FALSE
985e584faf3be7a41d1bca9d8e598817	5	0.665479921351427	FALSE

56d75f22814ed2e9bb37c1452b26bc10	5	0.665479921351427	FALSE
37ec4bfd1c6ad9de0c6feef00d98d8a1	5	0.665479921351427	FALSE
800f4b5ec4543c7757ff29f6d5941d39	5	0.665479921351427	FALSE
ed40b08eedec4f873d38551aa43b0fde	5	0.665479921351427	FALSE
1dbd3332661515bcd3aef32a9b9e43d1	5	0.665479921351427	FALSE
621f0a4f8c2414b17f3b40434862a251	5	0.665479921351427	FALSE
c504e0ca94534572016a31761f44776b	5	0.665479921351427	FALSE
9b1a22ceee6ecfbdd684d46412f9dc49	5	0.665479921351427	FALSE
3bd445c971cbfbe4d6f12c07a68deec7	5	0.665479921351427	FALSE
72cef79e08aeb54df80915bab3ee3c01	5	0.665479921351427	FALSE
694ee281b908e4bfceb8d88584224367	5	0.665479921351427	FALSE
805599865882c8802a1ebbc3d539b68a	5	0.665479921351427	FALSE
bc4de31fa467e0b792b58e230e7cee7a	5	0.665479921351427	FALSE
51716d6182efdb29a158fe9562c83312	5	0.665479921351427	FALSE
71ad47c3b8cca9e31443d2370f72b32f	5	0.665479921351427	FALSE
d60a0925b76d5201afe16641d9cc1992	5	0.665479921351427	FALSE
2f267743f640a6790a563ddfbd2bf647	5	0.665479921351427	FALSE
d5c502312559578912b7c07f862f08a7	5	0.665479921351427	FALSE
d473f30bb25b09ce020fabb57f4ce14a	5	0.665479921351427	FALSE
a05f9f73ca792d7702b8dc6521d169b6	5	0.665479921351427	FALSE
99ffc98eaa2402b754b593ee3769ceb1	5	0.665479921351427	FALSE
382e9f4d997c89d86390ff1db5eac316	5	0.665479921351427	FALSE
1e5a5b3bc1ec5dcac873a685e4efed1d	5	0.665479921351427	FALSE
448092b5340d77b71bc09f3b5f6ba580	5	0.665479921351427	FALSE
90ce0670d0c5674699a2cd54638a8f13	5	0.665479921351427	FALSE
db6999c4df54a3eee3136c6671fa3517	5	0.665479921351427	FALSE
0b22ed48eb3acaf7d1d9721c8b83cbdf	5	0.665479921351427	FALSE
69a0a9b3d2f25e615792f49a88477b76	5	0.665479921351427	FALSE
e738239f47d0c43f0d59b1bbd3e5b262	5	0.665479921351427	FALSE
cb16531b22a650335c98dcdc66c2dafd	5	0.665479921351427	FALSE
84ec1399e8ee3618cc0686f28b89dbcb	5	0.665479921351427	FALSE
b5b5ccf8d4b597516a51069273afe7c8	5	0.665479921351427	FALSE
8ecb3c546a958d83e04d925a962af7f6	5	0.665479921351427	FALSE
9ef7688ddcbba5179a5011ec2c64a228	5	0.665479921351427	FALSE
08bb29e76019a1d1ab85c15f745c3b07	5	0.665479921351427	FALSE
f2c5e1386e7119686922dadc60904088	5	0.665479921351427	FALSE
6552f4787f1b6115bf9c9cf04a0b836d	5	0.665479921351427	FALSE
9cb31a8163c8b8dc83ddb589443f06e1	5	0.665479921351427	FALSE
96145f0f0d8025639c22921be63a5696	5	0.665479921351427	FALSE
be5ee53525a2da42755c32880c374493	5	0.665479921351427	FALSE
933096a8abba3ba26115619300c7b569	5	0.665479921351427	FALSE
13e78875b36fe62a172099127b50eca7	5	0.665479921351427	FALSE
213125af63c3c90e20e0e9e71cf103e5	5	0.665479921351427	FALSE
d32ab6db74a0b97252425a6b4d99b4a9	5	0.665479921351427	FALSE
3fa9b1137ccd47fc0b8a5960215cd91d	5	0.665479921351427	FALSE
b5ca209c65510a1622f68fa535441443	5	0.665479921351427	FALSE
e747680849d7059f0721a86f541a58aa	5	0.665479921351427	FALSE
fcdf3e1e3fd860c9a76a22fe6da59882	5	0.665479921351427	FALSE
7ef97632ab2d719134190b64d0e20481	5	0.665479921351427	FALSE
8c69b52248230f6661f7718a1dff9f26	5	0.665479921351427	FALSE
5c2fe78d3aa8a5952c3261269b857cc1	5	0.665479921351427	FALSE
3bdf3962a4d6b9738dc1bfa095f6cc8e	5	0.665479921351427	FALSE
d0099ee91dbd9830ee9ca81d0ee85747	5	0.665479921351427	FALSE
93da342980ee5d7587028b142860e326	5	0.665479921351427	FALSE
f8bfa38966ea4a31ede4d309eff80d6f	5	0.665479921351427	FALSE
1a7a8f466b74b6b5e3497d10112cda38	5	0.665479921351427	FALSE
0e86e0627c7da65f03262253a0f36a37	5	0.665479921351427	FALSE
5f8fcfd5eae442ef695287a85c782c5d	5	0.665479921351427	FALSE
b3f501eb848286a3758e9ce88eb51d01	5	0.665479921351427	FALSE
c9afd558a3687a3ab1cb10e0979c6e26	5	0.665479921351427	FALSE
f795c30a2d4c83ad90760a26b8948100	5	0.665479921351427	FALSE
763da75108822f3211c6f8852fecb09d	5	0.665479921351427	FALSE
0ea5e1ee42a9add6e20e4cea1e13e61b	5	0.665479921351427	FALSE
fc16f182cda13158b7b53b99ec439473	5	0.665479921351427	FALSE
937130634699adf72e663b3206296108	5	0.665479921351427	FALSE
684661c135c9eac8d14167f4c0f04940	5	0.665479921351427	FALSE
a2a66718d4e7cf14e217a76f6d99900d	5	0.665479921351427	FALSE
25b00fbc430d7f939bd949e6460a84dd	5	0.665479921351427	FALSE
84a73b80b8405e0ddf23fcd9f218acbc	5	0.665479921351427	FALSE
e5ba1449f96ae19c21538f0ebf01b59e	5	0.665479921351427	FALSE
2deee102fe6514204af2bf0780bc1ac4	5	0.665479921351427	FALSE
b987b751d2357f99e82610cc984b09e9	5	0.665479921351427	FALSE
5e6725e17a517802fdf48cba3bc05c1c	5	0.665479921351427	FALSE
2fc9b7df35cf9b721acedcd9d3561cce	5	0.665479921351427	FALSE
23a7b18fbefa1fa371830e7db7876b29	5	0.665479921351427	FALSE
62a87d4db851644446615062d61d09e7	5	0.665479921351427	FALSE
99d5a4ffa07fc3904d57784b335f19bd	5	0.665479921351427	FALSE
f0b16686cde37268349599e11a1f8790	5	0.665479921351427	FALSE
934f56716628cf3758baa5e7b702d2a1	5	0.665479921351427	FALSE
05583e0e12acb4607a40b2f17ce39e18	5	0.665479921351427	FALSE
aec20e1a147e3f84f9794c1b9066aae4	5	0.665479921351427	FALSE
3b09d01fdbc5a425b7e984a53f59a9d8	5	0.665479921351427	FALSE

4581c03a9fbf22ca8d26d197945c5d06	5	0.665479921351427	FALSE
0e089dac21915d34e1ba6011443df2e9	5	0.665479921351427	FALSE
09b47236ecdcbe4627b4f19dadfb2867	5	0.665479921351427	FALSE
57706cd140992bbd0d4e96a833912f45	5	0.665479921351427	FALSE
0d2a4db996f43b65f6249c118c8d424c	5	0.665479921351427	FALSE
1dee7a9043af345aeb0c01ef1f4473ae	5	0.665479921351427	FALSE
0f21e6a5615139dabd413632d1378dc6	5	0.665479921351427	FALSE
9bf47859277b18f7164e848576d8829f	5	0.665479921351427	FALSE
9243a3b81d2e0ffe7466f757feccb47f	5	0.665479921351427	FALSE
687bb3b3b1b09601bd3a0ac6eecaf46f	5	0.665479921351427	FALSE
dff5823839d97156151935cda410bb6f	5	0.665479921351427	FALSE
d6eb49be064bb019a8dbb28451f649c3	5	0.665479921351427	FALSE
0bab6eefa020ad40122cff733972d7f3	5	0.665479921351427	FALSE
7b732016b53fc4c4128273ac1c83fae2	5	0.665479921351427	FALSE
6875c5259a37bc8844c092db19fec963	5	0.665479921351427	FALSE
1a2d11c18878fcdf1192fb22e7e45a93	5	0.665479921351427	FALSE
637acbfe700723da791ff81b54634740	5	0.665479921351427	FALSE
4fe609069ddb659da47531c688130164	5	0.665479921351427	FALSE
7b897f5ab602ed700ec9c10dd96fb997	5	0.665479921351427	FALSE
1b097ec2f993f8d93f006bdd6499a2b9	5	0.665479921351427	FALSE
3615fb777005e792f48281d9766a159a	5	0.665479921351427	FALSE
0b49776f60bc0f566ebe8585ec3c5e07	5	0.665479921351427	FALSE
c45fba966a7c86503b38a13e121fad0a	5	0.665479921351427	FALSE
e39d0106129d2cef95d226ab68163276	5	0.665479921351427	FALSE
69e91084b18e0bcb7404b6a55fc3b17f	5	0.665479921351427	FALSE
e30af441c32096bbff179e10a8d80c75	5	0.665479921351427	FALSE
a3fdee62edf9cb50662e84281124a241	5	0.665479921351427	FALSE
12b3baee9905c9965794265d33f8511e	5	0.665479921351427	FALSE
306a0dc859c17f4586aac1cbef4f7d45	5	0.665479921351427	FALSE
af7584c5080425da5c5b2bf7a2045749	5	0.665479921351427	FALSE
8fea6fb414f3973c60bb3d9c9b396391	5	0.665479921351427	FALSE
858e61f4b80d4c21c57fb23e4adf00b9	5	0.665479921351427	FALSE
f42fa1f361123808c5170776beea4f28	5	0.665479921351427	FALSE
a3d55180faf1984a6ecd4e4cf7cd9621	5	0.665479921351427	FALSE
833e68d8409bfed100f147d62868285d	5	0.665479921351427	FALSE
bada4b4d5ec4bb02547d109017bddbf6	5	0.665479921351427	FALSE
1de11ca9bfc49d2a24f3217092474de5	22	0.676227110786361	FALSE
e5d49fa90328817f763cd8e8f5b0eab1	22	0.676227110786361	FALSE
6da46d83d8eca17871b9937d084ea890	22	0.676227110786361	FALSE
bab727aead750abea99848d1f7b760d7	71	0.678242374515425	FALSE
3b482b3f9da81db80a221a66031aa159	113	0.684135587722467	FALSE
476738e317c846bab51ab3cbefc92f1d	6	0.691475346686081	FALSE
39ca85a8156900264d039677c05ca2e8	6	0.691475346686081	FALSE
db0a9bba4758c990e4d72f8558596479	6	0.691475346686081	FALSE
c00181a21a8c5b8b22d1b2793f6fdfb3	6	0.691475346686081	FALSE
0984f915f803bc5c24bf8e1c909ab5ca	6	0.691475346686081	FALSE
81d51386cb12c21a2f27ebdd8b9b2ccc	6	0.691475346686081	FALSE
375f634194a84d8e75ae82cf5a65769f	6	0.691475346686081	FALSE
642cd6c8b3c6a14c90307513a1249ea7	6	0.691475346686081	FALSE
ff4647415dccadee59cb477abc5de829	6	0.691475346686081	FALSE
771eef8a8acb8fe4bab03d049dd070fa	6	0.691475346686081	FALSE
fc48628625c6ec016a4d57e4e6d3d5af	6	0.691475346686081	FALSE
a810d316616fb1a7321351393c70ae70	6	0.691475346686081	FALSE
42adb322e812d7d110c13bc5c582464f	6	0.691475346686081	FALSE
f54538c9fe78a2491a7365d46ac7d04a	6	0.691475346686081	FALSE
5440bb497433d6fb4d592fc9dc4115fc	6	0.691475346686081	FALSE
846d9bbb062cdef518cf911cb5f2f48e	6	0.691475346686081	FALSE
bfd5a200f7cbc8ed661fb5e5659b809e	6	0.691475346686081	FALSE
d7d8e9ca0bb47e48bb7b11010bf44cd9	6	0.691475346686081	FALSE
19032423b2abbba6747425ffe54db960	6	0.691475346686081	FALSE
24578f723e54aac8e9d917365bcf8d87	6	0.691475346686081	FALSE
cf82d65756dce349e1dfeb64ab983821	6	0.691475346686081	FALSE
9c6f2b50ecee1d000f4981af2d9ab517	6	0.691475346686081	FALSE
b7a90c7afe20bf32a35c949e18984553	6	0.691475346686081	FALSE
2cefc396eb5f251d5f5142811640f757	6	0.691475346686081	FALSE
128494a261f2bae571bb1058b52c687a	6	0.691475346686081	FALSE
b493b2e590a925aa8aa5e8e5ef559dfd	6	0.691475346686081	FALSE
6645ac6e01b54dbf89dcb1a3515e387f	6	0.691475346686081	FALSE
eeb6eda609da001bc1cda61fcdb1f67b	6	0.691475346686081	FALSE
0fc819b99aeeaa0affa6d9db40378b17	6	0.691475346686081	FALSE
d89b8bf4cf0e4e17fe46c0d214238584	6	0.691475346686081	FALSE
20ed485a649cc0f03dd3cf2b9a488a22	6	0.691475346686081	FALSE
74df131c4c31e7469f7f71f4d93cf57d	6	0.691475346686081	FALSE
64a76d1d022bade634287ace4305d5c4	6	0.691475346686081	FALSE
99b0ace8f5564cc9f51d62e073d1389f	6	0.691475346686081	FALSE
c05eef21fa88381de2bcb04b003bcad6	6	0.691475346686081	FALSE
38e37205b8f101040fafa39445a00687	6	0.691475346686081	FALSE
7e28ca07fa82d553ff3e2eb951f96b1e	6	0.691475346686081	FALSE
2862752121447c336cdaee1089c3bf9d	6	0.691475346686081	FALSE
cfeaa32bd4779c561a79d9ca355a8a21	6	0.691475346686081	FALSE
d6a07a207695e918c576157088e00158	6	0.691475346686081	FALSE
69d658abc0bc72ff78e012446a0620d0	6	0.691475346686081	FALSE

78c77fe65ef85f77876913afafaf83ca	6	0.691475346686081	FALSE
21f57b10a24af2d07ef8bb29fde77308	6	0.691475346686081	FALSE
fbe1441247a9f5ff2154e296ce755b02	6	0.691475346686081	FALSE
19faab0613301c627436bea7ba130c03	6	0.691475346686081	FALSE
c372b43be47a60debddb8ba64f5c4f3b	6	0.691475346686081	FALSE
4281f5b1a9d4da51b4f84a86734e3016	6	0.691475346686081	FALSE
33fb7c931529eafff0ba2653fe5e55a4	6	0.691475346686081	FALSE
54eaa3dad1c82a5c1a864cb36dfa00a8	6	0.691475346686081	FALSE
09654246eee58793bd55f4f2e8866c94	6	0.691475346686081	FALSE
1554445ec0f58f8f3aa89dd4c19f6ca5	6	0.691475346686081	FALSE
725c3ce493fc20f290177b3cd00ac157	6	0.691475346686081	FALSE
d17be2e7da52698326ae236a7e80bf0c	6	0.691475346686081	FALSE
5f8ae198552bc39633ea5c3962946313	6	0.691475346686081	FALSE
07b2282aba42b7a552743011db8094f6	6	0.691475346686081	FALSE
3e9db007ba1ad11c10840b072cf82ac9	6	0.691475346686081	FALSE
ba2abd56e0c62e84a281b252c0ecf2c4	6	0.691475346686081	FALSE
ac58e247b4b4dc9952a63a8768842531	6	0.691475346686081	FALSE
f0c33566b57c73834e6715e4f843280f	6	0.691475346686081	FALSE
3447bba88a77090a6e1f60be2df80a8d	6	0.691475346686081	FALSE
c9495d05babd9ff3d12861c52ac48b10	6	0.691475346686081	FALSE
cf1c154dda5617a313977704c70b4fea	6	0.691475346686081	FALSE
665c06c0446675030ffb92788fbc2972	6	0.691475346686081	FALSE
de755d7719f17159286b670fb8ec8452	6	0.691475346686081	FALSE
f4009a122f9d6677ef2cc595acd70db1	6	0.691475346686081	FALSE
023f6419e31912178c881067c038cbe8	6	0.691475346686081	FALSE
0ba7ab54477ac1e358b95b12ae47cfcd	6	0.691475346686081	FALSE
e5efb19c8e76cad58107d2acd1913b91	6	0.691475346686081	FALSE
282cf87787bf8bc13d8469ce93ea9a83	6	0.691475346686081	FALSE
37593875f248af0a9f8a410adae37818	6	0.691475346686081	FALSE
e53ed171ef0054d5828f13109e670473	6	0.691475346686081	FALSE
0122b585024361ddaee2c8d00f210974	6	0.691475346686081	FALSE
88a665d3728e9f8cb9660a1ce9b3c3bf	6	0.691475346686081	FALSE
05e59e6acfa67481514206aea60b5817	6	0.691475346686081	FALSE
ce83fcf423e41adb5b0ed8d2ef7c692b	6	0.691475346686081	FALSE
9c23f56aa5a85f3d6ff72ab45dd6a8eb	6	0.691475346686081	FALSE
e6bbc8eaac8196675f99a78761aefab9	6	0.691475346686081	FALSE
6bc1b948bdac137349310bd8e6822f34	6	0.691475346686081	FALSE
e41b96f0ca69f99a45f8985d840fde43	6	0.691475346686081	FALSE
4202b2ba3e89e89d60d5e1657251a277	6	0.691475346686081	FALSE
3a75c7fcc9371c1129d5e490d67b5473	6	0.691475346686081	FALSE
302e745422686c8b40fecf517ec856ca	6	0.691475346686081	FALSE
d1cb4ea8ba58a5e6d1be97779e72dd3b	6	0.691475346686081	FALSE
5b115c09c95c3a6b956d53d2f1884614	6	0.691475346686081	FALSE
fac0a19f742f17a596e1554f6f94542e	6	0.691475346686081	FALSE
92ea3b676285053413ddbf4880f2a4d5	6	0.691475346686081	FALSE
8f339184fac227ee4142a3554f5cc64f	6	0.691475346686081	FALSE
f2b214c47f5b29b3edb2894e3fd95dca	6	0.691475346686081	FALSE
1e85713d0ee4b69c1ba7b57fecce1e7e	6	0.691475346686081	FALSE
1ae8041320c459acbb58d90de8a1b346	6	0.691475346686081	FALSE
f44603f6b65d6c112a977783a13461f5	6	0.691475346686081	FALSE
8de8c318baec968219319f50f648af60	6	0.691475346686081	FALSE
67e330de0289f13cf25c1f4ac71dbf19	6	0.691475346686081	FALSE
9729d58a6a7dd9ddc2d0977cc7bb68cf	6	0.691475346686081	FALSE
2bbafcda6fea1b9cbbc8610409b06bf8	6	0.691475346686081	FALSE
64485352a43d56f50f1b3a5f3167b0a4	6	0.691475346686081	FALSE
1509e5bbabd9d1c71b407a0ac218e46b	6	0.691475346686081	FALSE
a5dcc6de4c99de98137e828069413f13	6	0.691475346686081	FALSE
04e24a19d69d50187392f3b293672907	6	0.691475346686081	FALSE
9da052e6c59fcdfcf34e1afb8beb933f	6	0.691475346686081	FALSE
abbdde2546c73e95a40fa010995d2818	6	0.691475346686081	FALSE
29cfe68fe8b380a7be28a396d32cb6ab	6	0.691475346686081	FALSE
725fdb050bbeefec541866c137e9c50a	6	0.691475346686081	FALSE
eb2594d058017aaba416b552608fa95f	6	0.691475346686081	FALSE
8115bfdc03a0ef73ed285dc9716fc0f5	6	0.691475346686081	FALSE
b62deda5ed36b8c96b0acc4011f0001a	6	0.691475346686081	FALSE
9f6e744b36e913bb4649329926fbfee2	6	0.691475346686081	FALSE
96714a6842a401aa4fecabff7a334c5f	6	0.691475346686081	FALSE
2a7d7d28b641f4285a458ca5cfacd950	6	0.691475346686081	FALSE
6cc51fe5dd109eb63633c531f2ffb660	6	0.691475346686081	FALSE
3a228aeb41029dad281ee5d1ad796636	6	0.691475346686081	FALSE
511bf33ffff1bc6d33e8b7024543008d	6	0.691475346686081	FALSE
8839f92e94f7eaa6b15070cdd3a35932	6	0.691475346686081	FALSE
d04db31eb1abae895d806b53ef3e6d33	6	0.691475346686081	FALSE
f018335ed27f4ebb1191e4b07133ba72	6	0.691475346686081	FALSE
8210bded00785df70ea9736d5be8363a	38	0.694321371129945	FALSE
e73d7f3f0353d7c592d2e13108551f31	23	0.69531093863704	FALSE
d881f447ded933c782b33f0ed029eb81	24	0.713444141883855	FALSE
6df3a5390512a230ba46b8f69c083579	24	0.713444141883855	FALSE
78bade5ebbe4174d8be9924c6185e63f	7	0.715499807394952	FALSE
000653cdb81bc81c8296b722a5cfaa0a	7	0.715499807394952	FALSE
849d52ff327105ded96e282fe1bd3b62	7	0.715499807394952	FALSE
35ff75125aac19c43b821d316def91a6	7	0.715499807394952	FALSE

f6abd5e319f04daeaf391a2e5654004f	7	0.715499807394952	FALSE
30dbe645e8a27035b82f3e2724544d5e	7	0.715499807394952	FALSE
6e5b0b6f7e2bb31c463b644e85bde992	7	0.715499807394952	FALSE
6861dd72bd088689e5dfef66bf7e4135	7	0.715499807394952	FALSE
9c2ebf8f2921e2d7bb2e2270607342fa	7	0.715499807394952	FALSE
673173eec4db0acd7101cf10a16b6866	7	0.715499807394952	FALSE
18786531e995f35bfafe6938d96ec702	7	0.715499807394952	FALSE
af6374578fd7c52c9e70103c558b23ad	7	0.715499807394952	FALSE
5606b84bea115e968c56e015ba80be07	7	0.715499807394952	FALSE
6047e1cd01fa98962f888d9f00cbfa6e	7	0.715499807394952	FALSE
d1de072c491257d797a532babbf5764a	7	0.715499807394952	FALSE
15329d5033e2beb83d980271241d8308	7	0.715499807394952	FALSE
d1cfb51391363f63a2a4dae00098c508	7	0.715499807394952	FALSE
9f7c2bd6ef46996c8c194978d581c879	7	0.715499807394952	FALSE
5ce34ff294c4eb73dddb425696c47c60	7	0.715499807394952	FALSE
4cad7317878eea3b34ff87a0aae89c58	7	0.715499807394952	FALSE
75dcf4386d37d4fc0f8d2e6b17ecab8a	7	0.715499807394952	FALSE
1579f92d06c86388b8afd5ff14a3b211	7	0.715499807394952	FALSE
d7f26dea2af5078b6b71563ab7cd6eaa	7	0.715499807394952	FALSE
f9f6d2c29406a1846e6da618176b1e21	7	0.715499807394952	FALSE
cd46e01b7ade82713425c980e460e5ad	7	0.715499807394952	FALSE
426ca56efb6816b00bdf050ab01d0d0f	7	0.715499807394952	FALSE
24216962fb504c3ce0264bf24d1f9cb5	7	0.715499807394952	FALSE
84d7876514c79823177f54c6bad6c2b9	7	0.715499807394952	FALSE
bb7baf63968d36cf1bfbd5fcd580ed70	7	0.715499807394952	FALSE
8b85eaaafd6c5e4b10a90a4a32790e4b	7	0.715499807394952	FALSE
5d9b474ea44d8ca1f55007f87e53a13c	7	0.715499807394952	FALSE
a46eeca1b1204fe087c1f1f5b2e7cf55	7	0.715499807394952	FALSE
9b2e53b19d862d6826fbebe3d423e618	7	0.715499807394952	FALSE
8181046c6027b76fecba11accf9efe8c	7	0.715499807394952	FALSE
e95ef791d06b1d2f990345ee53ca21b8	7	0.715499807394952	FALSE
d3a26f6c315d1c86b14b88e81af8e3fc	7	0.715499807394952	FALSE
5c6edb849229155a7f835f12505003fd	7	0.715499807394952	FALSE
93b6acf12ab28286f735e299fdcf7e3e	7	0.715499807394952	FALSE
985c885e396f1e7ea883ebe31b3e3e66	7	0.715499807394952	FALSE
09595e80206d35b965f4cce02b2f48c3	7	0.715499807394952	FALSE
30658a6c43516aafecc4b553b81e6b8d	7	0.715499807394952	FALSE
4ec764dc072f2f639a74752149d1b9ef	7	0.715499807394952	FALSE
b6c6e727b8a2b38f3fe4f236aab51182	7	0.715499807394952	FALSE
d2851be2899896813406335f097e0766	7	0.715499807394952	FALSE
3b8a7748b36653caf4a339c462aeace7	7	0.715499807394952	FALSE
c219cba5ccd765a396f14d20423f4f5d	7	0.715499807394952	FALSE
b3c39c59af5fea7457921f190fddf246	7	0.715499807394952	FALSE
fddb21906df27ed56154006167998223	7	0.715499807394952	FALSE
2060cd013f6f8e1fdfda2e4fb5f9105c	7	0.715499807394952	FALSE
9068298bbfe44765abf5d4b36326b8f3	7	0.715499807394952	FALSE
f07c42e5c7ba021de7b60091ed440e76	7	0.715499807394952	FALSE
09c3e7640cd22403cb1b069579db6645	7	0.715499807394952	FALSE
cd615ef0610acbcbf4bafa1586ad08ee	7	0.715499807394952	FALSE
e0287daf63905b8cf23ad925f12009b2	7	0.715499807394952	FALSE
6dd8a4fb3215ac145571b0079405a312	7	0.715499807394952	FALSE
a116c09afca2ad940b8906ca4c225822	7	0.715499807394952	FALSE
8d8bd5de425b804441258f27801cd9f6	7	0.715499807394952	FALSE
75c37c29a6bc563618b656d63a21976b	7	0.715499807394952	FALSE
f40ca23c1724abd681edb4205954c756	7	0.715499807394952	FALSE
a8b203461e5cfead0462ac1dcfee07c0	7	0.715499807394952	FALSE
1c132342410d69d37d82a2aed3bfbb05	7	0.715499807394952	FALSE
dc41358fd306fc1053d80935c0ddf0b7	7	0.715499807394952	FALSE
073ce754d4fdcb4e4828e63e4a6387fe	7	0.715499807394952	FALSE
9bfc9ecb4864e67cd7d432a627d7fe11	7	0.715499807394952	FALSE
a84ac8fc73810467a41d4b54da068bf0	7	0.715499807394952	FALSE
169fb216a12f673fcdd8cdcb5de35a82	7	0.715499807394952	FALSE
00d9ad4346446bcd2b34269f283f39ff	7	0.715499807394952	FALSE
ee52da5d4bb76047ccf285398cad8f73	7	0.715499807394952	FALSE
978ba7cb1df2117169afdc062d0dbe46	7	0.715499807394952	FALSE
71c9b7fd6b750486bdf5c59543b98a6e	7	0.715499807394952	FALSE
f4d5b9520fb5e0c48fd3afb1fc9a5047	7	0.715499807394952	FALSE
c65b7d39ebce1feb1d58e27aee1bca47	7	0.715499807394952	FALSE
9c439916915fd3dba65264406eb263ba	7	0.715499807394952	FALSE
a91695ef6eb933fc1a78804ad848ca05	7	0.715499807394952	FALSE
5c046402b9d4eda1925b61e367712fbe	7	0.715499807394952	FALSE
a4591400fc7d7ddf8843573260636cca	7	0.715499807394952	FALSE
1aa75dad1dee675701b42a805fb0d42b	7	0.715499807394952	FALSE
a0999abb73d84e91d5134d80528ef229	7	0.715499807394952	FALSE
4d4c903ae6df84e7f79fbe3cecf19268	7	0.715499807394952	FALSE
d0b698c7298bf04110a6d2f220879bfb	431	0.725498775526386	FALSE
be895bf9176954ab8f55e2c2e1d4e047	25	0.730657519292985	FALSE
2ed220cab9db0862029af74d35ffbaf4	25	0.730657519292985	FALSE
d8cb744c811fa780dd595cf6df39bc60	25	0.730657519292985	FALSE
1357277b4f47cd8732a2707eca12fcdf	8	0.737699001068447	FALSE
170d6813dfbe0ce95dc78d234a5b7155	8	0.737699001068447	FALSE
bd6e442e02ccd1c8d74c8632a93d3648	8	0.737699001068447	FALSE

518b140a9a79e87da975636f1e42f403	8	0.737699001068447	FALSE
e0de3364d91c93c955f117c62e66a9d6	8	0.737699001068447	FALSE
769de0c67929c3c1a4007c54642dfe34	8	0.737699001068447	FALSE
b5e089a90fc5455638f85de23bb20993	8	0.737699001068447	FALSE
80098ec13d5319e6024df222a32d16bd	8	0.737699001068447	FALSE
49c5631b22e3c8af1bcf6caf37d3ff7d	8	0.737699001068447	FALSE
62169a1df5dd845f1a7de084c2219327	8	0.737699001068447	FALSE
42322165cfa8b7b298ef6e3516d0f4a3	8	0.737699001068447	FALSE
97a36b566a19741b7e7ce108e99a678a	8	0.737699001068447	FALSE
11b24486ce2911727bd082a6d0bd868b	8	0.737699001068447	FALSE
6d8da19cf86ecb2c26f21b692f794071	8	0.737699001068447	FALSE
0ca19cdac2fb9198a038aff1281a5e10	8	0.737699001068447	FALSE
18a5046a2252d72a8f9a425127ee63c4	8	0.737699001068447	FALSE
b161a9890947c468aade8a7f27ccc163	8	0.737699001068447	FALSE
a8b23d869d9990033ef96272f0fcf119	8	0.737699001068447	FALSE
6de665187bcc484ed4efad4465646a05	8	0.737699001068447	FALSE
69eb16a95fa594304be5c161c6b15c9e	8	0.737699001068447	FALSE
f14a4043d5ce3e764003e5fce86495e0	8	0.737699001068447	FALSE
daaabb96173b74ef79fd1aef40e9457b	8	0.737699001068447	FALSE
3016b2fab82ed08bfb5585b175919df6	8	0.737699001068447	FALSE
d2ad8ffb9c564a7a919a6d2c2ea25bb4	8	0.737699001068447	FALSE
1b6acfec1f1d9d018036a1e275703706	8	0.737699001068447	FALSE
67fd3a3f3f8db748efbe268136dfbded	8	0.737699001068447	FALSE
4ce4c3a69273ce1cc8fd219da9b381d4	8	0.737699001068447	FALSE
11c23f6cbbdfd03bd8e6224a9071fb43	8	0.737699001068447	FALSE
f40c0d4d9e06093a5ada229490ba0568	8	0.737699001068447	FALSE
9ee2b7f0b9e9c362f67a773511bc0f8f	8	0.737699001068447	FALSE
6232643e6f00d30f14f28555f4d3aeb3	8	0.737699001068447	FALSE
bd51e684722285320e57f327eca15b6b	8	0.737699001068447	FALSE
0789caf27bf5974dcea4d8031951972a	8	0.737699001068447	FALSE
6d113989510223e4c6dbd981301f7212	8	0.737699001068447	FALSE
0522bfa94eba6ceddf15a808b6ae96b7	8	0.737699001068447	FALSE
ae12d30bf4100c4d96adc3ca43677698	8	0.737699001068447	FALSE
233979536096fdadbd5a84680c813451	8	0.737699001068447	FALSE
24df451d973741ec25daf4a0faabb381	8	0.737699001068447	FALSE
a31bd41aba32e4789d6da6371e8a0351	8	0.737699001068447	FALSE
4cf9490af23af672d19005d40819f32a	8	0.737699001068447	FALSE
d9681b85947e3afdfe94497149ef007f	8	0.737699001068447	FALSE
8817c9e2e994bf779d0d9871bfaab685	8	0.737699001068447	FALSE
fc2be12ee7778a775c5e96a7e119d6d4	8	0.737699001068447	FALSE
47601118850e3bfedf4909d626f8ac75	8	0.737699001068447	FALSE
5ee7c51bcfa27a4b318bfb90a7fb84c5	8	0.737699001068447	FALSE
5a300b9a353ac9001a36a58e3306a300	8	0.737699001068447	FALSE
e6552817f250085328087b23ab0dc1a9	8	0.737699001068447	FALSE
085bc0620ad4765efb14c77f0959dfef	8	0.737699001068447	FALSE
f30c82900b5992c0322e1142908bbe21	8	0.737699001068447	FALSE
1c12865114a7479a355a5314a0962ff4	8	0.737699001068447	FALSE
5608c3e6c9de9ceb79610e7786bd0ac4	455	0.73908199478054	FALSE
b39c338f5e964b6cb87e07f10badc6c4	26	0.746982706665468	FALSE
749e45bde9313270898b93b8c4becd90	26	0.746982706665468	FALSE
e33b20a92e8323c00165fd246aa9a5ce	26	0.746982706665468	FALSE
e9c0e61a0dcfb70f290be826a755c503	9	0.758208132672148	FALSE
b36996030f130f5113ee26664007dc10	9	0.758208132672148	FALSE
23a6b01030772ac2e83ffc6a9ec32215	9	0.758208132672148	FALSE
3e039130d1c5ce6ee3bf389b3e16a6c7	9	0.758208132672148	FALSE
3e78c2d4071900023caf060ad6e4aea3	9	0.758208132672148	FALSE
c3a4eb91dcbf4532380498b716445ae3	9	0.758208132672148	FALSE
680be3278c6b80b1b8d60bbfca191947	9	0.758208132672148	FALSE
dbd8c548553b3b93df3ac057d7d2cb56	9	0.758208132672148	FALSE
eb872b492619292ca3b24e42f436f99c	9	0.758208132672148	FALSE
fb8f2032bbe5e226156ce9778d2509af	9	0.758208132672148	FALSE
0c1c7ad5d0260ba6e3bdcc9590c3c2c8	9	0.758208132672148	FALSE
5322c1b4520bfe8d265dfc591df5a64d	9	0.758208132672148	FALSE
c3b153bdcbcbc594c78f50e103ff2b2d	9	0.758208132672148	FALSE
ad73f4ab37e8e5f7ab14dee5fdf5c5ae	9	0.758208132672148	FALSE
b2c3bb2ac840eba4aa8a9cd75f8bc4d6	9	0.758208132672148	FALSE
01080e9f6920e834dd4353e0006970be	9	0.758208132672148	FALSE
0b974216c890cb7d7f1b671e54adc25d	9	0.758208132672148	FALSE
b34dc93f446851e20b7664594eefea4a	9	0.758208132672148	FALSE
9422fdd782051bd355702c8bc5c811c7	9	0.758208132672148	FALSE
4ae9adef4e7cf64f39d31521ce36fe69	9	0.758208132672148	FALSE
6fc9ce36185f7f68f3a3e677c395f7fd	9	0.758208132672148	FALSE
dd1b7f0afcfca1df0f341790f1a2c1e1	9	0.758208132672148	FALSE
cc89995866a6e6077eea2beb57f8a512	9	0.758208132672148	FALSE
bf62225734b915ae3e034aaf5cab2fc2	9	0.758208132672148	FALSE
a51bfff8dc3d75cc03636e70bbfaf47b	9	0.758208132672148	FALSE
5281eb7b2a57c232332fddd223fe88f5	9	0.758208132672148	FALSE
0e7017c0ccd789c11023b2baaa6aab3b	9	0.758208132672148	FALSE
35bbc323229797ab3654d3eb5d1dcbb7	9	0.758208132672148	FALSE
50b02ed56e5bd7071bd7b387464838b7	9	0.758208132672148	FALSE
145042b121fb19b759c9a71cfd6c3b11	9	0.758208132672148	FALSE
070502fc07b0ab293ac6d73549d3b974	9	0.758208132672148	FALSE

2d88b9122b9b887f026e2147867bc8df	9	0.758208132672148	FALSE
8a6ff32f51d7c2b5feb3a3c4507ddbcd	9	0.758208132672148	FALSE
f33382394a5a2e3263ec1b9462ea0b56	9	0.758208132672148	FALSE
393d85b4c2886b63cdb62e812d38b2af	9	0.758208132672148	FALSE
53d442e1e344ed5313bcc6bbd17496e2	9	0.758208132672148	FALSE
1e5faad8ce74f2172060dba245f52f96	9	0.758208132672148	FALSE
b944e446e87fa7a72fe2cccb95deee14	135	0.762399939224698	FALSE
31fafc31f41c40a6477d2bc148624981	27	0.762451888237852	FALSE
62ef06b414289af582c84fd276bdf945	27	0.762451888237852	FALSE
53be6d7e62277b36a2809a2301b96b9a	43	0.764801684642535	FALSE
23443f6140da99fd12fc5b0c4720a271	28	0.777097544572831	FALSE
da6a820d6236082bbee9da8395ecc162	28	0.777097544572831	FALSE
6f2414f3216c719e8f1a44de9f625c94	10	0.777152650112269	FALSE
5e4049b68851f2ee269dcac5f0f90322	10	0.777152650112269	FALSE
09b48cd4c1f3fc619ba1f437dae36dcb	10	0.777152650112269	FALSE
11e23ea5f65f90c78651bcbe86b74191	10	0.777152650112269	FALSE
4c457a70243c649760a1811351afeb0c	10	0.777152650112269	FALSE
257b9d2aab382808563d7f42d38d6964	10	0.777152650112269	FALSE
75fab318c01fbc0d769ab98f3d328f12	10	0.777152650112269	FALSE
f91246e65ef5f4a6eb9ffc444cba10f4	10	0.777152650112269	FALSE
0d0042373110580057296869406719fa	10	0.777152650112269	FALSE
38347f000c351f6a8b58265d41aaa725	10	0.777152650112269	FALSE
91101303b2fa23ed605482c99f27db6b	10	0.777152650112269	FALSE
c8c110fa739e613f951fae4412d6f9f1	10	0.777152650112269	FALSE
126c4c4926f559db383ec0b8320bfac2	10	0.777152650112269	FALSE
e683f1888d6f1ab46f404be9a98d7a3e	10	0.777152650112269	FALSE
fcda020e2366b6eb9a4f922739f4e6ab	10	0.777152650112269	FALSE
42813a7ac2260688d1e5def012a3bf78	10	0.777152650112269	FALSE
bd51a40a24ebd30ef796a7d484fbcd55	10	0.777152650112269	FALSE
dccb9df56eb71cad4bff7ed8431a7552	10	0.777152650112269	FALSE
f0f33d61309d8999591d129526908ddd	10	0.777152650112269	FALSE
33cdd6119240b68f35d70c12fc0aafa6	10	0.777152650112269	FALSE
32480562e8e85d61074673393aa0ec7b	10	0.777152650112269	FALSE
47addf6684fda1d186def383b96fee3c	10	0.777152650112269	FALSE
74d46334ea0cf84cc845e9b24cacc55a	10	0.777152650112269	FALSE
c37fa601378354e8583bb4334eb4e041	10	0.777152650112269	FALSE
addf1e3d60f6910392223e40dcfa898b	10	0.777152650112269	FALSE
106c1bcf09642488d48e631dc007f515	10	0.777152650112269	FALSE
4c476bff9c90e545fcaf52abf2433741	10	0.777152650112269	FALSE
627857f1adf233e328b08b9f4ac9b055	10	0.777152650112269	FALSE
6f5da0517a69410b3b7702e321396052	10	0.777152650112269	FALSE
7fba832c4a81a2dc868d445e56d77ec9	10	0.777152650112269	FALSE
73e59f5f5a61052a6e7255dd9a08aeca	10	0.777152650112269	FALSE
7a21c9fea813dd4d7670e06c68be9aa7	10	0.777152650112269	FALSE
70b3e46a9f56d2957a5da8bbcc4108cc	10	0.777152650112269	FALSE
ad796ee030e064cce08108c72a91eee6	10	0.777152650112269	FALSE
1b156a3626c51ee68eafec90c33e0734	95	0.780667151943677	FALSE
b9d2c93750a12ff7981a77e82f6a1547	29	0.790952233328766	FALSE
4224ae75b61b4c9687df8ff56db506ee	29	0.790952233328766	FALSE
d63133c757e4c822d016db63aaa83f83	11	0.794648929648909	FALSE
3044fec0f8b0b1e86e093d156925713c	11	0.794648929648909	FALSE
d960f6eb68bf555c7af553d60d44c613	11	0.794648929648909	FALSE
8a3bcb66a63e718322795fb3cf5786ba	11	0.794648929648909	FALSE
f52e34a07ff2e814f50950ea181cb271	11	0.794648929648909	FALSE
0c97cf0c7f79074bda3693bd7391dfd4	11	0.794648929648909	FALSE
1e7e1e01adb7640a6a7d607cb61de9ea	11	0.794648929648909	FALSE
6728d5bea5db1509d2bc27d229d9065a	11	0.794648929648909	FALSE
c720ca5d5668c68cf397a7fc3dd7fc92	11	0.794648929648909	FALSE
7581960d6d42dd79720ae6185de9891a	11	0.794648929648909	FALSE
fd7410edb0e0122ad8c41680a0c8cf7f	11	0.794648929648909	FALSE
eb30fe79ff2790662309c17e86b78943	11	0.794648929648909	FALSE
d88a6c6c55c19b1066649b6d8e929f74	11	0.794648929648909	FALSE
7dd5364869a72d25f903f2a7fdd10feb	11	0.794648929648909	FALSE
1a8d886228576ed91c68865e528df502	11	0.794648929648909	FALSE
7e910a4671368a87833508c5b7e64b5c	11	0.794648929648909	FALSE
fe93a26042d60e3601d4b58c7ffc3a80	11	0.794648929648909	FALSE
36fa43c25ed6c2d07c29e03e2894c586	11	0.794648929648909	FALSE
2f6051215455021cba7fae075f514065	11	0.794648929648909	FALSE
88ad1024d096672da0ec59be76064479	11	0.794648929648909	FALSE
c2815e98bbc9a5c26391b06e88536412	11	0.794648929648909	FALSE
34572a2307da886cbce2b191be5f213a	11	0.794648929648909	FALSE
2f36633f10f651dce4741ab9073fbe57	11	0.794648929648909	FALSE
859cd1dcd7e3525daa1e111756201de7	11	0.794648929648909	FALSE
72f332a962517e2081df0ebac4ce0e1a	11	0.794648929648909	FALSE
7aa55291042b0dc85bf66204aeec5f3f	11	0.794648929648909	FALSE
3fb2f665e9e58a2e7ad1fdd1ee77a271	11	0.794648929648909	FALSE
9a99c0b7962e2d34c24e789e2581217f	11	0.794648929648909	FALSE
3ec4481028596ce277448c7cb5385a74	207	0.796503292277668	FALSE
3da914834c78256cc61d1c1c470e4552	30	0.804048399611448	FALSE
045bb03f6292d0b36cd77c9e6c5c4020	30	0.804048399611448	FALSE
196a053b371610ff9ee00b66df0b4d35	12	0.810804914479843	FALSE
cc351e8607640aefef66b8b1b35fb44c	12	0.810804914479843	FALSE

60f0a102e1b3118286adc774264b5b7d	12	0.810804914479843	FALSE
88b3d853bf0ae026c108316bdbf7516d	12	0.810804914479843	FALSE
88f8c0389e4c0872fbee3232ebc0eb5d	12	0.810804914479843	FALSE
c2c99e10b5956b9c6b87131faf3a9467	12	0.810804914479843	FALSE
1590cb24d34f240f939466c1bca211e1	12	0.810804914479843	FALSE
3678d89e46e36d2dc9cdd1a84955bdb4	12	0.810804914479843	FALSE
a6ac3b4cd416b6f921f702db706730f4	12	0.810804914479843	FALSE
4d350a5bdf32548e11848a818277b8c2	12	0.810804914479843	FALSE
17473586f4b889c05139cc72c2e8062e	12	0.810804914479843	FALSE
db4c8dc0512e585410924ee31218577b	12	0.810804914479843	FALSE
e556ee5150ea74092458189357186622	12	0.810804914479843	FALSE
305ab46bc50af8c9b04a52005224274f	12	0.810804914479843	FALSE
6165cf874bd70cc64dd43f05eefe2264	12	0.810804914479843	FALSE
00a7b0037094adc4a8f638508824284b	12	0.810804914479843	FALSE
958362b80eb527e5d99cfc14727a45a8	12	0.810804914479843	FALSE
32891522d68b23b9fbbf71520146d04d	12	0.810804914479843	FALSE
b7dafc10c238527a63f20df8257221eb	12	0.810804914479843	FALSE
3be4093633164903309652a5ee96300f	12	0.810804914479843	FALSE
0ac81f5b1143565c25fdfb8deb4206e8	12	0.810804914479843	FALSE
2d30c2c53fd94f63d88b136d45aca3b0	12	0.810804914479843	FALSE
0251c873b9e6e76973408b336adeb8b2	12	0.810804914479843	FALSE
43bafcbff59dcf1ac8758da64ef9b5bc	31	0.816418212900771	FALSE
8d5e9411bb401bbf42175d0fd956d125	31	0.816418212900771	FALSE
3861ee62617a071584eabec1162b5126	31	0.816418212900771	FALSE
1b29cef84de6cbd9cd52eb9dbaa7fe7d	31	0.816418212900771	FALSE
9b9a1ae38e23758a979eed98d5f06dc9	13	0.825720709603839	FALSE
e7de984e21341b13ac754739096343b2	13	0.825720709603839	FALSE
e95ab89ff87282bb7ebfb109bb235db9	13	0.825720709603839	FALSE
7a17d452d41caf71ea4de6188fdff66e	13	0.825720709603839	FALSE
a0f355228c2bc92f7151668f1fc71558	13	0.825720709603839	FALSE
581add769634c1bb41fe0eac84da9423	13	0.825720709603839	FALSE
a6e12567a5a8ff604652fed640545653	13	0.825720709603839	FALSE
047c596149ab5e1e62eb9341525b0276	13	0.825720709603839	FALSE
a8146cf9c7c06aec25a563142d473b10	13	0.825720709603839	FALSE
4b36eec432ef4ffb3464a8a0a9eafee3	13	0.825720709603839	FALSE
5d55c5aaab36a98540bcd71cf23696b1	13	0.825720709603839	FALSE
c606ef358bc6506aca4c4b89cc56e45e	13	0.825720709603839	FALSE
6fdb0a43d61c08d2d4e244d97a48e9f2	13	0.825720709603839	FALSE
91702465b934dd02c76f76cfdabdf946	13	0.825720709603839	FALSE
997eb2712b5cb0cec6f883c8f2fb31b0	13	0.825720709603839	FALSE
92c64b7632c36c15c97decd4597f4f72	13	0.825720709603839	FALSE
475abf5e533ed261100a749ba2b5f8ec	13	0.825720709603839	FALSE
34c94d66644c9e3e641a5040c3113f72	13	0.825720709603839	FALSE
a9266e9cb66778a8c6c548877d9e204b	13	0.825720709603839	FALSE
c3018532c596c385e1f4cd12b2d3fe8d	13	0.825720709603839	FALSE
52dcfdea4e1a3fe11524ecda68df475d	13	0.825720709603839	FALSE
55166f0153519210b5f9ee37f47f518d	13	0.825720709603839	FALSE
4f10e321d38c10ef2b7a144f41da7d91	13	0.825720709603839	FALSE
edb6db65965a970c9eab814177f21796	13	0.825720709603839	FALSE
be6838d85b6c9b7429f1f7f4879c1d28	13	0.825720709603839	FALSE
f3325b6c39cd939584b540aa9894f1d5	13	0.825720709603839	FALSE
e19ce815c8643b851715aba05acf960b	72	0.835326575452095	FALSE
243efcb3cb4808110c65c8176347c6ea	33	0.839105266188764	FALSE
b98f8c87cd3159b66ec0a418919be624	14	0.839489135872143	FALSE
4d02c00f27d2b0369cae8697f8aa74e0	14	0.839489135872143	FALSE
804da4953d5a3c2dec62eceaafd77dbb	14	0.839489135872143	FALSE
e54de8f948e4f1d2a3c9e5a394c5bc37	14	0.839489135872143	FALSE
7c664e537af176112682a9792e06e757	14	0.839489135872143	FALSE
dbb77ef1903ce7b239d6c65a843ad3f3	14	0.839489135872143	FALSE
7d8a38181893e5747a2b6c5a1be1b9d3	14	0.839489135872143	FALSE
d9d7d7b8bf24da8102e83469458682bb	14	0.839489135872143	FALSE
6f3994ea8e29e64aa02df14a2846a8f6	14	0.839489135872143	FALSE
5655eff59411150b46cf6f2d75b35308	14	0.839489135872143	FALSE
f30cd40fb114ab501e51f2f8aa202e14	14	0.839489135872143	FALSE
b5df6b3e09aba5b4c07aaa7690198885	14	0.839489135872143	FALSE
670ab9f3126df45d2280c6d68eb6724d	14	0.839489135872143	FALSE
61e1d16b27c60bdff7b9d4a61877b777	14	0.839489135872143	FALSE
46bd431314d551a4394037b4b0e5f9da	14	0.839489135872143	FALSE
18430608bf7e39c8866f4592dae12694	14	0.839489135872143	FALSE
8f2f800e4a8e5c41bed76dbcb81203b8	14	0.839489135872143	FALSE
d5a541cd845afa61ac26e362a9368819	14	0.839489135872143	FALSE
6b32fa82cd34029494b5351fe148c7f7	146	0.843871452255949	FALSE
ff4ae0f811e8dcb2d76d7d8acc73deb8	132	0.844381485294231	FALSE
23d922f2b3e9315c90bb4189ba7f7f52	34	0.849484318274827	FALSE
621a423823ae9010632f322b6172946f	34	0.849484318274827	FALSE
6b11f2c5e75502f47952b01fc902ce16	15	0.852196245948932	FALSE
aa806b10a642f7016ecf85fa68d73b48	15	0.852196245948932	FALSE
255e84a7816e3f4f5e5c82c5b086195d	15	0.852196245948932	FALSE
ba9601da497863819d4758229b591e4c	15	0.852196245948932	FALSE
2c894c9e3a032a9824774aed6a96603d	15	0.852196245948932	FALSE
8d9f970561fe1a7c174364c656380fe2	15	0.852196245948932	FALSE
2bab1b7d627ef1fb38400f422073d46e	15	0.852196245948932	FALSE

ccd93a6f7c86fa38459587813126e9fe	15	0.852196245948932	FALSE
15b8ef9c8fd1c209fe10fe79b57a36f4	15	0.852196245948932	FALSE
7cf993146775b44a6b8f89833e71508b	15	0.852196245948932	FALSE
8d4b1c33aee9458da55ed0181ca9b8e3	15	0.852196245948932	FALSE
882f7684e960b257722e753254c34447	15	0.852196245948932	FALSE
465a1ce875ea713594915ac415e88ab9	15	0.852196245948932	FALSE
ece50a62168f85fc61385d8adb4c6494	15	0.852196245948932	FALSE
8a185c2d22cabacdf694587e629c5e53	15	0.852196245948932	FALSE
6b952a73de08f9fe604db1f30be44133	15	0.852196245948932	FALSE
7227f56c77a1a528f4eaa76c94e2004d	15	0.852196245948932	FALSE
4eb188c973c9d35a8ee1c68a9adb9a42	15	0.852196245948932	FALSE
a2d516468a24a0b51943203260a1e7fe	52	0.858720990665559	FALSE
ca79aa0ff5cff05327203d05dc798f8f	35	0.859260460867701	FALSE
e85924823aec7bd0bf33576e477e9902	16	0.863921804725426	FALSE
0e5ef2615e277c7143361c56e09c0b6f	16	0.863921804725426	FALSE
f33a119e73a383eef2ba3f02c6a134de	16	0.863921804725426	FALSE
65d47248136b94e72d9aa1fb5392b608	16	0.863921804725426	FALSE
0bf4f18b63b6282536d07bb07e9a993f	16	0.863921804725426	FALSE
82ac56ca828692be621d100e94a3d6ff	16	0.863921804725426	FALSE
d0ee179b720c3f1df4b1b44f35471a75	16	0.863921804725426	FALSE
a29e586ce34cb2edac8c292267d77ad2	16	0.863921804725426	FALSE
b8a62348b7c542b4e7f94bf0ccb486d6	16	0.863921804725426	FALSE
5160cc3e958946a0a4337b33b6533d3d	16	0.863921804725426	FALSE
88f0846ea1250a0029afddf977ba0e5d	16	0.863921804725426	FALSE
97d4e9ea1f067927134c5a34f0d5ad06	16	0.863921804725426	FALSE
a5b07cf8520236c817752cda1c893a9c	16	0.863921804725426	FALSE
368c8aaf10922a92b5d77aa9eb36a7c0	36	0.868462790609597	FALSE
a9a8ba05b0fcdbad9ee1282d3076b726	36	0.868462790609597	FALSE
28b378e38018bbf0b0484af036ef1e5d	54	0.874611180745984	FALSE
c8fb6ebbb3507d5257309f12e5e4f705	17	0.874739736567337	FALSE
fcff23401e2280bde7682f18fa1d1cf7	17	0.874739736567337	FALSE
60cdff71a273a431942ece7f772dcb10	17	0.874739736567337	FALSE
de4efdb3a52a4d915422b3bd60610041	17	0.874739736567337	FALSE
18c8488af7fada9ef923d6452aeb3a9d	17	0.874739736567337	FALSE
df2dacf39bb2269336cd04a5fefdb119	17	0.874739736567337	FALSE
c184aa75193f4e1f2a583a1fa827b3dc	17	0.874739736567337	FALSE
18b30b18da1d03ffe463fc69ef6441c2	17	0.874739736567337	FALSE
02e3c541b67c1f742460bbf565f63071	17	0.874739736567337	FALSE
1469f96bc2b17e99a7988e3fc891607d	17	0.874739736567337	FALSE
56b22d768a1396ed9e442d54a5ea4dbd	37	0.877119570694495	FALSE
a4d01993784ebbe3fec3839448d1f52d	94	0.87993196455902	FALSE
af41b8a7c03317c74926ead0fd355e8f	18	0.884718541620673	FALSE
73858fe4e97c180d5a7545ff94a6a788	18	0.884718541620673	FALSE
7fb82c9bf8a0918e18e5fd25a3064d11	18	0.884718541620673	FALSE
2d95ca79269a5201b9100b5bb7f969c5	18	0.884718541620673	FALSE
c3d3ad1283cfc17a3d466847de0f585c	18	0.884718541620673	FALSE
642e62255f6c842272cd37f56c189bb6	18	0.884718541620673	FALSE
e90425a6fe76084e079af542f4bd2632	18	0.884718541620673	FALSE
6f34eaefa706c2a148d34d4b640db857	18	0.884718541620673	FALSE
2895e2dc704bf22b9ac315e117faef1e	18	0.884718541620673	FALSE
06957346beaf0bfdf292900cf66ebdb6	18	0.884718541620673	FALSE
073718ac989b6696918a078173a59117	18	0.884718541620673	FALSE
43d98b132b922ada4a8b34d08dff7d38	18	0.884718541620673	FALSE
2d6f7915a01751f1ab279a4e17c15b8e	18	0.884718541620673	FALSE
8ac40970733480af91b7d4204a5ee421	18	0.884718541620673	FALSE
43f2b9bb3ec86a9c4b2322f3b1ca9660	18	0.884718541620673	FALSE
24513f3439ddfae0170dad90ebce403b	38	0.885258189454661	FALSE
df45ff114fca650e2150e761e33d947f	56	0.888943713616188	FALSE
e7929c7247681ff65b78376206017180	39	0.892905129428559	FALSE
a48c86bb43aa39dce41597a3ff0a026f	19	0.893921683255998	FALSE
794184e730540cc7ff89c9e06d29a1d3	19	0.893921683255998	FALSE
fe44c3d1e39fa07e328e63b858248557	19	0.893921683255998	FALSE
6c051d317e4e22c0ed7e6a0f1d665337	19	0.893921683255998	FALSE
8603c3db2bb660bfd3b8d633a4bd4c7a	19	0.893921683255998	FALSE
fdf86a1fa9f0935a6512bd850d5997ea	19	0.893921683255998	FALSE
9370d8d53940a56f66653b13014c2652	19	0.893921683255998	FALSE
413bcb036aa08e6c887c47488e63cfd7	19	0.893921683255998	FALSE
b448d68001582a65eae812c8015a15d2	19	0.893921683255998	FALSE
cff0ae11f6b5cd20ab0d14c2cb6f773c	19	0.893921683255998	FALSE
a9d9e22d46fd432964b7e70ceac35fd0	65	0.894119310001397	FALSE
25089d3f7bed37ea7e764b89a1d2f4e0	40	0.900085945646662	FALSE
cfdd70bdbf090e219bf771ede00b81bb	198	0.900672614915809	FALSE
56de38318fb195cc98890d09d1fb9fd7	20	0.902407948595518	FALSE
c3ecf8e6e7e7a856fbd2df2d4ee56b56	20	0.902407948595518	FALSE
5797b86035ec7c8fa28465a07b9805f0	20	0.902407948595518	FALSE
0ddcce08d539797d98bc25c7c02ae7f8	20	0.902407948595518	FALSE
f64861919bd8bf28858e81c625781fc2	20	0.902407948595518	FALSE
46f6ee1a78f2fa98ad1e29c553d32636	20	0.902407948595518	FALSE
1276c14169f9d464cdb5471ea52e902d	20	0.902407948595518	FALSE
ff2bd29ff42e4dc25a31714e0b6c2dca	41	0.906825251993826	FALSE
5f3673305f1d86e0ddbfa1ed980368b7	41	0.906825251993826	FALSE
4119e9aeb272d4ff8f0af3a026865785	59	0.907777302682907	FALSE

8db9ec3c6c2da03db22d64f72b6f0c7d	21	0.910231783940181	FALSE
6ac028dc5e7f0066b35194e99b072aa8	21	0.910231783940181	FALSE
a1bed12a320d811c7025e11b4aa933ec	21	0.910231783940181	FALSE
d0e7ee826a8a40ab80d2bcc61ab571f5	21	0.910231783940181	FALSE
0a1561a33d1b52b9b8846598ed4ca557	21	0.910231783940181	FALSE
8fd632a60760954f8497f1fc8a51f4b3	21	0.910231783940181	FALSE
219122904013b99064a9d2cf96b0106d	21	0.910231783940181	FALSE
f48e415e85476e0bc78c073767ad7196	21	0.910231783940181	FALSE
ad460170fa30ba7e8f74c918c6fbb3b9	21	0.910231783940181	FALSE
eb19b3f753e323f3d3d73a77b517e46e	21	0.910231783940181	FALSE
ee4b74373fc0cece03796e5db2aa3d3a	21	0.910231783940181	FALSE
2cab47f2bc2f1bdaefbd331f3a490414	21	0.910231783940181	FALSE
63355ac2e4dd546d908aa62e078e814b	22	0.917443606794881	FALSE
c324721a2dc34076ead3590c735a6779	22	0.917443606794881	FALSE
af8c2f69e1dece5ad3a2f23731ae5420	22	0.917443606794881	FALSE
aea976fc75991164def44c3dc183bb16	22	0.917443606794881	FALSE
cd9caf8d961e5c6a16348abd971aea04	22	0.917443606794881	FALSE
a5189f77a2cfeab3bc1602ff5c8ac3e9	492	0.923076923076923	FALSE
31f23810ce7087481ef862d15d9fd041	23	0.924090096078344	FALSE
3d535665aaaee9491f84783ece3dd889	23	0.924090096078344	FALSE
03b3b240d243a428811ea34a257d4bd6	23	0.924090096078344	FALSE
5cda16c50b985a14e0ec9910e3726133	23	0.924090096078344	FALSE
ec23b923e536e0a2279f4c973531e122	23	0.924090096078344	FALSE
e952ac57af197a320c22ecec62384f1c	23	0.924090096078344	FALSE
88da24edb3fb401aa506d44503445f2d	23	0.924090096078344	FALSE
2d834dae498cec30f6b9043cf2910a9d	23	0.924090096078344	FALSE
752353b9793ab0e9cd4dab60c7b72860	23	0.924090096078344	FALSE
bb00e9f32a7ad532a6c3ed50dcb3d251	23	0.924090096078344	FALSE
1afda3ce15867b1740804995147a56d1	23	0.924090096078344	FALSE
94202d625126dcb1729abbd7dd7018a9	23	0.924090096078344	FALSE
cfa03a7d8244ddf2a84841f464810ed5	23	0.924090096078344	FALSE
a67f11c0453dc4e295b206418d36b1f6	44	0.92462603707359	FALSE
19b699fcf40dfdeb5ed8ace52f4f2dc8	44	0.92462603707359	FALSE
d1566cbf914a151f452e881fc9cae2e6	88	0.92543879210039	FALSE
df72998479c8b1228c5c3943f047680b	177	0.926350766924639	FALSE
bba4e5e8793fe193ffacb3e7a66c9f29	63	0.928502518965154	FALSE
025f3ec3dafc2f35bc840c5669bccaff	63	0.928502518965154	FALSE
15d1ae7f19a3b5bcf4667544bde11d38	45	0.929826511957183	FALSE
4cf3fce832b0e75aa582596e5159e808	24	0.930214461999836	FALSE
5df57ffc227fabd2e10b3d6652ccc022	24	0.930214461999836	FALSE
1c6fdc27b561532077e6d9e80823a7fa	24	0.930214461999836	FALSE
e1b3da5566b52686c72b1c8b37d79ab9	24	0.930214461999836	FALSE
3db627cc5e513aef2a1a4690e49e095f	24	0.930214461999836	FALSE
54f785f2d739fa4cdf5fac6da3ebfe4b	24	0.930214461999836	FALSE
22be50f25335287f88a3b9c75961865b	24	0.930214461999836	FALSE
1c0b93dba78bead886310532c780520c	24	0.930214461999836	FALSE
4bf24b715c00092ece6c83803f8f80fc	24	0.930214461999836	FALSE
bc880c65f6166950172bc6317df770e3	24	0.930214461999836	FALSE
c652553c5dcf87deed14605ad770aef4	24	0.930214461999836	FALSE
deba196a20f64e12587b394043745e35	24	0.930214461999836	FALSE
70f27b663b51acdefb4c6e059612bcc5	24	0.930214461999836	FALSE
f248f33104991f8f21e7cd8053710b68	73	0.930864893815069	FALSE
28bd824140d5c67d97ab2272d1014303	74	0.934524190979015	FALSE
0e22b2e49a15e74e09bf1c151ed0f16a	46	0.934694395790298	FALSE
f8becf0c50c828245f68b0f69562ad0b	25	0.935856696987084	FALSE
e3e402965331906831146281f74a361c	25	0.935856696987084	FALSE
96716aeeb18233697ce5131e6a0e778a	25	0.935856696987084	FALSE
077e11092881c0e616329dbdb02051ea	25	0.935856696987084	FALSE
f6ecf45f639b721f6645c142b25b91b9	47	0.939248703899328	FALSE
5ee5bf5ebc1547aea9d338e88b0b7c3b	47	0.939248703899328	FALSE
642567877ebcb1fc9c9b26a7ea85fbc3	26	0.941053808958279	FALSE
7acfbffd36ba9fa46063bf2688c97afa	26	0.941053808958279	FALSE
bae9e2ba2c9f33c59e2c4d6127de0a3a	26	0.941053808958279	FALSE
e48e37a0467ce3d2ff2c3a0f1167497b	26	0.941053808958279	FALSE
2c22a0fda496e652c971a6cfec88ec26	26	0.941053808958279	FALSE
8b1f185c948b07011f747c2da9878f91	125	0.945011945405258	FALSE
75d434e8cbac96a34057771b55101151	27	0.945840038145428	FALSE
16458c9597c205072815592a80ecd7c5	27	0.945840038145428	FALSE
b3bd84d7babbb68dbd7a1528593265c4	27	0.945840038145428	FALSE
70f04406961b7b38ab74ba06cac3c090	27	0.945840038145428	FALSE
933bef6fc914f6f30ce2edd6cb048102	27	0.945840038145428	FALSE
d4dc3f83e8ada91f63124bd347103ae9	28	0.950247058596121	FALSE
5a4eda6fcdd6ab834dba0111fbe55d95	28	0.950247058596121	FALSE
1a5c35a922fd259929f1379e9fc89fe9	28	0.950247058596121	FALSE
61324ada608d3a8fc4f97057a7acd186	28	0.950247058596121	FALSE
4bb0efcbe14db9a98578c065540b3ce0	28	0.950247058596121	FALSE
d453b2abebc22f909d8e87353f4d03ad	28	0.950247058596121	FALSE
c33766d386623db8e71db4e9d44a2f8b	28	0.950247058596121	FALSE
a1b5c85117491a6fee703b82718600c6	28	0.950247058596121	FALSE
6cd8021d0df25c00c8f825e988b87b40	79	0.950296797263479	FALSE
6091b6f3a98a65dea5b6add6b3b7d887	50	0.951207180480497	FALSE
a73619885909abe32871924ea5d5573d	50	0.951207180480497	FALSE

8e7e470f27ee9dee24fe1063312db221	50	0.951207180480497	FALSE
70aa5a01ed7683fd122c10659d43706b	80	0.952997495063912	FALSE
1a95e5b78913fedee75e1f052399e016	80	0.952997495063912	FALSE
9ba80d636e5561c9a76f1aa0f67b5b4f	128	0.953018111878516	FALSE
dab9247c26155029ce7821e765ebc155	29	0.954304165405879	FALSE
89c4dd7f04661867c45fbdc395b3fcee	29	0.954304165405879	FALSE
480a9d52f57963fd090a9dd94ee44681	51	0.954679968359587	FALSE
07bf7669e42ebc17a676ae314133677b	114	0.956449115914962	FALSE
c7985a28aa4cf9627133974bee02eb07	30	0.958038448663033	FALSE
c4bffd0ab73cc7a0273cee68fcf49e24	30	0.958038448663033	FALSE
e27680d4009f98f30248d823bc17fb8e	481	0.958468877945874	FALSE
e71f2cd748d4be5bf9412164728834c2	65	0.95978801932764	FALSE
8d278be881c77f270f9250ee0f55ff8a	437	0.960667235356931	FALSE
8ba130bd59fb89ff9f115561c5945f96	53	0.960945634967021	FALSE
c88aa34a8ddb9b4f44cd46b7ca1ebdd4	31	0.961474955022526	FALSE
a2d411fff3a418ad531287e0f24eb090	31	0.961474955022526	FALSE
fbdd0ce9feb0e5bc7494354ad9296b92	31	0.961474955022526	FALSE
d684d6466b2c6e9ac91ecc13f105b1c4	31	0.961474955022526	FALSE
c0a5a04e7e5773e91892d4c41ab7e0cb	84	0.962505104355992	FALSE
2d983b4684cc85598f7454c72b58eb26	54	0.963765892214911	FALSE
9a2dc4f076b397af87aa6a5cf4404d41	133	0.96413379708232	FALSE
c5803199ff4c3da81551b3b5223ec5e7	67	0.964214349147749	FALSE
b519b2fa0b4b23dbc16576634c819d16	67	0.964214349147749	FALSE
9bee9198d6fd38b817cf2f3a7b118763	67	0.964214349147749	FALSE
23951d86554a06257abfffe1b4bcdf8f	85	0.964587351118251	FALSE
ac181dd33b4fc009cce071af5f0d21c0	32	0.964636837763658	FALSE
e9fac19935b286ddf2a78075e7017977	32	0.964636837763658	FALSE
3d8c220d0ff87025f87b4955bce98e2b	55	0.966394818614568	FALSE
664ba36a3ef364511b4b5865f662ea58	33	0.967545496129418	FALSE
40569c7ad9644afd194ec2f0c317d157	33	0.967545496129418	FALSE
38c2834f2d440309fa6dd0bdce32ed78	33	0.967545496129418	FALSE
b05bfcc2ed94261623c92866941caae4	33	0.967545496129418	FALSE
8c29c0ac4bed029ebbeb22b3935cc8bb	69	0.968175996315455	FALSE
b28ed22c1484414affbb2110e2cb8dd0	70	0.969997389487643	FALSE
73df01709999f4490d3dcd729cb9665f	88	0.970211815168778	FALSE
502d89eeb4d74d570458302627aa1beb	88	0.970211815168778	FALSE
19b611714457a10b79396b8ee7dfbba5	34	0.970220704691418	FALSE
34e6dd3019805dcef9989972c300b776	34	0.970220704691418	FALSE
6a316c83cc725ab296f381e1977fcc84	34	0.970220704691418	FALSE
e8760ab74d8bf916e7b9e6742d3fb1fd	34	0.970220704691418	FALSE
840600f831582858ed2ae3964571e4e7	34	0.970220704691418	FALSE
05a9bdb05e34d5848bf5144f0e58419b	380	0.971009992641975	FALSE
57d52f46acd5562d4edee17e354f9cc6	57	0.971125753566493	FALSE
d3247c936f8f4735909a8526ebf2f49e	391	0.971435119747249	FALSE
ae4ab154cb983f0fccdc437f469417cd	35	0.972680733434301	FALSE
b2bbfa9e2ea7dfdeb3807096c69b1e9a	35	0.972680733434301	FALSE
fd1c43ccf4c93ee1cd46c142e1485689	35	0.972680733434301	FALSE
8c623c0f9544735a038e1d30d2b51fa2	35	0.972680733434301	FALSE
d4f532a400b5a2e27e030558ec7e3a10	35	0.972680733434301	FALSE
ace63ae9de78bed2e5510a38c85812f1	35	0.972680733434301	FALSE
4c06188bc9c7b91eec9e1fe25da00706	312	0.972895070666662	FALSE
fc94161516cf5edaeb8c984c59c9ab21	73	0.974888026979453	FALSE
7ce80a763e15761f1fe86ef8581f826a	36	0.974942459206624	FALSE
a347935cc17b25761d608e0a04fa70ef	91	0.975001953706185	FALSE
ca461f4b5e8bd616a405daf9f2ae0f20	59	0.975226221953246	FALSE
d6be45afca6371cdd7d012d8ce94ebd8	108	0.97523648938085	FALSE
ba673c958da8e9385196463c6ef87a4a	140	0.97582220885328	FALSE
fa5757ea2e0bd2ada15a5592ae37c723	37	0.977021469141446	FALSE
51700328b249bef1561f6d7115121c77	37	0.977021469141446	FALSE
52f7717d7d4b29e0a22c4e08ed966ea3	37	0.977021469141446	FALSE
48cd0867fbe39c565a7343262fa9585e	37	0.977021469141446	FALSE
e3aa1e69ab25c04321fca9f0996fb994	37	0.977021469141446	FALSE
446d2c96ce6aaa50144d2da771ef8863	37	0.977021469141446	FALSE
e8e69f28dc2939bc04a1690ab27bd4bc	75	0.977718738157169	FALSE
9dfd78e79bba89ffe82035671fd61f9d	61	0.97877442548296	FALSE
60de5a18f62043a523b08ecc4e10a441	61	0.97877442548296	FALSE
0fb527bad6c2916e89a972cf6eaf8a42	279	0.978835312501384	FALSE
48957f322815bd853ff040b697d46043	38	0.978932156608897	FALSE
1320f4cb4f2f04a8f6c7709b994b937b	38	0.978932156608897	FALSE
ad65b63c122ba12caca48384f0f34ddf	38	0.978932156608897	FALSE
28323331d787c7344fc7407d7e26effc	38	0.978932156608897	FALSE
8134209f8d6001f476f316447084e099	77	0.980246285334548	FALSE
f9ff9d5eb1c59d26a9689d1b617eba2b	62	0.980363455186265	FALSE
66e6cf9c166194e84fdcf94637ce3bfe	39	0.980687810224822	FALSE
897b61e5d2dce1bf89fedb73ed0eca90	39	0.980687810224822	FALSE
45a68a9eee3cf83e27f4ea309d57ffc3	39	0.980687810224822	FALSE
3b150d94f8782944249fd0a73999227e	39	0.980687810224822	FALSE
5a8154c58ba9ea7bdadbcff21b3a3e6a	144	0.980868578285112	FALSE
8d16916dcdaa69cee304e39539b62bec	40	0.982300696403848	FALSE
ae6ea074d9cd8ec6896004a16d81702b	40	0.982300696403848	FALSE
0345b451b2a01dbc32e694d5310d02aa	97	0.982522440946451	FALSE
501dd344beeb7c69607fa1ffdf9cdfdf	41	0.983782135911896	FALSE

d70bd64edc0672b74dee70fad8437752	81	0.984512697502304	FALSE
c224c0288f2ed4a3bc2220986cad1932	42	0.985142574842024	FALSE
6d43cac09b0febf5f95abf46b0080739	149	0.985852356254384	FALSE
5f7b1039e8789ade0b68ff96715ebdc1	43	0.986391650408402	FALSE
8cbc77e3bd6a3cacd92289ed7497eb49	43	0.986391650408402	FALSE
7020e0243ea7e1d111d3eb43591f550f	43	0.986391650408402	FALSE
15b888fac2a8be4df2917b6670dad73f	235	0.986912054992386	FALSE
53dc126c6dcdbed4cdd3eecb29bbc2f5	84	0.98712561921398	FALSE
4cd36d8f6046c25842ef887d47e7b023	44	0.987538251926098	FALSE
67acfdff1a08a501e960ccc83a5c62c9	44	0.987538251926098	FALSE
b08ef9b722e924b7d7758709ea999bac	85	0.987900140202004	FALSE
2e94d4dfa19d87774d333b853fcaff60	66	0.988438983428952	FALSE
0339d50219c71731139f9ce51ded15c6	66	0.988438983428952	FALSE
3ac305e8c1b4c9ea90f35d8600f8b8d6	45	0.988590577319005	FALSE
830c3ea3526c42f09986097a09800338	104	0.988632991898301	FALSE
1e8a8c7842c164cb6f6c6029625e902c	67	0.989132559179371	FALSE
f924b59e41bdb872246acf7db5361fc2	67	0.989132559179371	FALSE
d3640519d63b009a839d93bb86a6bb29	87	0.98931962583368	FALSE
6b72125ffd5c3d74a2394b56bb6ea404	105	0.989322858710674	FALSE
8643001f205ade41838a964499dd97b8	169	0.989648763831626	FALSE
e2f82f601e2f4d6f8cc9001faa84d7e2	88	0.989969187801586	FALSE
bd23f3464dbee38f2352549d726766d1	88	0.989969187801586	FALSE
fb9868c77db10fc39a0129cde8a6d9b7	88	0.989969187801586	FALSE
52433c56fb7fbc1f94822c886b18b92f	69	0.990402291856229	FALSE
8dddef875ae1fd71b2f55cee552f56e9	47	0.990442044742412	FALSE
d2b22a6509e29fd8d160f8af18373fb7	47	0.990442044742412	FALSE
b86810a3acc42fe737e424e186df83f3	47	0.990442044742412	FALSE
f3b9cecc06cf0c99e801d491b67de2cc	89	0.990581499379893	FALSE
60efc1e5aaedaa4974a098acf1a85a2b	124	0.990649125722333	FALSE
8f98fb8693ed59c21399d83ce2d10724	156	0.990888861088775	FALSE
62bcf76a05df8d7152251dc1abacc559	70	0.990982774941752	FALSE
a54623313df876441f6bd6ea22292102	48	0.991254577851558	FALSE
4fb4479ec3e40df5abc4b828e8a43e85	48	0.991254577851558	FALSE
dd8f88921af5ba9b0e334e9b08aafe18	48	0.991254577851558	FALSE
8af0ba30611d668e5a3927f85542f627	109	0.991712936225779	FALSE
a86b3fc7197f2a1363443bf2934eff4c	109	0.991712936225779	FALSE
55af53a70bb5d746e81b9ce3713a110f	49	0.991999703505461	FALSE
2448d5a801ab0985c09a7c265d15308d	49	0.991999703505461	FALSE
e27762562e5f097f5dd5d3ddaade6200	110	0.99222751881737	FALSE
5d564d5db4ddb67c61b7fdfbffbe15d3	127	0.992284862432369	FALSE
1ff5d9c73c320602c29d8f6f67907114	73	0.992530200713038	FALSE
4ade57e5bf1dea163c998a806258ee84	50	0.992682874891512	FALSE
b4204990d266b67c13e644d5e2e3be91	50	0.992682874891512	FALSE
dd4411cbe45e5040a3a75c1c10cfa747	74	0.992987246573161	FALSE
c991e7b4a3dee5ea8515b854f6631824	74	0.992987246573161	FALSE
9e821a0ea32dc0a79c624893fa1742aa	145	0.993189977710979	FALSE
b121a17a2f7b359de8888212613f319c	129	0.993225110763305	FALSE
a75fdbd0512a2e0b09b67946674ea28e	51	0.993309115328724	FALSE
3ff22a19150bb5bb3fac504b21dd1e34	51	0.993309115328724	FALSE
1860bcc07a862435fe3fc78797201dad	51	0.993309115328724	FALSE
573610ba002103484c779191832a6c9f	76	0.99382283300343	FALSE
51e441cbdcc80da0656e82293ae160b5	52	0.993883051259895	FALSE
96a4b5621edd6a80e9bd93ec87b70e47	96	0.993981542524009	FALSE
e7610857aa92ccaca28d9abdee16c246	53	0.994408942780537	FALSE
2987f11421aba677b3ed1ba21faf8907	53	0.994408942780537	FALSE
b63b4aff4d8c479995ebbb5c206efcc5	53	0.994408942780537	FALSE
c45aec1dff493b3b3a92b769c3527575	53	0.994408942780537	FALSE
e5698bbc0af657f3e43e3cfbee8f55c7	53	0.994408942780537	FALSE
731043e4dbf3a367cdaae8b91bdddbfe	55	0.995331968584332	FALSE
47eacf9c2676422e360170281752eba7	55	0.995331968584332	FALSE
f033e2deafb6fd1525fc91a4d76a8b1f	100	0.995366986351095	FALSE
5a722836ab0e0124d64413717949ade4	81	0.995518134373802	FALSE
686b1c1752a35be01421dfdacfa244e1	101	0.995663257771153	FALSE
b1164c7556428ca28a0fa47436b70504	56	0.995736035085005	FALSE
d5a1283783f4f7170bdef0cd036ee48f	56	0.995736035085005	FALSE
ff7520f4d531ff12bb529c5403dbce5f	82	0.995799422473728	FALSE
9ad50086ebb6725181556774a235e206	102	0.995941708331355	FALSE
74f7929cfd22b1eae037d866281cfc63	83	0.996063945953789	FALSE
118ec7a179026c54887b0f117b8270b8	83	0.996063945953789	FALSE
a9b2c08281a57e2decf34d0892ba7947	83	0.996063945953789	FALSE
a3d81bc3894304b7c0a6df55297a8d08	57	0.99610596811416	FALSE
37ffc50b19370c0b659f4c3d919f99e8	57	0.99610596811416	FALSE
9dfda2206e7dcc1438c2594ff36f4b94	57	0.99610596811416	FALSE
96bf4520c8e056750f57133a13ed76dd	57	0.99610596811416	FALSE
cacfa81f2bc89116d93fe225248244da	85	0.996546457471862	FALSE
5aa73bdfc0eca9a7c83f64620d8828f4	59	0.996754455673928	FALSE
4f27892b2ff94c7f0d0cf982f27d8541	86	0.996766197016136	FALSE
56fd8c08e9e106325cabe0a193e11914	106	0.996896639309482	FALSE
58238c3d1af33f7a1e7c6769b3870a66	60	0.997037974488619	FALSE
a1c5c150d75a73a1e90ce9724214d0e3	61	0.997297322344454	FALSE
35e06063493e115e43f15cc0d438eab2	61	0.997297322344454	FALSE
53805a3bc578a4a6d9b8dcb1946e6d76	89	0.997348877863528	FALSE

1643fdafc7ede484acb8d727168ab741	189	0.997385663338198	FALSE
e6fc000a27af86cd26973287af4e061c	109	0.997469994209178	FALSE
5276610a51db67a6a227a6a3b486efd0	90	0.997519988992907	FALSE
374a0952cc1222bfe7e3f87423eb96a9	62	0.997534508836165	FALSE
8b4400776aa5b6f0268025915d1bee5a	62	0.997534508836165	FALSE
f4ff94f14b1feb5a48cc311c5c895ea0	62	0.997534508836165	FALSE
a8350956db53b9cb8e376f9406f154e3	218	0.997537442777406	FALSE
9ee696aaabfe93f028faf08377028b06	110	0.997637937380995	FALSE
54e7b2139640ec28fbaf7f0491cad845	91	0.99768064271719	FALSE
a26ea2de067690b7f7ed5e1d1efa4770	63	0.997751380744095	FALSE
cb31828b8f23e5e0d3bec4436df5444c	111	0.997795403298972	FALSE
5323c6dd766d629cc750b0aabb4aaf72	92	0.997831444998761	FALSE
a3b72b215a67e3caf3e3db37446195a4	64	0.997949634878026	FALSE
138a326ebca05adbe3f688c7b3ae46b6	64	0.997949634878026	FALSE
09a077421a0f673d4a4ef0a0e3f7a913	64	0.997949634878026	FALSE
54f3fa8080e96ee8c48b77627ac0ae3a	114	0.998210893905748	FALSE
d77a3cc416991d5d3767214641e5b2ca	95	0.99823031082286	FALSE
2a5837baf8baa50071f3e36a0ff46fe2	236	0.99827897279397	FALSE
0a4009eb300a0f2671383587d6d8401e	115	0.998332246750527	FALSE
1815dac9ba55db9afc80283229a98eb4	115	0.998332246750527	FALSE
dbfde3e3bdbc75dd679758dad4834b73	115	0.998332246750527	FALSE
05b920e10cddf14fb5b0f0b42237d3e3	96	0.998347123119959	FALSE
57c580b06ef43b527dce14437166ba0b	136	0.99869184496278	FALSE
d226235298c561b8476ed3e44d6da337	100	0.998745655854517	FALSE
e9e8170bb8209aa94f8ec9a4718ffe6e	138	0.998870720871609	FALSE
698b4f5204ec0b08253a421ffd6bf57f	102	0.998909146884648	FALSE
fcb7d25194c7a7e800b5f7063a557582	102	0.998909146884648	FALSE
e3beabd067252bb387cfe12787744633	121	0.998913223732208	FALSE
bd2d39a2564b0ecc2f4e153d55d8fb7f	121	0.998913223732208	FALSE
4ad750cce3d83d64cabb192681982484	156	0.998990942515244	FALSE
366511529e7fcd4e72ba41ced9049d7b	140	0.999026645773671	FALSE
bec577c7cb4088fbb5631dc6ca656132	124	0.999126678027944	FALSE
76e476e3f5080af8597b9ea02dea6b0e	160	0.999258361933329	FALSE
5ebafbc67cbf3c6e47caca9b43ce08ef	108	0.999287599053711	FALSE
b5a4fee1a67d3818a8b8e8fbc9e19424	109	0.999337145076013	FALSE
e7ea4dc0277b1a7a2abe593bdc1d0ead	110	0.999383439652355	FALSE
c17057f15de467ed9c2c7a57817aa221	112	0.999467066146307	FALSE
9d63796fb581267f616c12eefa801ec2	115	0.999572774925443	FALSE
2068bc70de0f52d80b3aeaa003321213	151	0.999582373188258	FALSE
4ddd7df6ea0439b9b350e9795d3df725	134	0.999588342208228	FALSE
eb71a8778089c7404c228b317e38fef5	116	0.999603391690688	FALSE
ea587b40e7bc63eadc37e63ec3ac249e	152	0.999614271499125	FALSE
7e62ee3da1211add21aedb397c0807d7	184	0.999626238308179	FALSE
b2ea94f362ee35da22832eb8c68eb9dd	118	0.99965854857485	FALSE
acecad0c65e14937a0c7dbc6cb5f43c3	137	0.999673892453825	FALSE
c395ce96ac93ca3a6c81d07dbf85a94f	255	0.999684196681777	FALSE
e6ba559878b76dfc9c3c35aad453efb6	157	0.999742452486147	FALSE
e12ab73726e1064cf0896d411a38c0d4	140	0.999742581542467	FALSE
00ca181ebe6aba8dc69ca15436732300	140	0.999742581542467	FALSE
d9749f8b3a9efd705cf66b9e6c2a8e6f	140	0.999742581542467	FALSE
db8252bed6b71c5e2c72242fe02ce5b1	122	0.999747971981451	FALSE
cda4e6f933bb3108ea3e92f9db411c00	479	0.999771789839331	FALSE
bae67a3f8b760d01ac5d851c35de517e	142	0.999780578992592	FALSE
b36b52b10a3708b8e5fbaac814ab0931	272	0.999798373786883	FALSE
947a3c66fd46a8560854dd1c0dade2f3	126	0.999815044263348	FALSE
5343298f6ab7d59eb55c114cfec8b1ab	126	0.999815044263348	FALSE
5b4f8b625d8fbb1268863be7dbc4db5d	161	0.999815098254206	FALSE
3b555d2d7a26e295bdbdc64f10220157	147	0.999853885653837	FALSE
fe2f04ee87e40784f5104a15bec2058f	164	0.999856504232813	FALSE
60f2c9280ca556fd607cc51bedbbd8b6	148	0.99986547084978	FALSE
da77cbea962a956691c9df99fc91cccf	131	0.999875427733902	FALSE
61abd8a8fc64fc4ea6aa6bb33c056efd	166	0.999879113464545	FALSE
3cbaa88b2064dc5314799d9201534ec7	133	0.999893933187167	FALSE
fa60ad5919ebdc9b39b18872718a2393	290	0.99989482601865	FALSE
0d36c8f594b37214b4b58f29e1721f83	412	0.999899452336954	FALSE
c0e8688a1ac2878e2e6f62fe2a9304a0	134	0.999902186582871	FALSE
114a25e4cdc99c47c718434af3821ad4	135	0.999909834237843	FALSE
e59dec1a8a7c223eec18dbac3621bfcd	154	0.999918812324862	FALSE
a74d576891e4a308b4e08ab2a713bf5b	216	0.999922674573975	FALSE
7945babb9c10211ec6dc054af97441a7	190	0.999942767697508	FALSE
448df6a475edcccc5b389fe189f85fd5	147	0.999967158466949	FALSE
10036e06b250b7624c5b886014e3b2ec	147	0.999967158466949	FALSE
608e6548b1b4cbb6176c8fce090991a5	440	0.999969856753885	FALSE
b078843bd82f039e57f68ab3a55a39c1	265	0.99997139406525	FALSE
75dfd64719ae4652eac85de6f5bfe498	198	0.999973467632021	FALSE
eb01a38d86dcf9d0cc9d44abaa8152f4	213	0.999974597960593	FALSE
9cc12e6a4537bd55623ad90bf6b7084c	254	0.999976179830259	FALSE
ef606438f599842862bffdb564c7148d	154	0.99998231804312	FALSE
02bab2db0a7782cf48dec72cbac4bb20	172	0.999983924849906	FALSE
67a8bfa400cf64467ae73d78ee9b825c	188	0.999984067773002	FALSE
dfc9a1ff9b06e08d2132da15e61b3919	306	0.999985833424557	FALSE
0cfd5d23461f5a71d9628ac22ce590ac	295	0.99998649106477	FALSE

5285ebad4f31b3e14f431007008acc63	235	0.999989412777277	FALSE
87cae47f30ea5a277a184645ac6c3b29	160	0.999989795870326	FALSE
d64d3900fff68de4ea004f18ba50c364	239	0.999993294835884	FALSE
a9a88c76960dfd5173b31ab28fe6828b	267	0.999995006944357	FALSE
29353ed4af9cbabb15cc3cc85a1f760a	215	0.999995510222954	FALSE
4c60dd7ba506649272fa67651b05a2f7	170	0.999996086406941	FALSE
b756bd4eff15dc5da0d88f9c34f65398	293	0.99999629171413	FALSE
d32274e6eeffddfd841ee35b5ba8ea1f	245	0.999996712798885	FALSE
ae8c85dfff97d56450e41e66c152ce85	271	0.999997027002998	FALSE
883d7985f03c15e9a5868bf9e14107d0	174	0.999997374135758	FALSE
babe77fccd08d91c140f37209e4bb5a5	220	0.999997444468929	FALSE
98679ce00beb048dfa099128eef70f21	175	0.99999762694986	FALSE
ad6f1612a5a7fdb80cf80a0402d48ee1	211	0.999998612465422	FALSE
b125cd368e6d851a7650f0a58795f5eb	181	0.99999872372356	FALSE
7ca9c8834010100a0dd569f0b2b5666b	213	0.999998898059349	FALSE
4cecd2478253cedd0dff65d35d7c434a	290	0.999998904915158	FALSE
a78fa572a60226d8150bfb0fc0fad652	241	0.999998942214219	FALSE
0a93c58cbaf5e764041cab0ad3e79b08	258	0.999999380746795	FALSE
50b47d356c39644171792954d17fce0b	188	0.999999398765722	FALSE
14fb9695d2e95bd663ad9a51b4d06723	276	0.999999726560132	FALSE
b78823261d78f2495896a69a3949e005	310	0.999999728244397	FALSE
773727ee7de31552dcf3f9609c18bdad	211	0.99999975236723	FALSE
28c1c628392df6a901829cfd2359a460	278	0.999999797358187	FALSE
f3a9e69bd296dd5d39ffecc544b36f25	398	0.99999988284644	FALSE
669fa3f5fbd7330211bc2d8d996a9e3e	220	0.999999919550495	FALSE
f5ef8bcbeee4753d8edc0b1d7e0aa499	206	0.999999926051858	FALSE
2081e8a45b69df88f6b3a5c914871247	240	0.999999965205849	FALSE
fb6931fb710455a0e1c19b26af270255	227	0.999999968102121	FALSE
5be8258fc43191236125bcc62765fdd9	293	0.999999982309437	FALSE
8cb3f4e315069c9334d1c069b96e3eac	234	0.999999987948591	FALSE
b8ba8f7a8bade720e87ca59fc4ab7fdb	274	0.999999991073874	FALSE
267d0f9a598522aa3ee4069277ea81bb	250	0.999999991988983	FALSE
6ace15b44dc69780c4bf45ef07ca096d	241	0.999999995678932	FALSE
b704e320a56fc1af1835929a3b71b7e3	255	0.999999996325366	FALSE
8114b1d0274e9e4bb6c91f6af1b8fac8	302	0.999999996590613	FALSE
3568d370fba8e070545cb84b03b7a088	268	0.999999996698289	FALSE
a2daf95d820d43e97bb2f8f61e3baaff	280	0.999999996796675	FALSE
de2a368a837144ad6e7bf64c176b742f	272	0.999999998330345	FALSE
fd75fae7f9721be5ba0e4a3704a1dedf	307	0.999999998724608	FALSE
ef54ef898867d22d2f65ebda83483ba1	331	0.999999999438227	FALSE
74299b73760426772762b92dde7d1131	322	0.999999999534007	FALSE
2eaaf1e130174d468d3b94cff4900e6e	281	0.99999999967167	FALSE
e7142feb387f929ee101779e66677cb9	297	0.999999999874447	FALSE
a2613697e6d6cc02bc530fd192f06bc9	311	0.999999999940768	FALSE
2f93e58b78f2842e83abd5fde37ad276	421	0.999999999980276	FALSE
2545c00c4b7992fda321d40d92a8cab4	336	0.99999999998447	FALSE
e083e2f58987c5f8db5d4dd16ddde91f	364	0.999999999990278	FALSE
5160485a724e51fcd3e4ebe658012aa8	416	0.999999999990541	FALSE
9e1d3dc63682fe2097c4dfa8dec76e9b	344	0.999999999999885	FALSE
24a60c6448e70d9198ad6ba93520958c	477	0.999999999999994	FALSE
28b8399c2b0790f21a60a17d495c620a	386	0.999999999999998	FALSE
520c77820886daeb8cf0d6497cfb1344	311	0.999999999999998	FALSE
5aa8281fbf9be4bfbf7ac852f1bdf8fe	373	0.999999999999999	FALSE
77accce4f7d181b78f7e66aad08abe01	439	1	FALSE
5f92443932d0deb4cf0d196b6e4fe4c0	396	1	FALSE
3f0d06b8c1b5e61b424af5b55ee3496d	411	1	FALSE
e92e9e21eab765c6ada40b76b7df1e0c	417	1	FALSE
523ac9f41cf23bf8c67d6537760b28d5	361	1	FALSE
49fc1baff761bf9d357844d43cd138ed	1	NA	FALSE
91d6bece60976a50b9051eea4bf40ad9	1	NA	FALSE
41916132a0320d7898614ab3368c5217	1	NA	FALSE
8811d5912a35b66ddd56a85f2f84756f	1	NA	FALSE
061cc3fd68a078dfc17c89dd4e7c1d57	1	NA	FALSE
f5b36d223324383df303174975ce46d5	1	NA	FALSE
36bb4e0630608c32e7b2a4b043c399c1	1	NA	FALSE
0984365862e8b81ae110762355cd5356	1	NA	FALSE
59f71f034d6f5f3211ce16437c08a399	1	NA	FALSE
8dcdc18ee9b372e12ad9acf3b560f0de	1	NA	FALSE
f42582c91ba40de887b64d7b2356d6d8	1	NA	FALSE
dbd6a52a5c2d8dd052e383c067109d73	1	NA	FALSE
3540e1ab31453e650aed7b1c16c68373	1	NA	FALSE
ae37473e32867acb33cd941ca2a0bc9f	1	NA	FALSE
6639175de58b5f0ee31f8300c07adc72	1	NA	FALSE
8cf34b55c0736c2a6e94f72fd0aa07e1	1	NA	FALSE
408f662e9702a6252066a482053dfa05	1	NA	FALSE
0556309e565282e3cdc6d22c7a1314ca	1	NA	FALSE
a31397646d05a84f8890b670601c2a88	1	NA	FALSE
c6e6c1884afc6be4ad05add5339cc95e	1	NA	FALSE
1e3ca86e4fa19f2b96529f8a78b6304a	1	NA	FALSE
90a7ad77ba874fb1ad37e15e4defbb91	1	NA	FALSE
6e0436014e7f545bb0c89c6f90bf2c82	1	NA	FALSE
1488392be87091b8779262c95753d489	1	NA	FALSE

71a639b66d4d99791d5c69e649932498	1	NA	FALSE
b90a3f4af43dd1eadaac36033d825b32	1	NA	FALSE
7c085dcaa22450a60d0bcdcff9c02f1d	1	NA	FALSE
7615b39e73064bf612abefe1336a5f5e	1	NA	FALSE
0cf91d09851e5165515a95c48f990b2c	1	NA	FALSE
40ef360e3de9eef2e54cc384c0f442fb	1	NA	FALSE
c9d2e62830d879d1b703ec112804ed4f	1	NA	FALSE
179dfc0f76a5c60ed0db3f36ea4103d5	1	NA	FALSE
59bbb81117691ae8817ac815f73b9945	1	NA	FALSE
27cee38f3d317da09942584a9e611701	1	NA	FALSE
a344d280d5637fa174318df87463e8b2	1	NA	FALSE
1abf91747a161b143a6733473add3d1f	1	NA	FALSE
58172f93459801cf780471559743a1a1	1	NA	FALSE
2dbe816b5bd581af1d9e40b426a3ccc5	1	NA	FALSE
ad213ddb834c49ba5c3fb2c35715ea31	1	NA	FALSE
f4759806bae7e262721beb08f96af6f2	1	NA	FALSE
9f6cc04b4b4b011455a72b5a60460c23	1	NA	FALSE
c3a7e5679db8cc9a7dcb122d21a62abd	1	NA	FALSE
99655509650822e576d12e2203109953	1	NA	FALSE
c4a5fa167be7022d7e8f6a24ea5a814e	1	NA	FALSE
a75568deb408dcc5be74bdf23e1f6dc7	1	NA	FALSE
6ac5bf8ed56c7709a77fc026615798ad	1	NA	FALSE
324760267b2fe263a0c835e9af329c00	1	NA	FALSE
6f5e0038f0b835752a599ff8ec9801ea	1	NA	FALSE
88d21bfdf6bb7dd539b1f97ef3fd3d11	1	NA	FALSE
7148747d2d38f0d895386df0c931dcb4	1	NA	FALSE
fa50250efdc5d41bd6577db321412f03	1	NA	FALSE
8d97a06ddc80deb17af3ed88f954f8e1	1	NA	FALSE
f68c7cd7566c0f1295c0e531c09b5c1f	1	NA	FALSE
8c3070a01c5d1017b27e2dac97270d05	1	NA	FALSE
7425d153990b97e5f0e131d34cad5aa4	1	NA	FALSE
c1d232ebefcae769cfd656b0931cd11a	1	NA	FALSE
7591056a10b3bd4cc9621543330349ef	1	NA	FALSE
20c1ba0382813ccc891453a727f45d2d	1	NA	FALSE
ced6caa0b6c0ffc98a8414b5bdc83112	1	NA	FALSE
3f1c0b87de5bb4df2b8d2e6b36bed0ab	1	NA	FALSE
f6799ce3e138384ebd9119e29709a3f3	1	NA	FALSE
855b88b34ea95ec7b5c3de4ad4c824ee	1	NA	FALSE
30adb700aa2336a724166e014d162e7f	1	NA	FALSE
5cd044dfb5feca3ee72dae76fa6e9ee9	1	NA	FALSE
5e6f632ca87cf43563b74e15539e7785	1	NA	FALSE
bd96e429d73d57cbb9c8ef9bc46b7820	1	NA	FALSE
65e2fe387bfdf0fa880811cceac1d59e	1	NA	FALSE
5a14b8ff1aecdf0489c6820e8e105b88	1	NA	FALSE
ef5a2480e55d717d0c565e287f82a473	1	NA	FALSE
9ae9c5171312ae7ce382a7baa6b81362	1	NA	FALSE
0911bb8210812727927796066a861651	1	NA	FALSE
85a7a27464dba46cd60d893ad0b3d35f	1	NA	FALSE
3114f9c7de279de58fe3dc15e2dd14fb	1	NA	FALSE
b4bee6fb68e88d00ba80c1bc627510c0	1	NA	FALSE
12679b50e6d1202949122b8b0622ade1	1	NA	FALSE
9f2cf0e7035d0f777c21936203005b14	1	NA	FALSE
394c2ec9cae1af76e0b585fedacb13a2	1	NA	FALSE
8bedbd900998bc8be0f4e32027c14b79	1	NA	FALSE
d91b9586180e2dc67db8d50bfc06d00c	1	NA	FALSE
9ae3e5447f15b13586d0d3803b8e68af	1	NA	FALSE
e50354b9c4d97287a20c1e167dfefa3e	1	NA	FALSE
ba76aae4914cd1c68039931188d050fe	1	NA	FALSE
03b679010b1bf8b116785f42667e02e1	1	NA	FALSE
e7eb03a5b2a368b8a6099d242bcacd54	1	NA	FALSE
945f64283b2eba0afa876de55b78ef31	1	NA	FALSE
c686148b8d01f84f7604bc5fcaddbebd	1	NA	FALSE
0407fd8be2c9084dafdf0c2cd3d0befa	1	NA	FALSE
8d2de3aea083cf4f7d1eae80c2af9146	1	NA	FALSE
3e23230e4686b96576316f5e033b7082	1	NA	FALSE
6e7e1fbac8c5e839a9cdfc5fba8f7770	1	NA	FALSE
7b3fa6d9e1062f0b6537b7372012b859	1	NA	FALSE
d19aea26e0ad7c2155fdd287a2ec6b77	1	NA	FALSE
0ca49b13050f305492d115000a1d001d	1	NA	FALSE
2b9cede5145c126ca5a5de9fc895a14b	1	NA	FALSE
1a902f04710149ed9498c3e99a5afd63	1	NA	FALSE
8577af56ac1b94a1aa8b74077b5a5cbc	1	NA	FALSE
bd1a351302ec362f46db2979bada621d	1	NA	FALSE
352144a7596df7857029b20703ae2eda	1	NA	FALSE
5c3c19675e638054dd3325e5ba5677d2	1	NA	FALSE
15d139a08d6c1648ba4c20c9995ab9b2	1	NA	FALSE
2ca106d719b5911c71a6e189459efa66	1	NA	FALSE
845f586ce16f4bd61ab41379809ae1e7	1	NA	FALSE
72b07965cf4f08eff549d0053d0906f1	1	NA	FALSE
1b336d762092361e9f7c04a56548a6a2	1	NA	FALSE
2429b0edc950114d01ddec0d631166cd	1	NA	FALSE
3f839cccd6e9568fcb569b0e4f7d6ee8	1	NA	FALSE

1c0e84ab16636a5d983a7f210354520b	1	NA	FALSE
f7163bb266bce765f0aa145cf7a6c37f	1	NA	FALSE
6fd9843eb698930e30818d77d505ed5c	1	NA	FALSE
94b612176f3b4cea22cda2f67134ebe6	1	NA	FALSE
25ddb3b811c0140bec907e45cf4b2a45	1	NA	FALSE
68f55b94cf23a2b444b7c8929907eb7f	1	NA	FALSE
97f006e854e73edfcecc1415ac23de6d	1	NA	FALSE
8171a8faf564215283a3fccfe23a4026	1	NA	FALSE
3eac2f1f0d1c5a98e4dcfe3bb7c16709	1	NA	FALSE
832fadfb5fe2b9bec572b5788cfb3f73	1	NA	FALSE
7a538dcb40656b6be4c267503c501b0a	1	NA	FALSE
4e4b6a3260a6061be4590ed7fd29a214	1	NA	FALSE
5ed7d64c93da6dfb32f43400d14ca8e3	1	NA	FALSE
3e92de515639a37692e35e261af54e98	1	NA	FALSE
1848ba16ac6e038d4be98fd5d7bfead2	1	NA	FALSE
bb5e5ae0efa9bf009dd646ebcac3bab4	1	NA	FALSE
703968088ed15033df6c62f2530e50b3	1	NA	FALSE
62107a3ffb0f00f6ff881660f970e449	1	NA	FALSE
d3f91dd2f8e03e272362cb77276b159c	1	NA	FALSE
37e1ba9d0783625524befd620d1e3d29	1	NA	FALSE
3d6c6053fcc0b58354ac9dbe2d2f699e	1	NA	FALSE
