# Supplementary material for: Responsiveness to pulmonary rehabilitation in COPD is associated with changes in microbiota
Source: Respir Res. 2023 Jan 25;24:29. doi: 10.1186/s12931-023-02339-z (PMC9875510; doi:10.1186/s12931-023-02339-z)
Supplement: Supplementary file 3 — Additional file 3. Clinical database summarizing the response to PR per patient in each domain (dyspnoea (mBorg), exercise capacity (6MWT) and impact of disease (CAT). [file 12931_2023_2339_MOESM3_ESM.pdf]

	#16s_sampleID			Subject	Monthcat	Group	mBorg	6MWT	CAT
Sample_1	16s_sampl	3R451_FU1	3R451_FU1	subject3R4	M0	Control	Not apply	Not apply	Not apply
Sample_2	16s_sampl	3R452_FU1	3R452_FU1	subject3R4	M0	Control	Not apply	Not apply	Not apply
Sample_3	16s_sampl	3R_455_M	3R_455_M	subject3R4	M0	Control	Not apply	Not apply	Not apply
Sample_4	16s_sampl	508_FU1_p	508_FU1_p	subject508	M0	Control	Not apply	Not apply	Not apply
Sample_5	16s_sampl	509_FU1_p	509_FU1_p	subject509	M0	Control	Not apply	Not apply	Not apply
Sample_6	16s_sampl	519_M1_p	519_M1_p	subject519	M0	Control	Not apply	Not apply	Not apply
Sample_7	16s_sampl	526_FU1_p	526_FU1_p	subject526	M0	Control	Not apply	Not apply	Not apply
Sample_8	16s_sampl	527_FU1_p	527_FU1_p	subject527	M0	Control	Not apply	Not apply	Not apply
Sample_9	16s_sampl	540_M1_L	540_M1_L	subject540	M0	Control	Not apply	Not apply	Not apply
Sample_10	16s_sampl	578_M1_L	578_M1_L	subject578	M0	Control	Not apply	Not apply	Not apply
Sample_11	16s_sampl	583_FU1_p	583_FU1_p	subject583	M0	Control	Not apply	Not apply	Not apply
Sample_12	16s_sampl	649_FU1_p	649_FU1_p	subject649	M0	Control	Not apply	Not apply	Not apply
Sample_13	16s_sampl	663_M1_L	663_M1_L	subject663	M0	Control	Not apply	Not apply	Not apply
Sample_14	16s_sampl	665_M1_p	665_M1_p	subject665	M0	Control	Not apply	Not apply	Not apply
Sample_15	16s_sampl	676_M1_L	676_M1_L	subject676	M0	Control	Not apply	Not apply	Not apply
Sample_16	16s_sampl	678_M1_p	678_M1_p	subject678	M0	Control	Not apply	Not apply	Not apply
Sample_17	16s_sampl	694_M1_p	694_M1_p	subject694	M0	Control	Not apply	Not apply	Not apply
Sample_18	16s_sampl	695_M1_L	695_M1_L	subject695	M0	Control	Not apply	Not apply	Not apply
Sample_19	16s_sampl	794_FU1_p	794_FU1_p	subject794	M0	Control	Not apply	Not apply	Not apply
Sample_20	16s_sampl	795_FU1_p	795_FU1_p	subject795	M0	Control	Not apply	Not apply	Not apply
Sample_21	16s_sampl	801_FU1_p	801_FU1_p	subject801	M0	Control	Not apply	Not apply	Not apply
Sample_22	16s_sampl	807_FU1_p	807_FU1_p	subject807	M0	Control	Not apply	Not apply	Not apply
Sample_23	16s_sampl	809_FU1_p	809_FU1_p	subject809	M0	Control	Not apply	Not apply	Not apply
Sample_24	16s_sampl	830_FU1_p	830_FU1_p	subject830	M0	Control	Not apply	Not apply	Not apply
Sample_25	16s_sampl	833_FU1_p	833_FU1_p	subject833	M0	Control	Not apply	Not apply	Not apply
Sample_26	16s_sampl	837_M1_L	837_M1_L	subject837	M0	Control	Not apply	Not apply	Not apply
Sample_27	16s_sampl	851_FU1_p	851_FU1_p	subject851	M0	Control	Not apply	Not apply	Not apply
Sample_28	16s_sampl	880_M1_L	880_M1_L	subject880	M0	Control	Not apply	Not apply	Not apply
Sample_29	16s_sampl	884_M1_L	884_M1_L	subject884	M0	Control	Not apply	Not apply	Not apply
Sample_30	16s_sampl	885_M1_p	885_M1_p	subject885	M0	Control	Not apply	Not apply	Not apply
Sample_31	16s_sampl	891_M1_L	891_M1_L	subject891	M0	Control	Not apply	Not apply	Not apply
Sample_32	16s_sampl	897_M1_L	897_M1_L	subject897	M0	Control	Not apply	Not apply	Not apply
Sample_33	16s_sampl	907_M1_L	907_M1_L	subject907	M0	Control	Not apply	Not apply	Not apply
Sample_34	16s_sampl	934_M1_L	934_M1_L	subject934	M0	Control	Not apply	Not apply	Not apply
Sample_35	16s_sampl	935_FU1_p	935_FU1_p	subject935	M0	Control	Not apply	Not apply	Not apply
Sample_36	16s_sampl	P101_FU1_	P101_FU1_	subjectP10	M0	Control	Not apply	Not apply	Not apply
Sample_37	16s_sampl	P201_FU1_	P201_FU1_	subjectP20	M0	Control	Not apply	Not apply	Not apply
Sample_38	16s_sampl	P204_FU1_	P204_FU1_	subjectP20	M0	Control	Not apply	Not apply	Not apply
Sample_39	16s_sampl	3R451_FU2	3R451_FU2	subject3R4	M1	Control	Not apply	Not apply	Not apply
Sample_40	16s_sampl	3R452_FU2	3R452_FU2	subject3R4	M1	Control	Not apply	Not apply	Not apply
Sample_41	16s_sampl	3R_455_M	3R_455_M	subject3R4	M1	Control	Not apply	Not apply	Not apply
Sample_42	16s_sampl	508_FU2_p	508_FU2_p	subject508	M1	Control	Not apply	Not apply	Not apply
Sample_43	16s_sampl	509_FU2_p	509_FU2_p	subject509	M1	Control	Not apply	Not apply	Not apply
Sample_44	16s_sampl	519_M2_p	519_M2_p	subject519	M1	Control	Not apply	Not apply	Not apply
Sample_45	16s_sampl	526_FU2_p	526_FU2_p	subject526	M1	Control	Not apply	Not apply	Not apply
Sample_46	16s_sampl	527_FU2_p	527_FU2_p	subject527	M1	Control	Not apply	Not apply	Not apply
Sample_47	16s_sampl	540_M2_L	540_M2_L	subject540	M1	Control	Not apply	Not apply	Not apply
Sample_48	16s_sampl	578_M2_L	578_M2_L	subject578	M1	Control	Not apply	Not apply	Not apply
Sample_49	16s_sampl	583_FU2_p	583_FU2_p	subject583	M1	Control	Not apply	Not apply	Not apply
Sample_50	16s_sampl	649_FU2_p	649_FU2_p	subject649	M1	Control	Not apply	Not apply	Not apply

Sample_51	16s_sampl	663_M2_L	663_M2_L	subject663	M1	Control	Not apply	Not apply	Not apply
Sample_52	16s_sampl	665_M2_p	665_M2_p	subject665	M1	Control	Not apply	Not apply	Not apply
Sample_53	16s_sampl	676_M2_L	676_M2_L	subject676	M1	Control	Not apply	Not apply	Not apply
Sample_54	16s_sampl	678_M2_p	678_M2_p	subject678	M1	Control	Not apply	Not apply	Not apply
Sample_55	16s_sampl	694_M2_p	694_M2_p	subject694	M1	Control	Not apply	Not apply	Not apply
Sample_56	16s_sampl	695_M2_L	695_M2_L	subject695	M1	Control	Not apply	Not apply	Not apply
Sample_57	16s_sampl	794_FU2_	794_FU2_	subject794	M1	Control	Not apply	Not apply	Not apply
Sample_58	16s_sampl	795_FU2_	795_FU2_	subject795	M1	Control	Not apply	Not apply	Not apply
Sample_59	16s_sampl	801_FU2_	801_FU2_	subject801	M1	Control	Not apply	Not apply	Not apply
Sample_60	16s_sampl	807_FU2_	807_FU2_	subject807	M1	Control	Not apply	Not apply	Not apply
Sample_61	16s_sampl	809_FU2_	809_FU2_	subject809	M1	Control	Not apply	Not apply	Not apply
Sample_62	16s_sampl	830_FU2_	830_FU2_	subject830	M1	Control	Not apply	Not apply	Not apply
Sample_63	16s_sampl	833_FU2_	833_FU2_	subject833	M1	Control	Not apply	Not apply	Not apply
Sample_64	16s_sampl	837_M2_L	837_M2_L	subject837	M1	Control	Not apply	Not apply	Not apply
Sample_65	16s_sampl	851_FU2_	851_FU2_	subject851	M1	Control	Not apply	Not apply	Not apply
Sample_66	16s_sampl	880_M2_L	880_M2_L	subject880	M1	Control	Not apply	Not apply	Not apply
Sample_67	16s_sampl	884_M2_L	884_M2_L	subject884	M1	Control	Not apply	Not apply	Not apply
Sample_68	16s_sampl	885_M2_p	885_M2_p	subject885	M1	Control	Not apply	Not apply	Not apply
Sample_69	16s_sampl	891_M2_L	891_M2_L	subject891	M1	Control	Not apply	Not apply	Not apply
Sample_70	16s_sampl	897_M2_L	897_M2_L	subject897	M1	Control	Not apply	Not apply	Not apply
Sample_71	16s_sampl	907_M2_L	907_M2_L	subject907	M1	Control	Not apply	Not apply	Not apply
Sample_72	16s_sampl	934_M2_L	934_M2_L	subject934	M1	Control	Not apply	Not apply	Not apply
Sample_73	16s_sampl	935_FU2_	935_FU2_	subject935	M1	Control	Not apply	Not apply	Not apply
Sample_74	16s_sampl	P101_FU2_	P101_FU2_	subjectP10	M1	Control	Not apply	Not apply	Not apply
Sample_75	16s_sampl	P201_FU2_	P201_FU2_	subjectP20	M1	Control	Not apply	Not apply	Not apply
Sample_76	16s_sampl	P204_FU2_	P204_FU2_	subjectP20	M1	Control	Not apply	Not apply	Not apply
Sample_77	16s_sampl	3R451_FU	3R451_FU	subject3R4	M2	Control	Not apply	Not apply	Not apply
Sample_78	16s_sampl	3R452_FU	3R452_FU	subject3R4	M2	Control	Not apply	Not apply	Not apply
Sample_79	16s_sampl	3R_455_M	3R_455_M	subject3R4	M2	Control	Not apply	Not apply	Not apply
Sample_80	16s_sampl	508_FU3_	508_FU3_	subject508	M2	Control	Not apply	Not apply	Not apply
Sample_81	16s_sampl	509_FU3_	509_FU3_	subject509	M2	Control	Not apply	Not apply	Not apply
Sample_82	16s_sampl	519_M3_p	519_M3_p	subject519	M2	Control	Not apply	Not apply	Not apply
Sample_83	16s_sampl	526_FU3_	526_FU3_	subject526	M2	Control	Not apply	Not apply	Not apply
Sample_84	16s_sampl	527_FU3_	527_FU3_	subject527	M2	Control	Not apply	Not apply	Not apply
Sample_85	16s_sampl	540_M3_L	540_M3_L	subject540	M2	Control	Not apply	Not apply	Not apply
Sample_86	16s_sampl	578_M3_L	578_M3_L	subject578	M2	Control	Not apply	Not apply	Not apply
Sample_87	16s_sampl	583_FU3_	583_FU3_	subject583	M2	Control	Not apply	Not apply	Not apply
Sample_88	16s_sampl	649_FU3_	649_FU3_	subject649	M2	Control	Not apply	Not apply	Not apply
Sample_89	16s_sampl	663_M3_L	663_M3_L	subject663	M2	Control	Not apply	Not apply	Not apply
Sample_90	16s_sampl	665_M3_p	665_M3_p	subject665	M2	Control	Not apply	Not apply	Not apply
Sample_91	16s_sampl	676_M3_L	676_M3_L	subject676	M2	Control	Not apply	Not apply	Not apply
Sample_92	16s_sampl	678_M3_p	678_M3_p	subject678	M2	Control	Not apply	Not apply	Not apply
Sample_93	16s_sampl	694_M3_p	694_M3_p	subject694	M2	Control	Not apply	Not apply	Not apply
Sample_94	16s_sampl	695_M3_L	695_M3_L	subject695	M2	Control	Not apply	Not apply	Not apply
Sample_95	16s_sampl	794_FU3_	794_FU3_	subject794	M2	Control	Not apply	Not apply	Not apply
Sample_96	16s_sampl	795_FU3_	795_FU3_	subject795	M2	Control	Not apply	Not apply	Not apply
Sample_97	16s_sampl	801_FU3_	801_FU3_	subject801	M2	Control	Not apply	Not apply	Not apply
Sample_98	16s_sampl	807_FU3_	807_FU3_	subject807	M2	Control	Not apply	Not apply	Not apply
Sample_99	16s_sampl	809_FU3_	809_FU3_	subject809	M2	Control	Not apply	Not apply	Not apply
Sample_10	16s_sampl	830_FU3_	830_FU3_	subject830	M2	Control	Not apply	Not apply	Not apply
Sample_10	16s_sampl	833_FU3_	833_FU3_	subject833	M2	Control	Not apply	Not apply	Not apply

Sample_10	16s_sampl	837_M3_L	837_M3_L	subject837	M2	Control	Not apply	Not apply	Not apply
Sample_10	16s_sampl	851_FU3_p	851_FU3_p	subject851	M2	Control	Not apply	Not apply	Not apply
Sample_10	16s_sampl	880_M3_L	880_M3_L	subject880	M2	Control	Not apply	Not apply	Not apply
Sample_10	16s_sampl	884_M3_L	884_M3_L	subject884	M2	Control	Not apply	Not apply	Not apply
Sample_10	16s_sampl	885_M3_p	885_M3_p	subject885	M2	Control	Not apply	Not apply	Not apply
Sample_10	16s_sampl	891_M3_L	891_M3_L	subject891	M2	Control	Not apply	Not apply	Not apply
Sample_10	16s_sampl	897_M3_L	897_M3_L	subject897	M2	Control	Not apply	Not apply	Not apply
Sample_10	16s_sampl	907_M3_L	907_M3_L	subject907	M2	Control	Not apply	Not apply	Not apply
Sample_11	16s_sampl	934_M3_L	934_M3_L	subject934	M2	Control	Not apply	Not apply	Not apply
Sample_11	16s_sampl	935_FU3_p	935_FU3_p	subject935	M2	Control	Not apply	Not apply	Not apply
Sample_11	16s_sampl	P101_FU3_	P101_FU3_	subjectP10	M2	Control	Not apply	Not apply	Not apply
Sample_11	16s_sampl	P201_FU3_	P201_FU3_	subjectP20	M2	Control	Not apply	Not apply	Not apply
Sample_11	16s_sampl	P204_FU3_	P204_FU3_	subjectP20	M2	Control	Not apply	Not apply	Not apply
Sample_11	16s_sampl	3R451_FU4	3R451_FU4	subject3R4	M3	Control	Not apply	Not apply	Not apply
Sample_11	16s_sampl	3R452_FU4	3R452_FU4	subject3R4	M3	Control	Not apply	Not apply	Not apply
Sample_11	16s_sampl	3R_455_M	3R_455_M	subject3R4	M3	Control	Not apply	Not apply	Not apply
Sample_11	16s_sampl	508_FU4_p	508_FU4_p	subject508	M3	Control	Not apply	Not apply	Not apply
Sample_11	16s_sampl	509_FU4_p	509_FU4_p	subject509	M3	Control	Not apply	Not apply	Not apply
Sample_12	16s_sampl	519_M4_p	519_M4_p	subject519	M3	Control	Not apply	Not apply	Not apply
Sample_12	16s_sampl	526_FU4_p	526_FU4_p	subject526	M3	Control	Not apply	Not apply	Not apply
Sample_12	16s_sampl	527_FU4_p	527_FU4_p	subject527	M3	Control	Not apply	Not apply	Not apply
Sample_12	16s_sampl	540_M4_L	540_M4_L	subject540	M3	Control	Not apply	Not apply	Not apply
Sample_12	16s_sampl	578_M4_L	578_M4_L	subject578	M3	Control	Not apply	Not apply	Not apply
Sample_12	16s_sampl	583_FU4_p	583_FU4_p	subject583	M3	Control	Not apply	Not apply	Not apply
Sample_12	16s_sampl	649_FU4_p	649_FU4_p	subject649	M3	Control	Not apply	Not apply	Not apply
Sample_12	16s_sampl	663_M4_L	663_M4_L	subject663	M3	Control	Not apply	Not apply	Not apply
Sample_12	16s_sampl	665_M4_p	665_M4_p	subject665	M3	Control	Not apply	Not apply	Not apply
Sample_12	16s_sampl	676_M4_L	676_M4_L	subject676	M3	Control	Not apply	Not apply	Not apply
Sample_13	16s_sampl	678_M4_p	678_M4_p	subject678	M3	Control	Not apply	Not apply	Not apply
Sample_13	16s_sampl	694_M4_p	694_M4_p	subject694	M3	Control	Not apply	Not apply	Not apply
Sample_13	16s_sampl	695_M4_L	695_M4_L	subject695	M3	Control	Not apply	Not apply	Not apply
Sample_13	16s_sampl	794_FU4_p	794_FU4_p	subject794	M3	Control	Not apply	Not apply	Not apply
Sample_13	16s_sampl	795_FU4_p	795_FU4_p	subject795	M3	Control	Not apply	Not apply	Not apply
Sample_13	16s_sampl	801_FU4_p	801_FU4_p	subject801	M3	Control	Not apply	Not apply	Not apply
Sample_13	16s_sampl	807_FU4_p	807_FU4_p	subject807	M3	Control	Not apply	Not apply	Not apply
Sample_13	16s_sampl	809_FU4_p	809_FU4_p	subject809	M3	Control	Not apply	Not apply	Not apply
Sample_13	16s_sampl	830_FU4_p	830_FU4_p	subject830	M3	Control	Not apply	Not apply	Not apply
Sample_13	16s_sampl	833_FU4_p	833_FU4_p	subject833	M3	Control	Not apply	Not apply	Not apply
Sample_14	16s_sampl	837_M4_L	837_M4_L	subject837	M3	Control	Not apply	Not apply	Not apply
Sample_14	16s_sampl	851_FU4_p	851_FU4_p	subject851	M3	Control	Not apply	Not apply	Not apply
Sample_14	16s_sampl	880_M4_L	880_M4_L	subject880	M3	Control	Not apply	Not apply	Not apply
Sample_14	16s_sampl	884_M4_L	884_M4_L	subject884	M3	Control	Not apply	Not apply	Not apply
Sample_14	16s_sampl	885_M4_p	885_M4_p	subject885	M3	Control	Not apply	Not apply	Not apply
Sample_14	16s_sampl	891_M4_L	891_M4_L	subject891	M3	Control	Not apply	Not apply	Not apply
Sample_14	16s_sampl	897_M4_L	897_M4_L	subject897	M3	Control	Not apply	Not apply	Not apply
Sample_14	16s_sampl	907_M4_L	907_M4_L	subject907	M3	Control	Not apply	Not apply	Not apply
Sample_14	16s_sampl	934_M4_L	934_M4_L	subject934	M3	Control	Not apply	Not apply	Not apply
Sample_14	16s_sampl	935_FU4_p	935_FU4_p	subject935	M3	Control	Not apply	Not apply	Not apply
Sample_15	16s_sampl	P101_FU4_	P101_FU4_	subjectP10	M3	Control	Not apply	Not apply	Not apply
Sample_15	16s_sampl	P201_FU4_	P201_FU4_	subjectP20	M3	Control	Not apply	Not apply	Not apply
Sample_15	16s_sampl	P204_FU4_	P204_FU4_	subjectP20	M3	Control	Not apply	Not apply	Not apply

Sample_15	16s_sampl	3R451_FU5	3R451_FU5	subject3R4	M4	Control	Not apply	Not apply	Not apply
Sample_15	16s_sampl	3R452_FU5	3R452_FU5	subject3R4	M4	Control	Not apply	Not apply	Not apply
Sample_15	16s_sampl	3R_455_M	3R_455_M	subject3R4	M4	Control	Not apply	Not apply	Not apply
Sample_15	16s_sampl	508_FU5_	508_FU5_	subject508	M4	Control	Not apply	Not apply	Not apply
Sample_15	16s_sampl	509_FU5_	509_FU5_	subject509	M4	Control	Not apply	Not apply	Not apply
Sample_15	16s_sampl	519_M5_p	519_M5_p	subject519	M4	Control	Not apply	Not apply	Not apply
Sample_15	16s_sampl	526_FU5_	526_FU5_	subject526	M4	Control	Not apply	Not apply	Not apply
Sample_16	16s_sampl	527_FU5_	527_FU5_	subject527	M4	Control	Not apply	Not apply	Not apply
Sample_16	16s_sampl	540_M5_L	540_M5_L	subject540	M4	Control	Not apply	Not apply	Not apply
Sample_16	16s_sampl	578_M5_L	578_M5_L	subject578	M4	Control	Not apply	Not apply	Not apply
Sample_16	16s_sampl	583_FU5_	583_FU5_	subject583	M4	Control	Not apply	Not apply	Not apply
Sample_16	16s_sampl	649_FU5_	649_FU5_	subject649	M4	Control	Not apply	Not apply	Not apply
Sample_16	16s_sampl	663_M5_L	663_M5_L	subject663	M4	Control	Not apply	Not apply	Not apply
Sample_16	16s_sampl	665_M5_p	665_M5_p	subject665	M4	Control	Not apply	Not apply	Not apply
Sample_16	16s_sampl	676_M5_L	676_M5_L	subject676	M4	Control	Not apply	Not apply	Not apply
Sample_16	16s_sampl	678_M5_p	678_M5_p	subject678	M4	Control	Not apply	Not apply	Not apply
Sample_16	16s_sampl	694_M5_p	694_M5_p	subject694	M4	Control	Not apply	Not apply	Not apply
Sample_17	16s_sampl	695_M5_L	695_M5_L	subject695	M4	Control	Not apply	Not apply	Not apply
Sample_17	16s_sampl	794_FU5_	794_FU5_	subject794	M4	Control	Not apply	Not apply	Not apply
Sample_17	16s_sampl	795_FU5_	795_FU5_	subject795	M4	Control	Not apply	Not apply	Not apply
Sample_17	16s_sampl	801_FU5_	801_FU5_	subject801	M4	Control	Not apply	Not apply	Not apply
Sample_17	16s_sampl	807_FU5_	807_FU5_	subject807	M4	Control	Not apply	Not apply	Not apply
Sample_17	16s_sampl	809_FU5_	809_FU5_	subject809	M4	Control	Not apply	Not apply	Not apply
Sample_17	16s_sampl	830_FU5_	830_FU5_	subject830	M4	Control	Not apply	Not apply	Not apply
Sample_17	16s_sampl	833_FU5_	833_FU5_	subject833	M4	Control	Not apply	Not apply	Not apply
Sample_17	16s_sampl	837_M5_L	837_M5_L	subject837	M4	Control	Not apply	Not apply	Not apply
Sample_17	16s_sampl	851_FU5_	851_FU5_	subject851	M4	Control	Not apply	Not apply	Not apply
Sample_18	16s_sampl	880_M5_L	880_M5_L	subject880	M4	Control	Not apply	Not apply	Not apply
Sample_18	16s_sampl	884_M5_L	884_M5_L	subject884	M4	Control	Not apply	Not apply	Not apply
Sample_18	16s_sampl	885_M5_p	885_M5_p	subject885	M4	Control	Not apply	Not apply	Not apply
Sample_18	16s_sampl	891_M5_L	891_M5_L	subject891	M4	Control	Not apply	Not apply	Not apply
Sample_18	16s_sampl	897_M5_L	897_M5_L	subject897	M4	Control	Not apply	Not apply	Not apply
Sample_18	16s_sampl	907_M5_L	907_M5_L	subject907	M4	Control	Not apply	Not apply	Not apply
Sample_18	16s_sampl	934_M5_L	934_M5_L	subject934	M4	Control	Not apply	Not apply	Not apply
Sample_18	16s_sampl	935_FU5_	935_FU5_	subject935	M4	Control	Not apply	Not apply	Not apply
Sample_18	16s_sampl	P101_FU5_	P101_FU5_	subjectP10	M4	Control	Not apply	Not apply	Not apply
Sample_18	16s_sampl	P201_FU5_	P201_FU5_	subjectP20	M4	Control	Not apply	Not apply	Not apply
Sample_19	16s_sampl	P204_FU5_	P204_FU5_	subjectP20	M4	Control	Not apply	Not apply	Not apply
Sample_19	16s_sampl	3R451_FU6	3R451_FU6	subject3R4	M5	Control	Not apply	Not apply	Not apply
Sample_19	16s_sampl	3R452_FU6	3R452_FU6	subject3R4	M5	Control	Not apply	Not apply	Not apply
Sample_19	16s_sampl	3R_455_M	3R_455_M	subject3R4	M5	Control	Not apply	Not apply	Not apply
Sample_19	16s_sampl	508_FU6_	508_FU6_	subject508	M5	Control	Not apply	Not apply	Not apply
Sample_19	16s_sampl	509_FU6_	509_FU6_	subject509	M5	Control	Not apply	Not apply	Not apply
Sample_19	16s_sampl	519_M6_p	519_M6_p	subject519	M5	Control	Not apply	Not apply	Not apply
Sample_19	16s_sampl	526_FU6_	526_FU6_	subject526	M5	Control	Not apply	Not apply	Not apply
Sample_19	16s_sampl	527_FU6_	527_FU6_	subject527	M5	Control	Not apply	Not apply	Not apply
Sample_19	16s_sampl	540_M6_L	540_M6_L	subject540	M5	Control	Not apply	Not apply	Not apply
Sample_20	16s_sampl	578_M6_L	578_M6_L	subject578	M5	Control	Not apply	Not apply	Not apply
Sample_20	16s_sampl	583_FU6_	583_FU6_	subject583	M5	Control	Not apply	Not apply	Not apply
Sample_20	16s_sampl	649_FU6_	649_FU6_	subject649	M5	Control	Not apply	Not apply	Not apply
Sample_20	16s_sampl	663_M6_L	663_M6_L	subject663	M5	Control	Not apply	Not apply	Not apply

Sample_20	16s_sampl	665_M6_p	665_M6_p	subject665	M5	Control	Not apply	Not apply	Not apply
Sample_20	16s_sampl	676_M6_L	676_M6_L	subject676	M5	Control	Not apply	Not apply	Not apply
Sample_20	16s_sampl	678_M6_p	678_M6_p	subject678	M5	Control	Not apply	Not apply	Not apply
Sample_20	16s_sampl	694_M6_p	694_M6_p	subject694	M5	Control	Not apply	Not apply	Not apply
Sample_20	16s_sampl	695_M6_L	695_M6_L	subject695	M5	Control	Not apply	Not apply	Not apply
Sample_20	16s_sampl	794_FU6_	794_FU6_	subject794	M5	Control	Not apply	Not apply	Not apply
Sample_21	16s_sampl	795_FU6_	795_FU6_	subject795	M5	Control	Not apply	Not apply	Not apply
Sample_21	16s_sampl	801_FU6_	801_FU6_	subject801	M5	Control	Not apply	Not apply	Not apply
Sample_21	16s_sampl	807_FU6_	807_FU6_	subject807	M5	Control	Not apply	Not apply	Not apply
Sample_21	16s_sampl	809_FU6_	809_FU6_	subject809	M5	Control	Not apply	Not apply	Not apply
Sample_21	16s_sampl	830_FU6_	830_FU6_	subject830	M5	Control	Not apply	Not apply	Not apply
Sample_21	16s_sampl	833_FU6_	833_FU6_	subject833	M5	Control	Not apply	Not apply	Not apply
Sample_21	16s_sampl	837_M6_L	837_M6_L	subject837	M5	Control	Not apply	Not apply	Not apply
Sample_21	16s_sampl	851_FU6_	851_FU6_	subject851	M5	Control	Not apply	Not apply	Not apply
Sample_21	16s_sampl	880_M6_L	880_M6_L	subject880	M5	Control	Not apply	Not apply	Not apply
Sample_21	16s_sampl	884_M6_L	884_M6_L	subject884	M5	Control	Not apply	Not apply	Not apply
Sample_22	16s_sampl	885_M6_p	885_M6_p	subject885	M5	Control	Not apply	Not apply	Not apply
Sample_22	16s_sampl	891_M6_L	891_M6_L	subject891	M5	Control	Not apply	Not apply	Not apply
Sample_22	16s_sampl	897_M6_L	897_M6_L	subject897	M5	Control	Not apply	Not apply	Not apply
Sample_22	16s_sampl	907_M6_L	907_M6_L	subject907	M5	Control	Not apply	Not apply	Not apply
Sample_22	16s_sampl	934_M6_L	934_M6_L	subject934	M5	Control	Not apply	Not apply	Not apply
Sample_22	16s_sampl	935_FU6_	935_FU6_	subject935	M5	Control	Not apply	Not apply	Not apply
Sample_22	16s_sampl	P101_FU6_	P101_FU6_	subjectP10	M5	Control	Not apply	Not apply	Not apply
Sample_22	16s_sampl	P201_FU6_	P201_FU6_	subjectP20	M5	Control	Not apply	Not apply	Not apply
Sample_22	16s_sampl	P204_FU6_	P204_FU6_	subjectP20	M5	Control	Not apply	Not apply	Not apply
Sample_22	16s_sampl	3R443_M3	3R443_M3	subject443	M0	interventic	NR	R	NR
Sample_23	16s_sampl	3R474_M3	3R474_M3	subject474	M0	interventic	NR	R	NR
Sample_23	16s_sampl	3R478_M3	3R478_M3	subject478	M0	interventic	NR	NR	R
Sample_23	16s_sampl	3R479_M3	3R479_M3	subject479	M0	interventic	NR	NR	R
Sample_23	16s_sampl	3R481_M3	3R481_M3	subject481	M0	interventic	NR	NR	R
Sample_23	16s_sampl	3R482_M3	3R482_M3	subject482	M0	interventic	NR	NR	NR
Sample_23	16s_sampl	3R486_M3	3R486_M3	subject486	M0	interventic	NR	R	R
Sample_23	16s_sampl	3R487_M3	3R487_M3	subject487	M0	interventic	R	R	R
Sample_23	16s_sampl	3R488_M3	3R488_M3	subject488	M0	interventic	R	R	NR
Sample_23	16s_sampl	3R492_M3	3R492_M3	subject492	M0	interventic	NR	R	NR
Sample_23	16s_sampl	3R493_M3	3R493_M3	subject493	M0	interventic	NR	R	R
Sample_24	16s_sampl	3R495_M3	3R495_M3	subject495	M0	interventic	NR	R	NR
Sample_24	16s_sampl	3R496_M3	3R496_M3	subject496	M0	interventic	NR	NR	NR
Sample_24	16s_sampl	3R506_M3	3R506_M3	subject506	M0	interventic	NR	NR	NR
Sample_24	16s_sampl	3R512_M3	3R512_M3	subject512	M0	interventic	NR	NR	R
Sample_24	16s_sampl	863_M1_L	863_M1_L	subject863	M0	interventic	NR	R	R
Sample_24	16s_sampl	P10_M1_p	P10_M1_p	subject10	M0	interventic	R	R	R
Sample_24	16s_sampl	P115_M1_	P115_M1_	subjectP11	M0	interventic	NR	R	R
Sample_24	16s_sampl	P11_M3_p	P11_M3_p	subject11	M0	interventic	NR	NR	R
Sample_24	16s_sampl	P121_M1_	P121_M1_	subjectP12	M0	interventic	NR	R	NR
Sample_24	16s_sampl	P125_M1_	P125_M1_	subjectP12	M0	interventic	NR	R	R
Sample_25	16s_sampl	P127_M1_	P127_M1_	subjectP12	M0	interventic	NR	R	R
Sample_25	16s_sampl	P129_M1_	P129_M1_	subjectP12	M0	interventic	NR	R	R
Sample_25	16s_sampl	P13_M3_p	P13_M3_p	subjectP13	M0	interventic	NR	NR	R
Sample_25	16s_sampl	P13_M7_p	P13_M7_p	subject13	M0	interventic	NR	R	R
Sample_25	16s_sampl	P140_M1_	P140_M1_	subjectP14	M0	interventic	NR	NR	NR

Sample_25	16s_sampl	P143_M1_	P143_M1_	subjectP14	M0		interventic	R	R	R
Sample_25	16s_sampl	P145_M1_	P145_M1_	subjectP14	M0		interventic	R	R	R
Sample_25	16s_sampl	P147_M1_	P147_M1_	subjectP14	M0		interventic	R	R	NR
Sample_25	16s_sampl	P149_M1_	P149_M1_	subjectP14	M0		interventic	NR	R	R
Sample_25	16s_sampl	P150_M1_	P150_M1_	subjectP15	M0		interventic	R	NR	NR
Sample_26	16s_sampl	P16_M3_p	P16_M3_p	subject16	M0		interventic	NR	R	R
Sample_26	16s_sampl	P18_M3_p	P18_M3_p	subject18	M0		interventic	NR	NR	NR
Sample_26	16s_sampl	P19_M3_p	P19_M3_p	subject19	M0		interventic	NR	NR	R
Sample_26	16s_sampl	P1_M3_ph	P1_M3_ph	subject1	M0		interventic	NR	NR	NR
Sample_26	16s_sampl	P2_M3_ph	P2_M3_ph	subject2	M0		interventic	R	R	R
Sample_26	16s_sampl	P6_M3_ph	P6_M3_ph	subject6	M0		interventic	NR	R	R
Sample_26	16s_sampl	P7_M3_ph	P7_M3_ph	subject7	M0		interventic	R	R	R
Sample_26	16s_sampl	3R443_M4	3R443_M4	subject443	M1		interventic	NR	R	NR
Sample_26	16s_sampl	3R474_M4	3R474_M4	subject474	M1		interventic	NR	R	NR
Sample_26	16s_sampl	3R478_M4	3R478_M4	subject478	M1		interventic	NR	NR	R
Sample_27	16s_sampl	3R481_M4	3R481_M4	subject481	M1		interventic	NR	NR	R
Sample_27	16s_sampl	3R482_M4	3R482_M4	subject482	M1		interventic	NR	NR	NR
Sample_27	16s_sampl	3R486_M4	3R486_M4	subject486	M1		interventic	NR	R	R
Sample_27	16s_sampl	3R487_M4	3R487_M4	subject487	M1		interventic	R	R	R
Sample_27	16s_sampl	3R488_M4	3R488_M4	subject488	M1		interventic	R	R	NR
Sample_27	16s_sampl	3R492_M4	3R492_M4	subject492	M1		interventic	NR	R	NR
Sample_27	16s_sampl	3R493_M4	3R493_M4	subject493	M1		interventic	NR	R	R
Sample_27	16s_sampl	3R495_M4	3R495_M4	subject495	M1		interventic	NR	R	NR
Sample_27	16s_sampl	3R496_M4	3R496_M4	subject496	M1		interventic	NR	NR	NR
Sample_27	16s_sampl	3R506_M4	3R506_M4	subject506	M1		interventic	NR	NR	NR
Sample_28	16s_sampl	3R512_M4	3R512_M4	subject512	M1		interventic	NR	NR	R
Sample_28	16s_sampl	863_M3_L	863_M3_L	subject863	M1		interventic	NR	R	R
Sample_28	16s_sampl	P10_M4_p	P10_M4_p	subject10	M1		interventic	R	R	R
Sample_28	16s_sampl	P115_M3_	P115_M3_	subjectP11	M1		interventic	NR	R	R
Sample_28	16s_sampl	P11_M4_p	P11_M4_p	subject11	M1		interventic	NR	NR	R
Sample_28	16s_sampl	P121_M3_	P121_M3_	subjectP12	M1		interventic	NR	R	NR
Sample_28	16s_sampl	P125_M3_	P125_M3_	subjectP12	M1		interventic	NR	R	R
Sample_28	16s_sampl	P127_M3_	P127_M3_	subjectP12	M1		interventic	NR	R	R
Sample_28	16s_sampl	P129_M3_	P129_M3_	subjectP12	M1		interventic	NR	R	R
Sample_28	16s_sampl	P13_M5_p	P13_M5_p	subjectP13	M1		interventic	NR	NR	R
Sample_29	16s_sampl	P134_M1_	P134_M1_	subject13	M1		interventic	NR	R	R
Sample_29	16s_sampl	P140_M3_	P140_M3_	subjectP14	M1		interventic	NR	NR	NR
Sample_29	16s_sampl	P143_M3_	P143_M3_	subjectP14	M1		interventic	R	R	R
Sample_29	16s_sampl	P145_M3_	P145_M3_	subjectP14	M1		interventic	R	R	R
Sample_29	16s_sampl	P147_M3_	P147_M3_	subjectP14	M1		interventic	R	R	NR
Sample_29	16s_sampl	P149_M3_	P149_M3_	subjectP14	M1		interventic	NR	R	R
Sample_29	16s_sampl	P150_M3_	P150_M3_	subjectP15	M1		interventic	R	NR	NR
Sample_29	16s_sampl	P16_M4_p	P16_M4_p	subject16	M1		interventic	NR	R	R
Sample_29	16s_sampl	P18_M4_p	P18_M4_p	subject18	M1		interventic	NR	NR	NR
Sample_29	16s_sampl	P19_M4_p	P19_M4_p	subject19	M1		interventic	NR	NR	R
Sample_30	16s_sampl	P1_M4_ph	P1_M4_ph	subject1	M1		interventic	NR	NR	NR
Sample_30	16s_sampl	P2_M4_ph	P2_M4_ph	subject2	M1		interventic	R	R	R
Sample_30	16s_sampl	P6_M4_ph	P6_M4_ph	subject6	M1		interventic	NR	R	R
Sample_30	16s_sampl	P7_M4_ph	P7_M4_ph	subject7	M1		interventic	R	R	R
Sample_30	16s_sampl	3R443_M5	3R443_M5	subject443	M2		interventic	NR	R	NR
Sample_30	16s_sampl	3R474_M5	3R474_M5	subject474	M2		interventic	NR	R	NR

Sample_30	16s_sampl	3R478_M5	3R478_M5	subject478	M2	interventic	NR	NR	R
Sample_30	16s_sampl	3R479_M5	3R479_M5	subject479	M2	interventic	NR	NR	R
Sample_30	16s_sampl	3R481_M5	3R481_M5	subject481	M2	interventic	NR	NR	R
Sample_30	16s_sampl	3R482_M5	3R482_M5	subject482	M2	interventic	NR	NR	NR
Sample_31	16s_sampl	3R486_M5	3R486_M5	subject486	M2	interventic	NR	R	R
Sample_31	16s_sampl	3R487_M5	3R487_M5	subject487	M2	interventic	R	R	R
Sample_31	16s_sampl	3R488_M5	3R488_M5	subject488	M2	interventic	R	R	NR
Sample_31	16s_sampl	3R492_M5	3R492_M5	subject492	M2	interventic	NR	R	NR
Sample_31	16s_sampl	3R493_M5	3R493_M5	subject493	M2	interventic	NR	R	R
Sample_31	16s_sampl	3R495_M5	3R495_M5	subject495	M2	interventic	NR	R	NR
Sample_31	16s_sampl	3R496_M5	3R496_M5	subject496	M2	interventic	NR	NR	NR
Sample_31	16s_sampl	3R506_M5	3R506_M5	subject506	M2	interventic	NR	NR	NR
Sample_31	16s_sampl	3R512_M5	3R512_M5	subject512	M2	interventic	NR	NR	R
Sample_31	16s_sampl	863_M2_L	863_M2_L	subject863	M2	interventic	NR	R	R
Sample_32	16s_sampl	P10_M5_p	P10_M5_p	subject10	M2	interventic	R	R	R
Sample_32	16s_sampl	P115_M2_	P115_M2_	subjectP11	M2	interventic	NR	R	R
Sample_32	16s_sampl	P11_M5_p	P11_M5_p	subject11	M2	interventic	NR	NR	R
Sample_32	16s_sampl	P121_M2_	P121_M2_	subjectP12	M2	interventic	NR	R	NR
Sample_32	16s_sampl	P125_M2_	P125_M2_	subjectP12	M2	interventic	NR	R	R
Sample_32	16s_sampl	P127_M2_	P127_M2_	subjectP12	M2	interventic	NR	R	R
Sample_32	16s_sampl	P129_M2_	P129_M2_	subjectP12	M2	interventic	NR	R	R
Sample_32	16s_sampl	P13_M4_p	P13_M4_p	subjectP13	M2	interventic	NR	NR	R
Sample_32	16s_sampl	P134_M2_	P134_M2_	subject13	M2	interventic	NR	R	R
Sample_32	16s_sampl	P140_M2_	P140_M2_	subjectP14	M2	interventic	NR	NR	NR
Sample_33	16s_sampl	P143_M2_	P143_M2_	subjectP14	M2	interventic	R	R	R
Sample_33	16s_sampl	P145_M2_	P145_M2_	subjectP14	M2	interventic	R	R	R
Sample_33	16s_sampl	P147_M2_	P147_M2_	subjectP14	M2	interventic	R	R	NR
Sample_33	16s_sampl	P149_M2_	P149_M2_	subjectP14	M2	interventic	NR	R	R
Sample_33	16s_sampl	P150_M2_	P150_M2_	subjectP15	M2	interventic	R	NR	NR
Sample_33	16s_sampl	P16_M5_p	P16_M5_p	subject16	M2	interventic	NR	R	R
Sample_33	16s_sampl	P18_M5_p	P18_M5_p	subject18	M2	interventic	NR	NR	NR
Sample_33	16s_sampl	P19_M5_p	P19_M5_p	subject19	M2	interventic	NR	NR	R
Sample_33	16s_sampl	P1_M5_ph	P1_M5_ph	subject1	M2	interventic	NR	NR	NR
Sample_33	16s_sampl	P2_M5_ph	P2_M5_ph	subject2	M2	interventic	R	R	R
Sample_34	16s_sampl	P6_M5_ph	P6_M5_ph	subject6	M2	interventic	NR	R	R
Sample_34	16s_sampl	P7_M5_ph	P7_M5_ph	subject7	M2	interventic	R	R	R
Sample_34	16s_sampl	3R443_M6	3R443_M6	subject443	M3	interventic	NR	R	NR
Sample_34	16s_sampl	3R474_M6	3R474_M6	subject474	M3	interventic	NR	R	NR
Sample_34	16s_sampl	3R478_M6	3R478_M6	subject478	M3	interventic	NR	NR	R
Sample_34	16s_sampl	3R479_M6	3R479_M6	subject479	M3	interventic	NR	NR	R
Sample_34	16s_sampl	3R481_M6	3R481_M6	subject481	M3	interventic	NR	NR	R
Sample_34	16s_sampl	3R482_M6	3R482_M6	subject482	M3	interventic	NR	NR	NR
Sample_34	16s_sampl	3R486_M6	3R486_M6	subject486	M3	interventic	NR	R	R
Sample_34	16s_sampl	3R487_M6	3R487_M6	subject487	M3	interventic	R	R	R
Sample_35	16s_sampl	3R488_M6	3R488_M6	subject488	M3	interventic	R	R	NR
Sample_35	16s_sampl	3R492_M6	3R492_M6	subject492	M3	interventic	NR	R	NR
Sample_35	16s_sampl	3R493_M6	3R493_M6	subject493	M3	interventic	NR	R	R
Sample_35	16s_sampl	3R495_M6	3R495_M6	subject495	M3	interventic	NR	R	NR
Sample_35	16s_sampl	3R496_M6	3R496_M6	subject496	M3	interventic	NR	NR	NR
Sample_35	16s_sampl	3R506_M6	3R506_M6	subject506	M3	interventic	NR	NR	NR
Sample_35	16s_sampl	3R512_M6	3R512_M6	subject512	M3	interventic	NR	NR	R

Sample_35	16s_sampl	863_M4_L	863_M4_L	subject863	M3	interventic	NR	R	R
Sample_35	16s_sampl	P10_M6_p	P10_M6_p	subject10	M3	interventic	R	R	R
Sample_35	16s_sampl	P115_M4_	P115_M4_	subjectP11	M3	interventic	NR	R	R
Sample_36	16s_sampl	P121_M4_	P121_M4_	subjectP12	M3	interventic	NR	R	NR
Sample_36	16s_sampl	P125_M4_	P125_M4_	subjectP12	M3	interventic	NR	R	R
Sample_36	16s_sampl	P127_M4_	P127_M4_	subjectP12	M3	interventic	NR	R	R
Sample_36	16s_sampl	P129_M4_	P129_M4_	subjectP12	M3	interventic	NR	R	R
Sample_36	16s_sampl	P13_M6_p	P13_M6_p	subjectP13	M3	interventic	NR	NR	R
Sample_36	16s_sampl	P134_M3_	P134_M3_	subject13	M3	interventic	NR	R	R
Sample_36	16s_sampl	P140_M4_	P140_M4_	subjectP14	M3	interventic	NR	NR	NR
Sample_36	16s_sampl	P143_M4_	P143_M4_	subjectP14	M3	interventic	R	R	R
Sample_36	16s_sampl	P145_M4_	P145_M4_	subjectP14	M3	interventic	R	R	R
Sample_36	16s_sampl	P147_M4_	P147_M4_	subjectP14	M3	interventic	R	R	NR
Sample_37	16s_sampl	P149_M4_	P149_M4_	subjectP14	M3	interventic	NR	R	R
Sample_37	16s_sampl	P150_M4_	P150_M4_	subjectP15	M3	interventic	R	NR	NR
Sample_37	16s_sampl	P16_M6_p	P16_M6_p	subject16	M3	interventic	NR	R	R
Sample_37	16s_sampl	P18_M6_p	P18_M6_p	subject18	M3	interventic	NR	NR	NR
Sample_37	16s_sampl	P19_M6_p	P19_M6_p	subject19	M3	interventic	NR	NR	R
Sample_37	16s_sampl	P1_M6_ph	P1_M6_ph	subject1	M3	interventic	NR	NR	NR
Sample_37	16s_sampl	P2_M6_ph	P2_M6_ph	subject2	M3	interventic	R	R	R
Sample_37	16s_sampl	P6_M6_ph	P6_M6_ph	subject6	M3	interventic	NR	R	R
Sample_37	16s_sampl	P7_M6_ph	P7_M6_ph	subject7	M3	interventic	R	R	R
Sample_37	16s_sampl	3R443_M7	3R443_M7	subject443	M4	interventic	NR	R	NR
Sample_38	16s_sampl	3R474_M7	3R474_M7	subject474	M4	interventic	NR	R	NR
Sample_38	16s_sampl	3R478_M7	3R478_M7	subject478	M4	interventic	NR	NR	R
Sample_38	16s_sampl	3R479_M7	3R479_M7	subject479	M4	interventic	NR	NR	R
Sample_38	16s_sampl	3R481_M7	3R481_M7	subject481	M4	interventic	NR	NR	R
Sample_38	16s_sampl	3R482_M7	3R482_M7	subject482	M4	interventic	NR	NR	NR
Sample_38	16s_sampl	3R486_M7	3R486_M7	subject486	M4	interventic	NR	R	R
Sample_38	16s_sampl	3R487_M7	3R487_M7	subject487	M4	interventic	R	R	R
Sample_38	16s_sampl	3R492_M7	3R492_M7	subject492	M4	interventic	NR	R	NR
Sample_38	16s_sampl	3R493_M7	3R493_M7	subject493	M4	interventic	NR	R	R
Sample_38	16s_sampl	3R495_M7	3R495_M7	subject495	M4	interventic	NR	R	NR
Sample_39	16s_sampl	3R496_M7	3R496_M7	subject496	M4	interventic	NR	NR	NR
Sample_39	16s_sampl	3R506_M7	3R506_M7	subject506	M4	interventic	NR	NR	NR
Sample_39	16s_sampl	3R512_M7	3R512_M7	subject512	M4	interventic	NR	NR	R
Sample_39	16s_sampl	P10_M7_p	P10_M7_p	subject10	M4	interventic	R	R	R
Sample_39	16s_sampl	P134_M4_	P134_M4_	subject13	M4	interventic	NR	R	R
Sample_39	16s_sampl	P16_M7_p	P16_M7_p	subject16	M4	interventic	NR	R	R
Sample_39	16s_sampl	P18_M7_p	P18_M7_p	subject18	M4	interventic	NR	NR	NR
Sample_39	16s_sampl	P1_M7_ph	P1_M7_ph	subject1	M4	interventic	NR	NR	NR
Sample_39	16s_sampl	P2_M7_ph	P2_M7_ph	subject2	M4	interventic	R	R	R
Sample_39	16s_sampl	P6_M7_ph	P6_M7_ph	subject6	M4	interventic	NR	R	R
Sample_40	16s_sampl	P7_M7_ph	P7_M7_ph	subject7	M4	interventic	R	R	R
Sample_40	16s_sampl	3R474_M8	3R474_M8	subject474	M5	interventic	NR	R	NR
Sample_40	16s_sampl	3R478_M8	3R478_M8	subject478	M5	interventic	NR	NR	R
Sample_40	16s_sampl	3R479_M8	3R479_M8	subject479	M5	interventic	NR	NR	R
Sample_40	16s_sampl	3R481_M8	3R481_M8	subject481	M5	interventic	NR	NR	R
Sample_40	16s_sampl	3R482_M8	3R482_M8	subject482	M5	interventic	NR	NR	NR
Sample_40	16s_sampl	3R487_M8	3R487_M8	subject487	M5	interventic	R	R	R
Sample_40	16s_sampl	3R488_M8	3R488_M8	subject488	M5	interventic	R	R	NR

Sample_40	16s_sampl	3R495_M8	3R495_M8	subject495	M5		interventic	NR	R	NR
Sample_40	16s_sampl	3R496_M8	3R496_M8	subject496	M5		interventic	NR	NR	NR
Sample_41	16s_sampl	3R506_M8	3R506_M8	subject506	M5		interventic	NR	NR	NR
Sample_41	16s_sampl	3R512_M8	3R512_M8	subject512	M5		interventic	NR	NR	R
Sample_41	16s_sampl	P10_M8_p	P10_M8_p	subject10	M5		interventic	R	R	R
Sample_41	16s_sampl	P16_M8_p	P16_M8_p	subject16	M5		interventic	NR	R	R
Sample_41	16s_sampl	P18_M8_p	P18_M8_p	subject18	M5		interventic	NR	NR	NR
Sample_41	16s_sampl	P19_M8_p	P19_M8_p	subject19	M5		interventic	NR	NR	R
Sample_41	16s_sampl	P2_M8_ph	P2_M8_ph	subject2	M5		interventic	R	R	R
Sample_41	16s_sampl	P6_M8_ph	P6_M8_ph	subject6	M5		interventic	NR	R	R
Sample_41	16s_sampl	P7_M8_ph	P7_M8_ph	subject7	M5		interventic	R	R	R
